# Integrated single-cell multi-omics characterization reveals lipid-associated macrophage-mediated immunosuppression in neoadjuvant immunotherapy of hepatocellular carcinoma

**DOI:** 10.1038/s41467-026-75949-y

**Published:** 2026-07-31

**Authors:** Shengxuan Peng, Chang Liu, Junyu Long, Jincheng Tian, Han Li, Donghai Lu, Qihang Cao, Daolin Zhang, Qiao He, Jisen Jia, Yuxuan Wang, Zhaoru Dong, Haitao Zhao, Lei Zhao, Dongxu Wang, Tao Li

**Affiliations:** 1 Department of General Surgery, Qilu Hospital of Shandong University, Jinan, PR China; 2 Department of Liver Surgery, State Key Laboratory of Complex Severe and Rare Diseases, Peking Union Medical College Hospital, Chinese Academy of Medical Sciences and Peking Union Medical College (CAMS & PUMC), Beijing, PR China; 3 Department of Hepatopancreatobiliary Surgery, Affiliated Tumor Hospital of Xinjiang Medical University, Urumqi, PR China; 4 Department of HPB Surgery, Shandong Cancer Hospital Affiliated to Shandong First Medical University and Shandong Academy of Medical Science, Jinan, PR China

**Keywords:** Hepatocellular carcinoma, Cancer immunotherapy, Immunosurveillance, Cancer microenvironment, Monocytes and macrophages

## Abstract

Hepatocellular carcinoma (HCC) is a cancer with high incidence and mortality rate. Although immune checkpoint inhibitors (ICIs) improved survival outcomes for HCC patients, limited objective response rate highlights the urgency of investigating determinants of immunotherapy. Here, we explore HCC resistance mechanisms following neoadjuvant αPD-1 immunotherapy by constructing a comprehensive multi-modal single-cell transcriptomic atlas consisting of 14 HCC patients treated with αPD-1 from our cohort (ClinicalTrials.gov ID: NCT06571396) and 60 external HCC cases with heterogeneous treatment backgrounds. Supervised by clinical outcomes of our cohort, we identify positive and negative regulators of immunotherapy within the tumor immune microenvironment (TIME), especially lipid-associated macrophages (LAM) with increased lipid metabolic state in non-responders and characterized by *C1QA*, *FABP1*, and *APOA1* expression. We further show the presence, exogenous inducements and immunosuppressive functions of LAM, along with regulation strategies of its lipid-associated condition, including lycopene and chiglitazar. Furthermore, we construct interaction networks of immune regulators across responders and non-responders, showing distinct ligand-receptor landscapes with intervention targets. We reveal the TIME components including immunosuppressive LAMs that influence immunotherapy outcomes, thus providing evidence and insights for exploring immune landscape and therapeutic strategies for HCC immunotherapy. ClinicalTrials.gov ID: NCT06571396

## Abstract (web summary)

Neoadjuvant Immunotherapy of hepatocellular carcinoma (HCC) has shown some clinical success. Here the authors look at the underlying immune cell changes after neoadjuvant immunotherapy in HCC implicating lipid associated macrophage involvement in non-responders.

## Introduction

Hepatocellular carcinoma (HCC) ranks as the third leading cause of cancer-related mortality^1^, with most patients diagnosed at intermediate to advanced stages^2^. Even following the surgical resection of tumor lesions, a considerable proportion of patients experience recurrence or metastasis within five years, resulting in compromised five-year survival rates^3^. Consequently, the high incidence and mortality of HCC impose a substantial burden on global health. In response to this grim scenario, the clinical application of immune checkpoint inhibitors (ICIs), such as PD-1 and CTLA-4 inhibitors, has provided new therapeutic options for HCC and other malignancies. Notably, clinical trials including IMbrave150^4^, CARES-310^5^, and CheckMate 040^6,7^ have paved the way for targeted immunotherapy combinations and dual-antibody combinations, providing more options for the first-line treatment of HCC. These strategies, which include neoadjuvant and conversion immunotherapy, have collectively begun to improve overall survival among patients with advanced HCC. Nevertheless, despite the progress driven by ICI-based strategies, the objective response rates (ORR) across various immunotherapeutic approaches remain below 30%, indicating that a significant number of HCC patients failed to benefit from these treatments^8–10^. Therefore, understanding the dynamics within the tumor microenvironment (TME) during ICI treatment is crucial for optimizing therapeutic strategies. This study aims to elucidate the therapeutic pressures and alterations in the TME of HCC patients undergoing neoadjuvant immunotherapy, as well as to identify key molecules and mechanisms associated with treatment efficacy.

The rapid advances in single-cell sequencing and spatial transcriptomics have provided unprecedented insights into the complex tumor immune microenvironment, allowing researchers to elucidate the immune landscape during immunotherapy. These technologies enable the identification of critical TIME components closely associated with clinical response and prognosis, thereby offering powerful tools to intervene, modulate, and improve immunotherapeutic outcomes. Based on this strategy, a number of studies have characterized the immune microenvironment in HCC, detailing the roles of T cells^11^, neutrophils^12^, cancer-associated fibroblasts (CAF)^13^ and dendritic cells (DC)^14^, among others, and their influences on patient prognosis. Within these studies, tumor-associated macrophages (TAMs) stand out as a critical component of TIME^15,16^, as these macrophages exist in various states ranging from pro-inflammatory M1 to immunosuppressive M2 phenotypes^17^, and are widely recognized as key regulators of TIME that ultimately influence the efficacy of HCC immunotherapy. For instance, vimentin-high macrophages were demonstrated to mediate immunosuppression via an IL-1β–Treg axis^18^; MMP9^+^ TAMs were reported to facilitate progression by promoting migration, invasion, and angiogenesis in tumor cells of HCC^19^; and TREM2^+^ macrophages were involved in immunosuppressive microenvironment remodeling in HCC^20^. However, there have been relatively few studies that dissected the TIME landscape of HCC patients undergoing immunotherapy through single-cell sequencing, and accordingly pinpoint the key components affecting their clinical outcomes—particularly with respect to macrophage subtypes and their immune functions^21–23^. Additionally, although the liver is the organ most densely populated with macrophages and plays a central role in metabolic regulation, the specific influence of metabolic regulation-related TAMs on clinical outcomes of HCC patients undergoing immunotherapy has not been thoroughly explored.

In this work, we recruit 14 HCC patients who received neoadjuvant αPD-1 immunotherapy and perform single-cell sequencing on resected tumor specimens. By integrating these data with single-cell sequencing public datasets consisting of an additional 60 HCC patients, we construct a high-throughput single-cell atlas of 74 HCC patients with heterogeneous backgrounds, which enables the identification of TIME components along with corresponding subtypes. From the HCC atlas, we identify immunotherapy-related regulators by combining clinical outcomes from our cohorts with single-cell transcriptome data. In particular, we discover that the LAMs characterized by *C1QA*, *APOA1* and *FABP1* expression, are closely related to immunosuppression among HCC patients. Our further exploration and experiments demonstrate immunosuppressive functionality and the underlying regulatory strategies of LAM in HCC. Moreover, we construct a communication network that delineates the roles of multiple key components in distinguishing responders from non-responders within our immunotherapy cohort, thereby providing evidence and insights into the tumor immune landscape in HCC and informing potential intervention strategies for HCC immunotherapy.

## Results

### Establish a single-cell atlas including immunotherapy-treated HCC patients

**Fig. 1:**
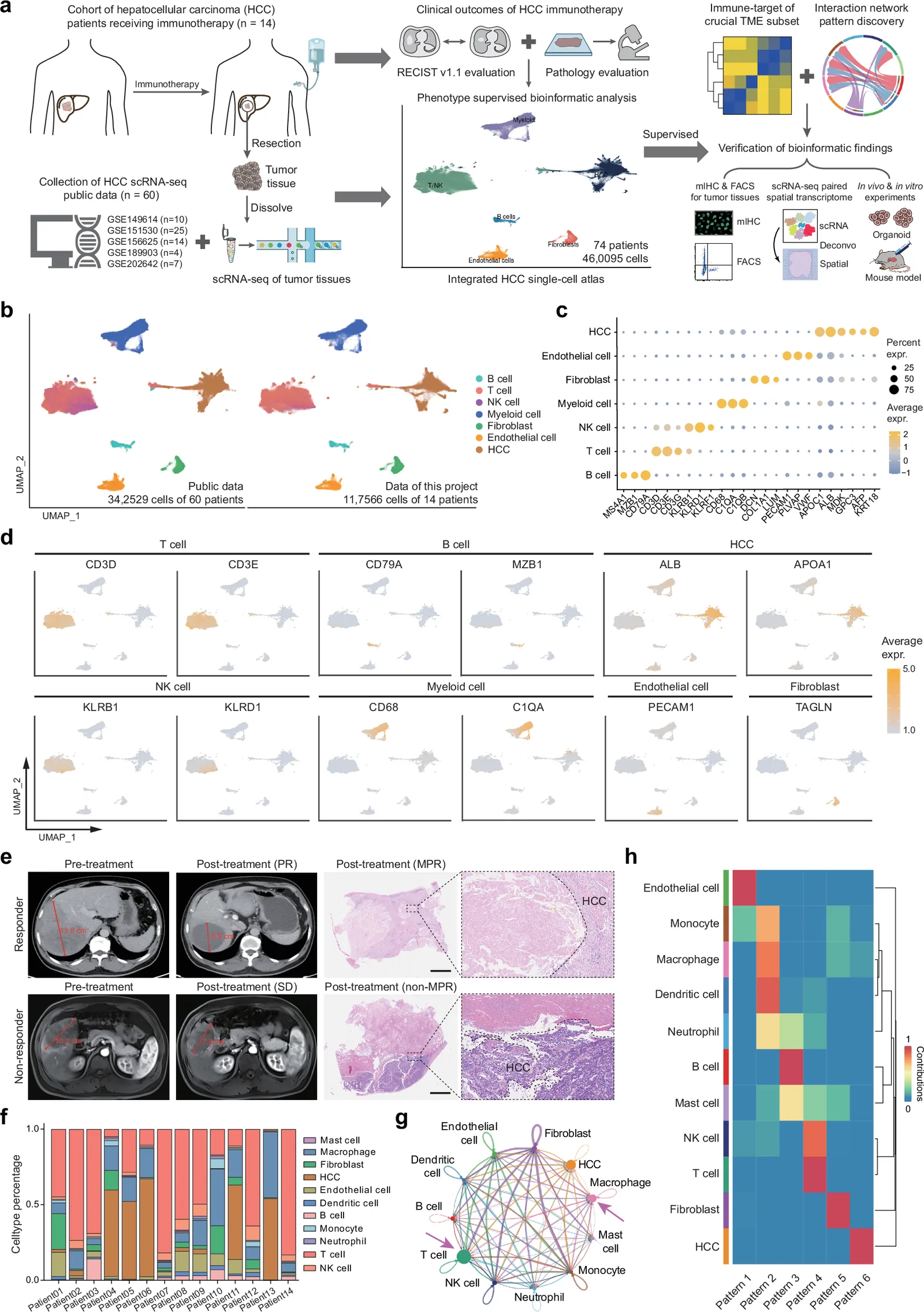
Single cell atlas integrated by public datasets of HCC patients and our HCC immunotherapy cohort with evaluation of clinical outcomes. **a** Scheme of overall study design, the single-cell atlas was integrated by five public datasets of HCC patients with heterogeneous treatment backgrounds (GSE149614, GSE151530, GSE156625, GSE189903, GSE202642) and our in-house cohort of 14 HCC patients under neoadjuvant αPD-1 immunotherapy. The following bioinformatic analysis was supervised under the clinical efficacy evaluated by histopathology, in order to determine critical TIME components and corresponding expression features related to immunotherapy. The corresponding findings were further explored and validated with mIHC visualization, flow cytometry, paired spatial transcriptome, as well as experiments based on in vivo and in vitro models. Images provided by Servier Medical Art (https://smart.servier.com), licensed under CC BY 4.0 (https://creativecommons.org/licenses/by/4.0/). **b** Uniform manifold approximation and projection (UMAP) plot of all cells colored by major cell types (B cells, T cells, NK cells, Endothelial cells, Fibroblasts, Myeloid cells and HCC) according to specific markers. **c** Major cell types identified from (**b**) and corresponding gene expression features. **d** FeaturePlot of gene markers related to (**c**) for identification of major cell types in UMAP space. **e** Typical CT images of responder and non-responder HCC patients before and after immunotherapy from our cohort, lines indicate the measured target lesion diameter. The tumor size, along with identification of partial response (PR), stable disease (SD) and progressive disease (PD) was determined by RECIST v1.1. All patients from our cohort exhibited various degrees of tumor regression after immunotherapy. Additionally, histopathological evaluation was fulfilled with H&E staining images of HCC patients under immunotherapy. The non-MPR, MPR, and CPR identification was determined by the residual tumor percentage of ≥50%, 0–50%, and 0%, respectively^28^. Scale bar: 1 cm. **f** Bar plot of cell type proportion among 14 patients of immunotherapy cohort. **g** CircosPlot exhibited cell-cell interactions analyzed by CellChat, indicating the significant functional components of TIME among our HCC immunotherapy cohort, including T cells and macrophages labeled with the arrow. **h** Heatmap of communication patterns identification from Non-negative Matrix Factorization (NMF), consistent with the major cell types determined in (**b**).

To comprehensively investigate the factors within TIME that affect the efficacy of HCC immunotherapy, we established a clinical cohort of 14 HCC patients that received αPD-1 based immunotherapy (Supplementary Table 1). Tumor samples resected after treatment were subjected to single-cell transcriptomic sequencing. To more precisely identify the cell types and corresponding subtypes of our single-cell dataset, we integrated 5 public scRNA-seq datasets^19,24–27^ consist of 60 HCC patients that not received αPD-1 treatment and mainly treatment-naïve, thus resulting in a high-throughput single-cell atlas of 74 HCC patients (Supplementary Table 2). By accurately identifying cell types and their subtypes within the atlas, we further explored and validated the critical immunoregulatory roles contributing to the diverse clinical outcomes within the TIME of patients from our HCC immunotherapy cohort (Fig. 1a and Supplementary Fig. 1a).

Based on the design above, we first established a high-throughput HCC single-cell transcriptomic atlas comprising about 460,000 cells, which integrated data from HCC immunotherapy patients in our study (14 patients, approximately 120,000 single cells) and data from public HCC single-cell datasets (60 patients, approximately 340,000 single cells). The integrated dataset was visualized via UMAP clustering (Fig. 1b, Supplementary Fig. 1b), with the identification of major cell types including HCC cells, T cells, B cells, NK cells, myeloid cells, endothelial cells and fibroblasts, along with the profiles of their characteristic gene expression (Fig. 1c, d and Supplementary Table 3), thereby confirming the accuracy of our cell type annotation. Moreover, statistical analyses of the major cell types across the five public datasets and our immunotherapy cohort revealed a relatively uniform compositions and proportions of TIME (Supplementary Fig. 1c).

We then focused on the data from our 14 patients, all of whom received αPD-1-based immunotherapy. The therapeutic efficacy was clinically assessed with CT imaging data according to RECIST v1.1 criteria, which was also regarded as the basis of surgical resection decision (Fig. 1e and Supplementary Table 1). After resection, the tumor tissues were further evaluated via H&E staining on pathological slides to quantify the residual tumor proportion, and a grading system was applied as the complete pathological response (CPR) defined with no residual tumor cell, major pathological response (MPR) for <50% residual tumor, and non-MPR was defined for the ≥50% of residual tumor, according to our local guidelines for the diagnosis and treatment of HCC^28^. For subsequent analyses in our study, patients with CPR and MPR clinical efficacies were classified as responders (R), while patients with non-MPR outcomes were classified as non-responders (NR) (Fig. 1e and Supplementary Fig. 1d), while the Kaplan–Meier survival analyses were conducted using the cutoff of 50%^28^ and 70%^29^ tumor regression for our cohort to illustrate the overall survival (OS) or progression-free survival (PFS) between the two groups (Supplementary Fig. 1e). A comparison of the cell type proportions across patients and response groups revealed that HCC cells were nearly absent in patients graded as CPR, while in non-MPR patients, HCC cells remained significantly present and were higher compared to the MPR group (Fig. 1f, Supplementary Fig. 1f, g, and Supplementary Table 3). To preliminarily evaluate TIME components that might exert a substantial impact, we performed CellChat^30^ analysis on the single-cell data from the 14 patients. The CircusPlot at the level of major cell types indicated that, aside from HCC cells, T cells and macrophages were prominently involved in intercellular communication within tumor microenvironment, suggesting a crucial role for these cell types in mediating the immune response and influencing immunotherapeutic efficacy (Fig. 1g). Additionally, non-negative matrix factorization (NMF) analysis of the TIME components within the cell-cell communication data (Fig. 1h) was consistent with the major cell type annotations via unsupervised clustering (Fig. 1b), further validating the accuracy of our single-cell atlas construction. On this basis, our subsequent analyses of the HCC immunotherapy cohort focused primarily on HCC cells, T cells, and macrophages.

### Fatty acid–associated HCC cells negatively impact immunotherapy efficacy

**Fig. 2:**
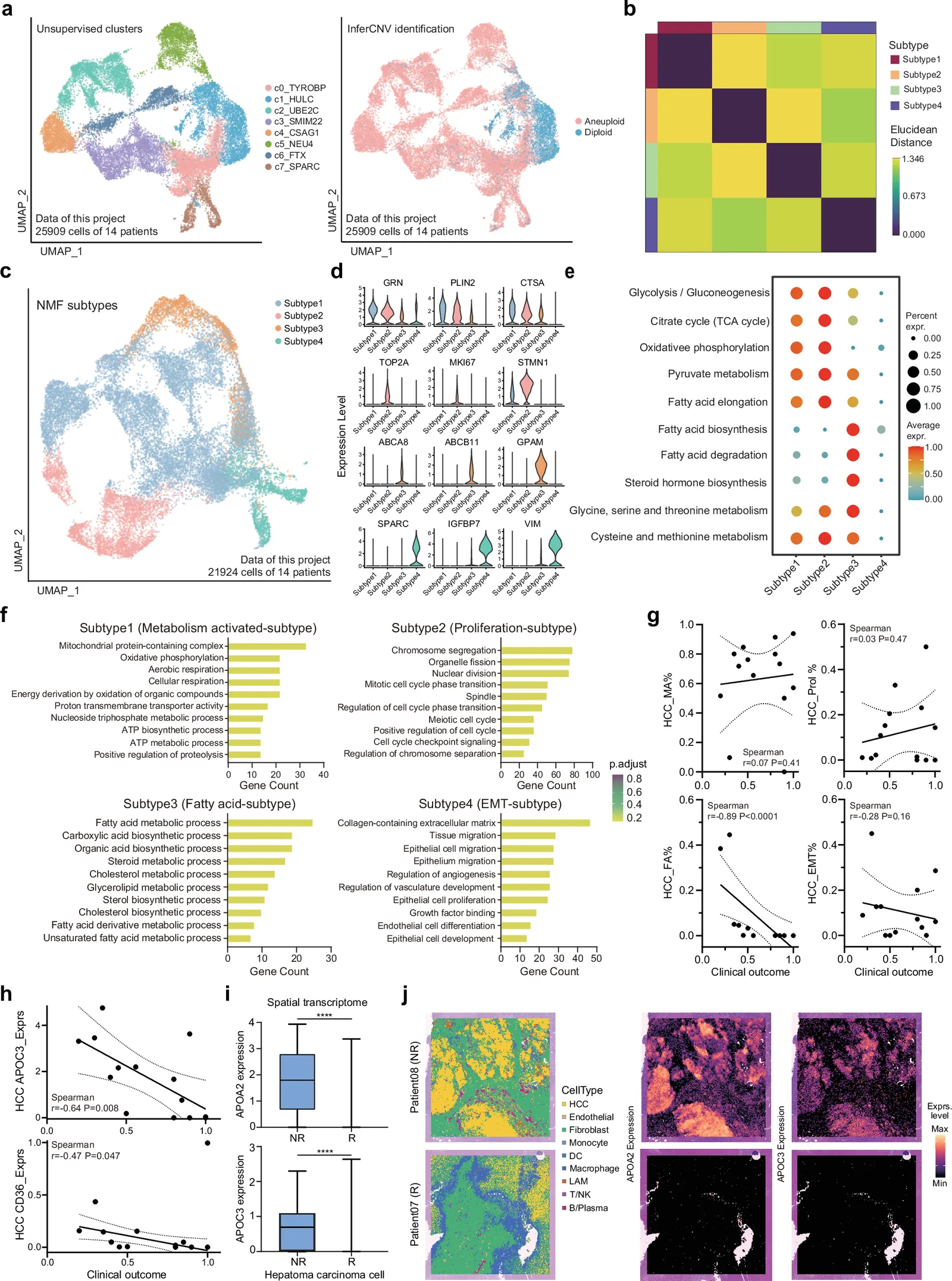
Characteristics of various HCC functional subtypes resulted in distinct clinical outcomes among responders and non-responders of immunotherapy. **a** UMAP plot of unsupervised clustering HCC cells colored by gene expression characteristics and chromosome copy number features identified by InferCNV. **b** Four functional subtypes determined by NMF from malignant HCC cells. **c** UMAP plot of malignant HCC cells colored by NMF subtypes. **d** Violin plot showing the gene expression features of four subtypes in malignant HCC cells. **e** Metabolism pathway analysis of each subtype by scMetabolism. **f** Bar charts showing the GO pathway enrichment of specific biological process based on the highly variable genes of four malignant subtypes, including metabolic active (HCC_MA), proliferation (HCC_prol), fatty acid-associated (HCC_FA) and EMT related (HCC_EMT) subtypes. **g** Spearman correlation analysis between clinical outcomes and proportion of each HCC subtype among 14 patients under immunotherapy (*n* = 14). **h** Spearman correlation analysis between clinical outcomes and normalized expression of *APOC3* and *CD36* in malignant HCC cells (*n* = 14). **i** Expression levels of *APOA2* and *APOC3* among HCC cells with different clinical outcomes in ST data that paired with scRNA-seq from six patients of our HCC cohort (NR, *n* = 108418 spots; R, *n* = 107559 spots, the *P* value is below the numerical reporting threshold of the statistical software). For box plots, the center line indicates the median, box limits indicate the 25th and 75th percentiles, and whiskers indicate the minimum and maximum values. **j** Spatial featureplots of ST data in our HCC immunotherapy cohort, showing distinct expression level of *APOA2* and *APOC3* among typical responder (Patient07) and non-responder (Patient08). (Unpaired student’s t tests, *P < 0.05, **P < 0.01, ***P < 0.001, ****P < 0.0001. Box-plot comparisons were assessed using two-sided statistical tests, while correlation analyses were assessed using one-sided Spearman correlation without multiple comparisons adjustments).

Based on our initial characterization of the immunotherapy-related components in the HCC single-cell atlas, we first analyzed HCC cells from the 14-patient cohort to assess feature subtypes therein and their correlation with immunotherapeutic efficacy. We extracted the HCC cells from the single-cell atlas corresponding to these 14 patients and performed dimensionality reduction and clustering, which yielded eight unsupervised clusters (c0–c7) in UMAP space. To more accurately isolate malignant HCC cells, we performed an InferCNV^31^ analysis on these cells to evaluate their copy number variation (CNV) profiles, thereby distinguishing aneuploid cells from diploid cells (Fig. 2a, Supplementary Fig. 2a, b, and Supplementary Table 4). After filtering out the normal diploid cells, we conducted NMF analysis, which classified the remaining malignant HCC cells into four distinct subtypes (Fig. 2b and Supplementary Fig. 2c), which also exhibited specific boundaries in the UMAP space (Fig. 2c). We then summarized the gene expression profiles of each subtype to evaluate their functional states. Subtype 1 showed high expression of *GRN* and *CTSA* features, which were associated with glucose metabolism; subtype 2 was characterized by classic proliferation markers, such as *TOP2A* and *STMN1*; subtype 3 exhibited elevated expression of lipid metabolism-related genes, including *ABCA8*, *ABCB11*, and *GPAM*, while subtype 4 was marked as the typical epithelial-to-mesenchymal transition (EMT) HCC cells (Fig. 2d, Supplementary Fig. 2d, and Supplementary Table 5). Given the pronounced metabolic features observed in many HCC cells, we further applied the scMetabolism approach^32^ to analyze the metabolic characteristics across four identified subtypes. We found that subtypes 1 and 2 were enriched in processes related to glycolysis/glycogen synthesis and oxidative phosphorylation, whereas subtype 3 was extensively involved in fatty acid synthesis, elongation, and degradation processes (Fig. 2e, Supplementary Fig. 2e, and Supplementary Table 6). The following Gene Ontology (GO) pathway enrichment of features from these four HCC subtypes allowed us to delineate their biological functions more intuitively (Fig. 2f). For instance, the gene signature of subtype 1 was significantly enriched in pathways related to oxidative phosphorylation, energy production, and metabolism, leading us to define it as a metabolically active subtype (HCC_MA). Subtype 2, with significant enrichment of cell cycle and mitosis-related pathways, was defined as a proliferation-associated subtype (HCC_prol). Subtype 3 is involved in the synthesis and metabolism of fatty acids, unsaturated fatty acids, cholesterol, and glycerolipids, and was thereby defined as a fatty acid-associated HCC subtype (HCC_FA). In contrast, subtype 4 was enriched in pathways associated with extracellular matrix remodeling and epithelial cell migration, and was accordingly defined as an EMT-related HCC subtype (HCC_EMT).

Building on these functional definitions, we further investigated the relationship between the four subtypes and immunotherapeutic efficacy within our 14-patient cohort. Spearman correlation analysis between the proportion of each HCC subtype in individual patients and their immunotherapeutic response revealed that the HCC_FA subtype was significantly negatively correlated with immunotherapeutic efficacy (R = –0.89, *P* = 0.005). This significant relationship was not observed for the other three subtypes (Fig. 2g). In addition, the expression levels of features related to lipid transport and fatty acid metabolism, including *APOC3* and *CD36*, were also significantly negatively correlated with clinical responses (Fig. 2h). These findings suggest that the presence of HCC cells with pronounced lipid—particularly fatty acid—metabolic features exerts a negative impact on the efficacy of immunotherapy. To further validate this observation, we explored our paired spatial transcriptomics data, along with the external single-cell and spatial transcriptomics datasets from HCC immunotherapy studies^14,33^. Consistently, in these paired and independent datasets, HCC cells from non-responders exhibited significantly higher expression of lipid-associated markers than those from responders (Fig. 2i, j and Supplementary Fig. 2f, g), which further supports the conclusion that fatty acid metabolism-associated subtypes negatively influence clinical immunotherapeutic outcomes of HCC patients.

### Effector and cytotoxic T cells with less exhaustion collectively improved immunotherapeutic responses

**Fig. 3:**
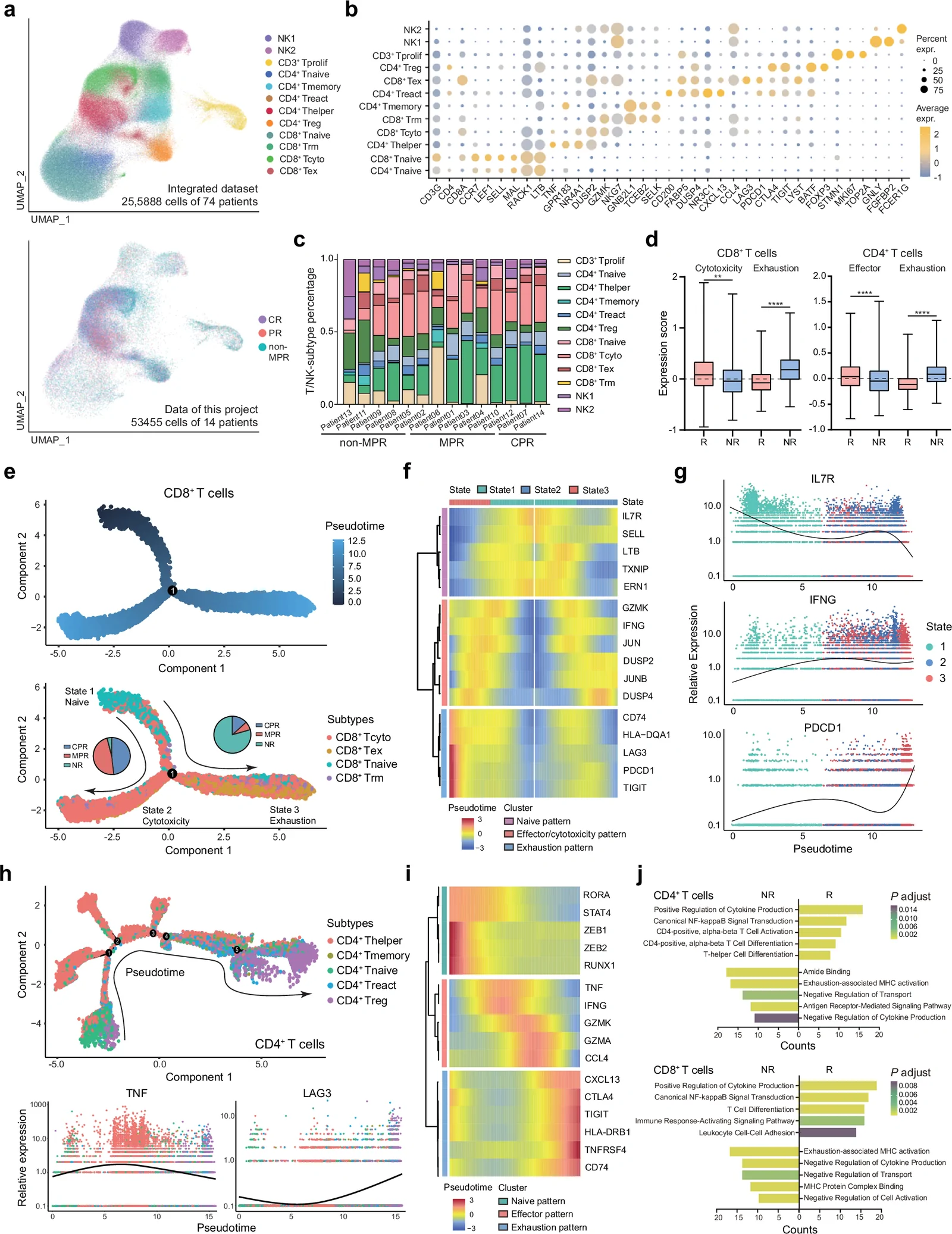
Developmental trajectory of various CD4^+^ and CD8^+^ T cell subtypes revealed diverse immunotherapy outcomes among responders and non-responders. **a** UMAP plots of 12 T/NK cell subtypes from the integrated single-cell atlas and our HCC immunotherapy cohort labeled with clinical efficiency. **b** Gene expression features of 12 T/NK subtypes. **c** Bar plots indicating the proportion of T/NK subtypes among 14 patients. **d** Box plots exhibited the average expression of cytotoxic, effector and exhausted signature scores for CD8^+^ (R, *n* = 11233 cells; NR, *n* = 9445 cells) and CD4^+^ T cells (R, *n* = 16727 cells; NR, *n* = 9129 cells). (CD8^+^ T cytotoxic, *P* = 0.008; CD4^+^ T effector, *P* = 1.6×10^-10^; the P value of exhaustion score is below the numerical reporting threshold of the statistical software). **e** The developmental trajectory of CD8^+^ T cells from our HCC immunotherapy cohort inferred by Monocle2, labeled with value of pseudotime and subtypes. **f** Heatmap exhibited the expression features of top differential genes in CD8^+^ T cells along the pseudotime, the pseudotime values of genes in State 2 were earlier than State 3. **g** The change in expression levels of functional genes in CD8⁺ T cells along the axis of pseudotime, as *IL7R* represented naive features, *IFNG* for effector features, and *PDCD1* represented T cell exhaustion. **h** The developmental trajectory of CD4^+^ T cells from our HCC immunotherapy cohort inferred by Monocle2, labeled with five CD4^+^ T cell subtypes and the black arrow indicated the development direction of pseudotime. The scatter plots showed the change in expression levels of functional genes in CD4⁺ T cells along the axis of pseudotime, as *TNF* represented effector features and *LAG3* for T cell exhaustion. **i** Heatmap exhibited the features of top differential genes in CD4^+^ T cells along the pseudotime. **j** Bar charts showing GO pathway enrichment of specific biological process based on the differentially expressed genes in CD4^+^ T cells and CD8^+^ T cells between responders and non-responders. (For box plots, the center line indicates the median, and the lower and upper hinges represent the 25 and 75th percentiles. Unpaired two-sided Student’s *t* tests, *P < 0.05, **P < 0.01, ***P < 0.001, ****P < 0.0001).

We further explored the driving effects of immune components within the tumor microenvironment on tumor immunity and clinical outcomes—particularly focusing on the T cells targeted by αPD-1 immune checkpoint inhibitors, which were prominently featured in our intercellular communication network analysis. To this end, we further performed dimensionality reduction and clustering on T/NK cells derived from the high-throughput HCC single-cell atlas. This approach enabled us to accurately identify four clusters of CD8⁺ T cells, five clusters of CD4⁺ T cells, one cluster of proliferative CD3⁺ T cells, and two clusters of NK cells (Fig. 3a, b and Supplementary Table 7), while the consistent clustering result was recapitulated within the T/NK cells from our cohort of 14 patients, thereby validating the functional definitions of T/NK cell subtypes in HCC patients (Supplementary Fig. 3a). We then quantified the proportions of T/NK cells in our 14 patients (Fig. 3c), with a particular focus on analyzing differences in T cell features between patients exhibiting different immunotherapeutic responses. Overall, responders possessed more cytotoxic CD8⁺ T cells along with activated CD4⁺ T cells, while the state of exhaustion in both CD8⁺ and CD4⁺ T cells were less compared to non-responders (Fig. 3d and Supplementary Table 8). These findings prompted us to further interrogate the distinct features of CD8⁺ and CD4⁺ T cells in patients with diverse therapeutic outcomes.

For CD8⁺ T cells, we employed Monocle2 method^34^ to perform pseudotime analysis on cells from the 14 patients, and generated corresponding DDRTree plots to elucidate the state transitions within the tumor microenvironment (Fig. 3e). Notably, based on pseudotime structure and topological ordering, the developmental trajectory initiated from CD8⁺ T naïve (CD8⁺ Tn) cells diverged upon entering an effector state (labeled as State 1). One branch (State 2) maintained a predominantly cytotoxic effector state and was derived primarily from patients with complete or major pathological responses. In contrast, the alternative branch (State 3) rapidly adopted an exhausted feature and was predominantly populated by cells from non-responders (Supplementary Fig. 3b). This observation suggests that a lower level of exhaustion in CD8⁺ cytotoxic T cells (CD8⁺ Tcyto) is associated with favorable immunotherapeutic outcomes among HCC patients. Further assessment of gene expression along the pseudotime trajectory revealed that CD8⁺ T cells in the initial state (State 1) bore typical naïve markers (e.g., *IL7R*, *SELL*). Cells in State 2 retained robust effector features—such as up-regulation of *LTB*, *GZMK*, and *IFNG*—with less expression of exhaustion markers. Conversely, cells in State 3 significantly upregulated key exhaustion markers, including *PDCD1*, *LAG3*, and *TIGIT* (Fig. 3f, g), thereby reinforcing the molecular distinction between responsive and non-responsive CD8⁺ T cell subsets.

In contrast, the DDRTree trajectory of CD4⁺ T cells followed a linear progression: beginning with a CD4⁺ T naïve (CD4⁺ Tn) state, transitioning into a CD4⁺ T helper (CD4⁺ Th) state that predominated in responders, and ultimately dominated by CD4⁺ regulatory T cells (CD4⁺ Treg), which were the majority in non-responders. This developmental sequence was accompanied by an up-regulation of *TNF* and *IL7R* in CD4⁺ Th, followed by a notable increase in exhaustion features (e.g., *PDCD1*, *LAG3*) and Treg-specific markers (e.g., *FOXP3*) toward the terminal differentiation phase (Fig. 3h, i, Supplementary Fig. 3c). Subsequently, we conducted Monocle3^35^ pseudotime analysis for both CD8⁺ and CD4⁺ T cells in UMAP formation to cross-validate these findings from Monocle2. The results further corroborated these differential differentiation patterns between responders and non-responders (Supplementary Fig. 3d, e). Moreover, GO pathway enrichment also revealed that T cells from responders exhibited heightened NF-κB signaling–mediated effector activation and immune response characteristics, while the negative regulation pathways were prominent among non-responders (Fig. 3j). Therefore, our analyses indicate that reduced exhaustion in CD8⁺ cytotoxic T cells, coupled with an activated condition of CD4⁺ T helpers, collectively relates to an improved immunotherapeutic response in HCC patients.

### Lipid-associated macrophages correlate with poor immunotherapy outcomes

**Fig. 4:**
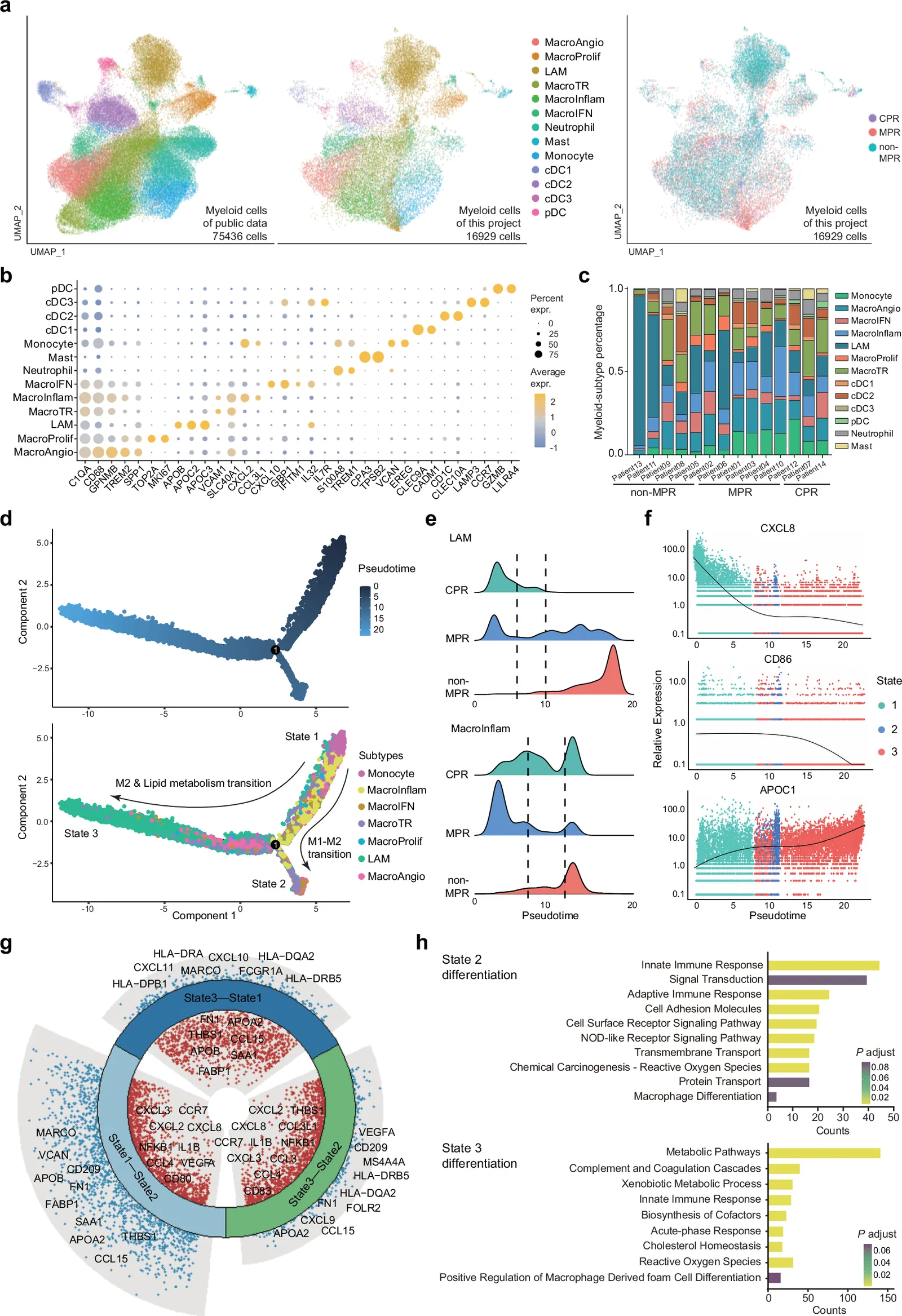
Poor clinical immunotherapy outcomes in HCC patients were significantly associated with lipid-associated macrophages. **a** UMAP plot of myeloid cells from the integrated single-cell atlas and our HCC immunotherapy cohort, colored by three types of clinical outcomes and 13 subtypes, including 6 types of macrophages. **b** Gene expression features of 13 myeloid subtypes. **c** Bar plots indicating the proportion of myeloid subtypes among 14 patients. **d** The developmental trajectory of monocytes and macrophages from our HCC immunotherapy cohort inferred by Monocle2, labeled with the value of pseudotime and myeloid subtypes. Monocytes and macrophages were the major cell types among the root of differentiation, the branch of State 2 mainly consisted of macrophages with M2 characteristics, while the subtype of LAM was predominantly located in State 3. **e** Ridge plot showing distribution of LAM and MacroInflam in different clinical efficacy groups along the axis of pseudotime. **f** Scatter plots exhibited the expression changing along the pseudotime of characteristic genes related to macrophage functions. As the differentiation progressed from State 1 to State 3, M1-associated features (*CXCL8*, *CD86*) diminished and lipid-associated marker (*APOC1*) up-regulated. **g** Volcano plot displaying differentially expressed genes across three states of monocytes and macrophages, visually represented the fold change of DEGs in macrophage clusters between different experimental groups. **h** Bar charts exhibited the enrichment of specific biological processes based on the characteristic gene expression of State 2 and State 3.

We further investigated the impact of myeloid cells—especially macrophages, which play a crucial role in modulating the tumor immune microenvironment—on immunotherapeutic efficacy. Therefore, we performed dimensionality reduction and clustering on the myeloid cells derived from the high-throughput single-cell atlas. This analysis enabled us to identify neutrophils, mast cells, monocytes, four subtypes of dendritic cells, and six macrophage subtypes with their characteristic expression profiles (Fig. 4a, amd Supplementary Fig. 4a). In particular, we delineated distinct functional states of macrophages, including subtypes associated with angiogenesis, cell proliferation, tissue residency, interferon induction, inflammation, and lipid metabolism (Fig. 4b, Supplementary Table 9). Quantitative evaluation of the composition of these myeloid subtypes in the 14 patients (Fig. 4c) revealed that a lipid-associated subtype of macrophage characterized by the expression of *C1QA*, *FABP1*, and multiple apolipoproteins (e.g., *APOA1*, *APOB*, *APOC1*), was significantly enriched in non-responders. In contrast, a subtype with high expression of *CCL3L1*, *CXCL2*, and *CXCL8*, designated inflammatory macrophages (MacroInflam), was more prevalent among responders. These findings prompted further exploration of the roles of macrophage subtypes in patients with different immunotherapeutic outcomes.

Accordingly, we performed pseudotime analysis on the monocyte-macrophage population from the 14 patients using Monocle2 and constructed a corresponding DDRTree trajectory with directionality inferred with pseudotime structure (Fig. 4d). Interestingly, we observed divergent differentiation features within the developmental trajectory initiated from monocytes (Fig. 4e, Supplementary Fig. 4c). In the early stage (State 1), monocytes transitioning into macrophages predominantly exhibited a MacroInflam subtype—resembling the classical M1-like state—which was largely represented among responders in the pseudotime trajectory. As differentiation progressed from State 1 to State 2, we observed a diminution of M1-associated features (e.g., *CD86*, *CXCL8*, *IL1B*) with a concomitant up-regulation of M2-related markers such as *ARG1* and *CD209* (Fig. 4f, g and Supplementary Fig. 4b), we refer to this transition as “M1-to-M2 polarization”. On the other side, during the transition from State 1 to State 3, macrophages rapidly differentiated into the condition with pronounced up-regulation of lipid-associated markers including *FABP1*, *APOA1*, and *APOC1*, and exhibited active fatty acid metabolism progress (Supplementary Fig. 4d). We designate this differentiation process as “M2 with lipid-associated polarization”, and observed that macrophages in State 3 were significantly dominant in non-responder patients. Meanwhile, the developmental trajectory analyzed via Monocle2 was also corroborated by subsequent Monocle3 analysis (Supplementary Fig. 4e). With pathway enrichment analyses comparing the transitions from the initial State 1 to States 2 and 3, results showed that the differentiation characteristic of State 2 was associated with immune response, signal transduction, and macrophage differentiation, whereas the transition toward State 3 was closely linked to metabolic functions, complement activation, cholesterol homeostasis, and pathways related to macrophage foam cell formation (Fig. 4h). Collectively, our analysis of myeloid cells—especially macrophages—in our HCC immunotherapy cohort revealed the existence of a lipid-associated macrophage subset with metabolically enriched state that showed up-regulated expression of lipid-metabolic genes (such as *FABP1* and *APOA1*), average *SPP1* expression and lower *TREM2* expression, which is different from traditional *SPP1*^+^ or *TREM2*^+^ macrophages. Based on its significant association with poor clinical efficacy, we shall further explore the detailed relationship between this LAM subset and HCC immunotherapy, together with the mechanisms driving their formation and function.

### Global correlation analysis combined clinical efficacy with single-cell transcriptome reveals key regulators of immunotherapy

**Fig. 5:**
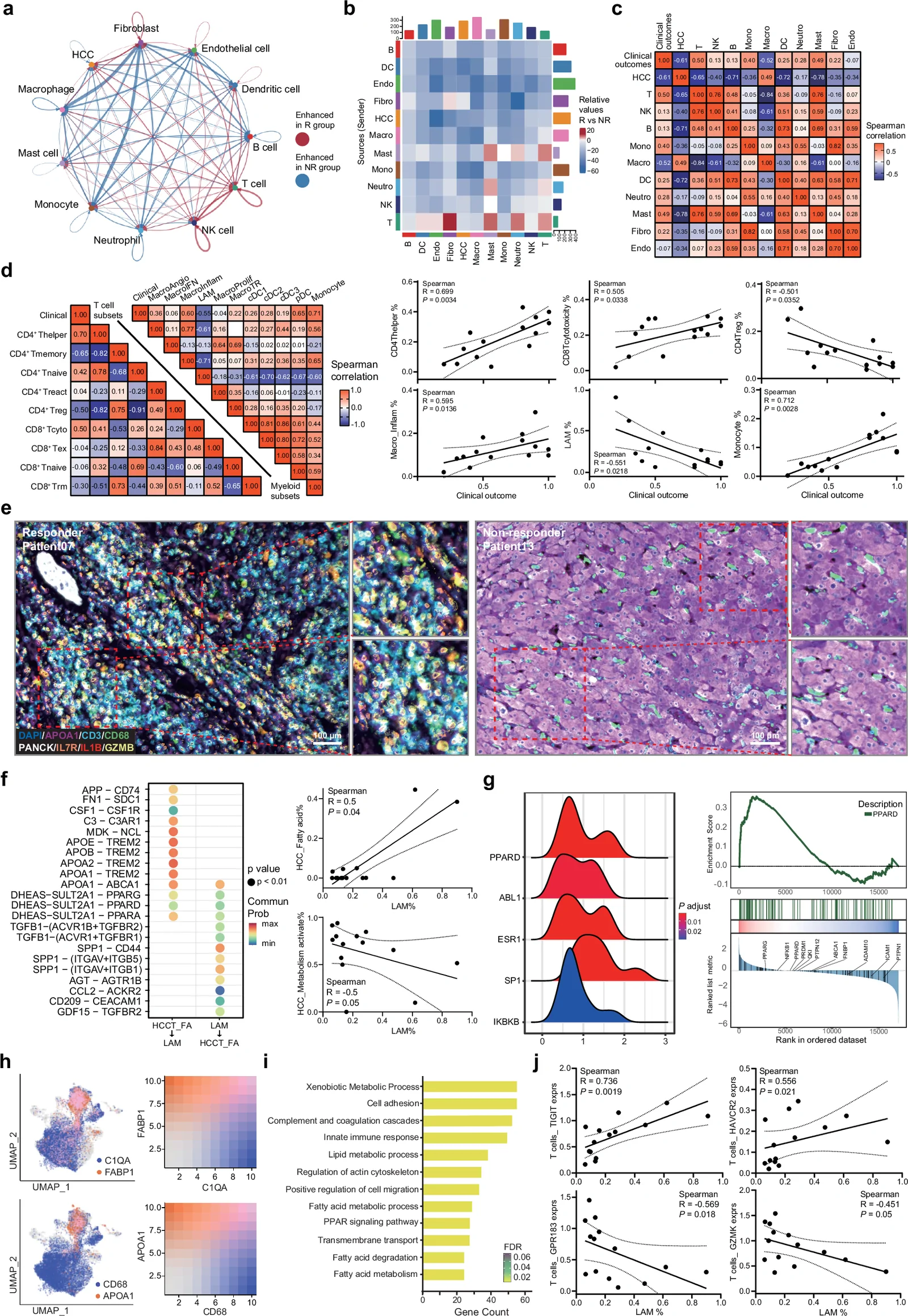
Overall evaluation of the immune-promoting and immunosuppressive subtypes of macrophages and T cells based on clinical outcomes. **a** Circus plot showing distinct interaction strength between responders and non-responders among major cell types. **b** Heatmap of cell-cell interaction between individual major cell types, colored with the relative interaction values of responder versus non-responder. **c** Matrix of Spearman correlation analysis across clinical outcomes and proportions of major cell types from HCC immunotherapy cohort. **d** Matrix of Spearman correlation analysis across clinical outcomes and proportions of T cell subtypes and macrophage subtypes, respectively (*n* = 14). **e** Representative images of mIHC staining from immunotherapy responder (Patient07) and non-responder (Patient13) derived tumor tissue slides. The signals of DAPI, PANCK, CD3, IL7R, GZMB, CD68, IL1B, and APOA1 were merged, indicating the distinct immune infiltration landscape that consistent with analyses of (**d**). Scale bar: 100 µm. **f** Dotplot of ligand-receptor (L-R) interactions between HCC_FA and LAM, dots were colored with the communication strength of L-R. The line chart of Spearman correlation analysis between the proportions of LAM and HCC subtypes indicated the significant positive correlation between LAM and HCC_FA (r = 0.5, *P* = 0.04). **g** Ridge plot showing the density distribution of fold change values of ligand-target genes for the five transcript factors from LAM, including *PPARD*. The following GSEA plot of transcription factor *PPARD* further indicated the genes enriched, such as *PPARG* and *ABCA1*. **h** Featureplot for co-expression analysis of *C1QA*-*FABP1* and *CD68*-*APOA1* illustrated the characteristics of both macrophage and lipid condition in LAM subtype. **i** Bar chart exhibited the GO pathway enrichment of specific biological process based on the expression features of LAM. **j** The line chart of Spearman correlation analysis between the proportions of LAM and expression levels of functional genes in T cells from 14 HCC immunotherapy patients, *TIGIT* and *HAVCR2* represented typical exhausted features, *GPR183* and *GZMK* for effector and cytotoxic condition of T cells (*n* = 14). (Correlation analyses were assessed using one-sided Spearman correlation without multiple comparisons adjustments).

To comprehensively evaluate the TIME components that are associated with clinical efficacy in our HCC immunotherapy cohort, we first utilized the CellChat approach to integrate and compare cell–cell communication networks derived from responder and non-responder patients (Fig. 5a, b). We observed that T cells played a significantly enhanced role in the communication network of responders, whereas macrophages dominated in non-responders. These findings were consistent with the significant weight of T cell and macrophage within the communication networks presented in Fig. 1h. One of the advantages of our 14-patient HCC immunotherapy cohort is the integration of pathological evaluations of resected tumor lesions, we defined the clinical outcomes of patients as “1 - residual tumor rate”, thereby allowing us to quantitatively assess the immunotherapeutic efficacy following αPD-1 treatment. This measure of response enabled a comprehensive correlation analysis between clinical efficacy and the proportions of major cell types, subtypes, and their expression profiles. Accordingly, we first generated a Spearman correlation matrix at the level of major cell types between quantitative clinical immunotherapy outcomes and proportions of cell types among 14 patients of our cohort (Fig. 5c and Supplementary Table 10). Notably, we found that the cell type most significantly negatively correlated with efficacy was the HCC cell population (r = −0.61, *P* = 0.01), thereby validating our use of pathology-based efficacy as an analytical metric. In addition, T cells exhibited a significant positive correlation with clinical efficacy (r = 0.50, *P* = 0.03), whereas macrophages were significantly negatively correlated (r = -0.52, *P* = 0.03). These findings further underscore the pivotal regulatory roles of these two cell types in modulating patient immunotherapy responses.

To further elucidate which specific subtypes within the T cell and macrophage compartments primarily governed immunotherapeutic outcomes, we constructed correlation matrices relating the proportions of T cell subtypes (as identified in Fig. 3a) and myeloid cell subtypes (as detailed in Fig. 4a) to patient clinical efficacy, and combined these results for integrated presentation (Fig. 5d and Supplementary Table 11, 12). Within the T cell subtypes, we confirmed that CD4⁺ Th (r = 0.70, *P* = 0.003) and the CD8⁺ Tcyto (r = 0.51, *P* = 0.034) were significantly positive associated with clinical efficacy, whereas the CD4⁺ Treg was significantly negative correlated (r = -0.50, *P* = 0.035). Among the monocyte–macrophage components, monocytes (r = 0.71, *P* = 0.003) and MacroInflam (r = 0.60, *P* = 0.014) were significantly positive associated with efficacy, while the LAM was significantly negative correlated (r = -0.55, *P* = 0.022). These results further validate our findings from Fig. 3 and Fig. 4 regarding the differentiation and efficacy-associated dynamics of T cells and macrophages. In addition, we performed subtype identification and correlation analyses for other major cell types such as B cells, fibroblasts, and endothelial cells (Supplementary Fig. 5a–f), accordingly identified a fibroblast subtype characterized by high *RGS5* expression that was also significantly negatively associated with clinical efficacy (r = -0.65, *P* = 0.007). Based on the observations, we performed multiplex immunohistochemistry (mIHC) on tissue sections from responders and non-responders. In a representative responder patient07, robust infiltration of GZMB^+^ CD8⁺ Tcyto and IL7R^+^ CD4⁺ Th were observed with minimal residual tumor cells as CPR, whereas in a representative non-responder patient13, a significant presence of residual tumor cells was accompanied by abundant infiltration of LAM exhibiting the *APOA1* signature. These spatial proteomics results further validated our single-cell analysis and the identified regulator roles correlated with clinical outcomes (Fig. 5e and Supplementary Fig. 5g).

To explore potential regulatory mechanisms underlying HCC immunotherapeutic efficacy, we focused on the LAM subtype that significantly negatively correlated with clinical responses, while the incentives of their lipid-associated state have not been fully elucidated. It occurred to us that we have identified an HCC subtype with fatty acid metabolism features (HCC_FA), which was also negatively correlated with clinical efficacy (Fig. 2g) in our previous analyses. By conducting ligand-receptor analysis (Fig. 5f), we discovered the existence of interactions between multiple apolipoproteins and *TREM2* from HCC_FA toward LAM, whereas LAM in turn displayed interactions including *SPP1*–*CD44*. Moreover, the proportion of HCC_FA in patients was significantly positive correlated with the proportion of LAM (r = 0.5, *P* = 0.04), suggesting that the fatty acid metabolic functions of HCC_FA may drive the development of the lipid-associated features in LAM, thereby promoting HCC progression. To demonstrate that LAM and HCC_FA subsets are independent of intrinsic hepatic steatosis in patients and associated with immunotherapy efficacy, we performed correlation analyses between the proportions of the LAM, HCC_FA subsets with liver-to-spleen (L/S) attenuation ratio and BMI (Supplementary Fig. 6a). Neither subset showed a statistically significant correlation with L/S attenuation ratio or BMI. Based on external cellular components that induce LAM lipid-associated states, we further adopted the CellCall approach^36^ to perform transcription factor enrichment analysis on the receptor profiles relevant to LAM (Fig. 5g), leading to the identification of *PPARD* as the key transcription factor, which is a lipid-activated nuclear receptor within the PPAR family. Subsequent GSEA analysis of *PPARD* also revealed its association with another transcription factor, *PPARG,* which pertains to the PPAR family, as well as the metabolism-related gene *ABCA1*. Furthermore, SCENIC analysis^37^ of LAM stratified by clinical response showed that the transcription factor *PPARA* was also significantly enriched in LAM from non-responders (Supplementary Fig. 6b). These comprehensive analyses collectively indicate that lipid-activated transcription factors of the PPAR nuclear receptor family played a crucial regulatory role in LAM, suggesting the metabolic behavior of lipid-accumulated LAM was regulated by the PPAR pathways under the induction of external metabolites. Downstream of PPAR regulation, LAM exhibited high expression of *APOA1*, *APOA2*, and *APOC3*—features regulated by *PPARA*—as well as *FABP1*, which is controlled by both *PPARD* and *PPARG* (Fig. 5h, Supplementary Fig. 6c), indicating that LAM potentially regulates lipid accumulation phenotypes through compensatory metabolic processes. These lipid metabolic features, together with the macrophage markers *CD68* and *C1QA*, formed a distinct metabolically enriched LAM state that regulated by the PPAR signaling pathway and integrated both immune regulation and lipid metabolism functions (Fig. 5i). In the aspect of influences on the immune function of T cells from LAM, we found that the proportion of LAM was significantly negative correlated with typical effector activation markers of T cells (*GZMK* and *GPR183*) and positively correlated with exhaustion markers (such as *TIGIT* and *HAVCR2*) (Fig. 5j). Moreover, the expression levels of *APOA1*, *APOC2* and *FABP1* in LAM were significantly negative correlated with clinical immunotherapeutic efficacy (Supplementary Fig. 6d). Therefore, our comprehensive analyses revealed that this metabolic LAM state is negatively associated with clinical immunotherapeutic efficacy. We further elucidated the external lipid-related incentives, intrinsic transcription factors, downstream formation of expression features, and their impact on the immune process mediated by LAM, thereby illustrating its critical negative regulatory role in the immunotherapeutic response among HCC patients.

### Exploring immunosuppressive function and intervention strategy of LAM

**Fig. 6:**
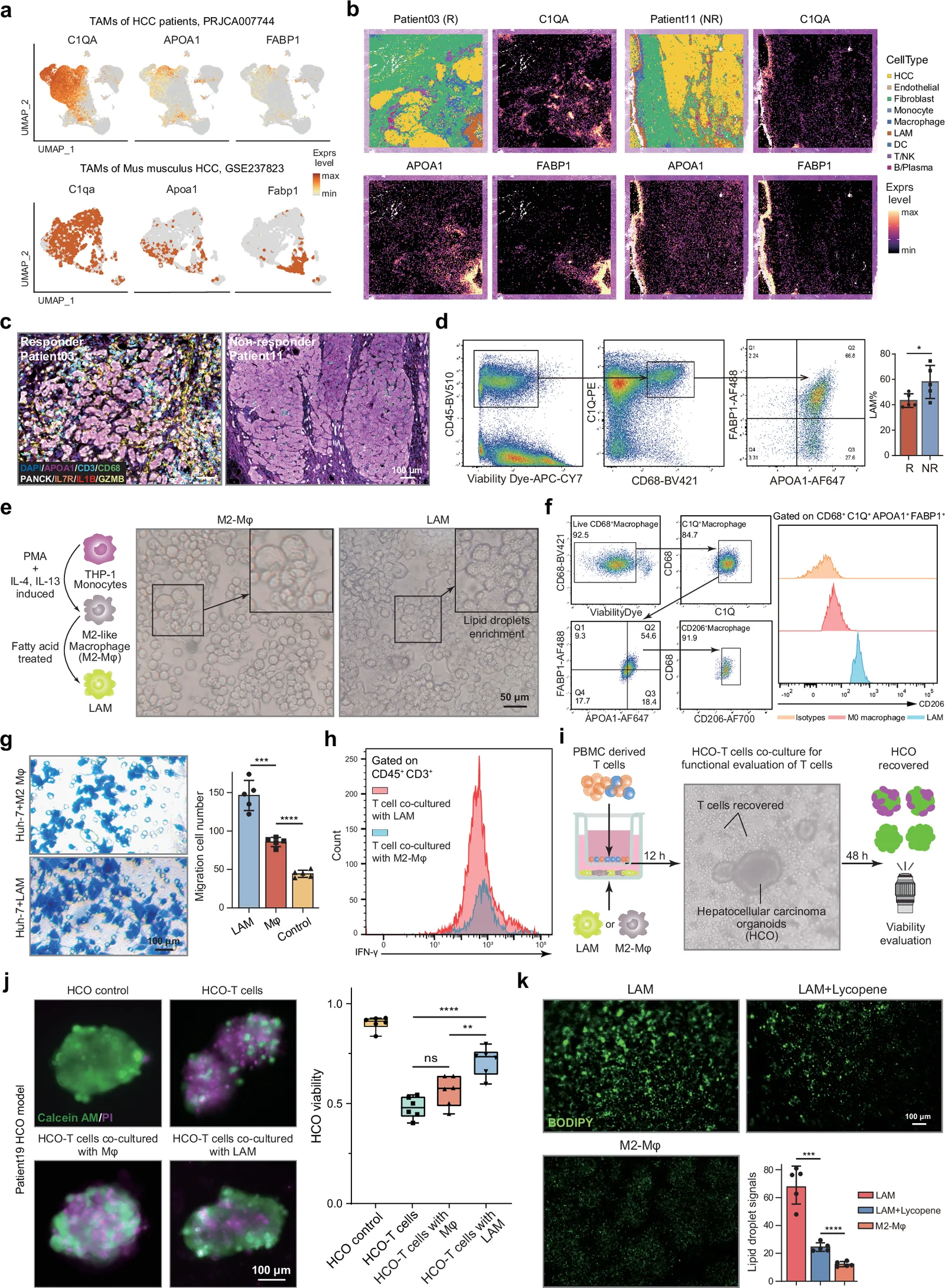
Existence verification and functional demonstration of lipid-associated macrophages in tumor immunosuppressive regulation. **a** External scRNA-seq analysis of HCC patients and mice illustrating the existence of LAM. **b** Spatial transcriptomic datasets that paired with scRNA-seq from our cohort demonstrated the LAM-related features expressed in HCC patients. **c** Representative images of mIHC staining from Patient03 (R) and Patient11 (NR) derived tumor tissue slides. The signals of DAPI, PANCK, CD3, IL7R, GZMB, CD68, IL1B, and APOA1 were merged, indicating the effector T cells in responders and CD68^+^ APOA1^+^ LAM in non-responders. **d** FACS analysis of tumor tissues from HCC patients showing the proportion of LAM in responders and non-responders (*P* = 0.047, *n* = 5). **e** Flow chart showing inducement procedure from THP-1 cell to LAM in two steps, as fatty acid inducement resulted in lipid droplets accumulation in LAM. Scale bar: 50 µm. **f** Corresponding FACS evaluation of induced LAM from (**e**). **g** Transwell assay to validate the impact of LAM and macrophages on Huh-7 cell invasiveness (LAM vs. macrophage, *P* = 1.6 × 10⁻^4^; macrophage vs. control, *P* = 2.0 × 10⁻^6^. *n* = 5). **h** Expression level of IFN-γ assessed by FACS in patient PBMC derived T cells co-cultured with LAM and M2 macrophages. **i** Flow chart illustrating the co-culture assay of T cells with LAM or M2-macrophages, following T cells recovery and co-culture with parental patient-derived hepatocellular carcinoma organoids (HCOs). **j** Fluorescence imaging of live/dead cells in Patient19 HCOs stained by Calcein AM/PI and statistics of six patients HCO models among four conditions (HCO-T-Mφ vs. HCO-T, *P* = 0.054; HCO-T-LAM vs. HCO-T, *P* = 8.1 × 10⁻^5^; HCO-T-LAM vs. HCO-T-Mφ, *P* = 0.006. *n* = 6). k Fluorescence imaging of lipid droplets in macrophages stained with BODIPY (LAM vs. LAM+Lycopene *P* = 1.2 × 10^-4^, LAM+Lycopene vs. M2-Mφ *P* = 5.9 × 10^-5^. *n* = 5). (Unless otherwise specified, for box plots, the center line indicates the median, box limits indicate 25 and 75th percentiles, whiskers indicate minimum and maximum values. Statistic significance was determined by two-sided unpaired student’s *t* tests. *P < 0.05, **P < 0.01, ***P < 0.001, ****P < 0.0001. Scale bar: 100 µm. Images in Fig. 6e, i are provided by Servier Medical Art (https://smart.servier.com), licensed under CC BY 4.0 (https://creativecommons.org/licenses/by/4.0/)).

Although our 14-patient HCC immunotherapy cohort has provided certain evidence for the existence, formation, and immunosuppressive function of LAM subtype, we further sought to validate its broader presence and negative impact on immunotherapeutic efficacy. First, we analyzed single-cell transcriptomic^12^ data from HCC patients and models of Mus musculus^38^, further identified a similar macrophage subtype that simultaneously exhibited characteristic myeloid markers and lipid metabolism features (Fig. 6a). Moreover, we conducted spatial transcriptome sequencing using paired patient samples from our HCC scRNA-seq cohort (Fig. 6b), along with external spatial transcriptomic data^33^ (Supplementary Fig. 6e), collectively demonstrating the presence of LAM and its particular accumulation in non-responders. Meanwhile, integrated analysis of our data with external scRNA-seq data^33^ showed distinctions between metabolically enriched LAM and SPP1^+^ TAMs (Supplementary Fig. 6f, g). Additionally, mIHC staining for CD68 and APOA1 in patient tumor sections revealed the infiltration of LAM subset, which was particularly prominent in non-responders (Fig. 6c and Supplementary Fig. 6h). Furthermore, we collected clinical HCC patient samples and performed flow cytometry with C1Q, APOA1 and FABP1 markers related to LAM to confirm the existence of this specific lipid-associated subtype condition within TIME of HCC patients (Fig. 6d). In summary, through the integration of external single-cell datasets and multi-omic analyses on clinical HCC samples, we have demonstrated the objective existence of the LAM subtype in tumor lesions derived from HCC patients.

Based on these findings, we then aimed to establish an in vitro model of macrophages exhibiting the LAM features for the functional exploration. We designed a lipid phenotype induction strategy using the human monocyte cell line THP-1. Briefly, THP-1 cells were first differentiated into M2-type macrophages with PMA and IL-4, combined with IL-13, successively. Then macrophages were stimulated with oleic acid—a typical unsaturated fatty acid—as an exogenous cue to up-regulate lipid metabolism and induce a LAM state characterized by visible lipid droplet accumulation (Fig. 6e and Supplementary Fig. 6i). Flow cytometry analysis of THP-1–derived macrophages before and after oleic acid inducement revealed up-regulated CD206, APOA1 and FABP1 features in C1Q^+^ CD68^+^ macrophages, demonstrating that in vitro induction strategy recapitulated the condition of LAM state (Fig. 6f). Based on the in vitro establishment of LAM, we further explored its regulatory effects on HCC and T cells. Through transwell assays, we discovered that compared to M2-macrophages, co-culture of LAM with the Huh-7 liver cancer cell line resulted in a significant enhancement of cell migration and invasion (Fig. 6g). This result of observation is consistent with the interactions between LAM and HCC cells identified in the patient single-cell transcriptomic data (Fig. 5f). Moreover, we constructed co-culture system consists of LAM and T cells derived from peripheral blood of HCC patients within transwell for 12 hours, the T cells exhibited significantly reduced IFN-γ production compared to T cells co-cultured with M2 macrophages, indicating that the LAM exerts stronger immunosuppressive effect on T cell immune functions (Fig. 6h).

To further investigate the impact of LAM on T cell–mediated tumor killing, we successively enrolled untreated HCC patients and generated six individuals of patient-derived hepatocellular carcinoma organoids (HCO) using our previously established “Dual grinding and filtering” approach^39,40^ optimized for pan-cancer tissues. Peripheral blood mononuclear cell (PBMC) derived T cells that homologous to HCOs derived patients had been co-cultured with LAM in transwell first, and then re-introduced to parental HCOs for an additional 48-hour co-culture, following with live/dead HCOs viability staining for assessment of T cell function (Fig. 6i). Despite the limited antigen-specific frequency and cytotoxicity of PBMC derived T cells, the results demonstrated that T cells pre-exposed to LAM exhibited significantly diminished tumor-killing capabilities, further suggesting the negative regulatory effect of the lipid-accumulated LAMs on T cell immune function and subsequently compromised immunotherapy efficacy in potential (Fig. 6j). These findings are consistent with the observed negative correlation between the lipid characteristics of LAM and T cell activation features, as well as the positive correlation with T cell exhausted conditions identified in our HCC immunotherapy cohort (Supplementary Fig. 6j). Additionally, in efforts to modulate the lipid state of LAM subset, we treated established LAM with lycopene, which is a compound known to correct lipid metabolic abnormalities. Lycopene treatment resulted in a significant reduction of lipid droplet accumulation in LAM (Fig. 6k) and attenuated their lipid-associated characteristics (Supplementary Fig. 6k), suggesting a potential adjunct therapeutic strategy for targeting the lipid-accumulation phenotype of LAMs in HCC patients to improve the outcomes of αPD-1 immunotherapy.

### Exogenous lipid intake induce LAM with impaired αPD-1 efficacy while restored through in vivo lipid metabolism regulation

**Fig. 7:**
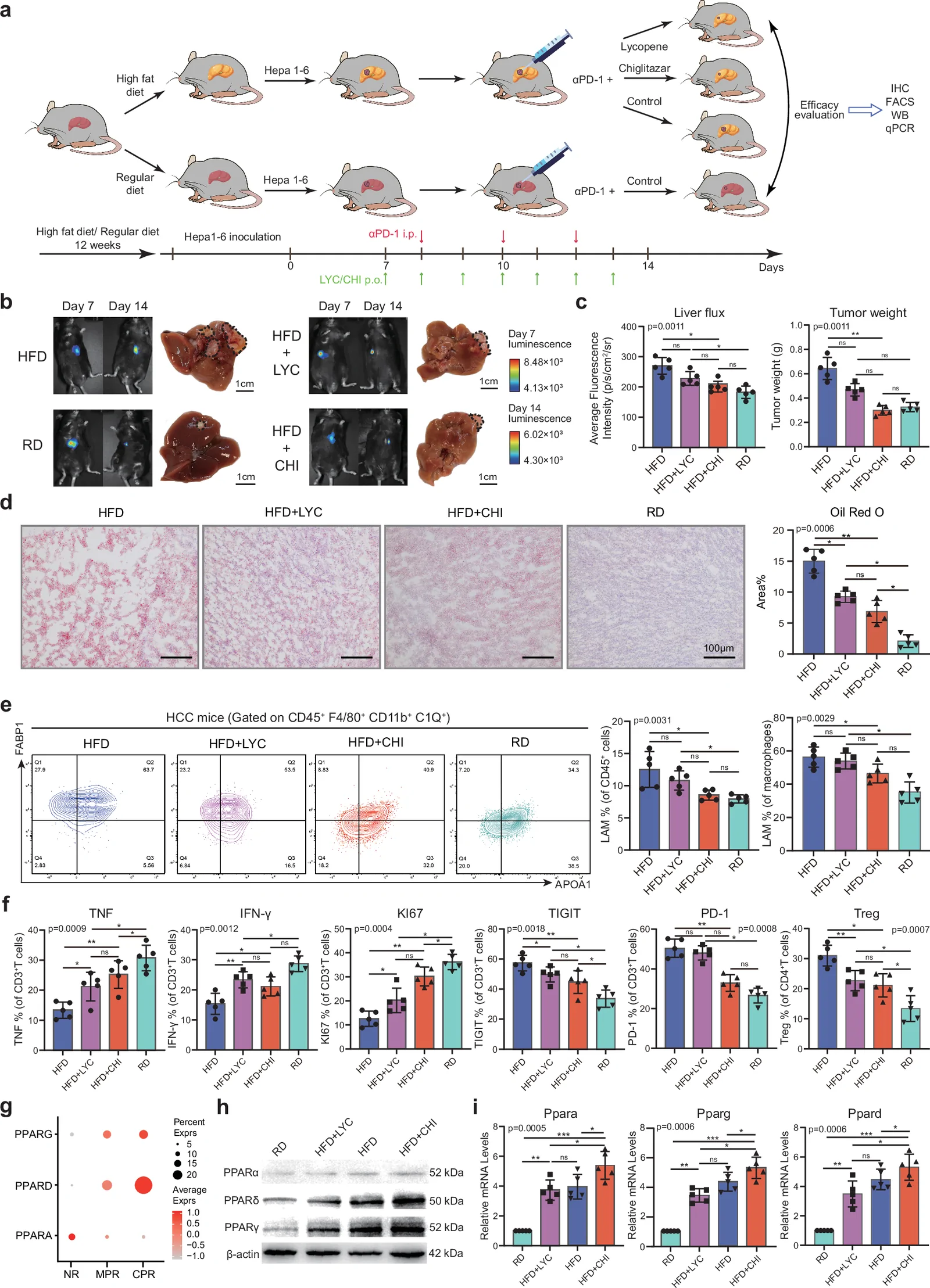
High-fat diet facilitates LAM-mediated immunosuppression in HCC and further regulated by lycopene and chiglitazar. **a** The HCC mice received αPD-1 immunotherapy were randomly assigned to four experimental groups: mice with regular diet (RD), mice with high fat diet (HFD), mice with HFD and lycopene (LYC), mice with HFD and chiglitazar (CHI) (*n* = 5). Subsequently, immunohistochemistry, flow cytometry, WB and qPCR were conducted to explore the features and mechanisms of LAM immunosuppressive function. **b** Representative in vivo bioluminescence images and tumor image (*n* = 5). **c** Average fluorescence intensity in day 14 (*n* = 5) and tumor weight of HCC mice at endpoint (*n* = 5). **d** Representative Oil Red O staining of tumor regions showing lipid accumulation degree of each group (*n *= 5). **e** The proportions of LAM in CD45⁺ cells and CD45⁺ F4/80⁺ CD11b⁺ C1Q⁺ macrophages analyzed by FACS (*n* = 5). **f** FACS analysis of intratumoral T cell function, The frequencies of effector cytokine production (TNF and IFN-γ), proliferative capacity (Ki-67) and exhausted (PD-1 and TIGIT) cells were assessed within total CD3⁺ T cells. Regulatory T cells were identified as CD4⁺ FOXP3⁺ cells and quantified as a percentage of CD4⁺ T cells (*n* = 5). **g** Bubble plot showing the expression level of PPAR-related genes in LAMs of patients with NR, MPR and CPR responses from our HCC immunotherapy cohort. **h** The protein expression levels of PPARα, PPARδ and PPARγ from mouse HCC tissue were measured by western blotting. The experiment was independently repeated three times with similar results. **i** The relative expression levels of Ppara, Ppard and Pparg genes were measured by qPCR in mouse HCC tissue (*n* = 5). (Data in Fig. 7c, d, e, f, i are presented as mean values ± s.e.m. For multi-group comparisons, statistical differences were assessed using the Kruskal–Wallis test with *P* value labeled as numerics, the statistical differences between every two groups were then assessed by Dunn’s multiple-comparison test with multiplicity adjusted for each comparison. *P < 0.05, **P < 0.01, ***P < 0.001, ****P < 0.0001, ns: not statistically significant).

To determine whether LAM contributes to impaired immunotherapy efficacy and whether modulation of LAM-associated metabolic programs could restore antitumor immunity, we established an orthotopic mouse HCC model combined with dietary and pharmacological interventions. In this behalf, we aimed to determine whether lycopene (LYC) and the PPAR agonist chiglitazar (CHI) could alleviate lipid metabolic dysregulation in LAMs, thereby restoring antitumor immunity and enhancing the efficacy of PD-1 blockade. Specifically, C57BL/6 J mice (Male) were fed either a regular diet (RD) or a high-fat diet (HFD) for 12 weeks. Mice fed with HFD were then randomly divided into three groups, including HFD, HFD supplemented with lycopene (HFD + LYC) or HFD supplemented with chiglitazar (HFD + CHI). Following dietary conditioning, orthotopic liver tumors were established and mice were treated with PD-1 antibody intraperitoneally at seventh day after tumor inoculation according to a standardized schedule (Fig. 7a). We found that mice maintained on a regular diet exhibited pronounced tumor regression following αPD-1 therapy. In contrast, tumors in the HFD group did not decrease, indicating resistance to immunotherapy. Notably, both metabolic intervention groups (HFD + LYC, HFD + CHI) displayed intermediate tumor control, with significantly reduced tumor burden compared to the HFD group but less pronounced regression than RD group (Fig. 7b, c). In Oil Red O staining, the HFD group demonstrated pronounced lipid deposition within the tumor microenvironment. In contrast, lipid accumulation was substantially reduced in the lycopene- and chiglitazar-treated groups, while the RD group exhibited minimal lipid staining (Fig. 7d).

Next, we assessed the impact of these interventions on HCC TIME using flow cytometry. The results revealed a marked expansion of LAM in tumors from HFD mice, whereas RD mice exhibited the lowest LAM abundance. Importantly, both lycopene and chiglitazar significantly reduced the accumulation of LAMs compared to the HFD group (Fig. 7e and Supplementary Fig. 7a). Corresponding to these macrophage changes, the HFD group displayed the most pronounced impairment of T cell function (Fig. 7f and Supplementary Fig. 7b, c), characterized by reduced effector cytokine production (TNF and IFN-γ), diminished proliferative capacity (Ki-67), increased expression of exhaustion markers (PD-1 and TIGIT) within CD3^+^ T cells, and an elevated proportion of regulatory T cells within CD4^+^ T cells (Supplementary Fig. 7d–g). In contrast, mice of the RD group showed preserved T cell effector function and minimal exhaustion, while the lycopene and chiglitazar-treated groups exhibited intermediate immune phenotypes.

Given the strong association between lipid accumulation and treatment resistance, we next investigated the metabolic characteristics of LAMs within the mice tumors. Based on patient scRNA-seq data, LAM from non-responders exhibited attenuated PPAR signaling compared with responders (Fig. 7g). Given the central role of PPAR pathways in regulating fatty acid oxidation and lipid homeostasis, we hypothesized that impaired PPAR activity may constrain lipid-handling capacity in LAM, thereby sustaining a lipid-accumulation and immunosuppressive phenotype. Western blot and quantitative PCR assays were performed in the mouse model to confirm activation of the PPAR pathway (Fig. 7h, i). In chiglitazar-treated tumors, enhanced PPAR signaling systemically promoted lipid oxidation and alleviated lipid accumulation within TIME of HCC, thereby reducing the proportion and mitigating the immunosuppressive phenotype of LAM. In contrast, lycopene treatment reduced lipid accumulation despite only modest activation of the PPAR pathway, indicating that lycopene likely acts through PPAR-independent mechanisms to modulate macrophage lipid metabolism. Together, these data suggest that responder-associated LAM maintains a higher lipid metabolic flux, enabling efficient lipid clearance and limiting lipid accumulation, whereas non-responder-associated LAMs remain metabolically constrained, reinforcing immunosuppressive and pro-tumorigenic functions.

Collectively, these in vivo findings indicated the immunosuppressive effects caused by LAMs, which resulted in αPD-1 immunotherapy resistance in HCC. Accordingly, the metabolic reprogramming of LAM through modulation of PPAR signaling and reduction of lipid accumulation led to the restored antitumor immunity and sensitivity to PD-1 blockade.

### Interaction networks among responders and non-responders indicate key regulators and intervention targets for immunotherapy

**Fig. 8:**
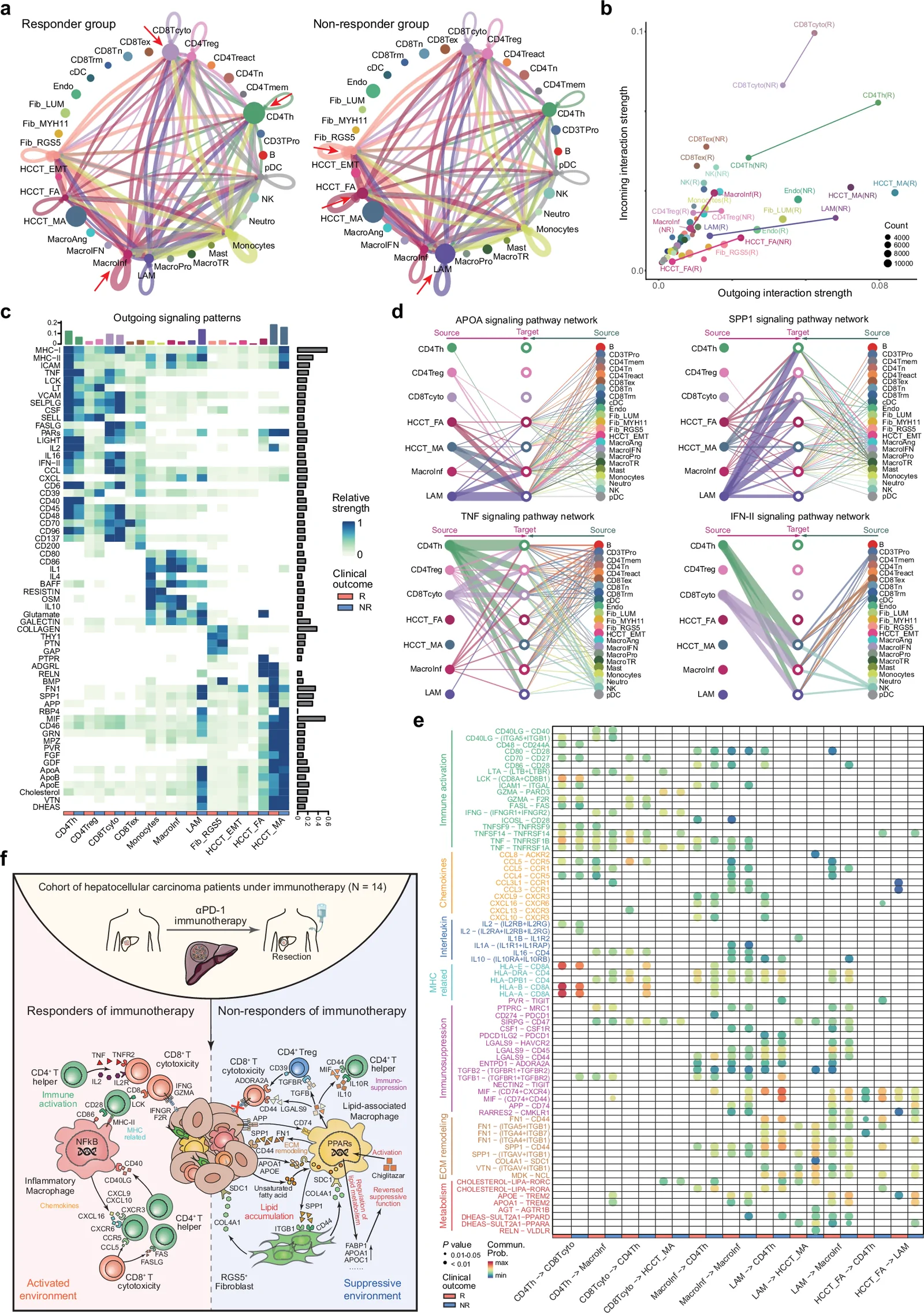
Immune interaction landscape of integrated responder and non-responder communication networks within TIME, revealing the functions of critical immunotherapy regulators. **a** Circus plot illustrating the intercellular communications strength among responders and non-responders of HCC immunotherapy cohort. The red arrows pointed the up-regulated interaction strength of MacroInflam, CD4⁺ Th and CD8⁺ Tcyto cells in the responder group, as well as the LAM, HCCT_FA and Fib_RGS5 were up-regulated in non-responders. **b** Scatter plot illustrated the transitions of incoming and outgoing interaction strength across TIME components between responder and non-responder groups. **c** Outgoing signaling patterns of critical TIME components in the responder group and the non-responder group, colors represented the values of relative signal strength. **d** Hierarchical plots showing the signaling pathways of APOA, SPP1, TNF and IFN-II within interaction network of TIME. **e** Bubble plot illustrated the ligand-receptor interaction landscape among critical TIME components, including CD4⁺ Th, CD8⁺ Tcyto, MacroInflam, LAM and HCC_FA of responder and non-responder groups. The ligand-receptor interactions were divided into six categories, including immune activation, chemokines, interleukin, MHC-related, immunosuppression, ECM remodeling and metabolism-related. **f** Figure of immune interaction landscape of integrated responder and non-responder communication networks within TIME, indicating the critical ligand-receptor interactions and intervention targets of HCC immunotherapy. Images provided by Servier Medical Art (https://smart.servier.com), licensed under CC BY 4.0 (https://creativecommons.org/licenses/by/4.0/).

Based on single-cell data from our HCC immunotherapy cohort, we have identified critical regulators of TIME that modulate clinical immunotherapeutic efficacy, with particular focus on the metabolically enriched LAM state as a negative regulator of immunotherapy response. Based on these findings, we further integrated the cell-cell communication networks within the tumor immune microenvironment of responders and non-responders to comprehensively elucidate the modes of immune functions and intervention targets for these pivotal immune regulatory components. Therefore, we constructed the interaction networks for responder and non-responder patients of our HCC immunotherapy cohort, respectively (Fig. 8a). Notably, in the network from responders, the relative contribution of CD4⁺ Th, CD8⁺ Tcyto and MacroInflam subtypes was significantly increased, whereas the contribution of HCC_FA, CD4⁺ Treg and especially LAM was up-regulated in non-responders. These differences were reflected in the distinct patterns of interaction signal sending and receiving across the R and NR groups (Fig. 8b and Supplementary Fig. 8a).

We then performed a detailed analysis of the specific signaling pathways mediated by these key immune regulatory components (Fig. 8c and Supplementary Fig. 8b). Our results revealed that CD4⁺ Th and CD8⁺ Tcyto predominantly facilitated immune activation via pathways involving *CD137*, *CD40*, *TNF*, and *IFN-II*, with these pathways being up-regulated in responders. Meanwhile, the MacroInflam subtype participated more significantly in pathways related to M1-like macrophage functions (e.g., via *CD80* and *CD86*) in responders, whereas the LAM subtype was primarily active within the network of non-responders. Specifically, LAM contributed to lipid-related pathways (involving APOA, APOB, APOE and cholesterol), as well as to extracellular matrix (ECM) remodeling (*SPP1*/*FN1*) and immune-suppressive (*MIF*) pathways, suggesting the mechanisms related to lipid phenotype formation and corresponding immunosuppressive function of LAM. To further delineate how these key signaling pathways are relayed among various cell subtypes, we constructed hierarchical pathway diagrams (Fig. 8d, Supplementary Fig. 8c). These diagrams revealed that CD4⁺ Th mainly exert immune-promoting effect via *CD40*/*TNF*/*LIGHT* pathways towards the rest of T cells, CD8⁺ Tcyto chiefly activate various T cell subsets through *CD70* signaling and kill tumor cells via *IFN-II* pathway. Within the macrophage components, the M1-like MacroInflam subtype contributes to T cell activation through pathways such as *CD80* and *ICOS*, while the LAM subtype is primarily influenced by lipid metabolism signals from HCC cells through *APOA*/*APOB*/*APOE* and affects other cell types via lipid and cholesterol metabolism pathways. Moreover, LAM also impacts multiple immune cell types, tumor cells, and endothelial cells via ECM remodeling through *FN1* and *SPP1*, thereby inhibiting immune cell infiltration through the fibrotic program. Their involvement in MIF signaling further exerts a significant suppressive effect on various T cell populations.

Based on the analyses above, we further compiled the ligand–receptor interaction networks of these key immunoregulatory roles across responders and non-responders (Fig. 8e and Supplementary Fig. 8d, e). We divided specific ligand–receptor interactions into seven groups of biological functions, including immune activation, chemokines, interleukins, MHC-related, immunosuppression, extracellular matrix remodeling and metabolic regulation. This integrative interaction map, which mainly includes CD4⁺ Th, CD8⁺ Tcyto, CD4⁺ Treg, RGS5^+^ Fibroblast, LAM, MacroInflam, and HCC cells, highlights the distinct intercellular communication patterns underlying different therapeutic responses (Fig. 8f). In immunotherapy responders, the LAM subtype is nearly absent and therefore exerts little influence, while the presence of MacroInflam subtype predominates, recruiting CD4⁺ Th via chemokines such as *CXCL9*-*CXCR3* and *CXCL16*-*CXCR6*, and subsequently activating T cells through interactions such as *CD40*–*CD40LG* and *CD86*–*CD28*. Meanwhile, the CD4⁺ Th activate CD8⁺ Tcyto via *IL2*–*IL2R* and *TNF*–*TNFRSF1B*, thus promoting the tumor killing effect mediated by CD8⁺ Tcyto through *IFNG*–*IFNGR* and *GZMA*–*PARD3*. In stark contrast, non-responder patients exhibited a higher prevalence of the LAM subtype, which predominantly drives immunosuppression as previously detailed. The lipid-associated condition of LAM was induced by lipid metabolic signals from HCC cells (e.g., via *APOA1*–*TREM2* and *APOE*–*TREM2*), and the LAM further influenced T cells and other immune cells through lipid and cholesterol-related pathways in return. Besides the lipid metabolism influence on the tumor immune microenvironment, LAM exerted immunosuppressive functions through two approaches. On the one hand, LAM promoted ECM fibrosis through *SPP1* and *FN1* (Supplementary Fig. 8f, g), thereby reducing immune cell infiltration and diminishing the antitumor activity of T-cells. On the other hand, LAM driven immune escape and T cell exhaustion thus established an immunosuppressive state via *MIF*–*CD44*, *LGALS9*–*CD44, TGFB-TGFBR* and *IL10-IL10R* interactions, ultimately contributing to poor prognosis in non-responders of HCC immunotherapy. In summary, by integrating the communication networks of key immune regulatory components in responders and non-responders, we have systematically delineated the driving factors and potential regulatory targets underlying heterogeneous responses in HCC immunotherapy. In particular, we propose that the LAM subtype exerts multiple immunosuppressive functions in non-responder patients and should be considered as a critical target for metabolic interventions, thus improving immunotherapeutic outcomes in HCC.

## Discussion

The immunotherapy of HCC is evolving at a rapid pace, multiple strategies such as combinations of ICIs with chemotherapy (HAIC/TACE)^41,42^, with targeted drugs (αVEGF/TKIs)^4,5^, or a combination of dual ICIs (αPD-1 and αCTLA-4)^6,7^ are bringing hope for higher objective response rates and prolonged survival in HCC patients. Although most immune checkpoint inhibitors primarily target T cells (e.g., PD-1, CTLA-4, TIGIT) or tumor cells (e.g., PD-L1), the current limited objective response rate is mainly attributable to intrinsic differences in the tumor immune microenvironment among HCC patients. Therefore, a comprehensive dissection of the tumor immune microenvironment by leveraging technologies such as single-cell sequencing, spatial transcriptomics, and spatial proteomics towards HCC patients undergoing immunotherapy is vital. Such analyses not only enhance our understanding of the immune components and underlying mechanisms that influence clinical efficacy but also pave the way for predicting immunotherapeutic responses from biopsy specimens, optimizing treatment strategies, and identifying potential therapeutic targets to extend treatment options and improve clinical outcomes.

In this study, to elucidate the interpatient heterogeneity in immunotherapeutic responses, we established a cohort of 14 HCC patients undergoing αPD-1 immunotherapy and performed scRNA-seq on resected tumor lesions. By integrating these data with public HCC single-cell datasets, we generated a high-throughput single-cell atlas. Accurate identification of major cell types and their subtypes, combined with quantitative pathology-based assessment of clinical outcomes, allowed us to delineate critical TIME components and their correlation with immunotherapy. Recent neoadjuvant αPD-1 translational studies in HCC have substantially advanced our understanding of immune determinants on therapeutic responses (Supplementary Table 13). Prior reports have largely emphasized T cell–centric mechanisms, including instructional niches coordinating dendritic cells and CD4⁺ T helper subsets^14^, clonal expansion dynamics of dominant CD8⁺ T cell populations^16^ and chemokine-driven peripheral-to-tumor trafficking as key drivers of response^43^. Other studies have described spatial immune exclusion patterns at the tumor boundary, highlighting immune barrier architectures associated with resistance^33^. While these findings collectively underscore the mechanism of T cell and physical barrier consists of macrophage and fibroblast, the myeloid compartment has remained comparatively less mechanistically dissected. Notably, we identified a metabolically enriched LAM state characterized by *C1QA*, *FABP1* and *APOA1* expression. This LAM subset with lipid accumulation phenotype was potentially induced by HCC-derived external metabolites and related to immunosuppressive effects, thus exhibiting significant negative correlation with clinical immunotherapy outcomes. Through the paired spatial transcriptome sequencing, multiplex immunohistochemistry and flow cytometry, we further confirmed the widespread presence of LAM within the TIME among HCC patients in our cohort and external HCC public datasets as well. Furthermore, by conducting experiments based on a patient-derived organoid in vitro model and an in vivo mouse model, we demonstrated that exogenous lipid intake induced the formation of LAM, leading to immunosuppression and impaired αPD-1 efficacy. Moreover, we discovered that the regulation of lipid metabolism with chiglitazar or lycopene could optimize the circumstances above and restore anti-tumor immunity in vivo. Besides, we constructed comprehensive communication networks of the tumor immune microenvironment across patients from responder and non-responder groups. These integrative network analyses unveiled a landscape of ligand–receptor interactions among critical immunotherapy regulators, including CD4⁺ Th, CD8⁺ Tcyto, MacroInflam and especially LAM that are closely associated with clinical outcomes. Therefore, we systematically mapped regulators of heterogeneous immunotherapeutic responses and identified potential targets for precise intervention.

Nevertheless, there are still certain limitations and deficiencies in our research at the current stage. Firstly, paired sampling of patients before and after neoadjuvant immunotherapy was not conducted, contrary to regional clinical guidelines, as paired results tend to improve the dynamic evaluation of HCC immunotherapy. In addition, no significant differences in RFS or OS were observed between response groups in our cohort, indicating the limited sample size and available follow-up period may restrict the ability to detect survival differences, while our study mainly aims to characterize the immune-metabolic features associated with pathological responses to neoadjuvant immunotherapy in TIME, rather than to establish a direct link between LAMs and clinical outcomes. Secondly, the LAM percentage of individual patients is not directly related to fatty liver, leaving the relationship between LAM formation and the state of fatty liver disease as a worthy issue to explore in the future^44^. Then, the comprehensive immune functions and processes exerted by T cells within patient-derived in vitro models were not fully studied. Antigen specificity of tumor-reactive T cells was not directly demonstrated, as the combination of scRNA-seq and scTCR-seq with co-culture models could be a suitable strategy to explore this issue. Finally, the high-fat diet model modulates systemic lipid metabolism and does not exclusively target macrophage-intrinsic pathways. Thus, although the in vivo data support a lipid-driven immunosuppressive axis, they do not fully isolate cell-autonomous mechanisms of LAMs. Moreover, while pharmacologic PPAR modulation functionally implicates lipid metabolic programs, direct genetic perturbation of specific LAM-associated molecules, such as *SPP1* or *TREM2* was not performed and remains an important direction for future study. We believe that the perspectives above should be further explored in the future work.

In summary, our study combined a valuable αPD-1 HCC immunotherapy cohort with single-cell transcriptomic profiling and multi-omics analyses to reveal critical immunomodulatory roles and mechanisms, particularly the immunosuppressive role of a metabolically enriched LAM state and corresponding intervention strategies. Although our discoveries were robustly correlated with clinical immunotherapeutic efficacy, further validation through rigorous cellular and animal experiments, as well as analyses involving larger clinical sample sets, is warranted. Nonetheless, the insights gained from our integrated, system-level analyses offer valuable perspectives on the mechanistic basis of HCC immunotherapy, strategies to enhance therapeutic efficacy, and the development of new targets that could ultimately benefit a broader patient population.

## Methods

### Study design and sample selection

The study protocol adhered to the principles of the Declaration of Helsinki. Written consent was obtained for all tissue samples with ethical approval from the Institutional Review Board and Ethics Committee of Qilu Hospital of Shandong University. The study included patients who received αPD-1-based systemic therapy prior to radical surgery and had complete clinical information. Sample selection criteria were as follows: 1. Patients with pathologically confirmed HCC; 2. The treatment administration was PD-1 inhibitor-based systemic therapy, with no prior local interventional therapy; 3. Pathological evaluation by two independent experts confirmed that the eligible patient samples contained sufficient residual cells for analysis, while the unqualified patient samples were largely necrotic, making it difficult to detect sufficient active cellular components. The treatment regimen was determined according to the institutional clinical protocol approved by regulatory authority and based on contemporary clinical practice guidelines for HCC^45^. Moreover, this study was conducted within the framework of our prospectively registered perioperative clinical research program (ClinicalTrials.gov identifier: NCT06571396). Sex was assigned (22 male and 8 female patients). Sex-based analyses were not performed due to the limited sample size. Gender was not determined.

### Inclusion and exclusion criteria of cohort construction

Participants must meet all of the following criteria to be eligible for enrollment:

1. Age ≥ 18 years.

2. Histologically, cytologically, or radiologically diagnosed HCC.

3. Child–Pugh liver function class A.

4. Eastern Cooperative Oncology Group (ECOG) performance status score of 0–1.

5. Estimated life expectancy ≥ 12 months.

6. Patients who received at least one cycle of PD-1 antibody–based immunotherapy prior to curative-intent surgical resection.

Participants meeting any of the following criteria were excluded from the study:

1. Known diagnosis of intrahepatic cholangiocarcinoma, sarcomatoid HCC, mixed hepatocellular–cholangiocarcinoma, or fibrolamellar carcinoma.

2. Current interstitial pneumonitis or interstitial lung disease, or a prior history of interstitial lung disease requiring systemic corticosteroid treatment.

3. Use of immunosuppressive agents or systemic corticosteroids within 2 weeks prior to enrollment for immunosuppressive purposes.

4. Presence of active infection; unexplained fever ≥38.5 °C within 1 week prior to randomization; baseline leukocyte count >15 × 10⁹/L; or receipt of therapeutic oral or intravenous antibiotics within 2 weeks prior to randomization (excluding prophylactic intravenous antibiotics administered for ≤48 h).

5. Congenital or acquired immunodeficiency, including known HIV infection.

6. Clinically significant bleeding events or clear bleeding tendency within 6 months prior to treatment initiation, such as gastrointestinal bleeding, severe esophagogastric varices, hemorrhagic gastric ulcer, or vasculitis. If fecal occult blood test was positive at baseline, require repeat test; persistent positivity mandated gastroscopic evaluation.

7. Known hereditary or acquired bleeding disorders (e.g., coagulation dysfunction) or thrombotic predisposition, high surgical risk, or inability for curative-intent surgery.

8. Major vascular diseases occurring within 6 months prior to randomization (e.g., aortic aneurysm requiring surgical repair or recent peripheral arterial thrombosis) or other conditions directly increasing surgical risk and precluding surgery.

9. Receipt of major surgery (excluding diagnostic procedures) within 4 weeks prior to study enrollment, or anticipated need for non-oncologic major surgery during the study period.

10. Known hypersensitivity or allergy to any investigational drug or its excipients.

11. Participation in another interventional drug clinical trial.

12. Pregnant or breastfeeding women.

13. Any other condition deemed by the investigator to make the patient unsuitable for participation in the study.

### Single cell sample collection and suspension preparation

Fresh tumor samples were obtained during surgical resection and rapidly transported to the research facility on ice. Tissues were transferred into a sterile culture dish containing 10 mL of 1× Dulbecco’s phosphate-buffered saline to remove residual storage solution and then minced into 1–3 mm³ fragments. Tissue digestion was performed using type I collagenase dissolved in 10 mL RPMI 1640 medium supplemented with 10% fetal bovine serum. Samples were incubated at 37 °C with gentle agitation (50 r.p.m.) for 20 min to achieve adequate dissociation.

The resulting cell suspensions were passed through a 70-µm nylon cell strainer, and red blood cells were removed using 1× Red Blood Cell Lysis Solution. Cells were washed with DPBS containing 2% FBS. Cell viability was assessed using 0.4% Trypan Blue staining on a Countess II Automated Cell Counter, and only samples with viability >85% were processed further.

Cells meeting the quality criteria were washed, resuspended to an appropriate concentration of 700–1200 cells/μl, and prepared for operation on 10x Genomics Chromium system. Specifically, cells were loaded according to the expected number of target cells to construct Gel Bead-in-Emulsion (GEM) droplets for single-cell isolation. Once GEMs were successfully formed, they were collected and subjected to reverse transcription in a PCR machine for tagging. After reverse transcription, GEMs were processed to break the oil emulsion, and single-strand cDNA was purified and enriched using magnetic beads. The cDNA was then amplified in a PCR machine and quality-checked. Quality-checked cDNA was used to construct sequencing libraries through fragmentation, adapter ligation, and sample index PCR. The libraries were subsequently quantified and subjected to quality control. Finally, the constructed libraries were sequenced using the Illumina HiSeq platforms, employing a PE150 sequencing mode to achieve a read depth of 50,000 read pairs per cell or greater.

### Raw sequencing data processing and quality control

Raw sequencing data was subjected to quality control analysis using FastQC software. The 10x Chromium single-cell transcriptome was analyzed through 5’ end transcript sequencing, alongside sequencing analysis of T cell and B cell receptor gene TCR/BCR sequences. From the Fastq Reads, a cell gene expression matrix was generated, with one of the key steps being the identification of valid cells from background signals. Cell Ranger software was employed to perform quality control statistics on the raw Fastq data, align it to the Ensembl reference genome, perform cell demultiplexing, remove PCR duplicates, and identify cells. Standard quality control filters were applied to remove low-quality cells based on gene counts, UMI counts, and mitochondrial gene percentages. We examined the overall distribution of gene counts in each cell and filtered out cells with gene counts of <200 or > 6000. To remove damaged cells indicated by the leakage of mitochondrial genes, we evaluated sample-wise percentage of mitochondrial reads sequenced per cell to eliminate potential debris and damaged cells that could affect the overall quality of the data. Cells with over 30% mitochondrial reads were removed from the dataset. Across samples with quality control (Supplementary Table 14), a median of 8932.5 cells were captured per library. Libraries were sequenced to a median depth of 71049.5 reads per cell, yielding a median of 1577 genes and 1692.5 unique molecular identifiers (UMIs) per cell, with library saturation ranging from 90% to 95%. To further address technical artifacts, DoubletFinder was used to identify potential doublets and the cells in top 1% pANN score were removed. For each sample, the optimal principal component neighborhood size (pK) was selected based on the highest bimodality coefficient using the mean–variance–normalized bimodality coefficient (BCMVN) maximization strategy, thereby improving doublet detection accuracy.

### Single-cell sample integration, clustering and differential expression analysis

The gene expression matrices were normalized by the NormalizeData function with default parameters by R package Seurat^46^. To adjust for biological sources of variation between samples, R package harmony^47^ with default parameters was used to correct the potential batch effects across patients and sequencing conditions. The corrected embeddings were used only for dimensionality reduction and clustering, while the original log-normalized expression values were used for all differential expression and gene-set-level analyses.

Principal components analysis (PCA) was performed on the integration-transformed expression matrix using the RunPCA function, and the first 15 principal components (PCs) were used in the FindNeighbors function. The resolution parameters of the FindClusters function was 0.3 for all cells.

Uniform manifold approximation and projection (UMAP) was performed for visualization in two dimensions using the RunUMAP function with the same PCs and other default parameters. Major cell lineages were assigned to each cluster of cells using the abundance of canonical marker genes, and marker genes for each cluster were found using the FindAllMarkers function with the parameter “min.pct = 0.25, thresh.use = 0.25”. Notably, the unsupervised clusters of potential doublets that expressed two or more major cell-type lineage markers on the UMAP plot were manually removed to ensure the reliability of the data.

NMF analysis was performed using the cNMF in downsampled tumor cells from scRNA data, with k = 4 as the number of factors^48^. Four expression patterns of tumor cell and the corresponding metagenes were identified. Unsupervised clustering on these metagenes was then performed using NMF consensus. To assess the robustness of NMF clustering, we repeated this analysis ten times by resampling tumor cells each time.

Differential gene analysis was conducted in the identification and annotation of all cells. For the significantly differentially expressed genes, Gene Ontology (GO) functional analysis, Kyoto Encyclopedia of Genes and Genomes (KEGG) pathway analysis, and Gene Set Enrichment Analysis (GSEA) were performed^49^.

### CNV estimation and identification of malignant cells

To identify malignant cells from epithelia, we used the InferCNV to estimate the copy number variations (CNVs)^31^. B cells were used as a normal reference, and the parameters were default. The sum of calculated CNV for each gene per cell was defined as the CNV score of the cell.

### Metabolism analysis

To easily quantify the single-cell metabolic activity of tumor cell and macrophage, we used R package scMetabolism to evaluate each metabolic pathway based on gene sets from KEGG database^32^. The metabolic scores were mapped onto UMAP and visualized using dotplot, with cells grouped by their celltypes.

### Trajectory analysis

We used Monocle2 and Monocle3 to infer the cell lineage trajectory of T cells and myeloid cells based on the top 700 differential signature genes with *q* value < 0.001 calculated by the differentialGeneTest function^50^. The differentiation trajectory, along with directionality, was inferred with the pseudotime structure using default parameters of Monocle, after the procedure of dimension reduction and cell ordering.

### pySCENIC analysis

Single-cell regulatory network inference and clustering (SCENIC) encompasses a tripartite process: co-expression analysis, target gene motif enrichment analysis, and regulon activity evaluation^37^. Initially, GENIE3 is employed to infer co-expression modules between transcription factors and their candidate target genes. Each module comprises a transcription factor alongside its predicted targets, identified purely through patterns of co-expression. Subsequently, RcisTarget is utilized to analyze each co-expressed module, aiming to identify enriched motifs. Only those modules and their targets for which the transcription factor’s motif was significantly enriched are retained, forming a regulon consisting of a transcription factor and its potential direct targets. In the final step, AUCell evaluates the activity level of each regulon within individual cells by calculating the area under the recovery curve, leading to the generation of a regulon activity matrix. This matrix can be binarized by setting an area under the curve (AUC) threshold for each regulon, thereby determining its “active” status in specific cells. The activated regulons across various cell types were analyzed using pySCENIC with raw count matrices as input. GRNboost was applied to compute the co-expression network, while RcisTarget identified regulons. Lastly, AUCell scored regulon activity for each cell, contributing to comprehensive cellular regulatory landscape characterization.

### Cell‑cell interaction analysis

We used CellChat to infer cell-cell interaction between different cell types^30^. This method infers the potential interaction strength between two cell subsets based on gene expression level and provides the significance through a permutation test (1000 times). The enriched ligand-receptor interactions between two cell subsets were calculated based on the permutation test. We extracted significant ligand-receptor pairs with *P* value < 0.01.

### Spatial transcriptomic sequencing and downstream analyses

The spatial features of tissue samples were analyzed using the DynaSpatial FFPE Spatial Gene Expression Kit (Dynamic Biosystems, Suzhou, China) for Human Transcriptome. Specifically, FFPE tissue sections were cut and placed on adhesive slides, followed by H&E staining. The gene expression slides were used to generate spatially barcoded cDNA from tissue sections following the manufacturer’s instructions. Each barcoded DNA array on the slide contained 36864 spots with unique barcoded oligonucleotides and poly(dT)VN within a 6 ×6 mm² capture area. Each spot measured 15 μm in diameter, with a center-to-center distance of 30 μm between adjacent spots. A fiducial frame was printed around the capture areas to ensure proper orientation. Next, the extended probe products were denatured and transferred from the capture area to a new tube, where they were amplified via Polymerase Chain Reaction (PCR) to generate sufficient material. Then, the sequencing library was then prepared following the DynaSpatial library preparation protocol (Dynamic Biosystems, Suzhou, China). Quality inspection of the final library was performed using the Qubit 4.0% & dsDNA HS Assay (Thermo, Q32854) to measure concentration and the Agilent 4150 High Sensitivity D5000 TapeStation to assess library integrity. Finally, paired-end 150 bp sequencing (150PE mode) was conducted on the Illumina NovaSeq X Plus.

The sequencing data were processed through the standard pipeline from DynaSpatial to generate an expression matrix. The R package Seurat was then used for downstream analysis, including the normalization of the expression matrix, Principal Component Analysis (PCA), clustering, and t-SNE dimensionality reduction. In order to identify the specific cell types, we adopted the RCTD approach (R package spacexr, 1.2.0) by using the annotated single-cell atlas of HCC immunotherapy as a reference dataset, and the identification of spatial transcriptome data was then verified by the expression features of each cell type.

### Statistics and reproducibility

The unit of study was the individual patient, independent clinical specimen, individual mouse or independent biological experiment, as appropriate. Technical replicates were not treated as independent biological replicates and were averaged before statistical testing where applicable. Representative images were selected from independently repeated experiments with similar results.

All statistical analyses were performed using R software or GraphPad Prism as appropriate. Data are presented as mean ± standard error of the mean or median with interquartile range, depending on data distribution. Normality was assessed using the Shapiro–Wilk test. The differences between the two groups were analyzed by an unpaired two-tailed Student’s t-test unless otherwise indicated in the corresponding figure legends. For multi-group comparisons, statistical differences were assessed with the Kruskal–Wallis test followed by Dunn’s multiple-comparison test. To assess the association between the relative proportions of all annotated cell types and clinical treatment efficacy, Spearman’s rank correlation coefficient was calculated, given its suitability for evaluating monotonic relationships between continuous or ordinal variables without assuming linearity or normality. Statistical difference results were demonstrated: ns, not significant; *, *P* < 0.05; **, *P* < 0.01; ***, *P* < 0.001; ****P < 0.0001^51^.

### Cell culture of THP-1 and lipid-associated macrophage staining

THP-1 (Pricella) were cultured in RPMI medium with 10 % fetal bovine serum, 1 % penicillin and streptomycin at 37 °C with 5% CO_2_. Cells were seeded in a 24-well plate at a density of 250,000 cells per well. At 24 h after PMA(RPMI containing 100 ng/ml PMA) inducement, cells were washed with PBS and induced by IL-4 and IL-13 (RPMI containing 20 ng/ml IL-4 and 20 ng/ml IL-13) for 72 h. Then, cells were washed with PBS and incubated in the presence of 0.5 ml of fatty acid medium (RPMI containing 250 μM oleic acid) and 0.5 ml Lycopene-fatty acid medium(RPMI containing 250 μM oleic acid and 50 μM lycopene) for 24 h.

Macrophages were incubated in a 24-well plate at a density of 250,000 cells per well. Cells were washed wih PBS and fixed with 4% paraformaldehyde and then incubated at room temperature in the dark for 15 min with 250 μl BODIPY 493/503 staining solution per well. Fluorescence microscope with excitation at approximately 488 nm was used to observe green fluorescence.

### Cell migration and invasion assays

Huh-7 (Pricella) cells were starved by changing the medium to a serum-free medium six hours before the experiment. Subsequently, the cells were resuspended in serum-free medium, adjusted to contain 1 × 10^5^ cells in 100 μL, and plated into 24-well transwell plates. Macrophage and lipid-associated macrophage were seeded in a 24-well plate at a density of 250,000 cells per well. In the invasion assay, the substrate was covered by 2% Matrigel, and the bottom layer was coated with medium with 10% FBS as chemoattractant. The cells were cultured for 12–18 h for migration and invasion assays. Subsequently, the upper chamber was fixed with 4% paraformaldehyde and air-dried at room temperature. After that, it was stained with 4% crystalline violet for 15 min, washed with PBS, and air-dried. Cells in the upper chamber were removed with a cotton swab and photographed in randomly selected fields using an automatic inverted fluorescence microscope. The number and percentage of migrating or invading cells were then calculated.

### Isolation of immune cell and flow cytometry

For PBMC-derived immune cells, peripheral blood mononuclear cells (PBMCs) were isolated from peripheral blood using Lymphoprep density gradient separation according to the manufacturer’s instructions. For tumor-derived immune cells, human or mouse tumor nodules were microdissected from liver tissues, minced into small fragments, and enzymatically digested with collagenase IV (1 mg/mL) and DNase I (0.25 mg/mL) at 37 °C for 60 min. The resulting cell suspensions were filtered through a 70 μm nylon cell strainer. For PBMC-derived T cells, monensin (eBioscience) was added during the final 4 h before intracellular IFN-γ staining. For flow cytometry analysis of mice tumor samples, the obtained single-cell suspensions were incubated for 4 h at 37 °C in the presence of monensin and were subsequently divided into five staining tubes averagely. Separate panels were used to evaluate LAM proportion and different functional aspects of T cells, including effector function (IFN-γ, TNF), proliferation (KI67), exhaustion (PD-1, TIGIT), and regulatory T cells (FOXP3).

The cells obtained after digestion were resuspended in a staining buffer and incubated with Fc block (BioLegend) for 10 min. Then surface antibodies were added and stained for 30 min at 4 °C in the dark. Cells were fixed by IC Fixtion Buffer (ebioscience) for 30 min at 4 °C in the dark, permeabilized by Permeabilization Buffer (Beyotime) for 30 min at 4 °C in the dark and then washed twice with diluted 1×PBS. Subsequently, intracellular antibodies were added and stained for 30 min at 4 °C in the dark. Finally, cells were analyzed by flow cytometer (BD Biosciences).

### Generation and culture of hepatocellular carcinoma organoids

The liver cancer tissues, which had been stored in a preservation solution (DMEM/F12 containing 1% penicillin and streptomycin), were transported to the laboratory, and processed within 24 hours. Initially, the liver cancer tissues were documented, weighed, and measured for volume. Then, the tissues were sectioned into multiple 1-5 mm3 pieces without bias. The pieces were minced into small fragments using surgical scissors, followed by resuspension in preservation solution (10 mL). The suspension was filtered through a strainer (Falcon, 100 µm) with gentle grinding and pushing of tissue masses through the strainer using a syringe plunger (5 mL). After rinsing, any residual impurities on the membrane were discarded along with the strainer. Subsequently, the filtrate containing cell clusters and single cells was strained through a strainer (40 µm) to collect the cell clusters with sizes ranging from 40 to 100 µm. The filter membrane was separated from the strainer, placed in liver cancer organoid medium (HCOM, 2 mL), and thoroughly washed with a pipette to release cell clusters into the media. The membrane was then discarded, and samples were cultured in suspension overnight. The success rate of HCC organoids construction is approximately 60% and the clinical data of corresponding patients was reported in Supplementary Table 2.

For culturing pHCOs in a multi-well plate, the pHCOs in suspension were centrifuged at 500 × g for 5 min at 4 °C and then resuspended in cold growth factor-reduced Matrigel (BD Biosciences). Subsequently, drops (60 µL) of the Matrigel cell cluster suspension were plated onto ultra-low attachment 96- or 48-well plates with a flat bottom (Corning) and allowed to solidify at 37 °C for 20 min. The seeding density was adjusted to about 500 organoids per well. Once Matrigel had solidified, HCOM (100 µL) was added to each well, and the plate was transferred to a cell culture incubator (37 °C with 5% CO2). The HCOM medium required to be refreshed at least twice a week.

### Co-culture assay for T cells-macrophages and T cells-HCOs

Human CD3^+^ T cells were isolated by MACS and cultured in ImmunoCult-XF T cell expansion medium for 72 h. Macrophage and LAM were induced from THP-1 cell line defined in the method, and were cultured with CD3^+^ T cells (Macrophage: CD3^+^ T cells ratio=2:1) in transwell for 12 h. Subsequently, T cells were used for flow cytometry analysis and further co-culture respectively. The HCOs were distributed in the round bottom 96-well plate for 10–20 organoids each well, containing approximately 500–1000 tumor cells in total of each well. Then introduced homologous T cells with an effector-target ratio of 3:1 for 48 h. The HCOs were then recovered for Calcein AM/PI staining and image quantification through Imagej software. The viability of HCO was calculated by dividing live cell signals into total cell signals.

### Moue models and treatments

C57BL/6 J (male) mice were purchased from Beijing Vital River Experimental Animal Technology (Beijing, China). Chiglitazar was purchased from Chipscreen Biosciences Ltd. (Shenzhen, China). Chiglitazar was first dissolved in dimethyl sulfoxide (DMSO) to prepare a stock solution (10 mg/ml) and subsequently diluted with sterile normal saline to a final administration volume of 0.4 mL (1 mg/ml) per mouse for oral gavage. All animals were maintained in a specific pathogen-free (SPF) barrier with a 12-h light/12-h dark cycle at an ambient temperature of 22 °C ± 2 °C and relative humidity of 40–70%. facility at the Animal Center of Shandong University. All animal experiments were approved by the Committee on Ethics of Animal Experiments at the Qilu hospital of Shandong University (IACUC Issue NO. DWLL-202500262), and were performed in accordance with the Guide for the Care and Use of Laboratory Animals. To establish HCC model, C57BL/6 mice (18 weeks old) either fed with HFD (mice with high-fat diet, 45% fat, 12 weeks) or RD (mice with regular diet, 12 weeks) were injected in situ with 7 × 10^5^ Hepa 1–6 cells (Pricella) in 20 μl (PBS: Matrigel=1:1) after anesthetized by 0.5% pentobarbital sodium. Seven days after inoculation with Hepa 1–6 cells, mice were imaged by bioluminescence to confirm the successful and uniform establishment of HCC models. Mice were then divided to four groups (5 mice/group), including RD, HFD, HFD+lycopene (mice with high-fat diet and lycopene, 10 mg/kg, oral gavage) and HFD+chiglitazar (mice with high-fat diet and chiglitazar, 10 mg/kg, oral gavage). Mice were treated by intraperitoneal injection with anti-PD-1 mAb (10 mg/kg) three times at 2-day intervals and were treated by oral gavage with lycopene or chiglitazar seven times at 1-day intervals. HCC specimens were collected and the weight was measured at 14th day. Additionally, animal experiments in this study were conducted using male mice only in order to exclude the influence of hormonal cycling, which substantially affects lipid metabolism and immune responses.

### Oil red O staining

Fresh liver tumor tissues were embedded in optimal cutting temperature (OCT) compound and sectioned into 8-μm cryosections. Sections were fixed in 4% paraformaldehyde for 15 min at room temperature, rinsed with distilled water, and incubated in 60% isopropanol for 5 min. Slides were then stained with freshly prepared Oil Red O working solution (0.5% Oil Red O in isopropanol, diluted 3:2 with distilled water and filtered before use) for 15 min at room temperature. After washing with 60% isopropanol and distilled water, nuclei were counterstained with hematoxylin for 1 min. Sections were mounted in aqueous mounting medium and imaged immediately. Brightfield images were acquired under identical microscope settings for all samples. For quantitative analysis, at least three non-overlapping random fields per section were captured, and three sections per sample were analyzed. Oil Red O–positive areas were quantified using ImageJ software (NIH). Images were converted to RGB stacks, and a standardized color threshold was applied uniformly across all samples to identify lipid-positive regions. The percentage of Oil Red O–positive area relative to the total tissue area within each field was calculated. The mean percentage of positive area per mouse was used for statistical analysis.

### Western blotting

HCC tumor tissues were lysed in radioimmunoprecipitation assay (RIPA) buffer (Beyotime Biotechnology) supplemented with protease and phosphatase inhibitor cocktails on ice for 30 min. Lysates were centrifuged at 12,000 × g for 15 min at 4 °C, and the supernatants were collected. Protein concentrations were determined using the BCA Protein Assay Kit (Beyotime Biotechnology). Equal amounts of protein (30 μg per lane) were mixed with 1× SDS loading buffer and boiled at 95 °C for 5 min. Proteins were separated by SDS–polyacrylamide gel electrophoresis (10% gels) and transferred onto polyvinylidene difluoride (PVDF) membranes at 300 mA for 60 min at 4 °C. Membranes were blocked with 5% non-fat milk in tris-buffered saline containing 0.1% Tween-20 (TBST) for 1 h at room temperature and incubated overnight at 4 °C with primary antibodies. After washing three times with TBST, membranes were incubated with HRP-conjugated secondary antibodies for 1 h at room temperature. Protein bands were visualized using enhanced chemiluminescence substrate (Vazyme) and imaged using a UVP chemiluminescence imaging system (Upland). β-actin was used as a loading control.

### Real-time quantitative PCR (RT-qRCR)

Total RNA was extracted from cells using TRIzol reagent (Yeasen) according to the manufacturer’s instructions. RNA concentration and purity were assessed using a NanoDrop spectrophotometer (NanoDrop Technologies), and samples with A260/280 ratios between 1.8 and 2.0 were used for downstream analysis. For reverse transcription, 1 μg of total RNA was converted into complementary DNA (cDNA) using HiScript qRT-PCR SuperMix (Vazyme) in a 20 μL reaction volume according to the manufacturer’s protocol. Quantitative real-time PCR (RT-qPCR) was performed using AceQ qPCR SYBR Green Master Mix (Vazyme) in a 20 μL reaction system containing 10 μL SYBR Green Master Mix, 0.4 μM forward and reverse primers, and 2 μL cDNA template. Amplification was carried out on an ABI 7500 real-time PCR system under the following cycling conditions: 95 °C for 5 min, followed by 40 cycles of 95 °C for 10 s and 60 °C for 30 s. GAPDH was used as an internal reference gene for normalization. Relative gene expression levels were calculated using the 2^−ΔΔCt^ method. All reactions were performed in triplicate. The primers were synthesized by Tsingke Biotech and the sequences are listed in Experimental Supplies (Supplementary Table 15).

### Reporting summary

Further information on research design is available in the Nature Portfolio Reporting Summary linked to this article.

## Supplementary information


Supplementary Information
Reporting Summary
Transparent Peer Review file


## Source data


Source Data


## Data Availability

The raw scRNA-seq data generated in this study have been deposited in the Genome Sequence Archive for Human (GSA-Human) under project number HRA011844. As related to human genetic resource, the data deposited and made public for non-commercial purposes, and are compliant with the regulations of the Ministry of Science and Technology of China. The publicly available datasets used in this study are available under accession codes (skrx2fz79n. GSE149614, GSE151530, GSE156625, GSE189903, GSE202642 and GSE206325. All other data are available in the article and its Supplementary files or from the corresponding author upon request. Source data are provided with this paper.

## Code availability

The codes used for bioinformatic analyses in this study are deposited at GitHub (https://github.com/Macr0phage/scRNA-seq-of-HCC-immunotherapy) (https://doi.org/10.5281/zenodo.20664347).

## References

[1] Bray, F. et al. Global cancer statistics 2022: GLOBOCAN estimates of incidence and mortality worldwide for 36 cancers in 185 countries. *CA Cancer J. Clin.* **74**, 229–263 (2024). 38572751 10.3322/caac.21834

[2] Li, L. & Wang, H. Heterogeneity of liver cancer and personalized therapy. *Cancer Lett.* **379**, 191–197 (2016). 26213370 10.1016/j.canlet.2015.07.018

[3] Gabbia, D. & De Martin, S. Insights into hepatocellular carcinoma: from pathophysiology to novel therapies. *Int. J. Mol. Sci.* **25**, 4188 (2024). 10.3390/ijms25084188 PMC11049888 38673774

[4] Finn, R. S. et al. Atezolizumab plus Bevacizumab in unresectable hepatocellular carcinoma. *N. Engl. J. Med* **382**, 1894–1905 (2020). 32402160 10.1056/NEJMoa1915745

[5] Qin, S. et al. Camrelizumab plus rivoceranib versus sorafenib as first-line therapy for unresectable hepatocellular carcinoma (CARES-310): a randomised, open-label, international phase 3 study. *Lancet* **402**, 1133–1146 (2023). 37499670 10.1016/S0140-6736(23)00961-3

[6] El-Khoueiry, A. B. et al. Nivolumab in patients with advanced hepatocellular carcinoma (CheckMate 040): an open-label, non-comparative, phase 1/2 dose escalation and expansion trial. *Lancet* **389**, 2492–2502 (2017). 28434648 10.1016/S0140-6736(17)31046-2 PMC7539326

[7] Melero, I. et al. Nivolumab plus ipilimumab combination therapy in patients with advanced hepatocellular carcinoma previously treated with sorafenib: 5-year results from CheckMate 040. *Ann. Oncol.* **35**, 537–548 (2024). 38844309 10.1016/j.annonc.2024.03.005

[8] Chen, Q. F., Chen, S., Chen, M., Lyu, N. & Zhao, M. Improving the conversion success rate of hepatocellular carcinoma: focus on the use of combination therapy with a high objective response rate. *J. Clin. Transl. Hepatol.* **12**, 298–304 (2024). 38426191 10.14218/JCTH.2023.00403 PMC10899866

[9] Qin, S. et al. Tislelizumab vs sorafenib as first-line treatment for unresectable hepatocellular carcinoma: a phase 3 randomized clinical trial. *JAMA Oncol.* **9**, 1651–1659 (2023). 37796513 10.1001/jamaoncol.2023.4003 PMC10557031

[10] Kim, H. D. et al. Regorafenib plus nivolumab in unresectable hepatocellular carcinoma: the phase 2 RENOBATE trial. *Nat. Med* **30**, 699–707 (2024). 38374347 10.1038/s41591-024-02824-y PMC10957471

[11] Guo, X. et al. Contrasting cytotoxic and regulatory T cell responses underlying distinct clinical outcomes to anti-PD-1 plus lenvatinib therapy in cancer. *Cancer Cell* **43**, 248–268.e249 (2025). 39889705 10.1016/j.ccell.2025.01.001

[12] Xue, R. et al. Liver tumour immune microenvironment subtypes and neutrophil heterogeneity. *Nature* **612**, 141–147 (2022). 36352227 10.1038/s41586-022-05400-x

[13] Cheng, Y. et al. Stromal architecture and fibroblast subpopulations with opposing effects on outcomes in hepatocellular carcinoma. *Cell Discov.* **11**, 1 (2025). 39870619 10.1038/s41421-024-00747-z PMC11772884

[14] Magen, A. et al. Intratumoral dendritic cell-CD4(+) T helper cell niches enable CD8(+) T cell differentiation following PD-1 blockade in hepatocellular carcinoma. *Nat. Med.* **29**, 1389–1399 (2023). 37322116 10.1038/s41591-023-02345-0 PMC11027932

[15] Huang, J., Wu, Q., Geller, D. A. & Yan, Y. Macrophage metabolism, phenotype, function, and therapy in hepatocellular carcinoma (HCC). *J. Transl. Med.* **21**, 815 (2023). 37968714 10.1186/s12967-023-04716-0 PMC10652641

[16] Zeng, F. et al. Multimodal sequencing of neoadjuvant nivolumab treatment in hepatocellular carcinoma reveals cellular and molecular immune landscape for drug response. *Mol. Cancer* **24**, 110 (2025). 40205519 10.1186/s12943-025-02314-w PMC11980310

[17] Wang, C. et al. Macrophage polarization and its role in liver disease. *Front. Immunol.* **12**, 803037 (2021). 34970275 10.3389/fimmu.2021.803037 PMC8712501

[18] Qiu, X. et al. Spatial single-cell protein landscape reveals vimentin(high) macrophages as immune-suppressive in the microenvironment of hepatocellular carcinoma. *Nat. Cancer* **5**, 1557–1578 (2024). 39327501 10.1038/s43018-024-00824-y

[19] Lu, Y. et al. A single-cell atlas of the multicellular ecosystem of primary and metastatic hepatocellular carcinoma. *Nat. Commun.* **13**, 4594 (2022). 35933472 10.1038/s41467-022-32283-3 PMC9357016

[20] Guo, H. et al. TREM2 promotes the formation of a tumor-supportive microenvironment in hepatocellular carcinoma. *J. Exp. Clin. Cancer Res.* **44**, 20 (2025). 39838454 10.1186/s13046-025-03287-w PMC11748316

[21] Li, Y. et al. Single cell analyses reveal the PD-1 blockade response-related immune features in hepatocellular carcinoma. *Theranostics* **14**, 3526–3547 (2024). 38948071 10.7150/thno.95971 PMC11209711

[22] Wang, H. et al. POSTN(+) cancer-associated fibroblasts determine the efficacy of immunotherapy in hepatocellular carcinoma. *J. Immunother. Cancer* **12**, e008721 (2024). 10.1136/jitc-2023-008721 PMC11284881 39067872

[23] Cappuyns, S. et al. Single-cell RNA sequencing-derived signatures define response patterns to atezolizumab + bevacizumab in advanced hepatocellular carcinoma. *J. Hepatol.* **82**, 1036–1049 (2024). 10.1016/j.jhep.2024.12.016 PMC12086051 39709141

[24] Ma, L. et al. Single-cell atlas of tumor cell evolution in response to therapy in hepatocellular carcinoma and intrahepatic cholangiocarcinoma. *J. Hepatol.* **75**, 1397–1408 (2021). 34216724 10.1016/j.jhep.2021.06.028 PMC8604764

[25] Sharma, A. et al. Onco-fetal reprogramming of endothelial cells drives immunosuppressive macrophages in hepatocellular carcinoma. *Cell* **183**, 377–394.e321 (2020). 32976798 10.1016/j.cell.2020.08.040

[26] Ma, L. et al. Multiregional single-cell dissection of tumor and immune cells reveals stable lock-and-key features in liver cancer. *Nat. Commun.* **13**, 7533 (2022). 36476645 10.1038/s41467-022-35291-5 PMC9729309

[27] Zhu, G. Q. et al. CD36(+) cancer-associated fibroblasts provide immunosuppressive microenvironment for hepatocellular carcinoma via secretion of macrophage migration inhibitory factor. *Cell Discov.* **9**, 25 (2023). 36878933 10.1038/s41421-023-00529-z PMC9988869

[28] Zhou, J. et al. China liver cancer guidelines for the diagnosis and treatment of hepatocellular carcinoma (2024 Edition). *Liver Cancer* **14**, 779–835 (2025). 41063733 10.1159/000546574 PMC12503762

[29] D’Alessio, A. et al. Pathological response following neoadjuvant immune checkpoint inhibitors in patients with hepatocellular carcinoma: a cross-trial, patient-level analysis. *Lancet Oncol.* **25**, 1465–1475 (2024). 39437804 10.1016/S1470-2045(24)00457-1 PMC12040480

[30] Jin, S. et al. Inference and analysis of cell-cell communication using CellChat. *Nat. Commun.* **12**, 1088 (2021). 33597522 10.1038/s41467-021-21246-9 PMC7889871

[31] Patel, A. P. et al. Single-cell RNA-seq highlights intratumoral heterogeneity in primary glioblastoma. *Science* **344**, 1396–1401 (2014). 24925914 10.1126/science.1254257 PMC4123637

[32] Wu, Y. et al. Spatiotemporal immune landscape of colorectal cancer liver metastasis at single-cell level. *Cancer Discov.* **12**, 134–153 (2022). 34417225 10.1158/2159-8290.CD-21-0316

[33] Liu, Y. et al. Identification of a tumour immune barrier in the HCC microenvironment that determines the efficacy of immunotherapy. *J. Hepatol.* **78**, 770–782 (2023). 36708811 10.1016/j.jhep.2023.01.011

[34] Trapnell, C. et al. The dynamics and regulators of cell fate decisions are revealed by pseudotemporal ordering of single cells. *Nat. Biotechnol.* **32**, 381–386 (2014). 24658644 10.1038/nbt.2859 PMC4122333

[35] Cao, J. et al. The single-cell transcriptional landscape of mammalian organogenesis. *Nature* **566**, 496–502 (2019). 30787437 10.1038/s41586-019-0969-x PMC6434952

[36] Zhang, Y. et al. CellCall: integrating paired ligand-receptor and transcription factor activities for cell-cell communication. *Nucleic Acids Res.* **49**, 8520–8534 (2021). 34331449 10.1093/nar/gkab638 PMC8421219

[37] Aibar, S. et al. SCENIC: single-cell regulatory network inference and clustering. *Nat. Methods* **14**, 1083–1086 (2017). 28991892 10.1038/nmeth.4463 PMC5937676

[38] Yang, X. et al. FABP5(+) lipid-loaded macrophages process tumour-derived unsaturated fatty acid signal to suppress T-cell antitumour immunity. *J. Hepatol.* **82**, 676–689 (2025). 39357545 10.1016/j.jhep.2024.09.029

[39] Liu, C. et al. Patient-derived tumor organoids combined with function-associated ScRNA-seq for dissecting the local immune response of lung cancer. *Adv. Sci.* **11**, e2400185 (2024). 10.1002/advs.202400185 PMC11336893 38896792

[40] Wu, Y. et al. Grouped-seq for integrated phenotypic and transcriptomic screening of patient-derived tumor organoids. *Nucleic Acids Res.* **50**, e28 (2022). 34893868 10.1093/nar/gkab1201 PMC8934649

[41] Zhao, W. et al. Transarterial chemoembolization (TACE)-hepatic arterial infusion chemotherapy (HAIC) combined with PD-1 inhibitors plus lenvatinib as a preoperative conversion therapy for nonmetastatic advanced hepatocellular carcinoma: a single center experience. *Transl. Cancer Res.* **13**, 2315–2331 (2024). 38881913 10.21037/tcr-24-93 PMC11170507

[42] Zhu, C., Dai, B., Zhan, H. & Deng, R. Neoadjuvant transarterial chemoembolization (TACE) plus PD-1 inhibitor bridging to tumor resection in intermediate-stage hepatocellular carcinoma patients. *Ir. J. Med. Sci.* **192**, 1065–1071 (2023). 35996068 10.1007/s11845-022-03131-6

[43] Zhang, Y. et al. Spatiotemporal dynamics of T cells in peripheral blood and tumor underlying differential responses to neoadjuvant PD-1 blockade in hepatocellular carcinoma. *J. Immunother. Cancer* **13**, e013516 (2025). 10.1136/jitc-2025-013516 PMC12673557 41330615

[44] Pfister, D. et al. NASH limits anti-tumour surveillance in immunotherapy-treated HCC. *Nature* **592**, 450–456 (2021). 33762733 10.1038/s41586-021-03362-0 PMC8046670

[45] Bi, X. et al. Consensus of Chinese experts on neoadjuvant and conversion therapies for hepatocellular carcinoma: 2023 update. *Liver Cancer* **14**, 223–238 (2025). 40255878 10.1159/000541249 PMC12005702

[46] Hao, Y. et al. Dictionary learning for integrative, multimodal and scalable single-cell analysis. *Nat. Biotechnol.* **42**, 293–304 (2024). 37231261 10.1038/s41587-023-01767-y PMC10928517

[47] Korsunsky, I. et al. Fast, sensitive and accurate integration of single-cell data with Harmony. *Nat. Methods* **16**, 1289–1296 (2019). 31740819 10.1038/s41592-019-0619-0 PMC6884693

[48] Kotliar, D. et al. Identifying gene expression programs of cell-type identity and cellular activity with single-cell RNA-Seq. *Elife* **8**, 43803 (2019). 10.7554/eLife.43803 PMC6639075 31282856

[49] Wu, T. et al. clusterProfiler 4.0: a universal enrichment tool for interpreting omics data. *Innovation* **2**, 100141 (2021). 34557778 10.1016/j.xinn.2021.100141 PMC8454663

[50] Qiu, X. et al. Single-cell mRNA quantification and differential analysis with Census. *Nat. Methods* **14**, 309–315 (2017). 28114287 10.1038/nmeth.4150 PMC5330805

[51] Ibrahimi, E. & Wolford, B. N. Ten essential tips for robust statistics in cell biology. *Nat. Cell Biol.* **27**, 1884–1886 (2025). 41174003 10.1038/s41556-025-01801-y

