# Supplementary material for: Integrated single-cell multi-omics characterization reveals lipid-associated macrophage-mediated immunosuppression in neoadjuvant immunotherapy of hepatocellular carcinoma
Source: Nat Commun. 2026 Jul 31;17:9381. doi: 10.1038/s41467-026-75949-y (PMC13534469; doi:10.1038/s41467-026-75949-y)
Supplement: Supplementary file 1 [file 41467_2026_75949_MOESM1_ESM.pdf]

Supplementary information

Integrated Single-cell Multi-omics Characterization Reveals Lipid-associated Macrophage-mediated Immunosuppression in Neoadjuvant Immunotherapy of Hepatocellular Carcinoma

Shengxuan Peng[1#], Chang Liu[1#], Junyu Long[2#], Jincheng Tian[1], Han Li[1], Donghai Lu[1], Qihang Cao[1], Daolin Zhang[1], Qiao He[1], Jisen Jia[1], Yuxuan Wang[1], Zhaoru Dong[1], Haitao Zhao[2], Lei Zhao[3,4]*, Dongxu Wang[1]*, Tao Li[1]*

[#] Shengxuan Peng, Chang Liu, Junyu Long contributed equally to this work.

*Corresponding author: Tao Li, E-mail: litao7706@163.com
Dong-Xu Wang, E-mail: drwangdongxu@163.com
Lei Zhao, E-mail: drzhaolei@hotmail.com

This supplementary information file includes:

Supplementary Figures 1-8, (Supplementary Fig. 1-8)

Legends for Supplementary Figures 1-8

Other supplementary materials for this manuscript include the following:

Supplementary Tables 1-15

Legends for Supplementary Tables 1-15

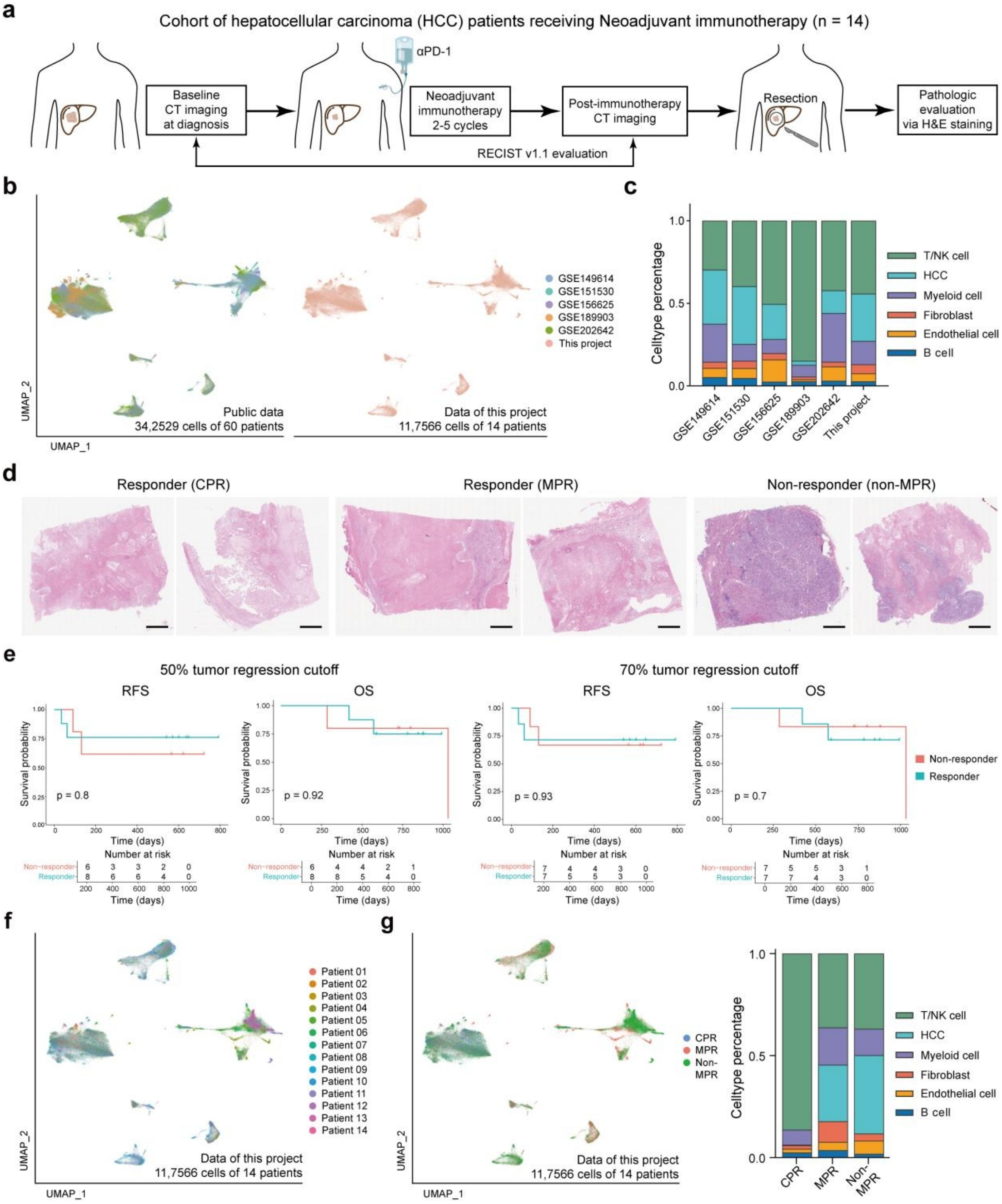

Supplementary Fig. 1 | Supplementary information of single-cell atlas and clinical status of HCC immunotherapy cohort, related to Fig. 1. a Schematic of neoadjuvant immunotherapy for HCC patients. Images provided by Servier Medical Art (https://smart.servier.com), licensed under CC BY 4.0 (https://creativecommons.org/licenses/by/4.0/). **b** UMAP plot of cells from five external database and in-house data. **c** Bar plots indicating the proportion of major cell types in each database. **d** H&E slides of typical patients with CPR, MPR and non-MPR clinical outcomes. The classification of non-MPR, MPR, and CPR are based on residual viable tumor percentages of ≥50%, 0–50%, and 0%. Scale bar: 1 cm. **e** Kaplan–Meier survival analyses of recurrence-free survival (RFS) and overall survival (OS) stratified by pathological response using two cutoff criteria: 50% tumor regression (non-responders [non-MPR] vs responders [MPR + CPR]) and 70% tumor regression. **f** UMAP plots of all cells colored by individual patients from our HCC immunotherapy cohort. **g** UMAP plots and bar plots of all cells grouped by clinical outcomes.

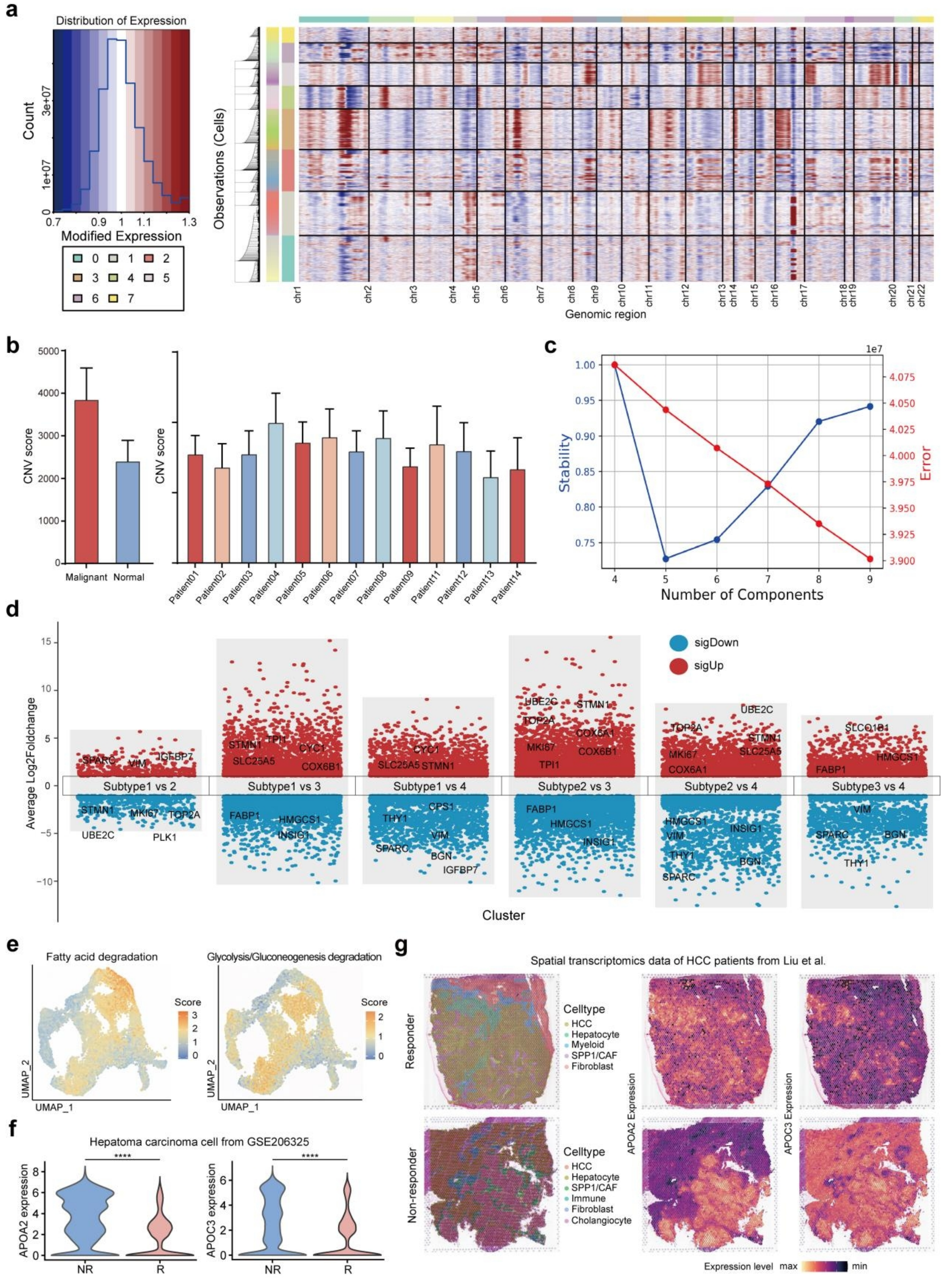

Supplementary Fig. 2 | Supplementary analyses of HCC characterization. a Heatmap showing large-scale CNVs for individual HCC cells. Each row represented a cell and the columns represented chromosomal regions. **b** Bar plots showing the CNV scores of malignant and benign cells and CNV scores from each individual patient. Bars represent the mean CNV score across individual cells, and error bars indicate the standard deviation. Each cell was treated as an individual observation. **c** Line chart of NMF clustering of malignant cells, 4 subtypes is the most stable clustering. **d** Volcano plot of differentially expressed genes (DEGs) for each paired

malignant subtype. **e** Features of fatty acid metabolism and glucose metabolism among malignant cells determined by scMetabolism. **f** Expression levels of *APOA2* and *APOC3* among patients with different clinical outcomes of external HCC immunotherapy cohort (scRNA-seq from GSE206325, NR, n=35051 cells, R, n=17906 cells, the P value is below the numerical reporting threshold of the statistical software). **g** Spatial transcriptomics analyses of external HCC immunotherapy cohort (Mendeley data, skrx2fz79n) showing distinct expression level of *APOA2* and *APOC3* among typical responder and non-responder.

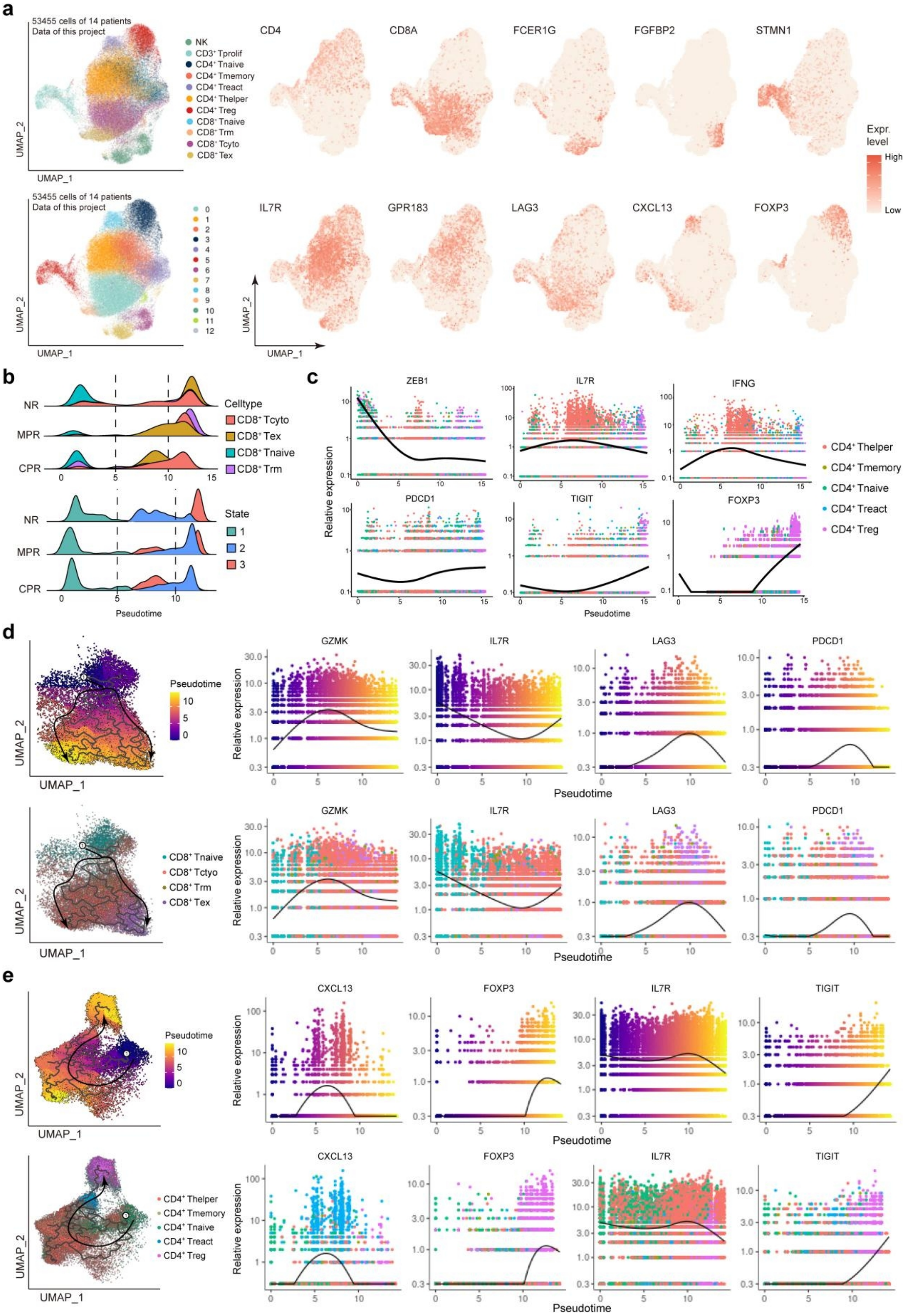

Supplementary Fig. 3 | Supplementary analyses for subtypes of T/NK cells. a UMAP plots showing the T/NK cells from our HCC immunotherapy cohort, colored by subtypes and unsupervised clusters. The featureplots showed T/NK expression features. **b** Distribution of different states and subtypes of $CD8^+$ T cells along pseudotime trajectories of three clinical efficacy groups. **c** The change in expression levels of functional genes in $CD4^+$ T cells along the

axis of pseudotime in complement with Fig. 3H, including naïve (*ZEB1*, *IL7R*), effect (*IFNG*) and exhaustion (*PDCD1*, *TIGIT*, *FOXP3*) features. **d** Monocle3 analysis of $CD8^+$ T cells related to Fig. 3E, along with the change in expression of functional genes in $CD8^+$ T cells along the axis of pseudotime, including *GZMK*, *IL7R*, *LAG3* and *PDCD1*. **e** Monocle3 analysis of $CD4^+$ T cells related to Fig. 3H, along with the change in expression of functional genes in $CD4^+$ T cells along the axis of pseudotime, including *CXCL13*, *FOXP3*, *IL7R* and *TIGIT*.

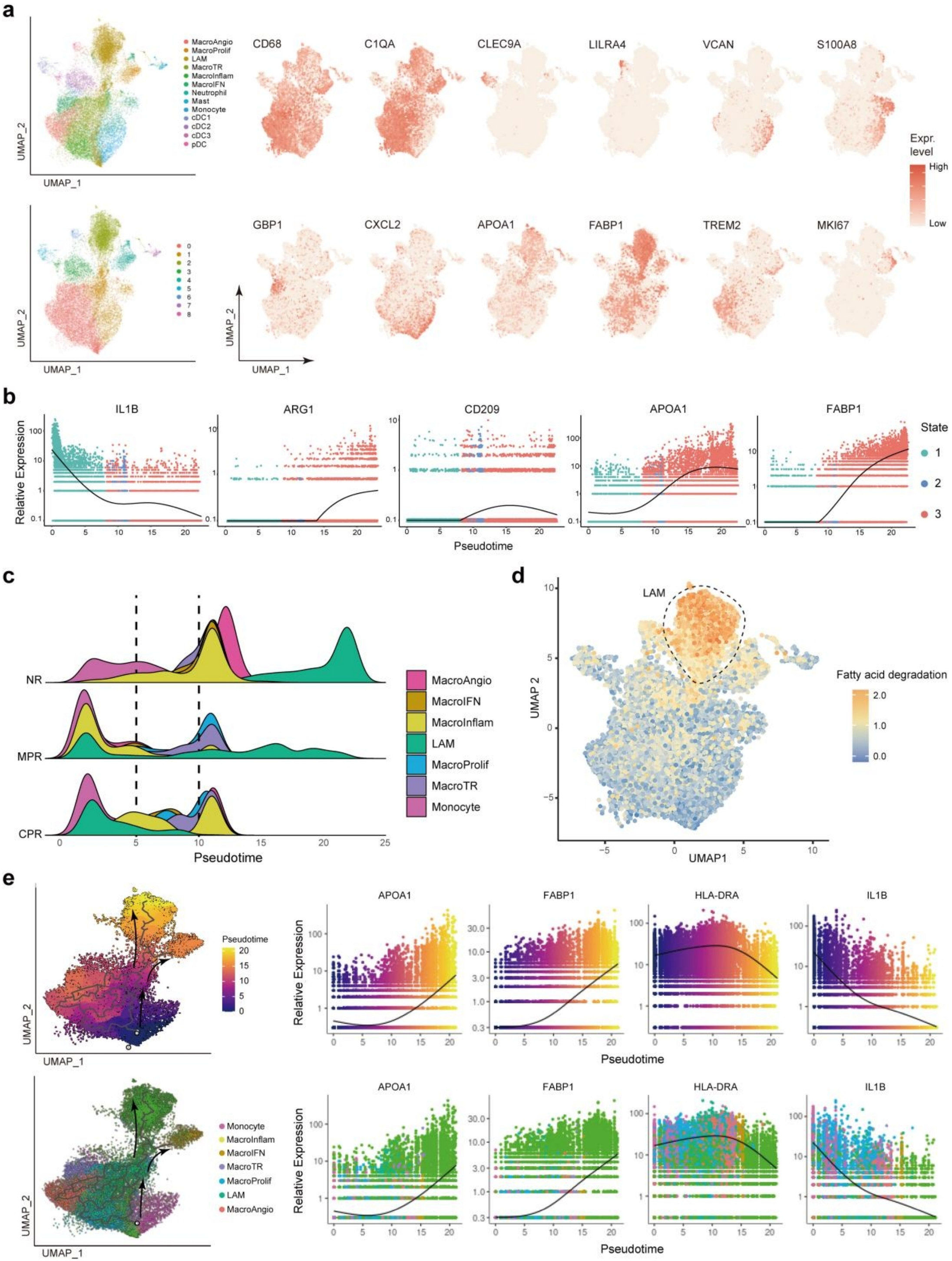

Supplementary Fig. 4 | Supplementary analyses for subtypes of myeloid cells. a UMAP plots showing the myeloid cells from our HCC immunotherapy cohort, colored by subtypes and unsupervised clusters. The featureplots showed myeloid expression features. **b** The change in expression levels of functional genes in macrophage along the axis of pseudotime, including M1 (*IL1B*), M2 (*ARG1*, *CD209*) and lipid metabolism (*APOA1*, *FABP1*) features. **c** Distribution of different subtypes of myeloid cells along pseudotime trajectories of three clinical efficacy groups. **d** Metabolism features of LAM calculated by scMetabolism. **e** Monocle3 analysis of monocytes and macrophages related to Fig. 4D, along with expression levels of functional genes in macrophages along the axis of pseudotime, including *APOA1*, *FABP1*, *HLA-DRA* and *IL1B*.

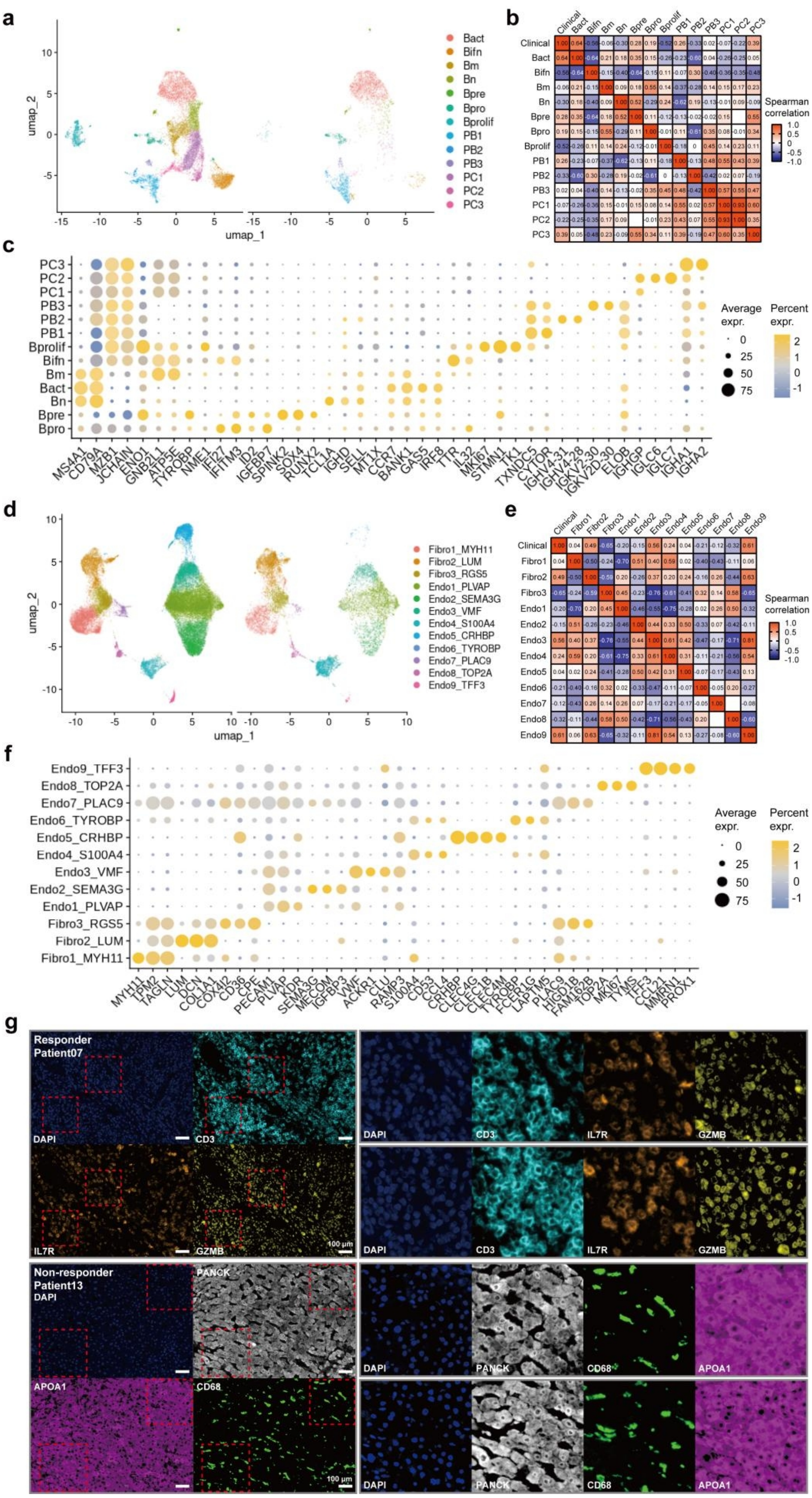

Supplementary Fig. 5 | Subtypes identification and clinical correlation of B cells, fibroblasts and endothelial cells. a UMAP plot of B cells colored by subtypes. **b** Matrix of one-sided Spearman correlation analysis across clinical outcomes and proportions of B cell subtypes from

HCC immunotherapy cohort. **c** Gene expression features of 13 B cell subtypes. **d** UMAP plot of endothelial cells and fibroblasts colored by subtypes. **e** Matrix of one-sided Spearman correlation analysis across clinical outcomes and proportions of endothelial cell and fibroblast subtypes from HCC immunotherapy cohort. **f** Gene expression features of 12 endothelial cell and fibroblast subtypes. **g** Representative mIHC images of splited single-channels for representative TIME features (PANCK, CD3, IL7R, GZMB, CD68 and APOA1), corresponding to the regions shown in **Fig. 5e**. Enlarged views of representative regions highlight $APOA1^{+}$ $CD68^{+}$ LAMs enriched in non-responder. Nuclei were stained with DAPI. Scale bars, 100 μm.

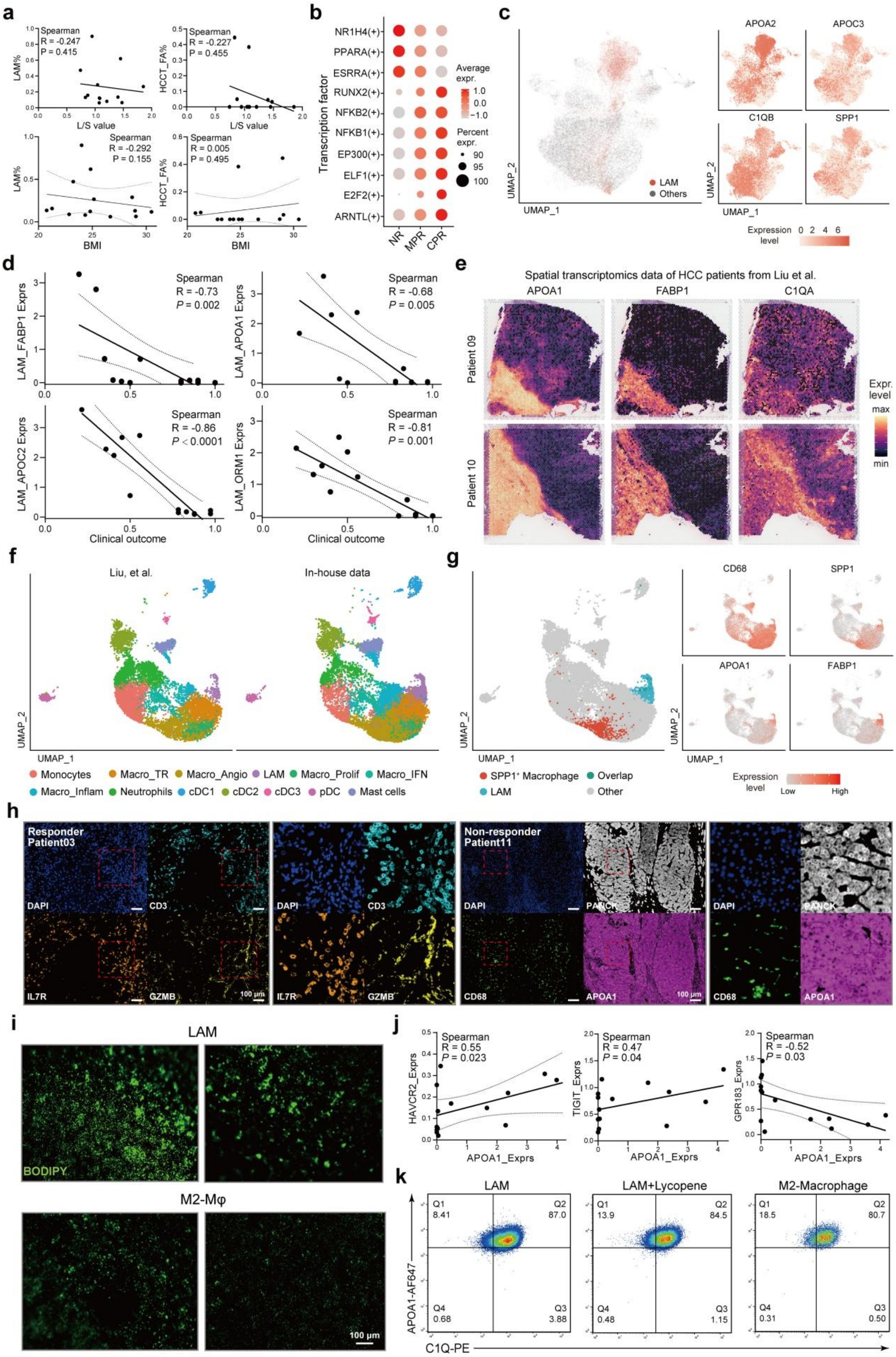

Supplementary Fig. 6 | Additional transcriptomic analyses and functional evaluation of LAM. **a** Correlation analysis between radiologic liver-to-spleen (L/S) attenuation ratio or BMI and proportions of LAM or HCCT_FA subsets among HCC patients of our cohort. **b** Enrichment

analysis of transcription factors of LAM in different clinical efficacy groups, calculated by SCENIC approach. **c** UMAP plot and feature plots showing the expression features of LAM, including *C1QB*, *APOA2*, *APOC3* and *SPP1*. **d** Spearman correlation analysis between clinical outcomes and expression levels of LAM features, including *FABP1*, *APOA1*, *APOC2* and *ORM1*. **e** External spatial transcriptomic dataset (Mendeley data, skrx2fz79n) also demonstrated the LAM-related features expressed in HCC patients, including *C1QA*, *APOA1* and *FABP1*. **f** Integrated single-cell atlas of external HCC immunotherapy scRNA-seq dataset (Mendeley data, skrx2fz79n) and in-house dataset. Spatial distributions of the $SPP1^{+}$ macrophages pre-defined in research of Liu, et al. and the LAMs identified in our study on UMAP embedding. **g** Featureplots showing expression levels of *CD68*, *SPP1* and lipid associated genes (*FABP1*, *APOA1*), indicating the shared features and especially distinctions of two subtypes. **h** Representative mIHC images of splited single-channels for representative TIME features (PANCK, CD3, IL7R, GZMB, CD68 and APOA1), corresponding to the regions shown in **Fig. 6c**. Enlarged views of representative regions highlight $APOA1^{+}$ $CD68^{+}$ LAMs within the TIME of non-responder. Nuclei were stained with DAPI. Scale bars, 100 μm. **i** Fluorescence imaging of lipid droplets in LAM and M2-macrophages stained with BODIPY (n=5). Scale bars, 100 μm. **j** Spearman correlation analysis between expression levels of LAM and T cell functional genes. **k** Flow cytometry confirming expression of APOA1 and C1Q features related to LAM. The expression of C1Q and APOA1 of macrophage induced with lycopene was lower than LAM. (Correlation analyses were assessed using one-sided Spearman correlation without multiple comparisons adjustments)

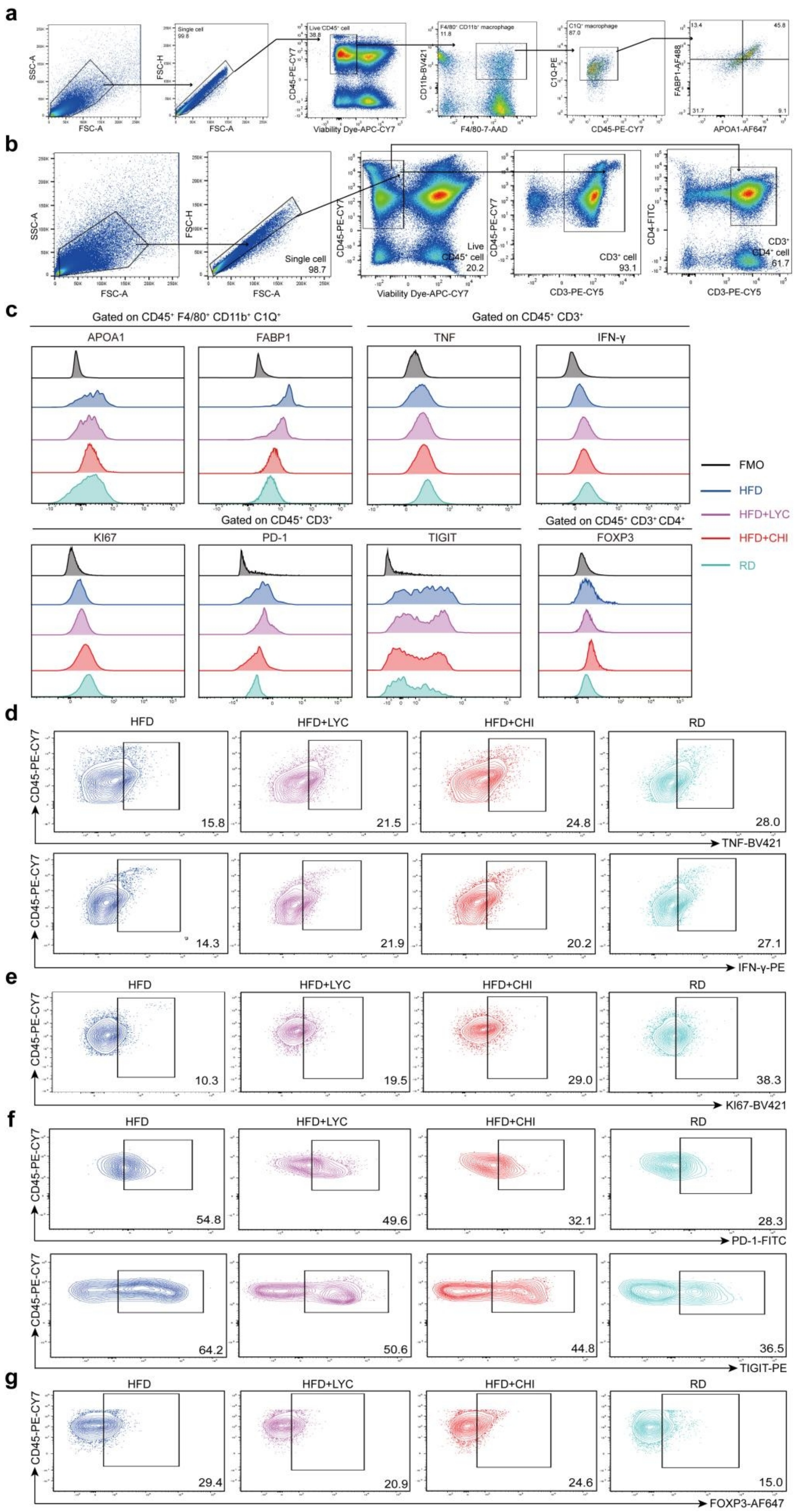

Supplementary Fig. 7 | High-fat diet–induced lipid-associated macrophages impair T cell function in a murine HCC model, related to Fig. 7f. Tumor tissues were analyzed by flow cytometry for intratumoral T cell function (Gated on CD45^{+} CD3^{+}) and LAM proportion (Gated

on $CD45^+$ $F4/80^+$ $CD11b^+$ $C1Q^+$ $APOA1^+$ $FABP1^+$). **a** Gating strategy of mice LAM. **b** Gating strategy of T cells. **c-g** Fluorescence minus one (FMO) controls were included for APOA1, FABP1, TNF, IFN-γ, KI67, PD-1, TIGIT and FOXP3 staining to define gating boundaries and ensure accurate discrimination of positive populations. Representative FMO plots are shown. T cell functions were analyzed from the aspects of **(d)** effector cytokine production (TNF and IFN-γ), **(e)** proliferative capacity (Ki-67), **(f)** exhaustion (PD-1 and TIGIT), as well as **(g)** quantification of Tregs by FOXP3 (Gated on $CD45^+$ $CD3^+$ $CD4^+$).

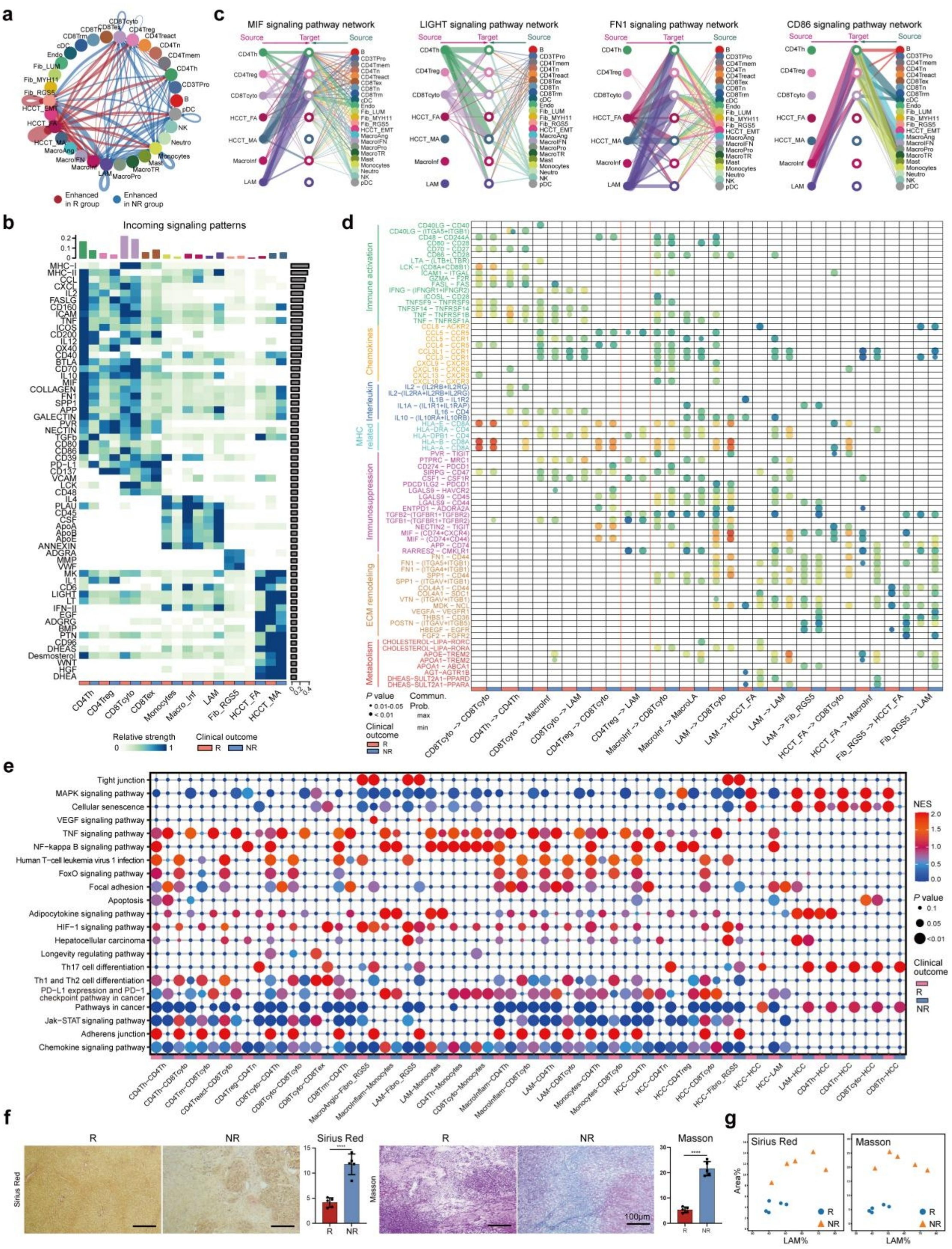

Supplementary Fig. 8 | Additional results related to immune interaction landscape of integrated responder and non-responder communication networks. a Circus plot showing distinct interaction strength between responders and non-responders among TIME components. **b** Hierarchical plots showing the signaling pathways of MIF, FN1, LIGHT and CD86 within interaction network of TIME. **c** Incoming signaling patterns of critical TIME components in responder group and non-responder group, colors represented the values of relative signal strength. **d** Bubble plot showing additional cell-cell communications, affiliating with Fig. 8e. **e** Bubble plot showing KEGG pathway activity analysis of intercellular communications among critical TIME component, calculated by CellCall approach. **f** Representative Sirius red and Masson's trichrome

staining of tumor regions from responders (R) and non-responders (NR) illustrating increased collagen deposition in NR samples (Sirius red, P=6.81×10^{-5} , Masson, P=2.06×10^{-6} . For box plots, the centre line indicates the median, box limits indicate the 25th and 75th percentiles, and whiskers indicate the minimum and maximum values. Statistic significance was determined by two-sided unpaired student's t tests. n=5). **g** Scatter plots showing patient-level distributions of LAM proportions and fibrosis staining metrics in R and NR groups (n=5).

**Supplementary Table 1**	**Clinical baseline and treatment line of patients from our scRNA-seq cohort**
**Supplementary Table 2**	**Clinical information of HCC patients from our study and public datasets**
**Supplementary Table 3**	**Proportion of major celltype in each patients and their expression characteristics**
**Supplementary Table 4**	**CNV quantification of HCC cells from 14 patients of our cohort**
**Supplementary Table 5**	**Features of HCC cells categorized as NMF subsets**
**Supplementary Table 6**	**Metabolism score of HCC cell subsets**
**Supplementary Table 7**	**Expression characteristics of T/NK cell subsets**
**Supplementary Table 8**	**Genes adopted for T cell state scoring**
**Supplementary Table 9**	**Expression characteristic of myeloid cell subsets**
**Supplementary Table 10**	**Correlation matrix of major celltype proportions and clinical outcomes**
**Supplementary Table 11**	**Correlation matrix of T cell subset proportions and clinical outcomes**
**Supplementary Table 12**	**Correlation matrix of myeloid cell subset proportions and clinical outcomes**
**Supplementary Table 13**	**Cross-comparison of neoadjuvant αPD-1 translational studies in HCC**
**Supplementary Table 14**	**Sample-level quality control metrics for single-cell RNA sequencing data**
**Supplementary Table 15**	**Experiment supplies**

Supplementary Table 1. Clinical baseline and treatment line of patients from our scRNA-seq cohort

Sample_ID	Pathology	Resource	Sex	Age	BMI	Hepatic steatosis	Liver CT attenuation value	Spleen CT attenuation value	Baseline tumor stage	Tumor size before treatment (cm)	Tumor size after treatment (cm)
Patient01	HCC	Post_surgery	Female	47	26.67	NO	73	68	T3N0M0	15.4	9.8
Patient02	HCC	Post_surgery	Male	41	29.04	NO	62	42	T3N0M0	9.9	7
Patient03	HCC	Post_surgery	Male	56	22.84	NO	66	Spleen resected	T3N1M0	12.1	10.6
Patient04	HCC	Post_surgery	Male	28	25.14	NO	89	48	T3N0M1	11.5	9
Patient05	HCC	Post_surgery	Male	41	28.69	NO	69	66	T3N0M0	7.1	3.7
Patient06	HCC	Post_surgery	Female	55	23.23	Mild	92	124	T3N0M0	8.1	12.7
Patient07	HCC	Post_surgery	Male	67	24.34	NO	58	48	T3N0M0	6.6	6
Patient08	HCC	Post_surgery	Male	56	20.76	NO	59	42	T3N0M0	14.1	5.1
Patient09	HCC	Post_surgery	Male	59	30.49	NO	90	83	T3N1M0	11.6	10.1
Patient10	HCC	Post_surgery	Male	45	21.26	Mild	88	101	T3N0M0	12.2	6
Patient11	HCC	Post_surgery	Male	65	24.84	NO	58	40	T3N0M0	8.3	7.6
Patient12	HCC	Post_surgery	Male	53	28.96	Mild	97	117	T3N0M0	12	8
Patient13	HCC	Post_surgery	Male	63	24.00	Mild	72	76	T3N0M0	6.1	6.8
Patient14	HCC	Post_surgery	Male	66	24.80	NO	85	79	T3N0M0	13.1	10.7
**Sample_ID**	**High-risk histopathological feature**	**Macrovascular invasion**	**Microvasucular invasion**	**Number of nodules**	**Baseline AFP (ng/ml)**	**Perioperative AFP (ng/ml)**	**PD-1 antibody**	**Dose Schedule**	**Cycles**	**Interval from Last Dose**	**Perioperative Delays**
Patient01	Cancer embolus,Satellite nodule, Poorly differentiated	Portal vein	0	Multiple	36926	314.4	Tislelizumab	200mg q3w	3	24	NO
Patient02	Cancer embolus,Poorly differentiated	Portal vein	1	Single	3.75	2.08	Camrelizumab	200mg q3w	3	41	NO
Patient03	Cancer embolus, Satellite nodule	Portal vein	0	Multiple	21	12.96	Tislelizumab	200mg q3w	5	47	NO
Patient04	No	No	0	Multiple	1093	532.2	Tislelizumab	200mg q3w	5	54	NO
Patient05	Cancer embolus,Poorly differentiated	Portal vein	1	Single	2.19	1.81	Camrelizumab	200mg q3w	3	40	YES
Patient06	No	Portal vein	2	Single	16301	10.3	Tislelizumab	200mg q3w	5	32	NO
Patient07	No	No	0	Multiple	1.63	6.23	Tislelizumab	200mg q3w	5	27	NO
Patient08	Cancer embolus	No	0	Single	340.4	8.33	Tislelizumab	200mg q3w	5	27	NO
Patient09	No	No	0	Single	549	113	Camrelizumab	200mg q3w	4	39	NO
Patient10	No	No	0	Single	24.2	4.7	Camrelizumab	200mg q3w	5	38	NO
Patient11	No	No	0	Multiple	766.5	710.9	Camrelizumab	200mg q3w	5	44	NO
Patient12	No	No	0	Single	1483	75.9	Sintilimab	200mg q3w	5	27	YES
Patient13	Satellite nodule	No	1	Multiple	2.78	2.62	Camrelizumab	200mg q3w	3	34	NO
Patient14	No	No	0	Single	4.44	2.77	Camrelizumab	200mg q3w	3	34	NO
**Sample_ID**	**Reasons for Perioperative delay**	**Treatment period**	**Adverse Events Level**	**Adverse Events**	**Surgical Complications**	**RECIST 1.1**	**Pathologic response**	**Efficacy group**	**Residual tumor (%)**	**RFS (days)**	**OS (days)**
Patient01	/	9w	/	/	NO	PR	MPR	Responder	20%	62	420
Patient02	/	9w	/	/	NO	SD	non-MPR	Non-responder	50%	131	1032
Patient03	/	15w	2	Transaminase Elevation	NO	SD	MPR	Responder	20%	792	992
Patient04	/	15w	2	Proteinuria	NO	SD	MPR	Responder	15%	34	573
Patient05	Hypoproteinemia	9w	3	Hypoproteinemia, Proteinuria	NO	PR	non-MPR	Non-responder	55%	723	800
Patient06	/	15w	2	Hypertension, Dizziness	NO	PD	MPR	Responder	45%	637	880
Patient07	/	15w	2	Hypertension, Gingival Bleeding	NO	SD	CPR	Responder	0	648	782
Patient08	/	15w	3	Hypothyroidism, Fatigue	NO	PR	non-MPR	Non-responder	60%	90	285
Patient09	/	12w	2	Transaminase Elevation	NO	SD	non-MPR	Non-responder	65%	622	730
Patient10	/	15w	3	Hypertension, Dizziness	NO	PR	MPR	Responder	10%	571	876
Patient11	/	15w	2	Loss of Appetite, Nausea	NO	SD	non-MPR	Non-responder	70%	566	724
Patient12	Headache accompanied by right-sided eyelid ptosis, considering neurologic adverse event	15w	3	Headache, Upper Eyelid Ptosis	Wound Oozing	PR	MPR	Responder	10%	601	847
Patient13	/	9w	2	Nausea, Abdominal Discomfort	NO	SD	non-MPR	Non-responder	80%	80	230
Patient14	/	9w	2	Hypertension	NO	SD	CPR	Responder	0	542	590

Supplementary Table 1 | Clinical baseline and treatment line of patients from our scRNA-seq cohort. Baseline demographic, clinical treatment, pathological features and other applicable information of 14 patients from our HCC αPD-1 immunotherapy cohort involved with scRNA-seq. Abbreviations: PR, partial response; SD, stable disease; PD, progressive disease; CPR, complete pathological response; MPR, major pathological response; AFP, alpha fetoprotein; RFS: recurrence free survival; OS, overall survival.

Supplementary Table 2. Clinical information of HCC patients from our study and public datasets

Source	Patient	Sex	Age	Other diseases	Cancer Type	Tissue (Tumor, Normal, Boder)			Treatment information	Usage	Drug	Therapy cycle
GSE149614	HCC01	Male	66	HBV	Hepatocellular Carcinoma	YES	NO	NO	Un-treatment	scRNA	/	/
GSE149614	HCC02	Male	65	HBV	Hepatocellular Carcinoma	YES	NO	NO	Un-treatment	scRNA	/	/
GSE149614	HCC03	Male	66	HCV	Hepatocellular Carcinoma	YES	YES	NO	Un-treatment	scRNA	/	/
GSE149614	HCC04	Male	60	HCV	Hepatocellular Carcinoma	YES	YES	NO	Un-treatment	scRNA	/	/
GSE149614	HCC05	Male	65	None	Hepatocellular Carcinoma	YES	YES	NO	Un-treatment	scRNA	/	/
GSE149614	HCC06	Female	64	HBV	Hepatocellular Carcinoma	YES	YES	NO	Un-treatment	scRNA	/	/
GSE149614	HCC07	Male	48	None	Hepatocellular Carcinoma	YES	YES	NO	Un-treatment	scRNA	/	/
GSE149614	HCC08	Male	64	None	Hepatocellular Carcinoma	YES	YES	NO	Un-treatment	scRNA	/	/
GSE149614	HCC09	Male	48	HBV	Hepatocellular Carcinoma	YES	YES	NO	Un-treatment	scRNA	/	/
GSE149614	HCC10	Male	53	HBV	Hepatocellular Carcinoma	YES	YES	NO	Un-treatment	scRNA	/	/
GSE156625	P1	Male	unknown	HBV	Hepatocellular Carcinoma	YES	YES	NO	Un-treatment	scRNA	/	/
GSE156625	P2	Female	unknown	HBV	Hepatocellular Carcinoma	YES	YES	NO	Un-treatment	scRNA	/	/
GSE156625	P3	Male	unknown	HBV	Hepatocellular Carcinoma	YES	YES	NO	Un-treatment	scRNA	/	/
GSE156625	P4	Male	unknown	HBV	Hepatocellular Carcinoma	YES	YES	NO	Un-treatment	scRNA	/	/
GSE156625	P5	Male	unknown	None	Hepatocellular Carcinoma	YES	YES	NO	Un-treatment	scRNA	/	/
GSE156625	P6	Female	unknown	None	Hepatocellular Carcinoma	YES	YES	NO	Un-treatment	scRNA	/	/
GSE156625	P7	Male	unknown	HBV	Hepatocellular Carcinoma	YES	YES	NO	Un-treatment	scRNA	/	/
GSE156625	P8	Female	unknown	None	Hepatocellular Carcinoma	YES	YES	NO	Un-treatment	scRNA	/	/
GSE156625	P9	Male	unknown	HBV	Hepatocellular Carcinoma	YES	YES	NO	Un-treatment	scRNA	/	/
GSE156625	P10	Male	unknown	HBV	Hepatocellular Carcinoma	YES	YES	NO	Un-treatment	scRNA	/	/
GSE156625	P11	Male	unknown	HBV	Hepatocellular Carcinoma	YES	YES	NO	Un-treatment	scRNA	/	/
GSE156625	P13	Male	unknown	None	Hepatocellular Carcinoma	YES	YES	NO	Un-treatment	scRNA	/	/
GSE156625	P14	Male	unknown	HBV	Hepatocellular Carcinoma	YES	YES	NO	Un-treatment	scRNA	/	/
GSE156625	P15	Male	unknown	None	Hepatocellular Carcinoma	YES	YES	NO	Un-treatment	scRNA	/	/
GSE202642	GSM6127499	Male	66	HBV	Hepatocellular Carcinoma	YES	NO	NO	Un-treatment	scRNA	/	/
GSE202642	GSM6127500	Male	54	HBV	Hepatocellular Carcinoma	YES	NO	NO	Un-treatment	scRNA	/	/
GSE202642	GSM6127501	Male	58	HBV	Hepatocellular Carcinoma	YES	NO	NO	Un-treatment	scRNA	/	/
GSE202642	GSM6127502	Male	64	HBV	Hepatocellular Carcinoma	YES	NO	NO	Un-treatment	scRNA	/	/
GSE202642	GSM6127503	Male	77	HBV	Hepatocellular Carcinoma	YES	NO	NO	Un-treatment	scRNA	/	/
GSE202642	GSM6127504	Male	41	HBV	Hepatocellular Carcinoma	YES	NO	NO	Un-treatment	scRNA	/	/
GSE202642	GSM6127505	Male	54	HBV	Hepatocellular Carcinoma	YES	NO	NO	Un-treatment	scRNA	/	/
GSE189903	Patient1<br>(GSM5709315/<br>GSM5709316/<br>GSM5709317/<br>GSM5709318/<br>GSM5709319)	unknown	unknown	unknown	Hepatocellular Carcinoma	YES	YES	YES	Un-treatment	scRNA	/	/
GSE189903	Patient2<br>(GSM5709320/<br>GSM5709321/<br>GSM5709322/<br>GSM5709336/<br>GSM5709337)	unknown	unknown	unknown	Hepatocellular Carcinoma	YES	YES	YES	Un-treatment	scRNA	/	/
GSE189903	Patient3<br>(GSM5709323/<br>GSM5709324/<br>GSM5709325/<br>GSM5709326/<br>GSM5709327)	unknown	unknown	unknown	Hepatocellular Carcinoma	YES	YES	YES	Un-treatment	scRNA	/	/
GSE189903	Patient4<br>(GSM5709328/<br>GSM5709329/<br>GSM5709330/<br>GSM5709331/<br>GSM5709332)	unknown	unknown	unknown	Hepatocellular Carcinoma	YES	YES	YES	Un-treatment	scRNA	/	/
GSE151530	H08	Male	66	HCV	Hepatocellular Carcinoma	YES	NO	NO	unknown	scRNA	Tremel	unknown
GSE151530	H18	Male	61	HCV	Hepatocellular Carcinoma	YES	NO	NO	unknown	scRNA	Durva/Tremel	unknown
GSE151530	H21	Male	77	None	Hepatocellular Carcinoma	YES	NO	NO	Un-treatment	scRNA	/	/
GSE151530	H23	Female	41	Fatty liver	Hepatocellular Carcinoma	YES	NO	NO	unknown	scRNA	Durva/Tremel	unknown
GSE151530	H28	Female	63	HCV	Hepatocellular Carcinoma	YES	NO	NO	unknown	scRNA	Durva/Tremel	unknown
GSE151530	H30	Male	63	HCV	Hepatocellular Carcinoma	YES	NO	NO	unknown	scRNA	Durva/Tremel	unknown
GSE151530	H34	Male	63	HCV	Hepatocellular Carcinoma	YES	NO	NO	unknown	scRNA	Durva/Tremel	unknown
GSE151530	H37	Male	65	HCV	Hepatocellular Carcinoma	YES	NO	NO	unknown	scRNA	Durva/Tremel	unknown
GSE151530	H38	Male	74	None	Hepatocellular Carcinoma	YES	NO	NO	Un-treatment	scRNA	/	/
GSE151530	H41	Male	61	HCV	Hepatocellular Carcinoma	YES	NO	NO	unknown	scRNA	No treatment	unknown
GSE151530	H43	Female	60	HBV	Hepatocellular Carcinoma	YES	NO	NO	unknown	scRNA	Durva/Tremel	unknown
GSE151530	H49	Male	54	HCV	Hepatocellular Carcinoma	YES	NO	NO	unknown	scRNA	Durva/Tremel	unknown
GSE151530	H54	Male	35	HBV	Hepatocellular Carcinoma	YES	NO	NO	unknown	scRNA	Durva/Tremel	unknown
GSE151530	H55	Female	70	None	Hepatocellular Carcinoma	YES	NO	NO	unknown	scRNA	Durva/Tremel	unknown
GSE151530	H58	Male	73	None	Hepatocellular Carcinoma	YES	NO	NO	unknown	scRNA	Durva/Tremel	unknown
GSE151530	H62	Female	67	HCV	Hepatocellular Carcinoma	YES	NO	NO	Un-treatment	scRNA	/	/
GSE151530	H63	Male	81	HBV	Hepatocellular Carcinoma	YES	NO	NO	unknown	scRNA	Durva/Tremel	unknown
GSE151530	H65	Female	62	HCV	Hepatocellular Carcinoma	YES	NO	NO	unknown	scRNA	Durva/Tremel	unknown
GSE151530	H68	Male	78	HBV	Hepatocellular Carcinoma	YES	NO	NO	unknown	scRNA	Durva/Tremel	unknown
GSE151530	H70	Male	72	None	Hepatocellular Carcinoma	YES	NO	NO	Un-treatment	scRNA	/	/
GSE151530	H72	Male	76	Fatty liver	Hepatocellular Carcinoma	YES	NO	NO	unknown	scRNA	No treatment	unknown
GSE151530	H73	Male	58	HBV,HDV	Hepatocellular Carcinoma	YES	NO	NO	unknown	scRNA	Durva/Tremel	unknown
GSE151530	H74	Male	67	HCV	Hepatocellular Carcinoma	YES	NO	NO	unknown	scRNA	Durva/Tremel	unknown
GSE151530	H75	Female	74	HCV	Hepatocellular Carcinoma	YES	NO	NO	Un-treatment	scRNA	/	/
GSE151530	H77	Male	72	HCV	Hepatocellular Carcinoma	YES	NO	NO	unknown	scRNA	Durva/Tremel	unknown

(See legend on next page)

Source	Patient	Sex	Age	Other diseases	Cancer Type	Tissue (Tumor, Normal, Boder)			Treatment information	Usage	Drug	Therapy cycle
GSE206325	15	Male	41	HBV	Hepatocellular Carcinoma	YES	YES	NO	Non-responder	scRNA	Nivolumab	2-4 times
GSE206325	57	Male	61	HCV+NASH	Hepatocellular Carcinoma	YES	YES	NO	Non-responder	scRNA	Nivolumab	2-4 times
GSE206325	63	Male	63	HCV+NASH	Hepatocellular Carcinoma	YES	YES	NO	Responder	scRNA	Nivolumab	2-4 times
GSE206325	71	Male	29	HBV	Hepatocellular Carcinoma	YES	YES	NO	Non-responder	scRNA	Nivolumab	2-4 times
GSE206325	98	Female	56	HBV	Hepatocellular Carcinoma	YES	YES	NO	Non-responder	scRNA	Nivolumab	2-4 times
GSE206325	104	Male	35	HBV	Hepatocellular Carcinoma	YES	YES	NO	Non-responder	scRNA	Nivolumab	2-4 times
GSE206325	106	Female	66	HCV	Hepatocellular Carcinoma	YES	YES	NO	Responder	scRNA	Nivolumab	2-4 times
GSE206325	121	Male	38	HBV	Hepatocellular Carcinoma	YES	YES	NO	Non-responder	scRNA	Nivolumab	2-4 times
GSE206325	124	Male	65	HCV	Hepatocellular Carcinoma	YES	YES	NO	Non-responder	scRNA	Cemiplimab	two cycles
GSE206325	1001	Male	65	HCV	Hepatocellular Carcinoma	YES	YES	NO	Non-responder	scRNA	Cemiplimab	two cycles
GSE206325	1003	Male	45	HBV	Hepatocellular Carcinoma	YES	YES	NO	Non-responder	scRNA	Cemiplimab	two cycles
GSE206325	1004	Female	76	NASH	Hepatocellular Carcinoma	YES	YES	NO	Non-responder	scRNA	Cemiplimab	two cycles
GSE206325	1005	Female	77	None	Hepatocellular Carcinoma	YES	YES	NO	Non-responder	scRNA	Cemiplimab	two cycles
GSE206325	1006	Male	47	ASH	Hepatocellular Carcinoma	YES	YES	NO	Responder	scRNA	Cemiplimab	two cycles
GSE206325	1012	Male	49	HBV	Hepatocellular Carcinoma	YES	YES	NO	Non-responder	scRNA	Cemiplimab	two cycles
GSE206325	1014	Male	82	HBV	Hepatocellular Carcinoma	YES	YES	NO	Responder	scRNA	Cemiplimab	two cycles
GSE206325	1017	Male	71	HBV	Hepatocellular Carcinoma	YES	YES	NO	Responder	scRNA	Cemiplimab	two cycles
GSE206325	1021	Male	68	HCV	Hepatocellular Carcinoma	YES	YES	NO	Non-responder	scRNA	Cemiplimab	two cycles
GSE206325	1023	Male	63	ASH	Hepatocellular Carcinoma	YES	YES	NO	Responder	scRNA	Cemiplimab	two cycles
GSE206325	1024	Male	45	HBV	Hepatocellular Carcinoma	YES	YES	NO	Non-responder	scRNA	Cemiplimab	two cycles
GSE206325	1025	Male	67	HCV	Hepatocellular Carcinoma	YES	YES	NO	Non-responder	scRNA	Cemiplimab	two cycles
GSE206325	1030	Male	66	HBV	Hepatocellular Carcinoma	YES	YES	NO	Non-responder	scRNA	Cemiplimab	two cycles
GSE206325	1031	Male	73	None	Hepatocellular Carcinoma	YES	YES	NO	Non-responder	scRNA	Cemiplimab	two cycles
GSE206325	1029	Male	68	HBV	Hepatocellular Carcinoma	YES	YES	NO	Non-responder	scRNA	Cemiplimab	two cycles
GSE206325	1032	Male	55	HBV	Hepatocellular Carcinoma	YES	YES	NO	Non-responder	scRNA	Cemiplimab	two cycles
GSE206325	1037	Male	68	HCV	Hepatocellular Carcinoma	YES	YES	NO	Responder	scRNA	Cemiplimab	two cycles
GSE206325	1039	Male	70	NASH	Hepatocellular Carcinoma	YES	YES	NO	Non-responder	scRNA	Cemiplimab	two cycles
GSE206325	1040	Male	77	HCV	Hepatocellular Carcinoma	YES	YES	NO	Non-responder	scRNA	Cemiplimab	two cycles
GSE206325	1041	Male	73	NASH	Hepatocellular Carcinoma	YES	YES	NO	Responder	scRNA	Cemiplimab	two cycles
skrx2fz79n	P1	Male	53	HBV	Hepatocellular Carcinoma	YES	YES	NO	Non-responder	scRNA+ST	αPD-1	unknown
skrx2fz79n	P2	Female	49	HBV	Hepatocellular Carcinoma	YES	YES	NO	Non-responder	scRNA+ST	αPD-1	unknown
skrx2fz79n	P3	Male	46	HBV	Hepatocellular Carcinoma	YES	YES	NO	Non-responder	scRNA+ST	αPD-1	unknown
skrx2fz79n	P4	Female	70	HBV	Hepatocellular Carcinoma	YES	YES	NO	Non-responder	scRNA	αPD-1	unknown
skrx2fz79n	P5	Male	68	HBV	Hepatocellular Carcinoma	YES	YES	NO	Non-responder	scRNA+ST	αPD-1	unknown
skrx2fz79n	P6	Male	48	HBV	Hepatocellular Carcinoma	YES	YES	NO	Non-responder	scRNA	αPD-1	unknown
skrx2fz79n	P7	Male	68	HBV	Hepatocellular Carcinoma	YES	NO	NO	Responder	ST	αPD-1	unknown
skrx2fz79n	P8	Male	65	HBV	Hepatocellular Carcinoma	NO	NO	YES	Non-responder	ST	αPD-1	unknown
skrx2fz79n	P9	Male	41	HBV	Hepatocellular Carcinoma	NO	NO	YES	Responder	ST	αPD-1	unknown
skrx2fz79n	P10	Male	38	HBV	Hepatocellular Carcinoma	YES	NO	NO	Responder	ST	αPD-1	unknown
skrx2fz79n	P11	Female	51	None	Hepatocellular Carcinoma	NO	NO	YES	Non-responder	ST	αPD-1	unknown
in house	Patient01	Female	47	HBV	Hepatocellular Carcinoma	YES	NO	NO	Responder	scRNA	Tislelizumab	3 cycles
in house	Patient02	Male	41	HBV	Hepatocellular Carcinoma	YES	NO	NO	Non-responder	scRNA	Camrelizumab	3 cycles
in house	Patient03	Male	56	HBV	Hepatocellular Carcinoma	YES	NO	NO	Responder	scRNA+ST	Tislelizumab	5 cycles
in house	Patient04	Male	28	HBV	Hepatocellular Carcinoma	YES	NO	NO	Responder	scRNA	Tislelizumab	5 cycles
in house	Patient05	Male	41	HBV	Hepatocellular Carcinoma	YES	NO	NO	Non-responder	scRNA	Camrelizumab	3 cycles
in house	Patient06	Female	55	HBV+Fatty liver	Hepatocellular Carcinoma	YES	NO	NO	Responder	scRNA	Tislelizumab	5 cycles
in house	Patient07	Male	67	HBV	Hepatocellular Carcinoma	YES	NO	NO	Responder	scRNA+ST	Tislelizumab	5 cycles
in house	Patient08	Male	56	HBV	Hepatocellular Carcinoma	YES	NO	NO	Non-responder	scRNA+ST	Tislelizumab	5 cycles
in house	Patient09	Male	59	HBV	Hepatocellular Carcinoma	YES	NO	NO	Non-responder	scRNA	Camrelizumab	4 cycles
in house	Patient10	Male	45	HBV+Fatty liver	Hepatocellular Carcinoma	YES	NO	NO	Responder	scRNA	Camrelizumab	5 cycles
in house	Patient11	Male	65	HBV	Hepatocellular Carcinoma	YES	NO	NO	Non-responder	scRNA+ST	Camrelizumab	5 cycles
in house	Patient12	Male	53	HBV+Fatty liver	Hepatocellular Carcinoma	YES	NO	NO	Responder	scRNA+ST	Sintilimab	5 cycles
in house	Patient13	Male	63	HBV+Fatty liver	Hepatocellular Carcinoma	YES	NO	NO	Non-responder	scRNA+ST	Camrelizumab	3 cycles
in house	Patient14	Male	66	HBV	Hepatocellular Carcinoma	YES	NO	NO	Responder	scRNA	Camrelizumab	3 cycles
in house	Patient15	Male	42	HBV	Hepatocellular Carcinoma	YES	NO	NO	Un-treatment	Organoid	/	/
in house	Patient16	Female	55	HBV	Hepatocellular Carcinoma	YES	NO	NO	Un-treatment	Organoid	/	/
in house	Patient17	Male	48	HBV	Hepatocellular Carcinoma	YES	NO	NO	Un-treatment	Organoid	/	/
in house	Patient18	Female	53	HBV	Hepatocellular Carcinoma	YES	NO	NO	Un-treatment	Organoid	/	/
in house	Patient19	Female	46	HBV	Hepatocellular Carcinoma	YES	NO	NO	Un-treatment	Organoid	/	/
in house	Patient20	Male	58	HBV	Hepatocellular Carcinoma	YES	NO	NO	Un-treatment	Organoid	/	/
in house	Patient21	Male	63	HBV	Hepatocellular Carcinoma	YES	NO	NO	Responder	IHC+Flow cytometry	Camrelizumab	5 cycles
in house	Patient22	Male	58	HBV	Hepatocellular Carcinoma	YES	NO	NO	Responder	IHC+Flow cytometry	Tislelizumab	3 cycles
in house	Patient23	Female	71	HBV+Fatty liver	Hepatocellular Carcinoma	YES	NO	NO	Responder	IHC+Flow cytometry	Tislelizumab	3 cycles
in house	Patient24	Male	52	HBV	Hepatocellular Carcinoma	YES	NO	NO	Responder	IHC+Flow cytometry	Tislelizumab	5 cycles
in house	Patient25	Female	69	HBV	Hepatocellular Carcinoma	YES	NO	NO	Responder	IHC+Flow cytometry	Camrelizumab	3 cycles
in house	Patient26	Male	74	HBV	Hepatocellular Carcinoma	YES	NO	NO	Non-responder	IHC+Flow cytometry	Camrelizumab	5 cycles
in house	Patient27	Male	55	HBV+Fatty liver	Hepatocellular Carcinoma	YES	NO	NO	Non-responder	IHC+Flow cytometry	Tislelizumab	3 cycles
in house	Patient28	Male	67	HBV+Fatty liver	Hepatocellular Carcinoma	YES	NO	NO	Non-responder	IHC+Flow cytometry	Tislelizumab	3 cycles
in house	Patient29	Male	60	HBV	Hepatocellular Carcinoma	YES	NO	NO	Non-responder	IHC+Flow cytometry	Camrelizumab	5 cycles
in house	Patient30	Female	73	HBV	Hepatocellular Carcinoma	YES	NO	NO	Non-responder	IHC+Flow cytometry	Tislelizumab	3 cycles

Supplementary Table 2 | Clinical information of HCC patients from our study and public datasets. Clinical information of HCC patients from our study and from external HCC multi-omics datasets that incorporated into our integrated analysis, including accession numbers of datasets, corresponding patient with basic status, and treatment strategies if applicable.

Supplementary Table 3. Proportion of major celltype in each patients and their expression characteristics

Proportion of major celltype in each patient

Patient	HCC	T cells	NK cells	B cells	Monocytes	Macrophages	Dendritic cells	Neutrophils	Mast cells	Fibroblasts	Endothelial cells
Patient01	0.020481	0.446749	0.016129	0.006656	0.016641	0.072197	0.020482	0.006656	0.000256	0.233999	0.159754
Patient02	0.03633	0.733578	0.054312	0.010275	0.00844	0.12055	0.007157	0.006055	0.000734	0.008257	0.014312
Patient03	0.003596	0.687329	0.022089	0.143836	0.008219	0.039041	0.011643	0.00274	0.000856	0.042466	0.038185
Patient04	0.572042	0.048824	0.008595	0.001432	0.034499	0.163901	0.00979	0.017071	0	0.127611	0.016235
Patient05	0.508791	0.290919	0.019032	0.002538	0.003535	0.159779	0.005799	0.00716	0.000453	0.000816	0.001178
Patient06	0.648058	0.105145	0.009355	0.001525	0.006508	0.192699	0.003968	0.004779	0.000203	0.008033	0.019727
Patient07	0.005195	0.815152	0.048613	0.019406	0.005195	0.037832	0.010781	0.005587	0.00392	0.019798	0.028521
Patient08	0.025542	0.593389	0.071462	0.03221	0.003756	0.060381	0.028361	0.00601	0.008545	0.036623	0.133721
Patient09	0.032859	0.49352	0.08096	0.031946	0.008123	0.168127	0.023823	0.016429	0.000456	0.022636	0.121121
Patient10	0	0.169556	0.007036	0.071907	0.064527	0.375837	0.026599	0.018363	0.002231	0.185344	0.0786
Patient11	0.491556	0.110478	0.011394	0.031841	0.009054	0.183316	0.012005	0.003459	0.000203	0.050763	0.095931
Patient12	0.000847	0.638374	0.095354	0.025611	0.033337	0.083395	0.027835	0.008572	0.001693	0.061276	0.023706
Patient13	0.536226	0.008031	0.005587	0.000786	0.001135	0.434445	0.002268	0.001222	0	0.007594	0.002706
Patient14	0.001663	0.825592	0.040307	0.028151	0.007038	0.059245	0.013691	0.003967	0.002175	0.004607	0.013564

Expression characteristics of each major celltype

Endothelial cells	Fibroblasts	T cells	Macrophages	Dendritic cells	B cells	NK cells	HCC	Neutrophils	Monocytes	Mast cells
PLVAP	TAGLN	CD3E	C1QA	CD1C	CD79A	KLRD1	AGT	S100A12	CD300E	CPA3
RAMP2	COL3A1	CD3D	CD68	CD1D	MS4A1	KLRC1	MGST1	S100A8	FCN1	TPSD1
VWF	PDGFRB	CD2	C1QB	CD1B	CD79B	NCAM1	ALB	FCGR3B	RETN	TPSB2
SOX18	BGN	CD3G	GPNMB	CLEC9A	CD19	KLRF1	AKR1C3	G0S2	NLRP3	GATA2
PTPRB	COL1A2	LTB	CD163	CD1E	BLK	FCGR3A	KRT18	CSF3R	EREG	SVOPL
CD34	SOD3	LCK	C1QC	CLEC10A	IGKC	FGFBP2	AMBP	CD300E	ITGB8	GALC
CLDN5	DCN	IL7R	FOLR2	S100B	IGHM	CX3CR1	ORM1	CXCL8	SLC11A1	GATA1
PODXL	COL6A1	ITK	TYROBP	LAMP3	BANK1	GNLY	KRT8	LILRB2	CLEC5A	ITGA2B
KDR	COL1A1	CD69	TREM2	CSF2RA	IGHG3	CLIC3	GPC3	CMTM2	CCL3L1	MS4A2
FLT1	THBS2	IL2RG	FCER1G	CD300LB	SPIB	GZMB	GSTA1	CLEC4E	VCAN	CTSG
RAMP3	RGS5	CD6	MS4A7	HLA-DQB1	IGHA1	KLRC2	RARRES2	FPR2	CXCL3	KIT
PECAM1	COL6A2	GZMK	LIPA	ITGAX	FCRL1	CD160	MDK	TREM1	SERPINB2	RHEX
EFNB2	TPM2	CD5	CSF1R	IL1R2	MZB1	PRF1	APOB	LILRA5	OLR1	PRG2
CAVIN2	PLAC9	CD96	MRC1	CLEC4A	IGHG1	NKG7	AKR1C1	S100A9	IL1RN	IL1RL1
LDB2	LUM	IL32	FCGR1A	SPINT2	CD22	GZMH	TF	LILRA2	IL1A	CMA1
CDH5	COL6A3	DUSP2	CTSB	HLA-DQA1	IGLC2	XCL2	APCS	FPR1	IL1B	FCER1A
CLEC14A	ACTA2	ICOS	LILRB5	HLA-DQB2	CXCR5	KLRK1	AFP	FCN1	CXCL2	TPSAB1
ADGRL4	MYL9	DUSP4	ABCA1	LGMN	JCHAIN	CD244	APOC1	IL1R2	CCL3	LIF
MMRN2	COL14A1	CD8A	HLA-DRA	LILRB4	CCR7	GZMA	BHMT2	LILRB3	CXCL8	CSF1
CLEC3B	AEBP1	TRBC2	CD209	CLEC12A	CD40	KLRC3	GC	BCL2A1	LYZ	CD82

Supplementary Table 3 | Proportion of major celltype in each patients and their expression characteristics. Proportion of major celltype from 14 patients of our αPD-1 HCC immunotherapy cohort according to scRNA-seq, with the characteristic expression features identified for each major celltype based on differential expression analysis.

Supplementary Table 4. CNV quantification of HCC cells from 14 patients of our coho

	CNV score		CNV score
Patient01	3122.179487	C0	3107.82752
Patient02	2737.193548	C1	2156.167147
Patient03	3164.333333	C2	3901.088124
Patient04	4028.50609	C3	3396.848888
Patient05	3459.52389	C4	3840.489144
Patient06	3628.325181	C5	3456.774084
Patient07	3247.486486	C6	4652.478025
Patient08	3887.371681	C7	3122.136591
Patient09	2711.439216		
Patient11	3316.45807		
Patient12	3062.2		
Patient13	2441.303581		
Patient14	2696.846154		

Supplementary Table 4 | CNV quantification of HCC cells from 14 patients of our cohort. Copy number variation (CNV) scores of identified HCC cells from our scRNA-seq cohort, averaged at the 14-individual level and unsupervised-cluster level.

Supplementary Table 5. Features of HCC cells categorized as NMF subsets			
**Subtype1**	**Subtype2**	**Subtype3**	**Subtype4**
CRP	UBE2C	SLCO1B3	IGFBP7
FUNDC2	CDC20	SOX5	SPARC
S100A13	NUF2	ABCB11	VIM
PLIN2	CDCA3	MYOM1	JUNB
MMP24OS	AURKB	ACSS2	ZFP36
RNASE4	CCNB2	PLIN5	FOS
S100A11	KIFC1	HMGCS1	CYP2E1
PGK1	CDK1	SLC23A2	FABP1
SERPINB1	CDCA8	ITPR2	TMSB4X
TCEAL9	CCNB1	ROBO1	DUSP1
TMEM141	AURKA	FBXO31	INSIG1
GALE	BIRC5	GPAM	APOA1
SLC50A1	PTTG1	MYRIP	JUND
LDHA	PRC1	TSHZ2	A2M
CTSA	TOP2A	MVD	SAT1
ORM2	PCLAF	SLCO1B1	MT-ATP8
RTL8A	MAD2L1	FTX	SCD
ANXA4	RRM2	MSMO1	ALB
DHRS7	MYBL2	PCSK6	ITM2B
EFNA1	CDKN3	CFHR3	CD74
FN3K	TPX2	CYP3A5	IFITM2
GLMP	CENPF	MLXIPL	HLA-E
CTSB	CENPW	ACSS3	TMSB10
GPC3	NUSAP1	INSIG1	ASS1
GRN	CKS2	CYP2E1	B2M
DUSP23	ZWINT	ACSL1	APOC3
GJB1	TYMS	CYP4F3	APOC2
MSRB1	HIST1H4C	ZBTB20	TPT1
CARHSP1	KPNA2	SH3D19	CST3
VEGFB	TK1	NEAT1	JUN
S100A16	CENPM	AQP9	HLA-A
AGT	UBE2S	CFHR5	SERPINA1
S100P	UBE2T	CPS1	MT-CO1
SCPEP1	HMGB2	PLG	RBP4
ORM1	CKS1B	ACACA	ACTB
GBA	SMC4	ALDH1L1	HLA-C
SOD2	STMN1	TTPA	MT-CO3
BEX3	TUBA1C	ACSL3	FGL1
NDUFA1	H2AFX	C9	SPINK1
SELENBP1	H2AFZ	AOX1	MALAT1
NEU1	MIS18A	FASN	IFI27
APOA2	TUBA1B	ELL2	SELENOP
NUPR1	MCM7	ACSL4	EEF1A1
CSTB	LMNB1	ADH4	AMBP
TMEM9	TUBB	MST1	SERPINA3
TCEAL4	TUBB4B	SQLE	MT-CO2
LAGE3	DTYMK	TNRC6A	HP
CYB5R1	CDC25B	SREBF2	CTSD
RRAGD	HMGN2	LEAP2	PFN1
VAT1	DHFR	LDLR	HINT1

Supplementary Table 5 | Features of HCC cells categorized as NMF subsets. Characteristic genes of HCC cell subsets identified by non-negative matrix factorization (NMF).

Supplementary Table 6. Metabolism score of HCC cell subsets				
	Subtype 1	Subtype 2	Subtype 3	Subtype 4
Glycolysis / Gluconeogenesis	0.889137384	0.943818909	0.845499517	0.546538473
Citrate cycle (TCA cycle)	0.883243606	1.044647951	0.666924116	0.284437423
Pentose phosphate pathway	0.736377918	0.883676968	0.394904574	0.271342227
Pentose and glucuronate interconversions	0.573665337	0.635204882	0.509421659	0.151057611
Fructose and mannose metabolism	0.592507287	0.695509869	0.292638131	0.21341381
Galactose metabolism	0.34582246	0.411810775	0.135196963	0.05401529
Ascorbate and aldarate metabolism	0.388803467	0.389838803	0.518693373	0.102441919
Starch and sucrose metabolism	0.099631091	0.130716092	0.074393243	-0.008410407
Amino sugar and nucleotide sugar metabolism	0.419765652	0.437554076	0.1556273	0.051939872
Pyruvate metabolism	0.710122837	0.794135755	0.755349253	0.306907312
Glyoxylate and dicarboxylate metabolism	0.905706521	0.954527551	1.036633958	0.41171388
Propanoate metabolism	0.505616208	0.497096772	0.731554999	0.258629483
Butanoate metabolism	0.41280218	0.340116311	0.784552903	0.24981715
Inositol phosphate metabolism	-0.024772556	-0.039919852	0.036117707	0.014837112
Oxidative phosphorylation	2.04743571	2.324840217	0.952975366	0.947173415
Nitrogen metabolism	0.345073644	0.265977546	1.175807334	0.416243227
Sulfur metabolism	0.56533975	0.498812767	0.44641512	0.241919167
Fatty acid biosynthesis	0.181461417	0.084598271	1.366964636	0.410307195
Fatty acid elongation	0.328525796	0.383900135	0.321921089	0.16429865
Fatty acid degradation	0.65821031	0.594710961	1.320321247	0.548954727
Synthesis and degradation of ketone bodies	0.542885947	0.453883674	1.191112284	0.524650832
Steroid biosynthesis	0.276526965	0.183159372	1.215272173	0.422933753
Primary bile acid biosynthesis	0.416983415	0.288272407	1.051600762	0.249528465
Steroid hormone biosynthesis	0.324972368	0.249930143	0.703377328	0.227911737
Glycerolipid metabolism	0.220239173	0.221017568	0.36448171	0.116428862
Glycerophospholipid metabolism	0.053096422	0.019736962	0.188561324	0.04056497
Ether lipid metabolism	0.037779764	0.007341032	0.117760638	0.056570592
Sphingolipid metabolism	0.107969139	0.050263305	0.094073138	0.054159766
Arachidonic acid metabolism	0.375482563	0.361482083	0.604917608	0.265316899
Linoleic acid metabolism	0.135507003	0.015302591	0.847082881	0.352177071
alpha-Linolenic acid metabolism	0.100861617	0.039245025	0.367205922	0.072673685
Biosynthesis of unsaturated fatty acids	0.390182189	0.364257485	0.891961739	0.363167851
Purine metabolism	0.219354075	0.32264749	0.064839968	0.058436854
Pyrimidine metabolism	0.332794025	0.511130228	0.156192789	0.093072604
Alanine, aspartate and glutamate metabolism	0.390517575	0.327343205	0.924332599	0.375134434
Glycine, serine and threonine metabolism	0.483018453	0.601357031	0.659833379	0.200075907
Cysteine and methionine metabolism	0.594981604	0.692096645	0.652231111	0.248036937
Valine, leucine and isoleucine degradation	0.548414266	0.530589424	0.816609693	0.295502318
Lysine degradation	0.198597303	0.213751928	0.348642665	0.115696063
Arginine biosynthesis	0.634665456	0.529596888	1.648401207	0.691670741
Arginine and proline metabolism	0.430806012	0.498553043	0.517347148	0.21070054
Histidine metabolism	0.299214021	0.310053679	0.627240029	0.131465969
Tyrosine metabolism	0.694740888	0.61206367	1.314541503	0.592377474
Phenylalanine metabolism	0.728592192	0.779841999	1.101134271	0.438805187
Tryptophan metabolism	0.383113142	0.377002631	0.63933431	0.170821874
Phenylalanine, tyrosine and tryptophan biosynthesis	0.64594332	0.774061437	1.687535846	0.424729554
beta-Alanine metabolism	0.425573402	0.440236035	0.680516187	0.181277813
Taurine and hypotaurine metabolism	0.091047613	-0.023530285	0.524699367	0.066594314
Phosphonate and phosphinate metabolism	0.24618791	0.320535032	0.452994557	0.037758109
Selenocompound metabolism	0.29256059	0.316221141	0.321824557	0.058014049
D-Glutamine and D-glutamate metabolism	0.185681126	0.144062402	0.912773611	0.426034306
Glutathione metabolism	0.832567235	1.006715954	0.542836617	0.385912572
N-Glycan biosynthesis	0.320616896	0.301237485	0.234516041	0.111025959
Mucin type O-glycan biosynthesis	-0.158075122	-0.195092493	-0.100023952	-0.093746135
Mannose type O-glycan biosynthesis	-0.136114266	-0.15861173	-0.07367026	-0.093494574
Other types of O-glycan biosynthesis	-0.061549261	-0.104902205	0.077087381	-0.00609602
Glycosaminoglycan biosynthesis - chondroitin sulfate / dermatan sulfate	-0.110166157	-0.147069836	-0.113928293	-0.026884556
Glycosaminoglycan biosynthesis - heparan sulfate / heparin	-0.11779854	-0.156462075	-0.003949668	-0.062383288
Glycosaminoglycan biosynthesis - keratan sulfate	-0.100212862	-0.125203758	0.001832107	-0.026789319
Glycosaminoglycan degradation	0.186288106	0.144150552	0.130783244	0.053704911
Glycosylphosphatidylinositol (GPI)-anchor biosynthesis	0.078545217	0.097061576	-0.031231408	-0.052478632
Glycosphingolipid biosynthesis - lacto and neolacto series	-0.125296615	-0.150201104	-0.115322125	-0.08423596
Glycosphingolipid biosynthesis - globo and isoglobo series	0.059560321	0.045965518	0.012624837	-0.027782397
Glycosphingolipid biosynthesis - ganglio series	0.068598158	0.017280219	0.058190549	0.00169086
Other glycan degradation	0.384129335	0.324874588	0.188926241	0.072559986
Thiamine metabolism	0.398445799	0.452824277	0.09455519	0.025572475
Riboflavin metabolism	0.674539131	0.69525583	0.28941237	0.165195197
Vitamin B6 metabolism	0.191485773	0.222478299	0.826404106	0.148715534
Nicotinate and nicotinamide metabolism	0.211203602	0.151604504	0.461450122	0.097894662
Pantothenate and CoA biosynthesis	0.145271509	0.153928729	0.391697081	0.030821543
Folate biosynthesis	0.908136512	1.200013925	0.654565244	0.248134603
One carbon pool by folate	0.327070318	0.648348091	0.543916285	0.124443252
Retinol metabolism	0.231050235	0.14391421	0.762227766	0.19806722
Porphyrin and chlorophyll metabolism	0.300716799	0.25862344	0.42854959	0.13043276
Ubiquinone and other terpenoid-quinone biosynthesis	0.48566589	0.629175804	0.394460627	0.159357384
Terpenoid backbone biosynthesis	0.376202918	0.358800236	0.961118561	0.393704888
Caffeine metabolism	-0.247727022	-0.300871318	-0.104043171	-0.152644035
Neomycin, kanamycin and gentamicin biosynthesis	-0.069913153	-0.075387257	-0.217905411	-0.098254869
Metabolism of xenobiotics by cytochrome P450	0.715161722	0.660047108	1.147548556	0.527104087
Drug metabolism - cytochrome P450	0.527580773	0.460170313	1.085559308	0.454241356
Drug metabolism - other enzymes	0.603566281	0.711345143	0.581404093	0.330953362

Supplementary Table 6 | Metabolism score of HCC cell subsets. Metabolic activity scores of major metabolic pathways across four HCC cell subsets derived from NMF, metabolism scores were quantified by R package scMetabolism.

Supplementary Table 7. Expression characteristics of T/NK cell subsets

$CD3^+$ Tprolif	$CD4^+$ Thelper	$CD4^+$ Tmemory	$CD4^+$ Tnaive	$CD4^+$ Treact	$CD4^+$ Treg	$CD8^+$ Tcyto	$CD8^+$ Tex	$CD8^+$ Tnaive	$CD8^+$ Trm	NK1	NK2
TYMS	TNF	GNB2L1	TIMP1	GNG4	FOXP3	TNFSF9	VCAM1	LEF1	CCL3L3	GNLY	FCER1G
UBE2C	CCL20	ATP5L	LTB	CD200	IL1R2	ZNF683	ETV1	ARMH1	TMEM2	FGFBP2	B3GNT7
MKI67	SNHG6	GLTSCR2	AQP3	CXCL13	CCR8	KLRK1	LAG3	CCR7	LINC00152	GZMB	KLRC1
RRM2	NR4A1	ATP5E	PVT1	IGFL2	RTKN2	GZMK	CXCL13	CHI3L2	ATP5E	FCGR3A	IRF8
TOP2A	IL4I1	ATP5G2	TRADD	LHFPL6	TNFRSF18	CCL5	DRAIC	LINC02446	FYB	MYOM2	CD160
BIRC5	CDKN1A	USMG5	FDXR	NMB	IL2RA	DUSP2	TNFRSF9	AIF1	FAM46C	CX3CR1	TYROBP
TK1	GAS5	ATP5H	DDB2	LINC01281	CTLA4	OASL	PDCD1	AES	ATP5L	SPON2	BCO2
ZWINT	GRASP	FYB	MAL	GEM	LAYN	PLAAT4	MYO1E	SELL	HN1	PRSS23	LAT2
AURKB	SNHG16	ATP5O	FLT3LG	CPM	TBC1D4	YBX3	HMOX1	PCBP2	GNB2L1	GZMH	KLRF1
TPX2	EEF1G	SELK	BAX	CHN1	TNFRSF4	IER5L	FABP5	LRRN3	TRGC2	PTGDS	KLRK1
GTSE1	BAG3	ATP5I	BBC3	PTPN13	LAIR2	UBE2S	PTMS	MAL	SELK	ADGRG1	ADGRG3
CDK1	SNHG29	SNHG25	PHPT1	BHLHE40-AS1	FANK1	CRTAM	MS4A6A	RACK1	TCEB2	PRF1	NCAM1
ASPM	EGR1	TCEB2	CTSH	BTLA	LINC01943	CD81	GEM	SPINT2	ATPIF1	S1PR5	HIP1
UHRF1	DSE	FAM65B	NOP53	ICA1	IL1R1	CCL3L1	TNFSF9	SEPT6	SHFM1	PLEK	TLE1
CCNA2	SNHG5	CCDC109B	LGALS3	DRAIC	ICA1	CCL4L2	HLA-DQA1	FLT3LG	ATP5G2	TTC38	XCL1
ASF1B	FOSB	AIM1	FXYD5	SMCO4	CLEC7A	CEMIP2	DUSP4	ACTN1	WHSC1L1	NKG7	CCL3
CDT1	ERN1	MYEOV2	TNFSF8	PDE7B	BATF	CD8B	HLA-DRB5	RGS10	ATP5B	KLRD1	SH2D1B
PCLAF	TNFAIP3	ATP5J	GSTK1	TSHZ2	IKZF2	IFNG	LYST	ATP5MC2	USMG5	AKR1C3	LDB2
PKMYT1	UBE2S	ATP5D	RACK1	KIAA1324	HACD1	HLA-DRB1	CRTAM	LTB	HLA-DQA2	ITGAM	CMC1
CCNB2	DUSP5	ATP5J2	ATP5MC2	LINC01480	GK	RESF1	MIR155HG	NOSIP	ATP5J2	HOPX	NCR3
DLGAP5	GPR183	GPX1	LAPTM5	MS4A6A	NGFRAP1	KLRC4	CCL4L2	SELENOH	ATP5I	TYROBP	SYK
SPC25	JUND	LINC00493	ZMAT3	TIAM1	TIGIT	TRGV10	HAVCR2	ABLIM1	RGS1	FGR	XCL2
CLSPN	JUN	ATP5G3	AES	FAAH2	STAM	PTPRCAP	SNAP47	IFITM1	ATP5J	GK5	AREG
CENPF	LINC01578	SELM	ITM2B	ZBED2	LAPTM4B	ARL4D	AKAP5	CD8B	GLTSCR2	KLRF1	SLFN13
CDC20	IER5	HBB	EMP3	DUSP4	ENTPD1	CYTOR	NAB1	MCUB	IGKC	ZEB2	NCR1
CDCA8	CD40LG	45915	VIM	CCDC50	F5	MCOLN2	CD70	SEPT1	ATP5G3	MTSS1	SPRY2
KIAA0101	LMNA	ATP5A1	APOBEC3H	ETV7	CRADD	DTHD1	HLA-DRB1	ATP5MG	ATP5D	FCRL6	MCTP2
GINS2	DUSP1	HBA2	LDHB	G0S2	IKZF4	CD8A	TOX	NOP53	ATP5C1	CEP78	IL2RB
KIFC1	PPP1R15A	SELT	PLP2	CTLA4	COL9A2	SEPTIN7	CLECL1	LIMS2	MYEOV2	CD300A	KLRB1
CDCA3	NEU1	SHFM1	ATP5MG	SGPP2	CARD16	CST7	HLA-DMA	TCF7	SELT	CST7	PLCG2

Supplementary Table 7 | Expression characteristics of T/NK cell subsets. Gene expression features of 12 T/NK cell subsets.

Supplementary Table 8. Genes adopted for T cell state scoring		
$CD8^+$ T Cytotoxic	$CD4^+$ T Effector	T Exhaustion
GZMB	LTA	CXCL13
GZMK	TNF	ENTPD1
TIA1	IL2	BATF
PRF1	IL18	LYST
GNLY	TBX21	TIGIT
FASLG	LTBR	LAG3
SLAMF7	CXCR3	HAVCR2
CD69	IFNG	PDCD1
TNF	TNFSF11	PRDM1
DUSP8	IFNGR1	RBPJ
CCR7	STAT1	CCL4
NFKB1	STAT4	NFATC1
	CCR4	TNFRSF4
	CCR6	CXCR6
	PARP8	HLA-DPB1
	PVRIG	HLA-DPA1
	SLC4A10	IRF9
		UBXN1
		PSMB9
		LCP2
		EWSR1

Supplementary Table 8 | Genes adopted for T cell state scoring. Genesets used for T cell functional scoring, including cytotoxicity, effector, and exhaustion signatures.

Supplementary Table 9. Expression characteristic of myeloid cell subsets												
**MacroAngio**	**MacroIFN**	**MacroInflam**	**LAM**	**MacroProlif**	**MacroTR**	**cDC1**	**cDC2**	**cDC3**	**pDC**	**Monocytes**	**Neutrophils**	**Mast**
ACP5	CXCL10	CXCL1	APOC2	MKI67	VCAM1	CLEC9A	CD1C	LAMP3	GZMB	SERPINB2	S100A8	CPA3
RAB42	CXCL11	CCL3L1	RBP4	TK1	CXCL12	CLNK	CD1E	CCL19	SCT	EREG	CMTM2	TPSB2
CD9	CXCL9	CCL4	FGG	RRM2	CD163L1	XCR1	FCER1A	CCL22	JCHAIN	VCAN	S100A12	TPSAB1
TREM2	GBP5	CXCL3	APOH	GTSE1	CD163	TACSTD2	CLEC10A	NCCRP1	PTPRS	OLR1	FCGR3B	TPSD1
PLA2G7	GBP1	CCL3	FGB	AURKB	SDC3	IDO1	CD207	HMSD	LRRC26	CD300E	FPR2	HDC
TM4SF19	GBP4	CXCL2	APOC3	UBE2C	NAIP	HLA-DOB	CD1B	BCL2L14	LILRA4	CCL20	CDA	IL1RL1
FABP5	HAPLN3	CCL4L2	AMBP	TYMS	SLCO2B1	BTLA	GPAT3	SYNPO2	NIBAN3	RETN	SMIM25	KIT
LPL	ANKRD22	IER3	APOA2	CENPM	CETP	PPY	ENHO	CD200	CLEC4C	FCN1	CD55	GATA2
RARRES1	TNFSF10	ICAM4	FGA	PKMYT1	MERTK	ASB2	CLEC4F	ACHE	TCL1A	INHBA	FCN1	CTSG
SPP1	APOL3	PLAU	VTN	CDK1	CD5L	KIAA0226L	CD1A	CCL17	MZB1	SEMA6B	S100A9	SLC18A2
FABP4	CD2	CXCL8	FABP1	PCLAF	FOLR2	WDFY4	PKIB	LIMCH1	RGS7	MCEMP1	PROK2	MS4A2
CD68	CCL8	FOSB	LCN2	CENPF	C1QA	DBN1	CFP	LAD1	CLIC3	IL1B	S100P	SLC24A3
MPP1	NR1H3	MAFF	HP	CEP55	MS4A6A	DNASE1L3	JAML	IDO1	SLC32A1	ITGB8	FPR1	CMA1
GPNMB	LAP3	MIR155HG	SPP1	TOP2A	CTSC	ENPP1	LGALS2	IL21R	ITM2C	PID1	CXCR2	RGS13
CD59	ETV7	IL1B	CES1	BIRC5	C1QB	FKBP1B	NDRG2	ENOX1	PLXNA4	CXCL3	MCEMP1	VWA5A
MATK	IFIT2	EGR1	C1QA	CDC20	GPR34	PLCD1	FCGR2B	GPR157	FAM129C	NLRP3	RIPOR2	ADCYAP1
MMP12	CALHM6	NR4A2	APOB	ZWINT	MS4A7	CPNE3	CD1D	LY75	DERL3	TREM1	CSF3R	RAB27B
LGALS3	IL15RA	CD83	ORM1	PLK1	SLC40A1	CADM1	CCND2	IL7R	PACSIN1	IL1RN	LILRA5	RHEX
CTSL	WARS	IL1A	APOA1	TPX2	MARCO	GCSAM	CEACAM4	TMCC2	SMPD3	SNAI1	MME	PRG2
CAPG	TAP1	PDPN	C1QB	HMMR	MS4A4A	BIK	UTF1	NLGN4Y	IRF4	PTGS2	G0S2	ENPP3
GM2A	PLAAT4	NRP2	RARRES2	CDKN3	LILRB5	NEGR1	PLD4	NTN1	MAP1A	PTGER2	IFITM2	SIGLEC6
PLD3	SLAMF7	NFKBIA	SAA1	CDCA3	FYB1	PPM1J	PPA1	MREG	LAMP5	ACKR3	TREM1	GCSAML
CTSD	CD38	ARL4C	TF	ANLN	MRC1	FCRL6	AREG	GPR153	PTCRA	FCAR	CPD	CALB2
OTOA	IL18BP	LHFPL2	APCS	CCNA2	DAB2	ZNF366	CST7	BIRC3	PHEX	CD44	CLEC4E	SVOPL
DHRS9	IFIT3	FCGR2A	AKR1C1	NUSAP1	WWP1	BATF3	SULF2	IDO2	IGKC	DNAAF1	BCL2A1	GATA1
CHCHD6	CD40	PLTP	GPX2	PIMREG	FCGR3A	GPR31	NRARP	FSCN1	ZFAT	HBEGF	CFP	HPGD
CSTB	CD7	PDGFB	MT1B	NCAPH	CPM	CCSER1	GHRL	CCR7	SHD	CXCL2	FCAR	CDK15
MMP9	STAT1	WTAP	TREM2	CCNB2	SIGLEC1	NET1	IL1R2	GCSAM	IGHM	EHD1	LST1	ITGA2B
FAM20C	RSAD2	DUSP2	MT1G	CDCA8	SELENOP	FBXO27	CIITA	POGLUT1	IL3RA	CLEC5A	TGM2	MLPH
CCL18	IL4I1	TNFAIP3	MDK	SHCBP1	MAF	S100B	HLA-DQB2	DAPP1	GPM6B	PPIF	MX2	GRAP2

Supplementary Table 9 | Expression characteristic of myeloid cell subsets. Differentially expressed genes of myeloid cell subsets.

Supplementary Table 10. Correlation matrix of major celltype proportions and clinical outcomes												
**Spearman correlation**												
	Clinical	HCC	T	NK	B	Monocytes	Macro	DC	Neutrophils	Mast	Fibro	Endo
Clinical	1	-0.6130133	0.50496417	0.12789486	0.13451011	0.40132524	-0.5181947	0.24696938	0.27563547	0.49281798	0.22491854	-0.0661525
HCC	-0.6130133	1	-0.6527473	-0.4021978	-0.7054945	-0.3626374	0.49010989	-0.7230769	-0.1736264	-0.7841429	-0.3538462	-0.3406593
T	0.50496417	-0.6527473	1	0.76263736	0.48131868	-0.0505495	-0.8373626	0.36263736	-0.0857143	0.75991374	-0.1604396	0.07252747
NK	0.12789486	-0.4021978	0.76263736	1	0.41098901	-0.0769231	-0.6131868	0.50769231	0.14725275	0.5903098	-0.0857143	0.23076923
B	0.13451011	-0.7054945	0.48131868	0.41098901	1	0.24835165	-0.3230769	0.72747253	0.04175824	0.68942899	0.31428571	0.58681319
Monocytes	0.40132524	-0.3626374	-0.0505495	-0.0769231	0.24835165	1	0.09450549	0.42857143	0.55164835	-0.0264318	0.82417582	0.35384615
Macro	-0.5181947	0.49010989	-0.8373626	-0.6131868	-0.3230769	0.09450549	1	-0.2967033	0.15604396	-0.6057283	0.0021978	-0.1604396
DC	0.24696938	-0.7230769	0.36263736	0.50769231	0.72747253	0.42857143	-0.2967033	1	0.4021978	0.63216012	0.57802198	0.70989011
Neutrophils	0.27563547	-0.1736264	-0.0857143	0.14725275	0.04175824	0.55164835	0.15604396	0.4021978	1	0.13436156	0.45054945	0.17802198
Mast	0.49281798	-0.7841429	0.75991374	0.5903098	0.68942899	-0.0264318	-0.6057283	0.63216012	0.13436156	1	0.03524238	0.28414166
Fibro	0.22491854	-0.3538462	-0.1604396	-0.0857143	0.31428571	0.82417582	0.0021978	0.57802198	0.45054945	0.03524238	1	0.7010989
Endo	-0.0661525	-0.3406593	0.07252747	0.23076923	0.58681319	0.35384615	-0.1604396	0.70989011	0.17802198	0.28414166	0.7010989	1
**Spearman significance**												
	Clinical	HCC	T	NK	B	Monocytes	Macro	DC	Neutrophils	Mast	Fibro	Endo
Clinical		0.01109355	0.03384742	0.33056011	0.32223666	0.07752761	0.02999386	0.19577264	0.16867525	0.03750308	0.21814336	0.41122574
HCC	0.01109355		0.00679007	0.07765515	0.00313247	0.10157006	0.03895855	0.00233817	0.27628468	0.00069388	0.10747303	0.11673783
T	0.03384742	0.00679007		0.00111541	0.04200937	0.43379747	0.00017656	0.10157006	0.38660402	0.00116851	0.29191487	0.40416061
NK	0.33056011	0.07765515	0.00111541		0.07291442	0.39827541	0.01118524	0.0333441	0.30790081	0.01440255	0.38660402	0.21326147
B	0.32223666	0.00313247	0.04200937	0.07291442		0.19554334	0.12987157	0.00216608	0.44575518	0.00398999	0.13677137	0.01513048
Monocytes	0.07752761	0.10157006	0.43379747	0.39827541	0.19554334		0.37500362	0.06404589	0.02192233	0.46516578	0.00025862	0.10747303
Macro	0.02999386	0.03895855	0.00017656	0.01118524	0.12987157	0.37500362		0.15123798	0.29720291	0.01209607	0.5	0.29191487
DC	0.19577264	0.00233817	0.10157006	0.0333441	0.00216608	0.06404589	0.15123798		0.07765515	0.00878715	0.01665497	0.00291682
Neutrophils	0.16867525	0.27628468	0.38660402	0.30790081	0.44575518	0.02192233	0.29720291	0.07765515		0.32245286	0.05405998	0.27116377
Mast	0.03750308	0.00069388	0.00116851	0.01440255	0.00398999	0.46516578	0.01209607	0.00878715	0.32245286		0.45311138	0.1610957
Fibro	0.21814336	0.10747303	0.29191487	0.38660402	0.13677137	0.00025862	0.5	0.01665497	0.05405998	0.45311138		0.00336005
Endo	0.41122574	0.11673783	0.40416061	0.21326147	0.01513048	0.10747303	0.29191487	0.00291682	0.27116377	0.1610957	0.00336005	

Supplementary Table 10 | Correlation matrix of major celltype proportions and clinical outcomes. Spearman correlation coefficients and corresponding significance values among major celltype proportions and clinical outcomes from 14 patients of our HCC immunotherapy cohort.

Supplementary Table 11. Correlation matrix of T cell subset proportions and clinical outcomes

Spearman correlation

	Clinical	$CD4^+$ Thelper	$CD4^+$ Tmemory	$CD4^+$ Tnaive	$CD4^+$ Treact	$CD4^+$ Treg	$CD8^+$ Tcyto	$CD8^+$ Tex	$CD8^+$ Tnaive	$CD8^+$ Trm
Clinical	1	0.699011541	-0.652704783	0.418965908	0.037486423	-0.500554006	0.504964173	-0.037486423	-0.059537261	-0.299117727
$CD4^+$ Thelper	0.699011541	1	-0.815384615	0.775824176	-0.226373626	-0.815384615	0.410989011	-0.248351648	0.318681319	-0.512651575
$CD4^+$ Tmemory	-0.652704783	-0.815384615	1	-0.683516484	0.112087912	0.749450549	-0.52967033	0.120879121	-0.481318681	0.730473489
$CD4^+$ Tnaive	0.418965908	0.775824176	-0.683516484	1	-0.287912088	-0.907692308	0.261538462	-0.331868132	0.687912088	-0.435643828
$CD4^+$ Treact	0.037486423	-0.226373626	0.112087912	-0.287912088	1	0.494505495	0.235164835	0.841758242	-0.432967033	0.391639401
$CD4^+$ Treg	-0.500554006	-0.815384615	0.749450549	-0.907692308	0.494505495	1	-0.287912088	0.428571429	-0.595604396	0.508251133
$CD8^+$ Tcyto	0.504964173	0.410989011	-0.52967033	0.261538462	0.235164835	-0.287912088	1	0.481318681	0.059340659	-0.11441151
$CD8^+$ Tex	-0.037486423	-0.248351648	0.120879121	-0.331868132	0.841758242	0.428571429	0.481318681	1	-0.485714286	0.517052018
$CD8^+$ Tnaive	-0.059537261	0.318681319	-0.481318681	0.687912088	-0.432967033	-0.595604396	0.059340659	-0.485714286	1	-0.653465742
$CD8^+$ Trm	-0.299117727	-0.512651575	0.730473489	-0.435643828	0.391639401	0.508251133	-0.11441151	0.517052018	-0.653465742	1

Spearman significance

	Clinical	$CD4^+$ Thelper	$CD4^+$ Tmemory	$CD4^+$ Tnaive	$CD4^+$ Treact	$CD4^+$ Treg	$CD8^+$ Tcyto	$CD8^+$ Tex	$CD8^+$ Tnaive	$CD8^+$ Trm
Clinical		0.003427307	0.006721182	0.068241048	0.450059374	0.035210533	0.033847419	0.450059374	0.420109492	0.147786136
$CD4^+$ Thelper	0.003427307		0.000328374	0.000845856	0.217815137	0.000328374	0.072914422	0.195543345	0.133296104	0.031540401
$CD4^+$ Tmemory	0.006721182	0.000328374		0.00439936	0.352140096	0.001447794	0.027191243	0.340884061	0.042009368	0.002034484
$CD4^+$ Tnaive	0.068241048	0.000845856	0.00439936		0.15879556	1.11356E-05	0.182808429	0.12319721	0.004119288	0.060245397
$CD4^+$ Treact	0.450059374	0.217815137	0.352140096	0.15879556		0.037495101	0.208745631	0.000154402	0.061954014	0.083024627
$CD4^+$ Treg	0.035210533	0.000328374	0.001447794	1.11356E-05	0.037495101		0.15879556	0.064045885	0.013715131	0.03283204
$CD8^+$ Tcyto	0.033847419	0.072914422	0.027191243	0.182808429	0.208745631	0.15879556		0.042009368	0.421890554	0.347779334
$CD8^+$ Tex	0.450059374	0.195543345	0.340884061	0.12319721	0.000154402	0.064045885	0.042009368		0.04046395	0.030287662
$CD8^+$ Tnaive	0.420109492	0.133296104	0.042009368	0.004119288	0.061954014	0.013715131	0.421890554	0.04046395		0.006635565
$CD8^+$ Trm	0.147786136	0.031540401	0.002034484	0.060245397	0.083024627	0.03283204	0.347779334	0.030287662	0.006635565	

Supplementary Table 11 | Correlation matrix of T cell subset proportions and clinical outcomes. Spearman correlation coefficients and corresponding significance values among T cell subsets proportions and clinical outcomes from 14 patients of our HCC immunotherapy cohort.

Supplementary Table 12. Correlation matrix of myeloid cell subset proportions and clinical outcomes												
**Spearman correlation**												
	Clinical	MacroAngio	MacroIFN	MacroInflam	LAM	MacroProlif	MacroTR	cDC1	cDC2	cDC3	pDC	Monocytes
Clinical	1	0.359428647	0.055127093	0.595372606	-0.551270932	-0.03528134	0.218303289	0.257994796	0.282250717	0.187432117	0.651215715	0.712242044
MacroAngio	0.359428647	1	0.112087912	0.771428571	-0.608791209	0.164835165	-0.002197802	0.221978022	0.265934066	0.437362637	0.193619479	0.56043956
MacroIFN	0.055127093	0.112087912	1	-0.125274725	-0.12967033	0.635164835	0.692307692	-0.147252747	0.024175824	0.01978022	0.006600664	-0.213186813
MacroInflam	0.595372606	0.771428571	-0.125274725	1	-0.70989011	0.054945055	0.068131868	0.305494505	0.221978022	0.358241758	0.349835195	0.648351648
LAM	-0.551270932	-0.608791209	-0.12967033	-0.70989011	1	-0.182417582	-0.314285714	-0.608791209	-0.701098901	-0.617582418	-0.668867291	-0.6
MacroProlif	-0.03528134	0.164835165	0.635164835	0.054945055	-0.182417582	1	0.353846154	-0.16043956	0.063736264	-0.010989011	-0.233223463	-0.107692308
MacroTR	0.218303289	-0.002197802	0.692307692	0.068131868	-0.314285714	0.353846154	1	0.279120879	0.16043956	0.353846154	0.198019922	-0.173626374
cDC1	0.257994796	0.221978022	-0.147252747	0.305494505	-0.608791209	-0.16043956	0.279120879	1	0.810989011	0.863736264	0.613861758	0.437362637
cDC2	0.282250717	0.265934066	0.024175824	0.221978022	-0.701098901	0.063736264	0.16043956	0.810989011	1	0.802197802	0.717272161	0.520879121
cDC3	0.187432117	0.437362637	0.01978022	0.358241758	-0.617582418	-0.010989011	0.353846154	0.863736264	0.802197802	1	0.578658216	0.340659341
pDC	0.651215715	0.193619479	0.006600664	0.349835195	-0.668867291	-0.233223463	0.198019922	0.613861758	0.717272161	0.578658216	1	0.58525888
Monocytes	0.712242044	0.56043956	-0.213186813	0.648351648	-0.6	-0.107692308	-0.173626374	0.437362637	0.520879121	0.340659341	0.58525888	1
**Spearman significance**												
	Clinical	MacroAngio	MacroIFN	MacroInflam	LAM	MacroProlif	MacroTR	cDC1	cDC2	cDC3	pDC	Monocytes
Clinical		0.102931301	0.426060832	0.013613347	0.021793156	0.453093568	0.225080047	0.185072733	0.162775374	0.25900614	0.006863377	0.002768655
MacroAngio	0.102931301		0.352140096	0.000929236	0.011781846	0.286659269	0.5	0.222423056	0.178674174	0.059908894	0.252109613	0.020045601
MacroIFN	0.426060832	0.352140096		0.335306371	0.329745042	0.008539819	0.003852505	0.307900812	0.469801071	0.475842198	0.492342376	0.231790737
MacroInflam	0.013613347	0.000929236	0.335306371		0.002916825	0.427838013	0.410051821	0.143890095	0.222423056	0.104490658	0.109381068	0.007199084
LAM	0.021793156	0.011781846	0.329745042	0.002916825		0.266070827	0.136771373	0.011781846	0.003360055	0.010612528	0.005389833	0.013045948
MacroProlif	0.453093568	0.286659269	0.008539819	0.427838013	0.266070827		0.107473027	0.291914869	0.415960426	0.487910563	0.209623288	0.357817863
MacroTR	0.225080047	0.5	0.003852505	0.410051821	0.136771373	0.107473027		0.16658526	0.291914869	0.107473027	0.247196229	0.276284682
cDC1	0.185072733	0.222423056	0.307900812	0.143890095	0.011781846	0.291914869	0.16658526		0.000368395	7.41797E-05	0.0109601	0.059908894
cDC2	0.162775374	0.178674174	0.469801071	0.222423056	0.003360055	0.415960426	0.291914869	0.000368395		0.000460051	0.002555922	0.029541875
cDC3	0.25900614	0.059908894	0.475842198	0.104490658	0.010612528	0.487910563	0.107473027	7.41797E-05	0.000460051		0.01635	0.116737834
pDC	0.006863377	0.252109613	0.492342376	0.109381068	0.005389833	0.209623288	0.247196229	0.0109601	0.002555922	0.01635		0.01526057
Monocytes	0.002768655	0.020045601	0.231790737	0.007199084	0.013045948	0.357817863	0.276284682	0.059908894	0.029541875	0.116737834	0.01526057	

Supplementary Table 12 | Correlation matrix of myeloid cell subset proportions and clinical outcomes. Spearman correlation coefficients and corresponding significance values among myeloid cell subset proportions and clinical outcomes from 14 patients of our HCC immunotherapy cohort.

Supplementary Table 13. Cross-comparison of neoadjuvant αPD-1 translational studies in HCC							
**Category**	**Clinical setting**	**Patient number**	**Clinical endpoint**	**Methodological approaches**	**Metabolic reprogramming**	**Macrophage markers characterized**	**Core discovery of immune subsets**
**Nature Medicine (PMID: 37322116)**	αPD-1	20	Surgery (Pathological response)	scRNA-seq+ST	No	Not specifically defined; general TAM signatures	T cell functional niche
**Molecular Cancer (PMID: 40205519)**	Neoadjuvant αPD-1	16	Surgery (Pathological response)	scRNA-seq+ST	No	Limited TAM characterization	T cell reprogramming related to therapeutic response
**Journal of Hepatology (PMID: 36708811)**	αPD-1	11	Surgery (Pathological response)	scRNA-seq+ST	No	SPP1, TREM2 TAM consisting tumor immune barrier	Immune exclusion barrier architecture
**J Immunother Cancer (PMID: 41330615)**	Neoadjuvant αPD-1	19	Surgery (Pathological response)	Bulk RNA-seq+Bulk TCR-seq	No	Not macrophage-focused	Systemic TCR clonotype dynamics
**Nature Communications (PMID: 38627377)**	Neoadjuvant αPD-1	20	Surgery (Pathological response) DFS and OS rates	Bulk RNA-seq+Bulk TCR-seq	No	Not macrophage-focused	Stereotactic body radiotherapy may systemically facilitate TCR clonal expansion
**Int J Surg (PMID: 38995162)**	Neoadjuvant αPD-1 +Lenvatinib	22	Surgery (Pathological response) RFS rates	No	No	No	No
**Nature Cancer (PMID: 34796337)**	Neoadjuvant αPD-1	15	Surgery (Pathological response)	Nanostring analysis+CyTOF	No	Not specifically defined; general TAM signatures	Spatial arrangement of B cells and plasma cells in responders
**This Study**	Neoadjuvant αPD-1	14	Surgery (Pathological response) RFS and OS rates	scRNA-seq+ST	Intervention of lipid metabolism reprogramming, including modulation of PPARs to improve immunotherapy outcomes	$C1QA^{+}$ $APOA1^{+}$ $FABP1^{+}$ $SPP1^{+}$ LAM induced lipid metabolism–associated immunosuppression and fibrotic function	Identification and modulation of immunosuppressive LAM to improve HCC immunotherapy

Supplementary Table 13 | Cross-comparison of neoadjuvant αPD-1 translational studies in HCC. This table summarizes study design, patient cohorts, clinical endpoints, methodological strategies, characterized macrophages and core discoveries across representative reports, highlighting conceptual commonalities and distinctions relative to our study.

Supplementary Table 14. Sample-level quality control metrics for single-cell RNA sequencing data					
**Patient**	**Cells per sample**	**Median genes per cell**	**Median UMI counts per cell**	**Percentage of mitochondrial reads**	**Doublet rate per sample**
Patient01	3904	739	1465	1.4987323	0.02%
Patient02	5436	1178	1324	1.7257494	0.10%
Patient03	5831	1426	1492	1.4663798	0.05%
Patient04	8182	2099	4881	2.1292548	0.14%
Patient05	10678	1787	4556	1.4227449	0.04%
Patient06	9388	1811.5	4697	1.393781	0.05%
Patient07	10180	1581	1725	2.5503356	0.04%
Patient08	10462	1143	1578	2.1451484	0.11%
Patient09	10843	1082	1549	1.7532874	0.01%
Patient10	5826	1828.5	4597	0.7201276	0.06%
Patient11	8477	1584	3852	2.7114967	0.07%
Patient12	9443	1573	1660	0.506791	0.10%
Patient13	11140	1379	1632	3.6231884	0.13%
Patient14	7776	1787	4691	1.790329	0.08%

Supplementary Table 14 | Sample-level quality control metrics for single-cell RNA sequencing data. Summary of cells captured, median genes and UMIs, median percentage of mitochondrial reads, and estimated doublet rates of 14 patients from our cohort.

Supplementary Table 15. Experiment supplies		
**Experiment supplies**		
**Antibodies**		
**Reagent**	**Source**	**Identifier**
Viability Dye 780	biogems	CAT#62910-0-100
Human TruStain FcX	biolegend	CAT#422301
PE/Cyanine7 anti-human IFN-γ Antibody	biolegend	CAT#502527
BD Horizon BV510 Mouse Anti-Human CD45	BD Biosciences	CAT#563204
BD Horizonm BV421 Mouse Anti-Human CD68	BD Biosciences	CAT#564943
CD3 Monoclonal Antibody (OKT3), eFluor 450, eBioscience	ThermoFisher	CAT#48-0037-42
CoraLite Plus 488-conjugated FABP1 Monoclonal antibody	proteintech	CAT#CL488-68227
CoraLite Plus 647-conjugated Apolipoprotein AI Polyclonal antibody	proteintech	CAT#CL647-14427
Anti-Human Complement Component C1q, PE	CEDARLANE	CAT#CL7611PE
PE/Cyanine7 anti-mouse CD45 Antibody	biolegend	CAT#103113
PE/Cyanine5 anti-mouse CD3 Antibody	biolegend	CAT#100273
FITC anti-mouse CD279 (PD-1) Antibody	biolegend	CAT#135213
PE anti-mouse TIGIT (Vstm3) Antibody	biolegend	CAT#156103
Brilliant Violet 421 anti-mouse Ki-67 Antibody	biolegend	CAT#652411
Alexa Fluor 647 anti-mouse FOXP3 Antibody	biolegend	CAT#126407
Brilliant Violet 421 anti-mouse TNF-α Antibody	biolegend	CAT#506327
PE anti-mouse IFN-γ Antibody	biolegend	CAT#505807
PE/Cyanine5 anti-mouse F4/80 Antibody	biolegend	CAT#123111
Brilliant Violet 421 anti-mouse/human CD11b Antibody	biolegend	CAT#101235
Alexa Fluor 700 anti-human CD206 (MMR) Antibody	biolegend	CAT#321131
FITC anti-mouse CD4 Antibody	biolegend	CAT#100405
Anti-Mouse Complement Component C1q, PE	CEDARLANE	CAT#CL7501PE
Anti-PPAR alpha Rabbit pAb	Epizyme	CAT#P010554
Anti-PPAR delta Rabbit pAb	Epizyme	CAT#R015982
Anti-PPAR gamma Rabbit pAb	Epizyme	CAT#R015221
**Chemicals**		
**Reagent**	**Source**	**Identifier**
Percoll	Yeasen	CAT#40501ES60
Collagenase II	Yeasen	CAT#40508ES60
Collagenase IV	Yeasen	CAT#40510ES60
Hyaluronidase	Yeasen	CAT#20426ES60
Deoxyribonuclease I	Yeasen	CAT#10607ES15
Monensin Solution (1000X)	eBioscience	CAT#00-4505-51
Immunostaining Permeabilization Buffer with Saponin	Beyotime	CAT#P0095-100ml
Invitrogen eBioscience IC Fixation Buffer	eBioscience	CAT#00-8222-49
Propyleneglycol monomethyl ether acetate	aladdin	CAT#108-65-6
Oleic acid	aladdin	CAT#112-80-1
Lycopene	aladdin	CAT#502-65-8
Lymphoprep	STEMCELL	CAT#18060
ImmunoCult-XF T Cell Expansion Medium	STEMCELL	CAT#10981
Lipid Droplets Green Fluorescence Assay Kit with BODIPY 493/503	Beyotime	CAT#C2053S
BeaverBeads Human CD3 Negative Selection Kit	Beaver	CAT#71003-100
Hepatocellular carcinoma organoid culture medium	MasterAim	CAT#10-100-319
Protease inhibitor	Servicebio	CAT#G2006
PMSF	Servicebio	CAT#G2008
RIPA Lysis Buffer	Servicebio	CAT#G2002
TRIzol	Yeasen	CAT#19201ES60
Hiscript qRT-PCR SuperMix	Vazyme	CAT#R122-01
AceQ qPCR SYBR Green Master Mix	Vazyme	CAT#Q111-02
**Primers**		
**Genes**	**Seuqence (5' to 3')**	
Ppara-F	AACATCGAGTGTCGAATATGTGG	
Ppara-R	CCGAATAGTTCGCCGAAAGAA	
Ppard-F	TCCATCGTCAACAAAGACGGG	
Ppard-R	ACTTGGGCTCAATGATGTCAC	
Pparg-F	GGAAGACCACTCGCATTCCTT	
Pparg-R	GTAATCAGCAACCATTGGGTCA	
**Experimental models: cell lines**		
**Cell line**	**Source**	**Identifier**
THP-1	Pricella	CAT#CL-0233
Hepa 1-6	Pricella	CAT#CL-0105
Huh-7	Pricella	CAT#CL-0120
**Experimental models: organisms/strains**		
C57BL/6 mice	Beijing Vital River Experimental Animal Technology Co., Ltd.	N/A
**Software and algorithms**		
**Name**	**Developer**	
FlowJo V10	Tree Star	
Prism 9.5	GraphPad	
R studio	RStudio. Inc., Boston, MAReference	
Python	Python Software Foundation.	
**Others**		
**Name**	**Source**	**Identifier**
40 µm Cell Strainer	Corning	Cat#431750
100 µm Cell Strainer	Corning	Cat#431752
6-well Clear Flat Bottom Ultra-Low Attachment Multiple Well Plates	Corning	Cat#3471

Supplementary Table 15 | Experiment supplies. Summary of of all antibodies, primers, reagents, commercial kits, and other materials used in this study.
