# Supplementary material for: Integrated single-cell multi-omics characterization reveals lipid-associated macrophage-mediated immunosuppression in neoadjuvant immunotherapy of hepatocellular carcinoma
Source: Nat Commun. 2026 Jul 31;17:9381. doi: 10.1038/s41467-026-75949-y (PMC13534469; doi:10.1038/s41467-026-75949-y)
Supplement: Supplementary file 2 [file 41467_2026_75949_MOESM2_ESM.pdf]

nature portfolio

Corresponding author(s): Tao Li & Dongxu Wang & Lei Zhao

Last updated by author(s): Jun 22, 2026

Reporting Summary

Nature Portfolio wishes to improve the reproducibility of the work that we publish. This form provides structure for consistency and transparency in reporting. For further information on Nature Portfolio policies, see our Editorial Policies and the Editorial Policy Checklist.

Statistics

For all statistical analyses, confirm that the following items are present in the figure legend, table legend, main text, or Methods section.

n/a	Confirmed	
[ ]	[x]	The exact sample size (n) for each experimental group/condition, given as a discrete number and unit of measurement
[ ]	[x]	A statement on whether measurements were taken from distinct samples or whether the same sample was measured repeatedly
[ ]	[x]	The statistical test(s) used AND whether they are one- or two-sided<br>*Only common tests should be described solely by name; describe more complex techniques in the Methods section.*
[ ]	[x]	A description of all covariates tested
[ ]	[x]	A description of any assumptions or corrections, such as tests of normality and adjustment for multiple comparisons
[ ]	[x]	A full description of the statistical parameters including central tendency (e.g. means) or other basic estimates (e.g. regression coefficient) AND variation (e.g. standard deviation) or associated estimates of uncertainty (e.g. confidence intervals)
[ ]	[x]	For null hypothesis testing, the test statistic (e.g. F, t, r) with confidence intervals, effect sizes, degrees of freedom and P value noted<br>*Give P values as exact values whenever suitable.*
[x]	[ ]	For Bayesian analysis, information on the choice of priors and Markov chain Monte Carlo settings
[ ]	[x]	For hierarchical and complex designs, identification of the appropriate level for tests and full reporting of outcomes
[ ]	[x]	Estimates of effect sizes (e.g. Cohen's d, Pearson's r), indicating how they were calculated

Our web collection on statistics for biologists contains articles on many of the points above.

Software and code

Policy information about availability of computer code

Data collection	For single cell analysis: Fresh HCC tumor tissue, weighing ≥ 0.2 g, was collected within 30 minutes of excision. Sample processing involved washing with 1X PBS or saline to thoroughly remove red blood cells and prevent clotting, which could interfere with digestion and dissociation. The tumor tissue was then dissociated into single-cell suspension. The single-cell suspension was subjected to quality control and cell counting, with a requirement for cell viability to be above 85%. Cells meeting the quality criteria were washed, resuspended to an appropriate concentration of 700–1200 cells/μl, and prepared for analysis using the 10x Genomics Chromium™ system. GEM Generation and Tagging: Cells were loaded according to the expected number of target cells to construct Gel Bead-in-Emulsion (GEM) droplets for single-cell isolation. Once GEMs were successfully formed, they were collected and subjected to reverse transcription in a PCR machine for tagging. Post-GEM RT Purification and cDNA Amplification: GEMs were processed to break the oil emulsion, and single-strand cDNA was purified and enriched using magnetic beads. The cDNA was then amplified and quality-checked. Library Construction and Quantification: Quality-checked cDNA was used to construct sequencing libraries through fragmentation, adapter ligation, and sample index PCR. The libraries were subsequently quantified and subjected to quality control. Sequencing: The constructed libraries were sequenced using the Illumina HiSeq platforms, employing a PE150 sequencing mode to achieve a read depth of 50,000 read pairs per cell or greater.<br>Raw sequencing data was subjected to quality control analysis using FastQC software. The 10x Chromium single-cell transcriptome was analyzed through 5′ end transcript sequencing, alongside sequencing analysis of T-cell and B-cell receptor gene TCR/BCR sequences. From the Fastq Reads, a cell gene expression matrix was generated, with one of the key steps being the identification of valid cells from background signals. Cell Ranger software was employed to perform data quality statistics on the raw Fastq data, align it to the Ensemble reference genome, carry out cell demultiplexing, remove PCR duplicates, and identify cells. This software quantitatively analyzes high-throughput single-cell transcriptome results by recognizing barcode sequences that distinguish cells and UMI tags that label different mRNA molecules within each cell.<br>For spatial transcriptomics analysis: The spatial features of tissue samples were analyzed using the DynaSpatial FFPE Spatial Gene Expression Kit for Human Transcriptome. Specifically, FFPE tissue sections were cut and placed on adhesive slides, followed by H&E staining. The gene

expression slides were used to generate spatially barcoded cDNA from tissue sections following the manufacturer's instructions. Next, the extended probe products were denatured and transferred from the capture area to a new tube, where they were amplified via PCR to generate sufficient material. Then, the sequencing library was then prepared following the DynaSpatial library preparation protocol. Finally, paired-end 150 bp sequencing was conducted on the Illumina NovaSeq X Plus.

Data analysis

Raw sequencing data were processed with the Cell Ranger software (version 4) using default parameters. The reads were aligned to the GRCh38 human reference genome, and we generated gene expression matrices using the STAR aligner.
Dimensionality reduction & cell type identification: Normalization and variance stabilization of scRNA-seq data were performed using Seurat (version 5.1.0). For dimensionality reduction and clustering analysis, a principal component analysis was performed, and 15 principal components was used to compute the Uniform Manifold Approximation & Projection (UMAP) dimensions and perform Louvain clustering at varying resolutions, ranging from 0.1 to 1.0. Differential gene expression analysis of previously characterized cell type-specific genes was used to identify the cell type for each cluster.
Differential gene expression analysis: To identify marker genes, differential gene expression analysis of all clusters was performed using Seurat (version 5.1.0) in merged datasets. Genes considered in the analysis needed to be expressed in at least 25% of cells in a cluster with a minimum natural log fold change of 0.25. All outputs of this analysis are included as supplemental tables. The gene filtering parameters used to generate dot plot from differential gene expression analysis are described in the legends.
InferCNV: we used the InferCNV(version 1.20.0) to estimate the copy number variations (CNVs). B cells were used as normal reference and the parameters were default. The sum of calculated CNV for each gene per cell was defined as the CNV score of the cell.
Trajectory analysis: We used Monocle2 (version 2.32.0) and Monocle3 to infer the cell lineage trajectory of T cells and myeloid cells with the top 700 signature genes with q value < 0.001 calculated by differential GeneTest function. The differentiation trajectory was inferred with the default parameters of Monocle after dimension reduction and cell ordering.
Cell-cell interaction analysis: We used CellChat (version 2.1.2) to infer cell-cell interaction between different cell types. This method infers the potential interaction strength between two cell subsets based on gene expression level and provides the significance through permutation test (1000 times). The enriched ligand-receptor interactions between two cell subsets were calculated based on the permutation test. We extracted significant ligand-receptor pairs with P value < 0.01.
Single-cell regulatory network inference and clustering (SCENIC) : SCENIC (version 1.3.1) was used to encompasses a tripartite process: coexpression analysis, target gene motif enrichment analysis, and regulon activity evaluation.
Spatial transcriptomics analysis: The sequencing data were processed through the standard pipeline from DynaSpatial to generate expression matrix. The R package Seurat was then used for downstream analysis, including the normalization of expression matrix, Principal Component Analysis (PCA), clustering, and the t-SNE dimensionality reduction. In order to identify the specific cell types, we adopted RCTD approach (R package spacexr, 1.2.0) by using the annotated single-cell atlas of HCC immunotherapy as reference dataset, and the identification of spatial transcriptome data was then verified by the expression features of each cell type.

For manuscripts utilizing custom algorithms or software that are central to the research but not yet described in published literature, software must be made available to editors and reviewers. We strongly encourage code deposition in a community repository (e.g. GitHub). See the Nature Portfolio guidelines for submitting code & software for further information.

Data

Policy information about availability of data

All manuscripts must include a data availability statement. This statement should provide the following information, where applicable:

- Accession codes, unique identifiers, or web links for publicly available datasets
- A description of any restrictions on data availability
- For clinical datasets or third party data, please ensure that the statement adheres to our policy

The raw scRNA-seq data generated in this study have been deposited in the Genome Sequence Archive for Human (GSA-Human) under project number HRA011844. [https://ngdc.cncb.ac.cn/gsa-human/browse/HRA011844]. As related to human genetic resource, the data deposited and made public for non-commercial purposes, and are compliant with the regulations of the Ministry of Science and Technology of China. The publicly available datasets used in this study are available under accession codes (skrx2fz79n [https://data.mendeley.com/datasets/skrx2fz79n/1]. GSE149614 [https://www.ncbi.nlm.nih.gov/geo/query/acc.cgi?acc=GSE149614], GSE151530 [https://www.ncbi.nlm.nih.gov/geo/query/acc.cgi?acc=GSE151530], GSE156625 [https://www.ncbi.nlm.nih.gov/geo/query/acc.cgi?acc=GSE156625], GSE189903 [https://www.ncbi.nlm.nih.gov/geo/query/acc.cgi?acc=GSE189903], GSE202642 [https://www.ncbi.nlm.nih.gov/geo/query/acc.cgi?acc=GSE202642] and GSE206325 [https://www.ncbi.nlm.nih.gov/geo/query/acc.cgi?acc=GSE206325]. Additionally, Source Data are provided with this paper.

Research involving human participants, their data, or biological material

Policy information about studies with human participants or human data. See also policy information about sex, gender (identity/presentation), and sexual orientation and race, ethnicity and racism.

Reporting on sex and gender

This study included both male and female patients with hepatocellular carcinoma undergoing neoadjuvant immunotherapy. Biological sex was recorded for all participants based on self-reporting, and informed consent was obtained from all patients for the use and sharing of de-identified individual-level data.
In total, 22 male and 8 female patients were included in the clinical cohort. Gender was not separately assessed or analyzed in this study. Sex was considered in the study design at the level of cohort inclusion; however, no sex-stratified analyses were performed, as the primary conclusions of this study and immunotherapy response were not driven by, nor appeared to differ according to, patient sex.
Accordingly, the findings are applicable to both sexes. Disaggregated sex information is provided in the supplementary table and summarized in this Reporting Summary. No additional gender-based analyses were conducted, as gender-related variables were not collected.

Reporting on race, ethnicity, or other socially relevant groupings

In this study, the only socially relevant categorization variable collected and reported was biological sex. Race, ethnicity, or other socially relevant informations were collected or used in the analyses.

Population characteristics

Liver tissue specimens were collected from patients diagnosed with primary hepatocellular carcinoma (HCC) and HCC

	patients under anti-PD-1 immunotherapy undergoing surgery at Qilu Hospital.
Recruitment	All clinical patients from whom samples were collected provided informed consent
Ethics oversight	The study protocol adhered to the principles of the Declaration of Helsinki and was approved by the Institutional Review Board and Ethics Committee of Qilu Hospital of Shandong University(Approval Nos. KYLL-2024(ZM)-1324). Additionally, this study was conducted within the framework of our prospectively registered perioperative clinical research program (ClinicalTrials.gov identifier: NCT06571396).

Note that full information on the approval of the study protocol must also be provided in the manuscript.

Field-specific reporting

Please select the one below that is the best fit for your research. If you are not sure, read the appropriate sections before making your selection.

[x] Life sciences [ ] Behavioural & social sciences [ ] Ecological, evolutionary & environmental sciences

For a reference copy of the document with all sections, see nature.com/documents/nr-reporting-summary-flat.pdf

Life sciences study design

All studies must disclose on these points even when the disclosure is negative.

Sample size	For HCC samples, 14 patients under anti-PD-1 immunotherapy were selected to explore the heterogeneity of HCC tumor immune microenvironment. Additionally, 10 patients under immunotherapy were collected to conduct flow cytometry and immunohistochemistry analyses. 6 primary HCC patients were collected for organoid construction.
Data exclusions	Cells that did not pass quality control were excluded from the scRNA-seq analysis.
Replication	Analytical workflows for scRNA-seq were repeated at least twice to confirm reproducibility of dimensionality reduction and clustering. All experiments were independently repeated three times.
Randomization	For patients, randomization not applicable. Groupings for analysis were based on tissue characteristics. For animal experiments, mice were randomly allocated to treatment groups where applicable.
Blinding	Blinding was not performed in this project because all analysis and experiments were assigned into groups with relevant controls and analysis were done objectively without bias

Reporting for specific materials, systems and methods

We require information from authors about some types of materials, experimental systems and methods used in many studies. Here, indicate whether each material, system or method listed is relevant to your study. If you are not sure if a list item applies to your research, read the appropriate section before selecting a response.

Materials & experimental systems

n/a	Involved in the study	
[ ]	[x]	Antibodies
[ ]	[x]	Eukaryotic cell lines
[x]	[ ]	Palaeontology and archaeology
[ ]	[x]	Animals and other organisms
[ ]	[x]	Clinical data
[x]	[ ]	Dual use research of concern
[x]	[ ]	Plants

Methods

n/a	Involved in the study	
[x]	[ ]	ChIP-seq
[ ]	[x]	Flow cytometry
[x]	[ ]	MRI-based neuroimaging

Antibodies

Antibodies used

Reagent Source Identifier
Viability Dye 780 biogems CAT#62910-0-100
Human TruStain FcX™ biolegend CAT#422301
PE/Cyanine7 anti-human IFN-γ Antibody biolegend CAT#502527
BD Horizon™ BV510 Mouse Anti-Human CD45 BD Biosciences CAT#563204
BD Horizonm BV421 Mouse Anti-Human CD68 BD Biosciences CAT#564943
CD3 Monoclonal Antibody (OKT3), eFluor™ 450, eBioscience™ ThermoFisher CAT#48-0037-42
CoraLite® Plus 647-conjugated Apolipoprotein AI Polyclonal antibody proteintech CAT#CL647-14427
Anti-Human Complement Component C1q, PE CEDARLANE CAT#CL7611PE
PE/Cyanine7 anti-mouse CD45 Antibody biolegend CAT#103113
PE/Cyanine5 anti-mouse CD3 Antibody biolegend CAT#100273

FITC anti-mouse CD279 (PD-1) Antibody biolegend CAT#135213
PE anti-mouse TIGIT (Vstm3) Antibody biolegend CAT#156103
Brilliant Violet 421™ anti-mouse Ki-67 Antibody biolegend CAT#652411
Alexa Fluor® 647 anti-mouse FOXP3 Antibody biolegend CAT#126407
Brilliant Violet 421™ anti-mouse TNF-α Antibody biolegend CAT#506327
PE anti-mouse IFN-γ Antibody biolegend CAT#505807
PE/Cyanine5 anti-mouse F4/80 Antibody biolegend CAT#123111
Brilliant Violet 421™ anti-mouse/human CD11b Antibody biolegend CAT#101235
Alexa Fluor® 700 anti-human CD206 (MMR) Antibody biolegend CAT#321131
FITC anti-mouse CD4 Antibody biolegend CAT#100405
Anti-Mouse Complement Component C1q, PE CEDARLANE CAT#CL7501PE
Anti-PPAR alpha Rabbit pAb Epizyme CAT#P010554
Anti-PPAR delta Rabbit pAb Epizyme CAT#R015982
Anti-PPAR gamma Rabbit pAb Epizyme CAT#R015221

Validation

Authors have extensively validated each antibody with associated positive and negative controls, and are highly familiar with the importance of optimizing staining conditions to avoid non-specific staining, and orthogonal approaches to increase confidence of antibody-based staining.
Validation data are available on the manufacturer's websites
1. Viability Dye 780 (https://www.bio-gems.com/viability-dye-780.html)
2. Human TruStain FcX™ (https://www.biolegend.com/en-gb/products/human-trustain-fcx-fc-receptor-blocking-solution-6462)
3. PE/Cyanine7 anti-human IFN-γ Antibody (https://www.biolegend.com/en-gb/products/pe-cyanine7-anti-human-ifn-gamma-antibody-5939)
4. BD Horizon™ BV510 Mouse Anti-Human CD45 (https://www.bdbiosciences.com/zh-cn/products/reagents/flow-cytometry-reagents/research-reagents/single-color-antibodies-ruo/bv510-mouse-anti-human-cd45.563204?tab=product_details)
5. BD Horizonm BV421 Mouse Anti-Human CD68 (https://www.bdbiosciences.com/zh-cn/products/reagents/flow-cytometry-reagents/research-reagents/single-color-antibodies-ruo/bv421-mouse-anti-human-cd68.564943?tab=product_details)
6. CD3 Monoclonal Antibody (OKT3), eFluor™ 450, eBioscience™ (https://www.thermofisher.com/antibody/product/CD3-Antibody-clone-OKT3-Monoclonal/48-0037-42)
7. CoraLite® Plus 647-conjugated Apolipoprotein AI Polyclonal antibody (https://www.ptgcn.com/products/Apolipoprotein-AI-Antibody-CL647-14427.htm)
8. Anti-Human Complement Component C1q, PE (https://www.cedarlanelabs.com/Products/Detail/CL7611PE?lob=AllProducts)
9. PE/Cyanine7 anti-mouse CD45 Antibody (https://www.biolegend.com/en-gb/products/pe-cyanine7-anti-mouse-cd45-antibody-1903)
10. PE/Cyanine5 anti-mouse CD3 Antibody (https://www.biolegend.com/en-gb/products/pe-cyanine5-anti-mouse-cd3-antibody-21198)
11. FITC anti-mouse CD279 (PD-1) Antibody (https://www.biolegend.com/en-gb/products/fitc-anti-mouse-cd279-pd-1-antibody-7004)
12. PE anti-mouse TIGIT (Vstm3) Antibody (https://www.biolegend.com/en-gb/products/pe-anti-mouse-tigit-vstm3-antibody-16484)
13. Brilliant Violet 421™ anti-mouse Ki-67 Antibody (https://www.biolegend.com/en-gb/products/brilliant-violet-421-anti-mouse-ki-67-antibody-8982)
14. Alexa Fluor® 647 anti-mouse FOXP3 Antibody (https://www.biolegend.com/en-gb/products/alexa-fluor-647-anti-mouse-foxp3-antibody-4662)
15. Brilliant Violet 421™ anti-mouse TNF-α Antibody (https://www.biolegend.com/en-gb/products/brilliant-violet-421-anti-mouse-tnf-alpha-antibody-7336)
16. PE anti-mouse IFN-γ Antibody (https://www.biolegend.com/en-gb/products/pe-anti-mouse-ifn-gamma-antibody-997)
17. PE/Cyanine5 anti-mouse F4/80 Antibody (https://www.biolegend.com/en-gb/products/pe-cyanine5-anti-mouse-f4-80-antibody-4069)
18. Brilliant Violet 421™ anti-mouse/human CD11b Antibody (https://www.biolegend.com/en-gb/products/brilliant-violet-421-anti-mouse-human-cd11b-antibody-7163)
19.Alexa Fluor® 700 anti-human CD206 (MMR) Antibody (https://www.biolegend.com/en-gb/products/alexa-fluor-700-anti-human-cd206-mmr-antibody-13264)
20.FITC anti-mouse CD4 Antibody (https://www.biolegend.com/en-gb/products/fitc-anti-human-cd4-antibody-8738)
21. Anti-Mouse Complement Component C1q, PE (https://www.cedarlanelabs.com/Products/Detail/CL7501PE?lob=AllProducts)
22. Anti-PPAR alpha Rabbit pAb (https://www.epizyme.cn/Products/Details/M014541)
23. Anti-PPAR delta Rabbit pAb (https://www.epizyme.cn/Products/Details/R015982)
24. Anti-PPAR gamma Rabbit pAb (https://www.epizyme.cn/Products/Details/R015221)

Eukaryotic cell lines

Policy information about cell lines and Sex and Gender in Research

Cell line source(s)

Cell line	Source	Identifier
THP-1	Pricella	CAT#CL-0233
Hepa 1-6	Pricella	CAT#CL-0105
Huh-7	Pricella	CAT#CL-0120

Authentication	All human cell lines were authenticated by short tandem repeat (STR) profiling. Cell line authentication was performed by the cell bank using STR profiling prior to distribution.<br>The murine hepatocellular carcinoma cell line Hepa1-6 was obtained from Pricella and used at low passage numbers.
Mycoplasma contamination	All cell lines were tested negative for mycoplasma contamination
Commonly misidentified lines<br>(See ICLAC register)	No commonly misidentified cell lines were used

Animals and other research organisms

Policy information about studies involving animals; ARRIVE guidelines recommended for reporting animal research, and Sex and Gender in Research

Laboratory animals	C57BL/6J (6 weeks old) were used for HCC research.
Wild animals	No wild animals were used in our research.
Reporting on sex	All animal experiments in this study were conducted using male mice only. This choice was made because the high-fat diet–induced metabolic model used to study lipid accumulation is known to be more stable and reproducible in male mice, whereas female mice exhibit significant variability due to hormonal cycling, which can substantially affect lipid metabolism and immune responses. Sex was therefore not used as a variable for group assignment, and no sex-based comparisons were performed. All reported animal numbers in the manuscript and Reporting Summary correspond exclusively to male mice. Female mice were not included in this study, and sex-disaggregated analyses were therefore not applicable.
Field-collected samples	No field-collected samples were used in our research.
Ethics oversight	All animal experiments were approved by the Committee on Ethics of Animal Experiments at the Qilu hospital of Shandong University (IACUC Issue NO. DWLL-202500262).

Note that full information on the approval of the study protocol must also be provided in the manuscript.

Clinical data

Policy information about clinical studies

All manuscripts should comply with the ICMJE guidelines for publication of clinical research and a completed CONSORT checklist must be included with all submissions.

Clinical trial registration	ClinicalTrials.gov ID: NCT06571396
Study protocol	Full trial protocol can be accessed in https://www.clinicaltrials.gov/study/NCT06571396
Data collection	All clinical specimens were obtained from patients treated at Qilu Hospital of Shandong University (Jinan, China). Patient enrollment, surgical specimen collection, and associated clinical data acquisition were carried out between June 2022 and January 2026.
Outcomes	The primary outcome measure is perioperative risk. The criteria for perioperative risk include four aspects: operation time, transfusion, postoperative hospital stay, and complication. Operation time >200min is counted as 3 points, transfusion is counted as 2 points, postoperative hospital stay >8 days is counted as 2 points, and complication is counted as 1 point. The total score range is 0-8 points. Participants with a score of 3 or lower are considered to have a low perioperative risk, while those above 3 are considered to have a high perioperative risk.<br>The secondary outcome measures are progression free survival time (RFS) and overall survival time (OS). RFS and OS were obtained through postoperative follow-up.

Plants

Seed stocks	*Report on the source of all seed stocks or other plant material used. If applicable, state the seed stock centre and catalogue number. If plant specimens were collected from the field, describe the collection location, date and sampling procedures.*
Novel plant genotypes	*Describe the methods by which all novel plant genotypes were produced. This includes those generated by transgenic approaches, gene editing, chemical/radiation-based mutagenesis and hybridization. For transgenic lines, describe the transformation method, the number of independent lines analyzed and the generation upon which experiments were performed. For gene-edited lines, describe the editor used, the endogenous sequence targeted for editing, the targeting guide RNA sequence (if applicable) and how the editor was applied.*
Authentication	*Describe any authentication procedures for each seed stock used or novel genotype generated. Describe any experiments used to assess the effect of a mutation and, where applicable, how potential secondary effects (e.g. second site T-DNA insertions, mosiacism, off-target gene editing) were examined.*

Flow Cytometry

Plots

Confirm that:

- [x] The axis labels state the marker and fluorochrome used (e.g. CD4-FITC).
- [x] The axis scales are clearly visible. Include numbers along axes only for bottom left plot of group (a 'group' is an analysis of identical markers).
- [x] All plots are contour plots with outliers or pseudocolor plots.
- [x] A numerical value for number of cells or percentage (with statistics) is provided.

Methodology

Sample preparation	For PBMC-derived immune cells, peripheral blood mononuclear cells (PBMCs) were isolated from peripheral blood using Lymphoprep™ density gradient separation according to the manufacturer's instructions. For tumor-derived immune cells, human or mouse tumor nodules were microdissected from liver tissues, minced into small fragments, and enzymatically digested with collagenase IV (1 mg/mL) and DNase I (0.25 mg/mL) at 37°C for 60 min. The resulting cell suspensions were filtered through a 70 µm nylon cell strainer. For flow cytometry analysis of mice tumor samples, the obtained single-cell suspensions were subsequently divided into five staining tubes averagely. Separate panels were used to evaluate LAM proportion and different functional aspects of T cells, including effector function (IFN-γ, TNF), proliferation (KI67), exhaustion (PD-1, TIGIT), and regulatory T cells (FOXP3).<br>The cells obtained after digestion were resuspended in a staining buffer and incubated with Fc block (BioLegend) for 10 min. Then surface antibodies were added and stained for 30 min at 4°C in the dark. Cells were fixed by IC Fixtion Buffer (ebioscience) for 30 min at 4°C in the dark, permeabilized by Permeabilization Buffer (Beyotime) for 30 min at 4°C in the dark and then washed twice with diluted 1×PBS. Subsequently, intracellular antibodies were added and stained for 30 min at 4°C in the dark. Finally, cells were analyzed by flow cytometer (BD Biosciences).
Instrument	BD Celesta was used for flow cytometry data collection
Software	FlowJo 10.8.1
Cell population abundance	We use lymphoprep and percoll to enrich the immune cells in peripheral blood and tissue.
Gating strategy	A gate was set on single cell population based on forward and side scatter properties. The gating strategies was provided in Fig. 6d, f and Supplementary Fig. 7a, b

- [x] Tick this box to confirm that a figure exemplifying the gating strategy is provided in the Supplementary Information.
