# Supplementary material for: Integrated single-cell multi-omics characterization reveals lipid-associated macrophage-mediated immunosuppression in neoadjuvant immunotherapy of hepatocellular carcinoma
Source: Nat Commun. 2026 Jul 31;17:9381. doi: 10.1038/s41467-026-75949-y (PMC13534469; doi:10.1038/s41467-026-75949-y)
Supplement: Supplementary file 3 [file 41467_2026_75949_MOESM3_ESM.pdf]

nature portfolio

Peer Review File

Integrated Single-cell Multi-omics Characterization Reveals Lipid-associated Macrophage-mediated Immunosuppression in Neoadjuvant Immunotherapy of Hepatocellular Carcinoma

Corresponding Author: Professor Tao Li

Parts of this Peer Review File have been redacted as indicated to remove third-party material.

This file contains all reviewer reports in order by version, followed by all author rebuttals in order by version.

Version 0:

Reviewer comments:

Reviewer #1

(Remarks to the Author)
Peng et al performed single-cell analysis of tumor tissue of patients who were previously treated by PD-1 (Tislelizumab or Camrelizumab). I agree that there have been relatively few studies dissecting the TIME (tumor immune microenvironment) in patients undergoing immunotherapy using single-cell sequencing, and I appreciate the effort made. However, the tissue was collected only after the PD-1 treatment. Therefore, there is no comparison with the situation before the treatment. Given the well-documented heterogeneity of HCC, it is challenging to draw conclusions about predictive TIME factors for immunotherapy response based solely on post-treatment samples.
The study rather details the picture about TIME that appears as a consequence of the treatment in responding or non-responding patients. In other words, it would be more accurate to describe the data as reflecting TIME components that result from the efficacy or inefficacy of PD-1 blockade, rather than presenting them as TIME components that influence clinical efficacy, as currently stated in the manuscript (line 326).
The authors observed that a lipid-associated macrophage subtype—characterized by expression of C1QA, APOA1, and FABP1—was more frequently present in the TIME of patients who showed a poor response to PD-1 blockade. Notably, APOA1 and FABP1 are typically expressed by hepatocytes, while C1QA is a marker of liver-resident macrophages. This raises the possibility that these markers may not reflect a true macrophage subtype, but rather result from contamination, originating from lysed cells and fat vesicles that were phagocytosed by macrophages—artificially inflating the expression of these genes in transcriptomic profiles (https://doi.org/10.1182/blood.2023020597). In vitro studies (Figure 6) focused on APOA1 only, not on FABP1. Why was FABP1 excluded from the confirmation experiments?

The observation – more macrophages with subtype positive for C1QA, APOA1, and FABP1 - could be simply linked to increased hepatic steatosis in non-responders. Unfortunately, data on the underlying liver etiology of the analyzed patients is not included in the Table S2. Previous studies (e.g., Pfister et al Nature 2021) have shown that anti-PD-1 monotherapy is less effective in HCC patients with NAFLD or NASH etiology. It remains a question whether the findings in this study are related to these prior observations. Data from Figure 2 show a negative correlation between therapy efficacy and Fatty acid associated HCC, and lipid-associated markers. Thus, the liver etiology of patients, and the level of liver steatosis need to be included in the manuscript and discussed in results and discussion in context of the study from Pfister et al Nature 2021.

As in some cases of non-responders, no residual tumor were observed, what was then the tissue collected? Based on the data it seem that those were only immune cells in necrotic area? he data suggest these were immune cells in necrotic areas. In such cases, is it valid to speak of “characterizing the TIME of HCC” when no viable tumor cells were present?

Five external cohorts (61 HCC cases) did not helped in the characterizations of TIME components associated with immunotherapy outcomes, as these patients were not treated by immunotherapy in that 5 cohorts. As I understood, the 5 external cohorts served rather as a control for analysis of the cohort of 14 patients. Therefore these data should be moved to supplementary part and study should focus on the main message already from the first figure.

Lipid-associated macrophages are commonly referred to as LAMs, not "MacroLA." Why did the authors introduce a new abbreviation, when "LAMs" is already well established in the field? Moreover, TREM2⁺ LAMs have previously been described in HCC (PMID: 35359989), and this should be acknowledged and discussed.

Many of the figures are confusing and do not clearly support the study's main message—for example, Figure 1H. In my view, such figures are more distracting than informative. A more focused presentation would be beneficial.

The correlations presented in the study are not clearly defined, particularly with regard to the clinical outcome data. For example, the clinical outcome appears to be represented on a 0–1 scale, but the criteria for this scoring system are not explained. The manuscript refers to this score as a measure of "immunotherapy efficacy" or "response", but a precise definition or explanation of how it was derived is missing. This lack of clarity makes it difficult to interpret the validity.

Figure 1C: I am surprised by mixing T and NK cells in the same cluster characterised by CD3D,CD3E, CD3G etc. NK cells are known to be CD3-. Can authors comment on this point?

Legends of graphs are often not completed: for instance, Figure 2E, what are the value 0-1, and the second value 0-1?

Table S1: "Expression characteristics of each major cell type" – This list raises many questions about the clusters. HCC markers are rather hepatocyte markers, so their presence suggests hepatic origin, but they are not specific enough to be definitive for HCC. Why AFP (Alpha-fetoprotein) or GPC3 (Glypican-3) were not included? Some markers listed as NK cell markers, are not relevant for NK cells and indicate contamination by other cell types. TRDC – TCR delta chain; associated with γδ T cells, not NK cells. KRT81, KRT86 are typically expressed in epithelial cells, and CLIC3 is also not an established NK cell marker, at least to my knowledge. Concerning T cells: TUBA4A – A tubulin gene is ubiquitously expressed, should not be used as T cell marker. SPOCK2 is an ECM-related gene not specific to T cells, and its expression is generally low in immune cells. PTPRC (CD45) is a pan-leukocyte marker that is expressed in all lymphocytes, not specifically in T cells.

Figure 4: DCs are traditionally marked by genes like CLEC4C (BDCA2), TCF4, IRF7, IL3RA (CD123), but GZMB expression is generally absent or very low in bona fide pDCs. In this study, GZB is used as a marker of pDC, which is very surprising to me.

The English requires revision, as some sentences lack clarity or coherence—for example, sentence: "To address these issues above, our study established a cohort of 14 HCC patients undergoing neoadjuvant immunotherapy and performed single-cell sequencing on their resected tumor lesions." Should be replaced by "To address these issues, we recruited 14 patients with HCC undergoing neoadjuvant immunotherapy and performed single-cell sequencing on resected tumour specimens." As "The study" can't establish a cohort.

Reviewer #2

(Remarks to the Author)
Summary:
In this study, the authors perform single-cell RNA sequencing (scRNA-seq) on 14 hepatocellular carcinoma (HCC) specimens obtained from patients who received neoadjuvant anti-PD-1 immune checkpoint inhibitor (ICI) prior to surgical resection. The primary aim of the study is to elucidate mechanisms underlying resistance to ICI in HCC and to characterize tumor immune microenvironment (TIME) features associated with treatment response. Through a comparative analysis of responders (R) versus non-responders (NR), the authors identify a fatty-acid associated HCC subtype (HCC_FA) negatively correlated with ICI efficacy.They further identify key immune components associated with clinical outcome. Specifically, enhanced infiltration of CD4⁺ T helper cells, CD8⁺ cytotoxic T lymphocytes, and inflammatory macrophages (MacroInflam) is associated with treatment response, while enrichment of CD4⁺ regulatory T cells and a lipid-associated macrophage (MacroLA) population expressing C1QA, FABP1, and APOA1 is linked to resistance. Their findings are further supported by validation in multiple publicly available HCC scRNA-seq datasets and posterior preclinical assays (in vitro), flow cytometry and IHQ. The study effectively recapitulates previously reported associations, including reduced T cell exhaustion and elevated CD4⁺ helper activation in responders, while also identifying novel immune subtypes, such as a metabolically distinct HCC subtype (HCC_FA) and the MacroLA population, which may influence therapeutic outcomes.
This manuscript represents a well-executed and thoughtfully designed study. The hypotheses are clearly articulated, the methods are rigorous and appropriately applied, and the results are coherently presented. The conclusions are well-supported by the data, and the discussion clearly contextualizes the findings within the current landscape of immunotherapy in HCC. The identification of lipid-associated macrophages as mediators of immunotherapy resistance and the link with metabolic HCC subtypes offer novel mechanistic insights with potential translational relevance. The work contributes significantly to the understanding of the heterogeneity of immunotherapy responses in HCC and may contribute to the future development of predictive biomarkers and combinatorial therapeutic strategies.

Below, I provide constructive comments and suggestions aimed at improving the quality of this manuscript and encouraging further discussion and clarification of certain results.

Major Points for Discussion:
1. The observed response rate in the study cohort (8 responders vs. 6 non-responders) appears higher than the typically

reported ~20-30% response to immunotherapy in HCC. Can the authors clarify whether this discrepancy may be due to differences in disease stage (e.g., early-stage, resectable HCC vs. advanced, unresectable), the use of neoadjuvant rather than palliative immunotherapy, or other clinical factors?
2. In the clinical cohort, patients received Camrelizumab, Tislelizumab or Sintilimab. Are there known differences between these PD-1 inhibitors? Please, clarify whether differences in dosing regimens or administration protocols could have been applied.
3. The scRNA-seq datasets referenced in the manuscript sum to 60, not 61 samples as stated (e.g., GSE156625 in ref. 23 includes 14 samples). Please reconcile this discrepancy and provide a detailed table summarizing each dataset, including sample size, treatment status, and clinical context.
4. The datasets used are heterogeneous in terms of treatment status, disease stage, etiology (e.g., MASH-related HCC), and other clinical variables. Did the authors observe any associations between MacroLA frequency or HCC_FA subtype and these clinical parameters beyond response status? This is a very relevant aspect to address since MASH disease is highly associated with hepatic lipids accumulation and fatty acid metabolism.
5. Have the authors examined whether pre-defined signatures previously associated with immunotherapy response in HCC (ex. Cappuyns, S., et al. Single-cell RNA sequencing-derived signatures define response patterns to atezolizumab + bevacizumab in advanced hepatocellular carcinoma. J Hepatol (2024)) are validated in their cohort?
6. In Figure S1C, all datasets show consistent TIME composition except for GSE189903, which has fewer HCC cells and a higher proportion of T/NK cells. Do the authors attribute this to technical differences in tissue dissociation or biological differences (e.g., tumor reactivity or early-stage disease)? Could this hypothesis be supported by the similarity with the CPR cohort shown in Figure S1F?
7. In Figure 2I, the authors validate their findings using two independent scRNA-seq datasets. Why were these not included in earlier integrative analyses? Sample sizes of each validation cohort should be clearly stated.
8. In Figures 3A and S3A, 12 T/NK clusters are identified. The manuscript states that clustering was recapitulated only in the authors' own cohort. Does this mean the clustering was not reproducible in external datasets, or that these datasets were not analyzed in other datasets?
9. High naïve CD8$^+$ T cell frequency could reflect poor antigen exposure and impaired effector function. Differences in naïve CD8$^+$ T cell proportions have been linked to response in prior studies. Was a similar association observed in this study?
10. Page 7, lines 265–269 refer to several markers (TNF, IL7R, PDCD1, LAG3, FOXP3), but only IL7R and PDCD1 are shown in Figure S3C. Please ensure that all highlighted markers are included in the figures referenced. A similar issue occurs in Figure 4F–G and S4C for macrophage markers.
11. In Figure 3J, antigen presentation-related pathways are enriched in non-responders, which is unexpected in T cells (which typically do not present antigen but recognize). Could the authors comment on whether this is an artifact of pathway annotation or if it reflects aberrant gene expression signatures in T cells?
12. While macrophage subtypes are well-characterized, the role of other relevant myeloid populations such as MDSCs is not addressed, did authors check if this population was present in their cohort? On the other hand, monocytes appear to be enriched in responders (Figure 5D). Could the authors elaborate on these findings and their potential clinical implications?
13. It would greatly strengthen the manuscript to include histological assessment (e.g., Masson's trichrome, Sirius red, Oil red…) of fibrosis and lipid accumulation to support the proposed functional roles of MacroLA in ECM remodeling and steatosis.
14. Including staining from a responder sample in parallel to the non-responders in Figure C would provide a useful comparison. In Figure 6D, adding cytometry data from a responder (as negative control) or including multiple NR samples would improve scientific rigor (N=1 is insufficient). If this additional samples cannot be provided, consider moving these data to Supplementary figure.
15. Figure 7C shows differential expression of MHC-I and MHC-II molecules between R and NR. Given the relevance of antigen presentation in immunotherapy response, a deeper discussion of these findings is warranted. (This may be discussed together with my prior comment #11)
16. Line 536, Page 13 states that MacroLA promote ECM fibrosis via SPP1 and FN1. This would be better substantiated by direct histological evidence (e.g., fibrosis staining) of induced fibrosis in patients (non-responders) with higher MacroLA.

Minor Comments:
1. The current title is overly long (29 words) and descriptively dense. A more concise and impactful title is recommended.
2. Abstract (Page 1, line 34): Please replace "hepatocellular carcinoma" with the abbreviation "HCC," as the term has already been introduced.
3. Minor grammatical inconsistencies and typographical errors are present and should be corrected. For example, "respons" (Page 12, line 479) should be "response."
4. More details in the statistical methos section or including references of the computational methods may provide stronger transparency and reproducibility.
5. Title of Results 1 may be revised to "Cohort of Patients with HCC Treated with Immunotherapy" instead of "HCC Immunotherapy Cohort."

In conclusion, this study is a significant and high-quality contribution to the field of HCC immunotherapy. The findings are compelling and novel, by the identification of a lipid-associated macrophage subtype that may underlie immunotherapy resistance. Addressing the above comments will further strengthen the clarity, rigor, and translational relevance of the work.

Reviewer #3

(Remarks to the Author)
This manuscript by Peng et al. integrates single-cell transcriptomic data from 14 hepatocellular carcinoma (HCC) patients

treated with neoadjuvant PD-1/PD-L1 blockade together with five large publicly available HCC scRNA-seq datasets (GSE149614, GSE151530, GSE156625, GSE189903, GSE202642). The authors identify responder-associated $CD4^+$ and $CD8^+$ T cell states, and highlight a macrophage population termed "MacroLA," enriched in non-responders, which they propose as immunosuppressive. Functional assays using THP-1–derived macrophages, patient-derived organoids, and lycopene supplementation are presented as supporting evidence. Spatial transcriptomic data (PMID: 36708811; Mendeley skrx2fz79n) are also incorporated.

The integration of large public datasets with new single-cell data from a neoadjuvant immunotherapy cohort is a major strength and reflects substantial work. The dataset will likely be useful for the field. However, the manuscript suffers from several critical limitations in terms of novelty, clinical reporting, methodological rigor, macrophage annotation, and functional validation. These issues significantly limit the mechanistic conclusions and translational impact.

Major concerns:

1. The clinical description in the current manuscript is incomplete and requires substantial clarification. Response assessment is presented only using RECIST 1.1 and CPR/MPR categories, but no information is provided on recurrence-free survival (RFS) or overall survival (OS)—both of which are essential endpoints in the neoadjuvant immunotherapy setting and are routinely reported in comparable studies. In addition, patient demographics and critical treatment details are missing or insufficiently described. Specifically, the manuscript does not provide: Major inclusion and exclusion criteria for trial enrollment. The exact PD-1 inhibitor used for each patient (tislelizumab or camrelizumab). Dosing schedule, number of cycles, and whether the regimens were standardized across patients. The interval from last dose to surgery, whether any perioperative delays occurred, and how long patients remained on treatment prior to surgery. Reporting of treatment-emergent adverse events (AEs) or surgical complications, which are crucial for evaluating feasibility and safety in the neoadjuvant setting. Moreover, the pathological response definitions are unconventional. The authors classify CPR = 0% residual tumor and MPR < 50% residual tumor, which are unusually lenient. Established neoadjuvant criteria in solid tumors (including HCC) typically use ≤10–30% residual viable tumor as the cutoff for MPR. The rationale for adopting a less stringent threshold is not justified. Sensitivity analyses using widely accepted cutoffs should be provided, and the justification for the authors' chosen thresholds must be explicitly stated. These omissions limit the interpretability and clinical relevance of the reported findings. At minimum, the missing details should be provided in the Supplementary Information to allow readers to properly contextualize the results and assess their reproducibility in relation to existing neoadjuvant immunotherapy trials.

2. The authors' findings overlap extensively with previously published neoadjuvant anti–PD-1 translational studies, most notably the Nature Medicine study (PMID: 37322116) and the Molecular Cancer study (PMID: 40205519), which have already characterized the immunotherapy-associated tumor microenvironment, including T cell and macrophage states in HCC. Also, there are 5 similar published neoadjuvant IO trials in HCC that the authors should at least discuss and compare with.

To strengthen the manuscript, the authors should directly compare their clinical readout and translational results with landmark datasets, at minimum discussing the degree of similarity or divergence between their MacroLA cluster and the previously described macrophage populations, including phenotypic markers and proposed functions. Without such cross-comparison, it is difficult to assess what is truly novel in this study relative to prior work.

3. The methodology for tissue dissociation prior to single-cell RNA-Seq is not described. Dissociation procedures (enzymatic digestion, timing, temperature) can introduce artifacts, bias cell recovery, and alter gene expression signatures, and therefore need to be reported in sufficient detail. In addition, key sequencing quality metrics such as matrix characteristics (number of cells captured, median genes per cell, sequencing depth) and library saturation are not provided. These are essential for evaluating dataset robustness and reproducibility. The authors should include both dissociation details and standard scRNA-seq QC metrics, ideally in the Methods or Supplementary Information. Furthermore, it is unclear how the authors handled potential artifacts such as cell doublets or ambient RNA contamination ("RNA soup"), which can significantly affect clustering and downstream inferences. Beyond the standard CellRanger pipeline and typical cell filtering (e.g., mitochondrial read percentage, gene/cell thresholds), were additional steps taken (e.g., Scrublet, DoubletFinder, SoupX) to ensure data quality? Explicitly addressing these points would increase confidence in the robustness of the single-cell dataset.

4. The spatial transcriptomics data presented in the manuscript are obtained from an external dataset (PMID: 36708811; Mendeley skrx2fz79n). However, the manuscript provides little discussion on whether these data are directly comparable or transferrable to the current study cohort, given potential differences in sample processing, sequencing depth, and clinical context. The limitations of relying on a public dataset for validation, rather than generating in-house spatial data from the same patients, should be explicitly acknowledged. At minimum, the authors should clarify how differences in cohort characteristics and methodology might affect the interpretation and generalizability of their findings.

5. Macrophage annotation in the manuscript is insufficient. For example, PMID: 36708811 already identified five macrophage subtypes in HCC ($SPP1^+$, $FOLR2^+$, $CXCL10^+$, $CCL3L1^+$, and proliferating), and demonstrated that $SPP1^+$ macrophages form the tumor immune barrier (TIB) with CAFs, thereby restricting immunotherapy efficacy. The authors' MacroLA cluster appears to strongly overlap with this previously described $SPP1^+$ population: SPP1 is listed among the top five genes in Table S8, and line 499 introduces SPP1 in the context of ECM remodeling (together with FN1). Furthermore, ligand–receptor analysis (line 379) highlights fatty acid–enriched tumor cells interacting with TREM2 on MacroLA and SPP1–CD44 interactions, both of which are canonical features of $SPP1^+$/$TREM2^+$ LAM populations. Taken together, the data strongly suggest that the "MacroLA" described here is not a novel population but corresponds to the already reported

SPP1⁺ and/or TREM2⁺ macrophages. A direct cross-comparison to the established macrophage subtypes from prior atlases is essential to properly contextualize these findings and to clarify whether the current work adds incremental insight or represents a rediscovery.

6. “MacroLA” is not a commonly used term. The established terminology is lipid-associated macrophages (LAMs). The authors should adopt consistent nomenclature and explicitly align their cluster with prior SPP1⁺ LAM descriptions.

7. The manuscript inconsistently applies Monocle2 for some pseudotime analyses and Monocle3 for others, without providing an explanation or justification for this choice. This inconsistency is concerning, as different versions of the tool use distinct algorithms and assumptions that may influence trajectory inference. The authors should: Clearly state in the Methods section why Monocle2 was used in some cases while Monocle3 was applied in others. Demonstrate concordance between the pseudotime inferences from Monocle2 and Monocle3 to increase confidence in the robustness of the results. Consider complementing these analyses with RNA velocity, which can provide orthogonal support for directionality of differentiation, particularly in the macrophage and T cell trajectories central to the study’s conclusions.

8. The study employs pySCENIC for transcription factor (TF) activity inference and CellChat for cell–cell communication analysis. While both are widely used and appropriate for exploratory scRNA-seq analyses, the current manuscript at times presents these outputs as though they provide direct mechanistic evidence. These tools are largely predictive and hypothesis-generating, rather than definitive. To strengthen their claims, the authors should reframe these results as exploratory insights rather than conclusive mechanisms, consider supplementing their analysis with NicheNet, which is designed to integrate ligand–receptor interactions with downstream gene expression changes in target cells, thereby providing a stronger link between intercellular signaling and observed transcriptional states, and where possible, cross-validate predictions with orthogonal datasets (e.g., spatial transcriptomics, mIHC, or functional assays).

9. The functional validation using patient-derived organoid–T cell co-culture assays is insufficiently described and raises several interpretive concerns:
- It is not clear how many independent organoid lines and patients’ T cells were tested, what the take rate of organoid was, or whether these were derived from the same patients enrolled in the neoadjuvant PD-1 trial versus unrelated HCC patients with similar demographics. Patient demographics are not provided. This lack of detail prevents assessment of representativeness and clinical relevance.
- It is not specified whether the T cells used in co-culture were autologous to the organoids or derived from unrelated donors. Without this clarification, the observed organoid killing could be explained by allo-reactivity rather than bona fide tumor antigen–specific T cell responses. The absence of information on HLA matching further limits the interpretability of the findings. In addition, the frequency of truly tumor antigen–specific T cells within bulk PBMC is typically very low; unless the relevant antigens were identified and the corresponding T cell populations enriched, it is difficult to conclude that the observed cytotoxicity reflects genuine antigen-specific recognition. At minimum, these caveats should be explicitly acknowledged and discussed.
- The only T cell function reported is IFN-γ secretion. No assessment of T cell proliferation (e.g., CFSE dilution, Ki-67), expression of exhaustion markers (PD-1, TIM-3, LAG-3), Treg abundance (FOXP3), or effector molecules (granzyme B, perforin, TNF-α) is presented. Without these metrics, it is unclear whether the co-cultured T cells mirror the dysfunctional states identified in the scRNA-seq analysis.
- In the Methods, the authors state that “PMA induces M2 macrophages.” This is not accurate: PMA is generally used to differentiate monocytes into macrophages (M0 state), but does not drive M2 polarization. Canonical M2 polarization typically requires exposure to IL-4 and/or IL-13. The authors should clarify the actual polarization conditions used, and provide supporting evidence that M2 identity was achieved, for example by presenting marker expression data (e.g., CD206, MRC1, ARG1). Without such clarification, the functional interpretations of their macrophage co-culture assays remain uncertain.
- Only lycopene was tested, which is a nonspecific intervention. No targeted perturbations of the key pathways highlighted by the single-cell analyses (e.g., PPAR family signaling, SPP1, TREM2) were performed, limiting mechanistic insight.

Minor Points:
The authors should use the widely recognized abbreviation “LAM” (lipid-associated macrophages) rather than introducing “MacroLA,” unless a strong justification is provided.

Typographical errors: Several spelling errors should be corrected, including “demostrated” (lines 115, 431, 1095, 1100) and “Macropahge” (line 765).

Provide full accession identifiers for all datasets analyzed (e.g., GSE IDs, Mendeley DOIs, and related citations) clearly in the Methods section for transparency and reproducibility.

It is not clear whether the in-house single-cell dataset generated in this study will be deposited in a public repository (e.g., GEO, SRA, EGA). Public accessibility is essential for transparency, reproducibility, and re-use by the community, and should be clearly stated.

Version 1:

Reviewer comments:

Reviewer #1

(Remarks to the Author)
I appreciate the authors' efforts to address the previous comments; however, I still have a number of concerns. In particular, several issues arise from the newly added data and information. These comments are outlined in detail below:

1) Main text: In the text of results is now written: "To more precisely identify the cell types and corresponding subtypes of our single-cell dataset, we integrated 5 public scRNA-seq datasets, consist of 60 untreated HCC patients, thus resulting in a high-throughput single-cell atlas of 74 HCC patients (Supplementary Table 2)." This is not in line with the information in Supplementary Table 2. The number of patients does not match, etc. The explanation of which patients were included is missing, etc. The legend of the table is missing.

2) Authors newly added "To demonstrate that LAM and HCC_FA subsets are independent of hepatic steatosis in patients and associated with immunotherapy efficacy, we performed correlation analyses between the proportions of the LAM, HCC_FA subsets and patient BMI (Supplementary Fig. 6a). Neither subset showed a statistically significant correlation with BMI.". I find this argument unconvincing. BMI is an imprecise and indirect surrogate for hepatic steatosis and does not reliably reflect liver fat content, particularly in patients with cancer or advanced liver disease. Therefore, the lack of correlation between LAM or HCC_FA subsets and BMI is insufficient to support the claim that these subsets are independent of hepatic steatosis.

3) Figure 7: Panel Figure 7e, representative pictures of flow cytometry IC control or FMO control for FABP1 and APOA1 are missing. In graph 7g meaning of "NR" MPR" "pCR" is missing in the figure legend.

4) Statistical analysis are not correct for data in Figure 7. Unpaired student's t tests can be used if 2 groups are compared. Authors should either use ANOVA followed by Tukey's multiple-comparison test, if normality was proved. However, given the small sample size (n=5), authors should use Kruskal–Wallis test (non-parametric equivalent of one-way ANOVA). In addition, the graphs in figure 7 show only selected statistical comparisons, with "ns" reported inconsistently. All relevant pairwise comparisons should be displayed, and non-significant results should either be reported systematically or omitted entirely.

5) Supplementary table was not modified in the new submitted version, there is still the table without liver background etiology etc. (Previously comment 4)

6) Supplementary Fig. 7 – The authors have added multiparametric flow cytometric analyses of tumor-infiltrating lymphocytes; however, the presented data appear to contain inaccuracies. It is written that the axis y represents CD45-PE-Cy7 in all graphs, which does not seem to be correct. Moreover, representative pictures of flow cytometry IC control or FMO control need to be included for all conditions.

7) I could not find any publicly available clinical-trial registration or results for ClinicalTrials.gov identifier: NCT06571369

8) Newly added paragraphs for the method part contain inaccuracies. For instance, lines 982-986.

9) Line 1002-1003: "The survival status of HCC mice was observed daily until a fatal outcome for all animals." Why is this sentence added? The experiment was not designed as a survival study.

10) English should be carefully corrected in the whole manuscript and the supplementary part. For instance "In particular, we discover that the lipid-associated macrophages (LAM) characterized by C1QA, APOA1 and FABP1 expression, is closely related to immunosuppression among HCC patients." Modify : "discover" → "discovered" past tense; "is" → "are" the plural subject "macrophages"

11) Abbreviations need to be corrected. Tables and Supplementary tables should include legends where all Abbreviations used in the tables are explained. For instance, abbreviations "CNV", PR, PD, SD, MPR, CPR, etc.… Some abbreviations are introduced several times in the text, for instance, lipid-associated macrophage (LAM)

(Remarks on code availability)

Reviewer #2

(Remarks to the Author)
The authors have thoroughly addressed all comments.

(Remarks on code availability)
N/A

Reviewer #3

(Remarks to the Author)
Peng et al. have substantially revised and improved the manuscript in terms of both clarity and scientific rigor. In particular, the authors have addressed the major conceptual and technical concerns raised in the previous round of review. The additional analyses and methodological clarifications have strengthened the overall robustness of the study. However, there remain several areas where clarification, refinement, or minor additional adjustments would further improve the manuscript and enhance its interpretability. Below are specific comments related to individual points for the authors' consideration.

Related to point 1:
I appreciate the authors' efforts to expand the clinical description and provide inclusion/exclusion criteria and supplementary clinical tables. However, several clinically important elements remain insufficiently clarified. It would be ideal if these data could be provided, baseline tumor characteristics - including tumor stage, number of nodules, tumor size before and after treatment, presence of macro- or microvascular invasion, and other high-risk histopathological features. These can help better interpreting recurrence-free survival and pathological response outcomes. In addition, the units for RFS, OS, and residual tumor percentage should be explicitly specified. The rationale for administering 3–5 cycles of anti–PD-1 therapy prior to surgery remains unclear, and it is not evident whether treatment duration was standardized across patients or individualized. It would be ideal to list out in the method section about the authority approved treatment regimen. Finally, baseline and perioperative AFP measurements, and any associations with treatment response, should be reported if available. Inclusion of these details - at least within the Supplementary Information - would substantially improve the clinical transparency, reproducibility, and interpretability of the study in relation to existing neoadjuvant immunotherapy literature.

Since the authors have now provided RFS and OS data, these outcomes should be formally visualized using Kaplan–Meier survival analyses stratified by their pathological response classification. This would substantially improve the clinical interpretability of the study and allow readers to directly assess the prognostic relevance of the reported response categories.

While I understand that the authors have followed the 2024 Chinese clinical guidelines for hepatocellular carcinoma in defining MPR as >50% tumor regression, it would be important to additionally perform survival analyses using a more widely accepted pathological response cutoff (e.g., >70% tumor regression), as reported by D'Alessio et al. (PMID: 39437804). This additional Kaplan–Meier analysis could be presented in the Supplementary Materials if space is a concern. Including such a comparison would improve the global comparability of the study and enhance its relevance to the broader international neoadjuvant immunotherapy literature, while allowing the main analyses to remain aligned with the 2024 Chinese guideline classification.

The authors should explicitly cite the 2024 Chinese clinical guidelines for hepatocellular carcinoma in the main text when introducing their pathological response classification criteria. In addition, the corresponding definition (e.g., MPR defined as >50% tumor regression) should be clearly stated in the relevant table or figure legend, with the guideline properly referenced. This will ensure transparency of the classification framework and allow readers to clearly understand the rationale behind the chosen cutoff.

Related to point 2:
I thank the authors for their detailed and thoughtful response regarding the relationship of the current study to previously published neoadjuvant anti–PD-1 translational analyses in HCC, including the Nature Medicine (PMID: 37322116) and Molecular Cancer (PMID: 40205519) studies, as well as other recent neoadjuvant immunotherapy trials. The clarification that the present work addresses a complementary mechanistic layer - focusing on a myeloid-driven metabolic and fibrotic program rather than primarily on T cell instructional niches - is helpful and improves conceptual positioning. That said, I believe the manuscript would benefit from a more explicit and structured cross-comparison to prior datasets in order to clearly delineate what is novel versus confirmatory, or at minimum to discuss these similarities and distinctions more systematically in the Discussion section. While the authors provide a narrative differentiation in the letter of reply to reviewers, a clearer comparison within the manuscript itself, such as highlighting shared and divergent macrophage phenotypes, key marker overlap, and differences in mechanistic interpretation, would strengthen the transparency of the study's contribution. A concise comparative summary (either textual or tabulated in the Supplementary Information) outlining clinical endpoints, immune subsets identified, and methodological approaches across studies would further help readers contextualize the current findings within the rapidly evolving neoadjuvant HCC immunotherapy literature. Overall, the authors' argument for complementarity is reasonable, and the manuscript has improved substantially. Clarifying the degree of overlap and divergence with landmark studies would further enhance its clarity and perceived novelty.

Related to point 3:
I thank the authors for substantially expanding the Methods section to describe tissue dissociation procedures, sequencing workflow, and quality control metrics. The inclusion of digestion conditions, cell viability thresholds, sequencing depth, median genes per cell, UMI counts, and library saturation meaningfully improves transparency. The addition of DoubletFinder-based doublet detection is also appropriate and increases confidence in data quality. However, a few clarifications would further strengthen methodological rigor:

1. While the digestion temperature and enzyme (type I collagenase) are now described, the duration of digestion is not explicitly stated and if the digestion time was standardized across samples.

2. an additional supplementary table showing sample-level QC would be helpful, for example, cells captured per sample, median genes and UMIs per sample, percentage of mitochondrial reads, and doublet rate per sample.

3. is any batch correction method used?

Overall, the methodological reporting has improved significantly and is now much more transparent. Addressing the above points would further strengthen the robustness and reproducibility of the single-cell dataset.

Related to point 4:
I thank the authors for their detailed response and, importantly, for generating additional in-house spatial transcriptomic (ST) data paired with their single-cell cohort. The addition of six paired spatial transcriptomic samples substantially strengthens the manuscript and meaningfully addresses the concern regarding reliance on external public datasets. The integration of spatial data directly from the same clinical cohort provides much stronger support for the reported enrichment of LAMs in non-responders.

Related to point 5:
I thank the authors for the substantial additional analyses performed in response to this comment. The integrated cross-dataset comparison using the Liu et al. (PMID: 36708811) HCC immunotherapy scRNA-seq dataset, the Harmony-based batch correction, and the side-by-side UMAP visualization of predefined SPP1⁺ macrophages and the LAM cluster significantly strengthen the manuscript. The updated gene ranking criteria and the new figure comparing canonical SPP1⁺/TREM2⁺ features (SPP1, TREM2) with lipid-associated genes (FABP1, APOA1, APOC2, APOB) provide a more objective basis for distinguishing the populations.

Related to point 6:
I thank the authors for adopting the established terminology "lipid-associated macrophages (LAMs)" throughout the revised manuscript. This improves clarity and aligns the study with current conventions in the field. The authors also clarify the relationship between their identified LAM population and previously described SPP1⁺ LAM subsets. The explanation that their LAMs share certain canonical tissue-remodeling markers (e.g., SPP1) but are additionally characterized by prominent lipid metabolic features (FABP1, APOA1, APOC1, etc.) is helpful and consistent with the expanded comparative analyses provided in response to Comment 5. That said, I recommend slightly refining the language in the manuscript to avoid over-fragmenting macrophage nomenclature. Given the partial overlap in marker expression and functional programs, it may be more accurate to describe the identified LAMs as a metabolically enriched LAM state within the broader lipid-associated macrophage spectrum, rather than implying a completely separate macrophage category. Careful framing will help ensure that the study is perceived as extending and functionally refining existing LAM concepts rather than redefining terminology. Overall, the nomenclature issue has been appropriately addressed. Minor clarification in the Discussion to position this LAM subset within the evolving macrophage-state landscape would further enhance conceptual coherence.

Related to point 7:
I thank the authors for the detailed clarification regarding the use of Monocle2 and Monocle3. The explanation that both tools were applied to the same cell populations and used as complementary validation frameworks—rather than to generate independent biological claims—is helpful and resolves the initial concern about potential methodological inconsistency. The addition of the new figure directly comparing trajectory inferences from Monocle2 and Monocle3 substantially strengthens confidence in the robustness of the inferred state transitions. The rationale for prioritizing Monocle2 for primary visualization and using Monocle3 as algorithmic confirmation is reasonable. Demonstrating concordant lineage ordering across DDRTree- and graph-based approaches meaningfully increases methodological confidence. Regarding RNA velocity, the authors provide a reasonable explanation for not applying it, particularly considering potential variability in RNA integrity from post-treatment surgical specimens. This justification is acceptable. That said, it would be helpful if the manuscript explicitly states that trajectory directionality is inferred based on pseudotime structure rather than independently validated by splicing kinetics, to avoid overinterpretation of directional claims.

Related to point 8:
I thank the authors for their thoughtful response and for revising the manuscript to more appropriately frame pySCENIC and CellChat analyses as hypothesis-generating rather than definitive mechanistic evidence. The revised wording improves interpretive rigor and aligns with the exploratory nature of these computational tools. The addition of NicheNet analysis represents a meaningful strengthening of the study. Integrating ligand–receptor interactions with downstream target gene programs provides a more mechanistically coherent bridge between predicted intercellular signaling and observed transcriptional states. The concordance between CellChat and NicheNet interaction trends further increases confidence in the robustness of the communication patterns described. The inclusion of paired spatial transcriptomics and the murine intervention experiments targeting lipid metabolic pathways (e.g., high-fat diet, lycopene, pan-PPAR agonist) also provides important orthogonal support that moves the study beyond purely computational inference.

Related to point 9:
I thank the authors for the extensive clarifications and additional experimental data provided in response to this comment. The revised manuscript substantially improves the transparency and functional depth of the validation studies. The clarification that six independent patient-derived HCC organoid (HCO) lines were generated, with a reported take rate of approximately 60%, and that autologous PBMC-derived T cells were used for co-culture, meaningfully strengthens the methodological clarity. The inclusion of patient clinical information in Supplementary Table 2 is also appreciated. The explicit acknowledgment that the HCO patients had not received prior immunotherapy appropriately avoids confounding treatment-related immune alterations. The authors' explanation regarding the use of PBMC-derived T cells based on prior "clonal expansion/revival" frameworks is reasonable. Importantly, they now acknowledge the limitation that the frequency of tumor-reactive T cells within bulk PBMC is likely low. This caveat should remain clearly stated in the manuscript. The

organoid co-culture system should be framed as an assay assessing immunomodulatory potential rather than definitive evidence of antigen-specific cytotoxicity. The addition of the murine HCC model with high-fat diet (HFD), lycopene, and pan-PPAR agonist (chiglitazar) interventions significantly strengthens the functional component of the study. The multi-parameter flow cytometric analysis demonstrating alterations in: Effector cytokine production (TNF, IFN-γ) Proliferative capacity (Ki-67) Exhaustion markers (PD-1, TIGIT) Regulatory T cell abundance provides a much more comprehensive assessment of T cell dysfunction associated with LAM enrichment. This in vivo validation meaningfully supports the link between lipid metabolic programs and impaired antitumor immunity. The correction of the PMA polarization description is also appropriate. The clarified two-step protocol (PMA-induced differentiation followed by IL-4/IL-13 polarization) and supporting CD206 expression data adequately address the earlier methodological inaccuracy. That said, several interpretive boundaries should remain clearly defined: While autologous pairing excludes allo-reactivity, antigen specificity was not directly demonstrated. This limitation should be explicitly acknowledged. The murine HFD model modulates systemic lipid metabolism rather than macrophage-specific pathways. Therefore, while the data strongly support a lipid-driven immunosuppressive axis, they do not fully isolate LAM-intrinsic mechanisms. Although PPAR modulation was incorporated, direct perturbation of key molecules highlighted in the single-cell analyses (e.g., SPP1 or TREM2) was not performed. The Discussion should carefully distinguish between pathway-level validation and target-specific mechanistic proof. Overall, the functional validation arm has been substantially strengthened compared to the initial submission and now provides meaningful biological support for the central hypothesis. With careful framing of the remaining limitations, this section is sufficiently robust.

Related to minor points:
The authors have comprehensively addressed all minor comments raised in the previous review round.

Comments on Revised Figures and Corresponding Text
- Related to revised Fig 1 and corresponding text
In the abstract, revised Fig 1, and corresponding text, the authors should explicitly state that the 60 public-domain cases are all treatment-naïve, whereas the authors' own cohort consists of patients treated with neoadjuvant immunotherapy. This distinction is essential for appropriate interpretation, as baseline (treatment-naïve) TIME features are not directly equivalent to post-treatment specimens. Explicitly highlighting this difference will improve transparency and contextual accuracy.

- Related to revised Fig 2 and corresponding text
Given that the authors have now generated paired in-house spatial transcriptomics data, these data should be prioritized in the main figure (e.g., Fig 2i–j), while the public-domain spatial dataset could be moved to the Supplementary Information. The in-house paired data provide stronger internal validation and are more directly aligned with the study cohort, and therefore should take precedence in the main narrative.

- Related to revised Fig 5 and corresponding text
In Fig 5e, mIHC staining is presented; however, it is difficult to clearly visualize APOA1$^+$ CD68$^+$ macrophages in tissues where APOA1$^+$ tumor cells are also abundant. It would strengthen the figure if the authors: a) Select one or two representative regions and provide zoomed-in/enlarged panels highlighting APOA1$^+$ CD68$^+$ double-positive cells. B) Provide single-channel images in the Supplementary Information to allow clearer assessment of co-localization. A similar improvement would be beneficial for the mIHC images presented in Fig 6c.

- Related to revised Fig 6 and corresponding text
In Fig 6b, spatial data from patients 3 and 11 are shown as representatives, but their responder/non-responder status is not labeled. In contrast, Fig 6c uses different representative cases (patients 13 and 10). For consistency and clarity, the same representative patients should ideally be used across the main panels. All remaining patient images can be presented in the Supplementary Information. In addition, Fig 6k would be more logically placed immediately after LAM characterization (i.e., after Fig 6f and before the co-culture experiments), to improve narrative flow.

- Related to revised Fig 7 and corresponding text
The inclusion of in vivo experiments is commendable and substantially strengthens the manuscript. However, several clarifications and revisions are needed:

a. Fig 7a (schematic): The liver cartoon appears to depict human anatomy rather than mouse liver. This should be corrected for anatomical accuracy.

b. Fig 7b–c (bioluminescence and tumor weight): Fig 7b shows representative bioluminescence images. If available, quantitative bioluminescence signal measurements should be presented alongside tumor weight data in Fig 7c, with statistical comparisons between groups.

c. Fig 7d (Oil Red O staining): The methodology for Oil Red O staining quantification is not described. The authors should clarify:
How staining was performed,
How the positive area was quantified,
Whether quantification was automated or manual, and
How normalization was conducted.

d. Fig 7e (LAM gating and denominator clarification): The denominator for LAM percentage needs clarification. Specifically: Is LAM frequency calculated as % of CD45$^+$ F4/80$^+$ CD11b$^+$ C1Q$^+$ macrophages? Or as % of CD45$^+$ cells?

If the denominator is macrophage-gated, then the statistic reflects only co-expression (FABP1⁺ APOA1⁺ within macrophages), not overall LAM abundance. It would be equally important to show absolute numbers or percentage relative to CD45⁺ cells to determine whether total LAM abundance changes across conditions.

Furthermore, the gating suggests a relatively high proportion of CD11b⁻ F4/80⁺ cells (Kupffer-like cells) compared to CD11b⁺ F4/80⁺ monocyte-derived macrophages. This is unexpected in many HCC models, where monocyte-derived macrophages typically predominate. Therefore, the manuscript must clearly describe how mouse tissues were processed: Were tumor nodules microdissected prior to digestion? Or was whole liver homogenized for single-cell suspension? If whole liver was processed, interpretation of tumor-infiltrating macrophages may be confounded by resident Kupffer cells. Currently, there is insufficient description of mouse tissue processing and single-cell preparation in the revised Methods.

e. Fig 7f (T cell flow cytometry): The y-axis labels are unclear and should be clarified. Supplementary Fig 7 shows gating strategy but appears to focus primarily on CD4⁺ CD3⁺ cells. CD8⁺ T cells are central to cytotoxic function and should be explicitly analyzed. The manuscript does not clearly state whether "T cells" refer to CD4⁺, CD8⁺, or total CD3⁺ cells. If CD8 staining was not included, CD4⁻ CD3⁺ could serve as a surrogate for CD8⁺ T cells, although direct CD8 staining would be preferable.

f. Fig 7h-i (Interpretation of chiglitazar effects):
Chiglitazar (a pan-PPAR agonist) appears to increase PPAR expression in mouse liver. However, as this is a systemic drug, it likely affects hepatocytes, tumor cells, and immune cells. Since tumor lipid deposition is also reduced, the authors should avoid directly attributing the effect solely to LAM modulation. The conclusions should be phrased more cautiously as associations unless PPAR expression/activity was specifically measured in LAMs (e.g., via gated FACS analysis or sorted populations). Minor point: gene symbols should be italicized throughout.

Overall, the authors have made substantial revisions in response to the previous round of comments, including expanded clinical characterization, strengthened cross-dataset benchmarking, clarified macrophage annotation and nomenclature, improved methodological transparency for single-cell and spatial analyses, and added meaningful in vivo functional validation experiments. The major conceptual and technical concerns raised previously have been largely addressed. However, several remaining issues require clarification and refinement before the manuscript can be considered for acceptance. While these points are relatively minor in scope and primarily relate to presentation, methodological transparency, and figure organization, they are important for ensuring clarity, rigor, and interpretability. I therefore recommend minor revision, with the expectation that the authors address the remaining comments comprehensively prior to final consideration.

(Remarks on code availability)
I regret that I am not able to evaluate or review the provided code in detail. However, I note that the code has been made publicly accessible, which is appropriate for transparency and reproducibility.

Version 2:

Reviewer comments:

Reviewer #1

(Remarks to the Author)

The authors have substantially revised and improved the manuscript. Most of the previous concerns have been adequately addressed, particularly comments 1–3, 5, 7-11, and I thank the authors for their detailed and thoughtful responses.

However, I still have some minor concerns regarding comments 4 and 6, which were not fully resolved in the previous round of review. Additional clarification on these points would further enhance the interpretability of data.

Concerning comment 4, authors stated in their response that "We now present a standardized set of pre-specified pairwise comparisons in Fig. 7c–f and Fig. 7i (HFD vs HFD+LYC; HFD vs HFD+CHI; HFD+CHI vs HFD+LYC; RD vs HFD+LYC; RD vs HFD+CHI) to directly address the biological hypotheses of diet-induced immunotherapy resistance and corresponding metabolic interventions. Meanwhile, all of the remaining pairwise comparisons, including non-significant results, are provided systematically in corresponding tables of Source data to ensure transparency while maintaining figure readability. " If this were fully the case, I would not have further concerns. However, many of the specified comparisons are still missing in the figure. For example, the comparison between HFD+CHI vs HFD+LYC is not shown in Fig. 7i, and comparisons such as RD vs HFD+LYC seem to be absent in many graphs in Fig. 7c–f and Fig. 7i as well. Similarly, RD vs HFD+CHI does not appear to be included in Fig. 7i. These omissions make it difficult to fully interpret the data. In addition, I was unable to locate the referenced "Source Data tables" containing the remaining pairwise comparisons. It would be helpful if the authors could clarify it.

Concerning my comment 6. I previously wrote the following comment (6. Supplementary Fig. 7 – The authors have added multiparametric flow cytometric analyses of tumor-infiltrating lymphocytes; however, the presented data appear to contain inaccuracies. It is written that the axis y represents CD45-PE-Cy7 in all graphs, which does not seem to be correct.) This comment was not completely answered. The issue is that the plots of Supplementary Figure 7 d-f are clearly not gated on the same parent population, even though the caption claims they are. The axis shows CD45-PE-Cy7, but the distribution

and density of events are clearly different from panels d to f. If the same population had been used, only the marker on the other axis (for instance, TNF or TIGIT) would change, not the entire population. But here, the mean fluorescence intensity and overall distribution along the CD45 axis are not consistent between d, e and f panels. Therefore, the data seem to be derived from different upstream gating strategies, not from the same population. Please clarify it.

(Remarks on code availability)

Reviewer #3

(Remarks to the Author)
I thank the authors for their detailed and comprehensive responses, as well as for the substantial revisions made to the manuscript. The additional analyses, clarifications, and newly incorporated data have significantly improved the scientific rigor, transparency, and overall clarity of the study.

Below I provide a brief evaluation of the authors' responses to the major points:

Related to Point 1 (Clinical annotation and survival analyses)
The authors have satisfactorily addressed the concerns. The inclusion of expanded clinical characteristics in Supplementary Table 1, clarification of units, and the addition of Kaplan–Meier analyses using both >50% and >70% cutoffs are appropriate and significantly enhance the clinical interpretability of the study. The citation of the 2024 Chinese clinical guidelines is now clearly stated. Overall, this point is adequately resolved. For the newly added Fig. 1e, the authors may consider expanding the figure legend to clearly define the response groups (e.g., non-responders [non-MPR] and responders [MPR + CPR]) to improve clarity. In addition, in the updated Supplementary Table 1, "RECIST" should be specified as "RECIST 1.1."

The authors should briefly discuss the clinical implications of the lack of separation in RFS and OS observed in the current cohort under Discussion section. In particular, this finding suggests that pathological response, as defined in this study, may not directly translate into measurable survival benefit within the available follow-up period. It would therefore be important for the authors to clearly position the study as primarily aiming to characterise the tumour microenvironment and pathological response to neoadjuvant immunotherapy, rather than to demonstrate treatment efficacy at the clinical outcome level. In this context, the current title may be misleading, as it implies that lipid-associated macrophages (LAMs) impair therapeutic efficacy. Given that no corresponding difference in RFS or OS is observed, such a causal or outcome-linked interpretation is not fully supported by the data. I would therefore recommend revising the title to adopt a more cautious, association-based framing that better reflects the scope and strength of the findings.

Related to Point 2 (Cross-study comparison and positioning)
The addition of a structured comparison in the Discussion and Supplementary Table 13 meaningfully improves contextualisation relative to prior studies. While some narrative elements could still be slightly streamlined for clarity, the current revision is acceptable. This point is largely resolved.

Related to Point 3 (scRNA-seq methodology and QC)
The authors have adequately clarified digestion duration, batch correction strategy, and provided sample-level QC metrics in Supplementary Table 14. These additions sufficiently address concerns regarding data robustness and reproducibility. This point is resolved.
Related to Point 5–6 (Macrophage annotation and nomenclature)
The additional cross-dataset integration and refined framing of LAMs as a metabolically enriched state within the broader macrophage spectrum are appropriate. The revised positioning avoids over-fragmentation of nomenclature and is now conceptually sound. These points are satisfactorily addressed.

Related to Point 7 (Pseudotime analysis)
The clarification regarding Monocle2/3 usage and the explicit statement on pseudotime-based directionality are appropriate. The addition of comparative analyses supports robustness. This point is resolved.

Related to Point 8 (Computational inference tools)
The authors have appropriately revised the language to reflect the exploratory nature of pySCENIC and CellChat analyses and have strengthened the study with NicheNet and orthogonal validations.
This point is well addressed.

Related to Point 9 (Functional validation)
The authors have substantially improved the clarity and depth of functional validation, including better description of organoid systems, appropriate acknowledgment of limitations, and strengthened in vivo data. The revised Discussion now appropriately delineates interpretive boundaries.
This point is satisfactorily addressed.

Figure-related and minor points
The authors have generally responded well to the figure-related suggestions, including improved visualisation, clearer gating strategies, and additional methodological details. Most concerns have now been adequately addressed.

Overall comments

Based on the revised manuscript and the clarified in vivo methodology, the data suggest that LAM enrichment may arise, at least in part, as a downstream consequence of lipid metabolism–active tumour states (e.g., $APOA2^+$/$APOC3^+$ HCC), as indicated in Fig. 2i–j. In the in vivo experiments, systemic modulation of lipid metabolism (e.g., via chiglitazar or lycopene) may therefore reduce LAM abundance indirectly through effects on tumour metabolic activity, rather than through direct targeting of macrophage-intrinsic pathways.

In this context, the current title—"Integrated Single-cell Multi-omics Characterization Reveals Lipid-associated Macrophages Impairing Neoadjuvant Immunotherapy Efficacy in Hepatocellular Carcinoma"—may overstate both the directionality and causality of the findings. The data presented do not clearly establish that LAMs are primary drivers of therapeutic resistance; rather, they may reflect a tumour-driven metabolic microenvironment associated with non-response. I would therefore recommend that the authors revise the title to adopt a more cautious, association-based framing and to avoid implying a direct causal role of LAMs in impairing treatment efficacy. Such a revision would also better align with the absence of clear survival differences observed in the cohort.

Final assessment
Overall, the authors have made substantial revisions and have addressed the major conceptual and technical concerns raised in the previous review. The remaining issues are relatively minor and primarily relate to presentation clarity, figure organisation, and language refinement. Provided these points are carefully addressed, the manuscript would be suitable for acceptance. I therefore recommend minor revision, with the expectation that the remaining comments are adequately incorporated.

(Remarks on code availability)

Version 3:

Reviewer comments:

Reviewer #1

(Remarks to the Author)
All remarks are satisfactorily addressed.

(Remarks on code availability)
All remarks are satisfactorily addressed.

Reviewer #3

(Remarks to the Author)
Peng et al. have substantially revised and improved the manuscript in terms of both clarity and scientific rigor. In particular, the authors have addressed the major conceptual and technical concerns raised in the previous rounds of review. The additional analyses, methodological clarifications, expanded clinical annotations, cross-dataset benchmarking, improved spatial validation, and strengthened in vivo functional studies have significantly enhanced the robustness and interpretability of the study.

Regarding the clinical analyses, the authors have now appropriately acknowledged in the Discussion that pathological response in the current cohort does not clearly translate into measurable RFS or OS benefit within the available follow-up period. This clarification helps appropriately position the study as primarily focused on characterization of the tumor immune microenvironment and pathological response associated with neoadjuvant immunotherapy, rather than direct demonstration of treatment efficacy at the survival outcome level.

The revised title is also improved compared with the previous version and appropriately avoids directly claiming impairment of therapeutic efficacy. However, the current wording — "Integrated Single-cell Multi-omics Characterization Reveals Lipid-associated Macrophages Mediated Immunosuppression in Neoadjuvant Immunotherapy of Hepatocellular Carcinoma" — remains grammatically awkward and could be further refined for clarity and readability. In particular, the phrase "Lipid-associated Macrophages Mediated Immunosuppression" would benefit from grammatical restructuring (e.g., "Lipid-associated Macrophage–Mediated Immunosuppression" or equivalent association-based phrasing). The latter part of the title could also be simplified to improve readability and precision.

Overall, the authors have now satisfactorily addressed the major conceptual and technical concerns raised previously. The remaining issue primarily relate to title refinement, language polishing, presentation clarity, and overall manuscript readability. I therefore support acceptance of the manuscript pending editorial refinement. In my opinion, an additional full round of external review is not necessary once minor issues are addressed.

(Remarks on code availability)

Open Access This Peer Review File is licensed under a Creative Commons Attribution 4.0 International License, which permits use, sharing, adaptation, distribution and reproduction in any medium or format, as long as you give appropriate credit to the original author(s) and the source, provide a link to the Creative Commons license, and indicate if changes were made.
In cases where reviewers are anonymous, credit should be given to 'Anonymous Referee' and the source.
The images or other third party material in this Peer Review File are included in the article's Creative Commons license, unless indicated otherwise in a credit line to the material. If material is not included in the article's Creative Commons license and your intended use is not permitted by statutory regulation or exceeds the permitted use, you will need to obtain permission directly from the copyright holder.
To view a copy of this license, visit https://creativecommons.org/licenses/by/4.0/

Reviewers' comments:

Reviewer #1:

1.Peng et al performed single-cell analysis of tumor tissue of patients who were previously treated by PD-1 (Tislelizumab or Camrelizumab). I agree that there have been relatively few studies dissecting the TIME (tumor immune microenvironment) in patients undergoing immunotherapy using single-cell sequencing, and I appreciate the effort made. However, the tissue was collected only after the PD-1 treatment. Therefore, there is no comparison with the situation before the treatment. Given the well-documented heterogeneity of HCC, it is challenging to draw conclusions about predictive TIME factors for immunotherapy response based solely on post-treatment samples.

Author's response: We thank the reviewer for this insightful comment. We fully acknowledge that our study is based on tumor specimens collected after PD-1 inhibitor treatment of HCC patients, and therefore does not include paired pre-treatment samples for scRNA-seq and direct temporal comparison. Given the well-known heterogeneity of HCC, this is indeed a limitation when attempting to infer predictive TIME features and we have added corresponding discussion about this issue (line 669 to 672).

It is important to clarify, however, that the primary aim of our study was not to analyze pre- and post-treatment dynamics within the same patient, but rather to investigate the inter-patient heterogeneity between responders and non-responders following neoadjuvant PD-1 blockade. Our focus is to understand how the tumor immune microenvironment differs across clinical response categories, which does not require paired sampling but does require high-resolution profiling of post-treatment tumor beds from both groups.

From a clinical standpoint, obtaining pre-treatment tumor biopsies is not routine practice in China, as current clinical guidelines allow HCC diagnosis based on AFP levels and characteristic imaging findings and do not recommend additional biopsy when diagnostic confidence is adequate (Zhou, et al.). Therefore, paired sample collection is generally not feasible. Although a few studies from the United States (Ma, et al.) and Korea (Lim, et al.) have reported single-cell analyses of biopsy samples, these are exceptions rather than standard practice in our setting.

Despite this constraint, using post-treatment samples to dissect the immunotherapy-shaped tumor immune microenvironment is a widely accepted strategy in immuno-oncology research. Notably, a recent lung cancer immunotherapy study (Liu, et al.) also relied primarily on post-treatment samples for single-cell multi-omics characterization. Following this established precedent, our study provides detailed insights into the treatment-shaped immune landscape associated with response status.

Reference

Zhou J, Sun H, Wang Z, Cong W, Zeng M, et al. China Liver Cancer Guidelines for the Diagnosis and Treatment of Hepatocellular Carcinoma (2024 Edition). Liver Cancer. 2025 Jul 10.

Ma L, Wang L, Khatib SA, Chang CW, Heinrich S, Dominguez DA, Forgues M, Candia J, Hernandez MO, Kelly M, Zhao Y, Tran B, Hernandez JM, Davis JL, Kleiner DE, Wood BJ, Greten TF, Wang XW. Single-cell atlas of tumor cell evolution in response to therapy in hepatocellular carcinoma and intrahepatic cholangiocarcinoma. J Hepatol. 2021 Dec;75(6):1397-1408.

Lim J, Goh MJ, Song BG, Sinn DH, Kang W, Gwak GY, Choi MS, Lee JH, Cha DI, Gu K, Ha SY, Hwang I, Park WY, Paik YH. Unraveling the immune-activated tumor microenvironment correlated with clinical response to atezolizumab plus bevacizumab in advanced HCC. JHEP Rep. 2024 Dec 15;7(4):101304.

Liu Z, Yang Z, Wu J, Zhang W, Sun Y, Zhang C, Bai G, Yang L, Fan H, Chen Y, Zhang L, Jiang B, Liu X, Ma X, Tang W, Liu C, Qu Y, Yan L, Zhao D, Wu Y, He S, Xu L, Peng L, Chen X, Zhou B, Zhao L, Zhao Z, Tan F, Zhang W, Yi D, Li X, Gao Q, Zhang G, Wang Y, Yang M, Fu H, Guo Y, Hu X, Cai Q, Qi L, Bo Y, Peng H, Tian Z, She Y, Zou C, Zhu L, Cheng S, Zhang Y, Zhong W, Chen C, Gao S, Zhang Z. A single-cell atlas reveals immune heterogeneity in anti-PD-1-treated non-small cell lung cancer. Cell. 2025 May 29;188(11):3081-3096.e19.

2.The study rather details the picture about TIME that appears as a consequence of the treatment in responding or non-responding patients. In other words, it would be more accurate to describe the data as reflecting TIME components that result from the efficacy or inefficacy of PD-1 blockade, rather than presenting them as TIME components that influence clinical efficacy, as currently stated in the manuscript (line 326).

Author's response: We thank the reviewer for this important and insightful comment. We agree that, given our study design based on post-treatment surgical specimens, the data primarily describe the TIME landscape that emerges as a consequence of effective or ineffective PD-1 blockade, rather than definitively identifying TIME components that determine clinical efficacy. We appreciate this distinction and have revised the manuscript accordingly. In particular, the statement in line 330 to 331 has been provided to clarify that our findings reflect immune cell states associated with treatment response without implying causality, and now present as: "*To comprehensively evaluate the TIME components that associated with clinical efficacy in our HCC immunotherapy cohort, ……*"

To address this limitation, and to further explore whether the TIME features observed in patients with different therapeutic outcomes may actively drive differential immunotherapy efficacy rather than merely reflect its consequences, we designed additional *in vivo* experiments focusing on lipid-associated macrophages (LAMs) as the most prominent immune component identified in our study. In this model, mice were stratified by high-fat diet (HFD) versus control diet to induce differential accumulation of lipid-associated macrophages (LAMs), following dietary conditioning, orthotopic liver tumors were established and mice were treated with PD-1 antibody to evaluate treatment efficacy. These experiments demonstrated that HFD-induced enrichment of LAMs causally impairs antitumor immune activity and reduces the efficacy of PD-1 therapy, indicating that LAMs can function as a causal factor contributing to poorer immunotherapy outcomes rather than merely a consequence of treatment. These results are presented in Fig.7 and below.

At the same time, we would like to emphasize that, even though only post-treatment samples were available, the major effector populations in antitumor immunity, T cells and macrophages often manifest functional states that are closely linked to therapeutic outcomes (DeNardo, et al.), (Jiang, et al.). Numerous studies have shown that the activation, exhaustion, differentiation, or polarization patterns of these cells can still provide meaningful indications of treatment efficacy, even when assessed after therapy (Liu, et al.), (Li, et al.). Therefore, the response-related immune signatures identified in our study, especially within T cell and macrophage compartments, may still offer biologically relevant clues regarding the mechanisms underlying successful or failed PD-1 blockade.

Nonetheless, we fully acknowledge that causal inference at the patient level cannot be established without paired pre-treatment samples. We have strengthened the discussion (line 669 to 672) to reflect this limitation. Importantly, we also highlight that, to overcome this constraint, we performed complementary animal model experiments targeting key immune regulatory components identified in the single-cell analysis. These *in vivo* studies provide additional mechanistic support and help to clarify the functional role of LAM-centered immunoregulatory programs in shaping the response to immunotherapy in HCC.

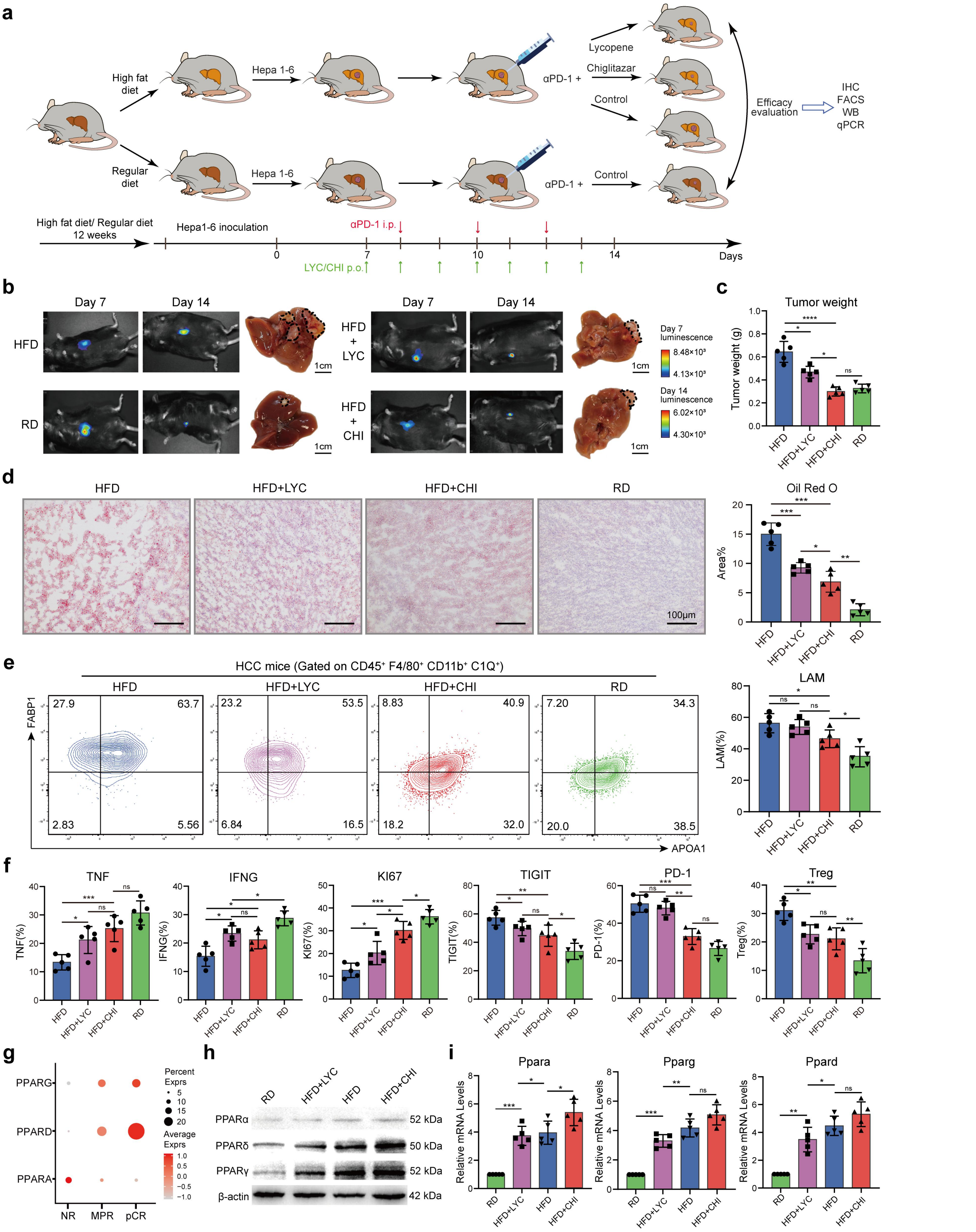

High-fat diet facilitates LAM-mediated immunosuppression in HCC and further regulated by lycopene and chiglitazar. High-fat diet facilitates LAM-mediated immunosuppression in HCC and further regulated by lycopene and chiglitazar. **a** The HCC mice received αPD-1 immunotherapy were randomly assigned to four experimental groups: mice with regular diet (RD), mice with high fat diet (HFD), mice with HFD and lycopene (LYC), mice with HFD and chiglitazar (CHI) (N=5). Subsequently, immunohistochemistry, flow cytometry, WB and qPCR were conducted to explore the features and mechanisms of LAM immunosuppressive function. **b** Representative in vivo bioluminescence images and tumor image of the growth of mice HCC. **c** Tumor weight of HCC mice (N=5). **d** Representative Oil Red O staining of tumor regions showing lipid accumulation degree of each group (N=5). **e** Tumor infiltrating immune cells were isolated from mouse HCC. The percentage of lipid-associated macrophage (LAM) was analyzed by flow cytometry (N=5). **f** Flow cytometric analysis of intratumoral T cell function, including effector cytokine production (TNF and IFN-γ), proliferative capacity (Ki-67) and expression of exhaustion markers (PD-1 and TIGIT) and proportion of Treg

cells (N=5). **g** Bubble plot showing the expression level of PPAR-related genes in LAMs from our HCC immunotherapy scRNA-seq cohort. **h** The protein expression levels of PPARα, PPARδ and PPARγ in mouse HCC tissue were measured by western blotting. **i** The relative expression levels of Ppara, Ppard and Pparg genes were measured by qPCR in mouse HCC tissue (N=5). (Unpaired student's t tests, $^{*}P < 0.05$, $^{**}P < 0.01$, $^{***}P < 0.001$, $^{****}P < 0.0001$, ns: not significant)

Reference:
DeNardo DG, Ruffell B. Macrophages as regulators of tumour immunity and immunotherapy. Nat Rev Immunol. 2019 Jun;19(6):369-382.
Jiang, P., Gu, S., Pan, D. et al. Signatures of T cell dysfunction and exclusion predict cancer immunotherapy response. Nat Med 24, 1550–1558 (2018)
Liu Z, Yang Z, Wu J, Zhang W, Sun Y, Zhang C, Bai G, Yang L, Fan H, Chen Y, Zhang L, Jiang B, Liu X, Ma X, Tang W, Liu C, Qu Y, Yan L, Zhao D, Wu Y, He S, Xu L, Peng L, Chen X, Zhou B, Zhao L, Zhao Z, Tan F, Zhang W, Yi D, Li X, Gao Q, Zhang G, Wang Y, Yang M, Fu H, Guo Y, Hu X, Cai Q, Qi L, Bo Y, Peng H, Tian Z, She Y, Zou C, Zhu L, Cheng S, Zhang Y, Zhong W, Chen C, Gao S, Zhang Z. A single-cell atlas reveals immune heterogeneity in anti-PD-1-treated non-small cell lung cancer. Cell. 2025 May 29;188(11):3081-3096.e19.
Li Y, Li F, Xu L, Shi X, Xue H, Liu J, Bai S, Wu Y, Yang Z, Xue F, Xia Y, Dong H, Shen F, Wang K. Single cell analyses reveal the PD-1 blockade response-related immune features in hepatocellular carcinoma. Theranostics. 2024 Jun 1;14(9):3526-3547.

3.The authors observed that a lipid-associated macrophage subtype—characterized by expression of C1QA, APOA1, and FABP1—was more frequently present in the TIME of patients who showed a poor response to PD-1 blockade. Notably, APOA1 and FABP1 are typically expressed by hepatocytes, while C1QA is a marker of liver-resident macrophages. This raises the possibility that these markers may not reflect a true macrophage subtype, but rather result from contamination, originating from lysed cells and fat vesicles that were phagocytosed by macrophages—artificially inflating the expression of these genes in transcriptomic profiles (https://doi.org/10.1182/blood.2023020597) . In vitro studies (Figure 6) focused on APOA1 only, not on FABP1. Why was FABP1 excluded from the confirmation experiments?

Author's response: We fully appreciate the reviewer's concern regarding the possibility that the expression of C1QA, APOA1, and FABP1 in the identified lipid-associated macrophage (LAM) population could arise from technical artifacts rather than representing a bona fide macrophage subtype. As correctly pointed out by the reviewer and discussed in the referenced study (Hume, et al.), scRNA-seq is inherently susceptible to confounding factors such as cell doublets and ambient RNA contamination, for instance in liver tissue where hepatocytes are abundant and prone to lysis. These issues may lead to apparent detection of hepatocyte-associated transcripts within macrophage clusters if not carefully validated.
Importantly, the study cited by the reviewer (Hume, et al.) explicitly addressed these concerns by proposing a validation framework to distinguish true macrophage-intrinsic expression from contamination artifacts. This framework emphasizes orthogonal experimental validation which most notably flow cytometry-based lineage gating supported by multiplex immunohistochemistry (mIHC) and computational doublet detection, to confirm the cellular origin of such lipid-associated signatures.
Following this established strategy, we performed both experimental and bioinformatic validations to rigorously assess the authenticity of the LAM population identified in our dataset. For flow cytometric validation, we have expanded our experimental validation using an independent, newly enrolled neoadjuvant immunotherapy cohort (R=5, NR=5), which was specifically collected for additional flow cytometric analyses. Hierarchical gating strategy was applied to unambiguously define macrophages. After excluding debris and doublets and gating on live cells, we sequentially gated on $CD45^{+}$ $CD68^{+}$ $C1Q^{+}$ cells to identify macrophages, and subsequently examined APOA1 and FABP1 expression within this population. Using this approach, we consistently detected a distinct $APOA1^{+}$ $FABP1^{+}$ subpopulation within the $CD45^{+}$ $CD68^{+}$ $C1Q^{+}$ gate in tumor tissues, providing direct evidence that these transcripts are present in bona fide macrophage-lineage cells rather than reflecting extracellular RNA contamination or engulfed hepatocyte debris.
In parallel, we conducted a computational assessment of potential doublet artifacts using the R package DoubletFinder, as recommended in the literature. The predicted doublet rate within the LAM cluster was relatively low, arguing against

the possibility that this population results from macrophage–hepatocyte doublets or other technical artifacts.

Meanwhile, mIHC in previous cohort from non-responder (NR) samples was also conducted to validate the presence of LAM. The result illustrates that NR patients exhibited great abundance of $CD68^+$ $APOA1^+$ LAMs, which were in close spatial proximity and interaction with HCC cells.

Together, by combining flow cytometric validation with computational doublet analysis and mIHC, which are consistent with the validation strategies proposed in prior study, we demonstrate that the $APOA1^+$ $FABP1^+$ $C1Q^+$ population identified in our study represents a genuine macrophage subset instead of a spurious artifact of single-cell sequencing.

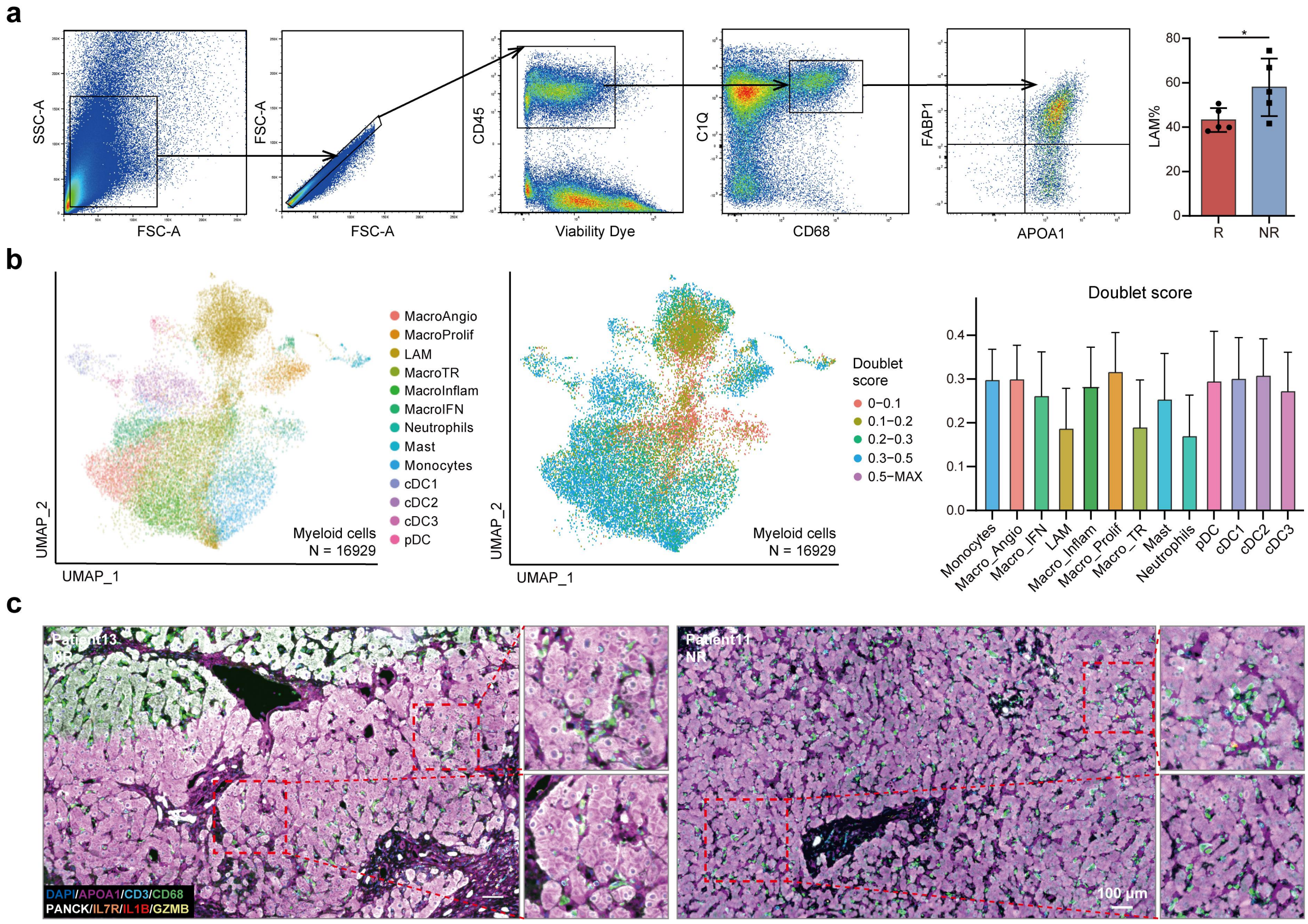

Validation of lipid associated macrophage. a Flow cytometry of tumor tissue from HCC patient demonstrated the presence of $CD68^+$ $C1Q^+$ $APOA1^+$ $FABP1^+$ LAM. **b** R package DoubletFinder estimate the proportion of predicted doublets within the myeloid cluster. LAM population is mostly singlet. **c** Representative mIHC staining of tumor tissues from non-responders patient 11 and 13. LAMs are identified based on the co-expression of CD68 and APOA1.

Reference:

Hume DA, Millard SM, Pettit AR. Macrophage heterogeneity in the single-cell era: facts and artifacts. Blood. 2023 Oct 19;142(16):1339-1347.

4.The observation – more macrophages with subtype positive for C1QA, APOA1, and FABP1 - could be simply linked to increased hepatic steatosis in non-responders. Unfortunately, data on the underlying liver etiology of the analyzed patients is not included in the Table S2. Previous studies (e.g., Pfister et al Nature 2021) have shown that anti-PD-1 monotherapy is less effective in HCC patients with NAFLD or NASH etiology. It remains a question whether the findings in this study are related to these prior observations. Data from Figure 2 show a negative correlation between therapy efficacy and Fatty acid associated HCC, and lipid-associated markers. Thus, the liver etiology of patients, and the level of liver steatosis need to be included in the manuscript and discussed in results and discussion in context of the study from Pfister et al Nature 2021.

Author’s response: We thank the reviewer for raising this important point regarding the potential contribution of underlying liver etiology and hepatic steatosis to the enrichment of lipid-associated macrophages (LAMs) in

non-responders. We fully agree that metabolic liver diseases, including NAFLD/NASH and MASH, have been reported to negatively influence the efficacy of immune checkpoint blockade in HCC, as demonstrated by Pfister, et al. (Nature, 2021) and Chan, et al. (Lancet, 2025).

In response, we have now provide detailed baseline information on liver disease etiology and the degree of hepatic steatosis for all patients from our cohort in Supplementary Table 2. Notably, while a subset of patients exhibited mild hepatic steatosis, none of the patients met diagnostic criteria for overt NAFLD or NASH, and no patients had advanced metabolic liver disease at baseline.

To further address the reviewer's concern, we performed an additional analysis examining the relationship between systemic metabolic status and LAM abundance. Specifically, we assessed the correlation between LAM proportion and body mass index (BMI) across patients. This analysis revealed only a weak negative correlation without statistical significance, suggesting that LAM enrichment in non-responders cannot be simply explained by baseline obesity or systemic metabolic status. The BMI of HCC immunotherapy patients are recorded in Supplementary Table 1 and below.

Importantly, the enrichment of LAMs in non-responders was observed within the TIME and was accompanied by transcriptional programs related to lipid handling, extracellular matrix remodeling, and immune suppression, rather than reflecting generalized hepatic steatosis alone. These findings suggest that LAM accumulation represents a localized, therapy-associated state, rather than a mere surrogate of underlying liver fat content. Accordingly, we have conducted description and discussion in corresponding parts of our manuscript (line 390 to 394) (line 673 to 676).

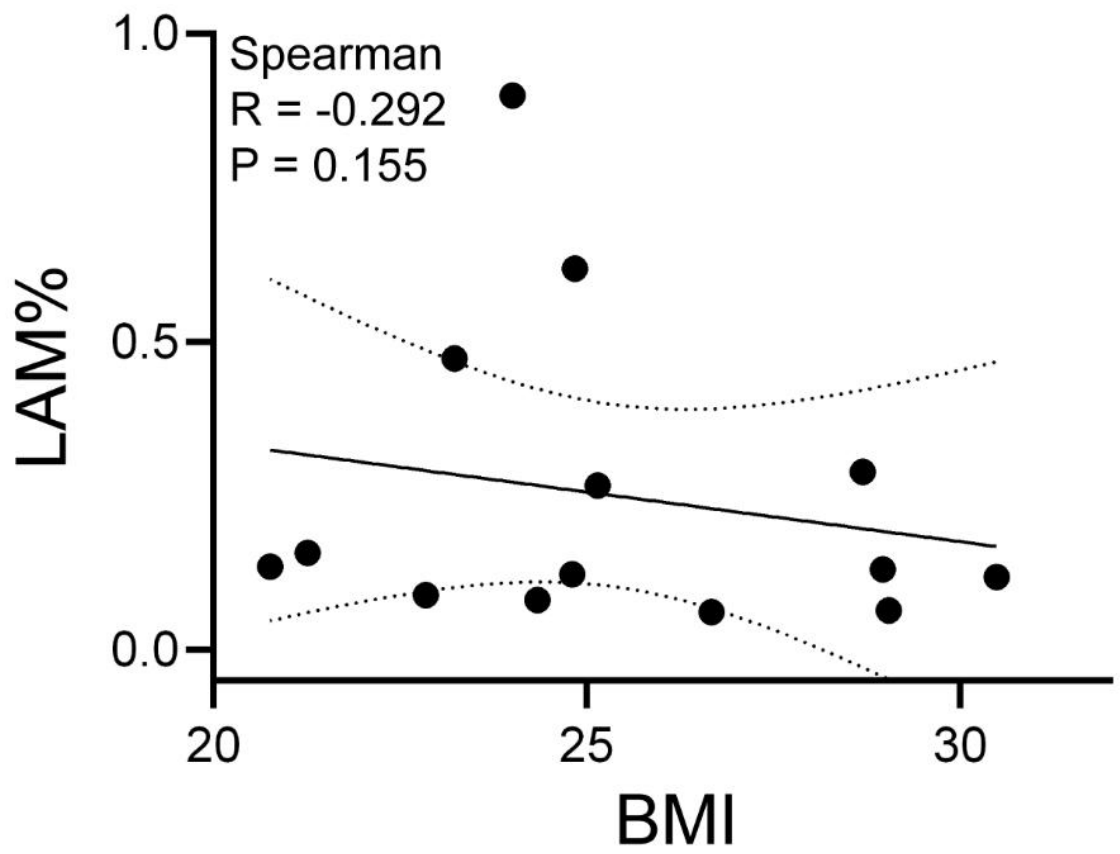

Correlation analysis between proportion of LAM in myeloid cells and BMI of each patient.

Supplementary Table 1. Patient treatment line of our scRNA-seq cohort

Sample_ID	Pathology	Resource	Sex	Age	Other disease	BMI	PD-1 antibody	Dose Schedule	Cycles	Interval from Last Dose to Surgery (Days)	Perioperative Delays
Patient01	HCC	Post_surgery	Female	47	HBV	26.67	Tislelizumab	200mg q3w	3	24	NO
Patient02	HCC	Post_surgery	Male	41	HBV	29.04	Camrelizumab	200mg q3w	3	41	NO
Patient03	HCC	Post_surgery	Male	56	HBV	22.84	Tislelizumab	200mg q3w	5	47	NO
Patient04	HCC	Post_surgery	Male	28	HBV	25.14	Tislelizumab	200mg q3w	5	54	NO
Patient05	HCC	Post_surgery	Male	41	HBV	28.69	Camrelizumab	200mg q3w	3	40	YES
Patient06	HCC	Post_surgery	Female	55	HBV+Fatty liver	23.23	Tislelizumab	200mg q3w	5	32	NO
Patient07	HCC	Post_surgery	Male	67	HBV	24.34	Tislelizumab	200mg q3w	5	27	NO
Patient08	HCC	Post_surgery	Male	56	HBV	20.76	Tislelizumab	200mg q3w	5	27	NO
Patient09	HCC	Post_surgery	Male	59	HBV	30.49	Camrelizumab	200mg q3w	4	39	NO
Patient10	HCC	Post_surgery	Male	45	HBV+Fatty liver	21.26	Camrelizumab	200mg q3w	5	38	NO
Patient11	HCC	Post_surgery	Male	65	HBV	24.84	Camrelizumab	200mg q3w	5	44	NO
Patient12	HCC	Post_surgery	Male	53	HBV+Fatty liver	28.96	Sintilimab	200mg q3w	5	27	YES
Patient13	HCC	Post_surgery	Male	63	HBV+Fatty liver	24.00	Camrelizumab	200mg q3w	3	34	NO
Patient14	HCC	Post_surgery	Male	66	HBV	24.80	Camrelizumab	200mg q3w	3	34	NO
**Sample_ID**	**Reasons for Perioperative delay**	**Treatment period**	**Adverse Events Level**	**Adverse Events**	**Surgical Complications**	**RECIST**	**Pathologic response**	**Efficacy group**	**Residual tumor**	**RFS**	**OS**
Patient01	/	9w	/	/	NO	PR	MPR	Responder	0.2	62	420
Patient02	/	9w	/	/	NO	SD	non-MPR	Non-responder	0.5	131	1032
Patient03	/	15w	2	Transaminase Elevation	NO	SD	MPR	Responder	0.2	792	992
Patient04	/	15w	2	Proteinuria	NO	/	MPR	Responder	0.15	34	573
Patient05	Hypoproteinemia	9w	3	Hypoproteinemia, Proteinuria	NO	PR	non-MPR	Non-responder	0.55	723	800
Patient06	/	15w	2	Hypertension, Dizziness	NO	PD	MPR	Responder	0.45	637	880
Patient07	/	15w	2	Hypertension, Gingival Bleeding	NO	SD	CPR	Responder	0	648	782
Patient08	/	15w	3	Hypothyroidism, Fatigue	NO	PR	non-MPR	Non-responder	0.6	90	285
Patient09	/	12w	2	Transaminase Elevation	NO	SD	non-MPR	Non-responder	0.65	622	730
Patient10	/	15w	3	Hypertension, Dizziness	NO	PR	MPR	Responder	0.1	571	876
Patient11	/	15w	2	Loss of Appetite, Nausea	NO	/	non-MPR	Non-responder	0.7	566	724
Patient12	Headache accompanied by right-sided eyelid ptosis was observed, considering neurologic adverse event	15w	3	Headache, Upper Eyelid Ptosis	Wound Oozing	PR	MPR	Responder	0.1	601	847
Patient13	/	9w	2	Nausea, Abdominal Discomfort	NO	SD	non-MPR	Non-responder	0.8	/	/
Patient14	/	9w	2	Hypertension	NO	SD	CPR	Responder	0	542	590

Result section:

To demonstrate that LAM and HCC_FA subsets are independent of hepatic steatosis in patients and associated with immunotherapy efficacy, we performed correlation analyses between the proportions of the LAM, HCC_FA subsets and patient BMI (Supplementary Fig. 6a). Neither subset showed a statistically significant correlation with BMI.

Discussion section:

Secondly, the percentage of lipid-associated macrophage (LAM) of individual patients is not directly related to fatty liver, leaving the relationship between LAM formation and the state of fatty liver disease as a worthy issue to explore in the future.

Reference:

Pfister D, Núñez NG, Pinyol R, Govaere O, Pinter M, et al. NASH limits anti-tumour surveillance in immunotherapy-treated HCC. Nature. 2021 Apr;592(7854):450-456.

Chan SL, Sun HC, Xu Y, Zeng H, El-Serag HB, et al. The Lancet Commission on addressing the global hepatocellular carcinoma burden: comprehensive strategies from prevention to treatment. Lancet. 2025 Aug 16;406(10504):731-778.

5.As in some cases of non-responders, no residual tumor were observed, what was then the tissue collected? Based on the data it seem that those were only immune cells in necrotic area? he data suggest these were immune cells in necrotic areas. In such cases, is it valid to speak of "characterizing the TIME of HCC" when no viable tumor cells were present?

Author's response: We would like to first clarify that all surgical resections following immunotherapy in this study were accurately targeted to HCC lesions, and all tissues subjected to single-cell RNA sequencing were obtained from resected HCC tumor specimens, rather than from non-tumor liver tissue. Therefore, even when viable tumor cells were limited in some samples, the tissues used for scRNA-seq still represent the post-treatment tumor bed of HCC and characterized the TIME which is methodologically appropriate to describe our analyses as characterizing the TIME of HCC.

Therapeutic response in our study was assessed based on postoperative pathological evaluation of residual tumor percentage, which is widely regarded as the gold standard for pathological response assessment (Llovet, et al.) (D'Alessio, et al.). Accordingly, cases classified as non-responders did exhibit residual viable tumor according to pathological criteria, although the amount of tumor detected in certain sections was limited. It is important to note that the tissue region used for single-cell dissociation does not necessarily correspond exactly to the region examined in diagnostic pathological sections. As a result, discrepancies between the number of tumor cells captured by single-cell sequencing and those observed on pathology slides can occur. Such sampling-related mismatches have been reported in prior single cell study, particularly in the neoadjuvant immunotherapy setting (Yang, et al.), where spatial heterogeneity and treatment-induced tumor regression can lead to partial representation of tumor cells in dissociated samples.

In our study, single-cell profiling was performed on tissue obtained from the tumor bed region, which contains the immune microenvironment shaped by PD-1 blockade, including infiltrating immune cells, stromal components, and residual tumor. Even in samples with low tumor cell representation, these regions remain highly informative for understanding the treatment-modulated TIME. Notably, overall therapeutic efficacy showed a significant negative correlation with the proportion of HCC, reflecting this was caused by biological and spatial heterogeneity rather than systematic bias. To transparently address this point, we include a scatter plot illustrating the relationship between tumor residual rate and clinical outcomes, which helps contextualize individual outliers such as data of patient04 and patient06, and supports the robustness of our conclusions.

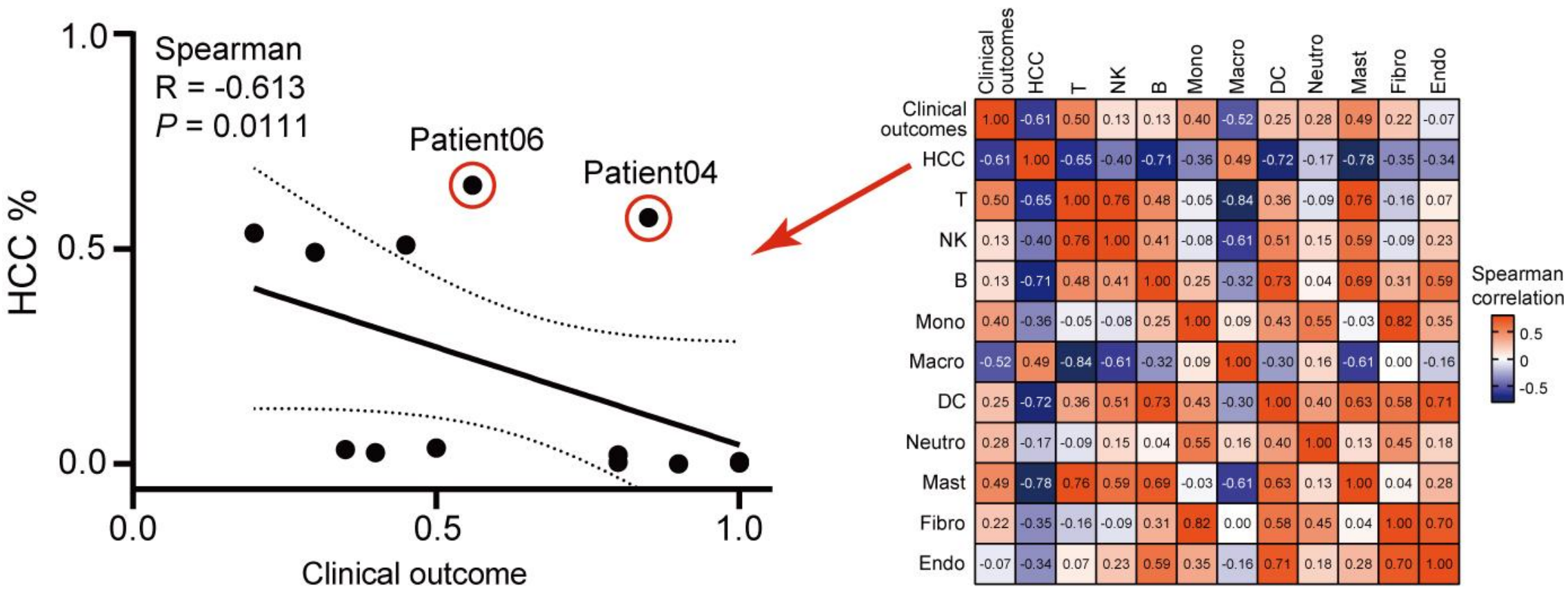

Scatter plot of correlation between HCC proportion and clinical outcome of our HCC immunotherapy cohort.

Reference:

Llovet JM, Pinyol R, Yarchoan M, Singal AG, Marron TU, Schwartz M, Pikarsky E, Kudo M, Finn RS. Adjuvant and neoadjuvant immunotherapies in hepatocellular carcinoma. Nat Rev Clin Oncol. 2024 Apr;21(4):294-311.

D'Alessio A, Stefanini B, Blanter J, Adegbite B, Crowley F, Yip V, Slater S, Fulgenzi CAM, Celsa C, Manfredi GF, Pai M, Goldin RD, Ward SC, Fiel MI, Shu DH, Su YY, Cortellini A, Baretti M, Anders R, Yarchoan M, Hsu C, Marron TU, Pinato DJ. Pathological response following neoadjuvant immune checkpoint inhibitors in patients with hepatocellular carcinoma: a cross-trial, patient-level analysis. Lancet Oncol. 2024 Nov;25(11):1465-1475.

Yang Z, Tian H, Chen X, Li B, Bai G, Cai Q, Xu J, Guo W, Wang S, Peng Y, Liang Q, Xue L, Gao S. Single-cell sequencing reveals immune features of treatment response to neoadjuvant immunochemotherapy in esophageal squamous cell carcinoma. Nat Commun. 2024 Oct 22;15(1):9097.

6.Five external cohorts (61 HCC cases) did not helped in the characterizations of TIME components associated with immunotherapy outcomes, as these patients were not treated by immunotherapy in that 5 cohorts. As I understood, the 5 external cohorts served rather as a control for analysis of the cohort of 14 patients. Therefore these data should be moved to supplementary part and study should focus on the main message already from the first figure.

Author's response: We thank the reviewer for this helpful comment. We agree that the five external cohorts adopted in our study were not designed to evaluate immunotherapy outcomes, and therefore do not directly contribute to the identification of TIME features associated with αPD-1 response.

We would like to clarify that these external datasets were used exclusively for robust cell-type and cell-state annotation, serving as large-scale transcriptional references to improve the accuracy and stability of immune and stromal cell classification in HCC. Importantly, all analyses related to immunotherapy efficacy, including response-associated cell state comparisons, cell–cell communication networks, and pathway enrichment were restricted to our in house neoadjuvant immunotherapy cohort and these independent immunotherapy-treated cohorts were used to validate the features of HCC_FA and LAM (Fig. 2h-j, Supplementary Fig. 6e).

In addition, to further strengthen the reliability of our annotations, we constructed an expanded external HCC immunotherapy single-cell cohort and performed auxiliary annotation using this dataset. Under this independent immunotherapy-specific reference framework, we obtained highly consistent identification of the same T cell and macrophage subpopulations, including the LAM subset highlighted in our study. These results further support that the identified cell states are robust, reproducible, and not artifacts of dataset selection or annotation bias.

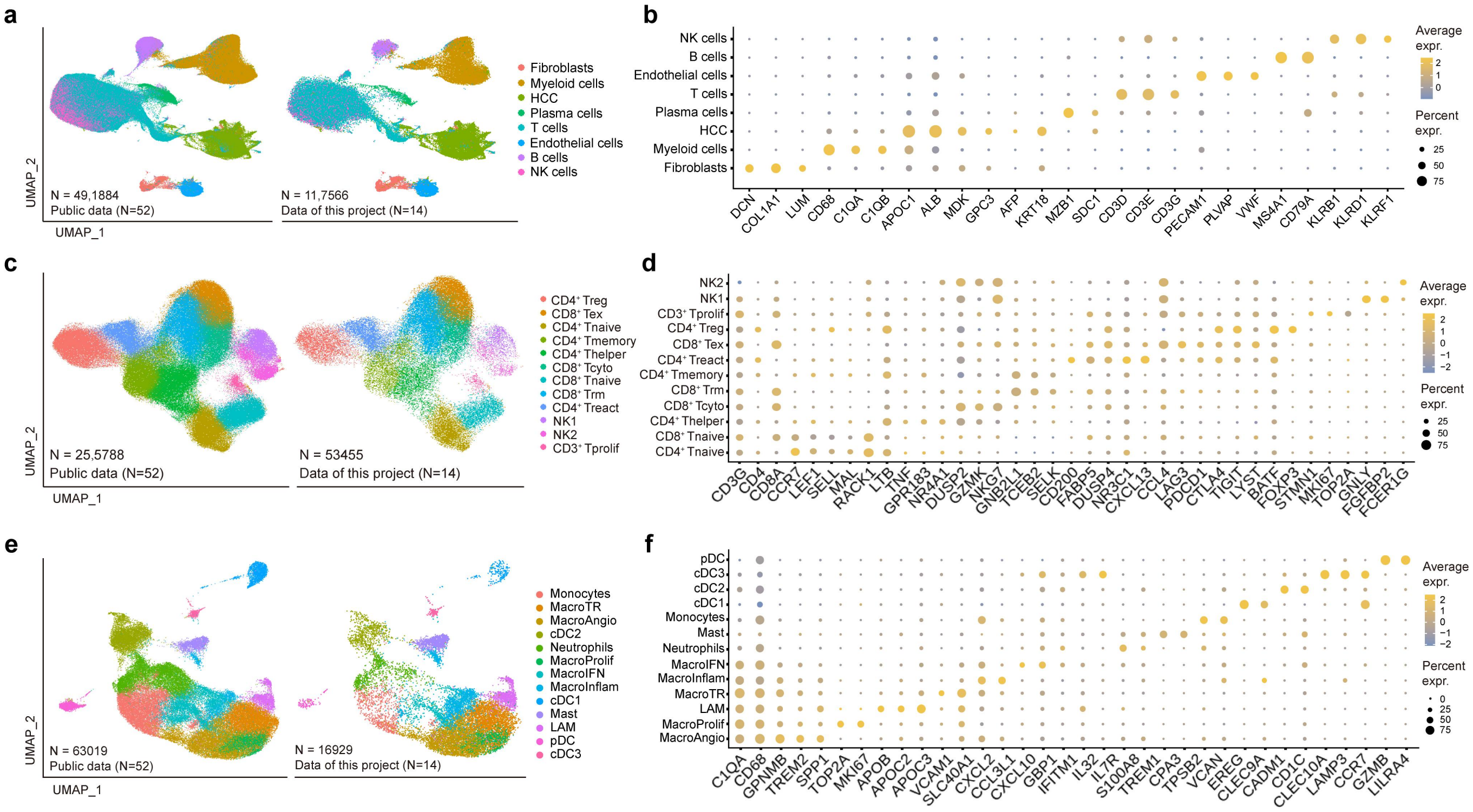

Clustering and annotation of major cell types, T cell and myeloid cell subtypes in HCC immunotherapy cohort of our and external datasets. a UMAP plot of all cells from the integrated single cell immunotherapy atlas and our HCC immunotherapy cohort. **b** Major cell types identified from **(a)** and corresponding gene expression features. **c** UMAP plot of T cells and NK cells from the integrated single cell immunotherapy atlas and our HCC immunotherapy cohort. **d** Major cell types identified from **(c)** and corresponding gene expression features. **e** UMAP plot of myeloid cells from the integrated single cell immunotherapy atlas and our HCC immunotherapy cohort. **f** Major cell types identified from **(e)** and corresponding gene expression features.

7. Lipid-associated macrophages are commonly referred to as LAMs, not "MacroLA." Why did the authors introduce a new abbreviation, when "LAMs" is already well established in the field? Moreover, TREM2^{+} LAMs have previously been described in HCC (PMID: 35359989), and this should be acknowledged and discussed.

Author's response: We thank the reviewer for this constructive comment and fully acknowledge that "LAMs" is the widely accepted term for lipid-associated macrophages. In response to the reviewer's suggestion, we have revised the manuscript to replace the term "MacroLA" with "LAM" throughout.

We would also like to clarify why we initially used a different abbreviation. The lipid-associated macrophage population identified in our dataset shows a transcriptional profile that differs from the previously reported TREM2^{+}/SPP1^{+}/FOLR2^{+} LAMs, including those described in HCC (PMID: 35359989, Zhou, et.al). Specifically, the LAMs in our study do not express high levels of canonical LAM markers such as TREM2 or FOLR2 (Ma, et.al); instead, they exhibit a distinct lipid-handling signature characterized by high expression of FABP1, APOA1, and related genes. Conversely, these lipid metabolism related genes were rarely expressed in typical SPP1^{+} macrophages, where the SPP1 expression is extensive and intense. This molecular divergence led us to initially adopt a separate abbreviation. However, given that these cells are clearly lipid-associated macrophages, we agree that using the standard terminology "LAM" is more appropriate and improves clarity for readers.

Importantly, we did identify a TREM2^{+} macrophage population in our cohort as well. Consistent with prior studies, this TREM2^{+} group displayed features associated with angiogenesis rather than lipid metabolism, and it is transcriptionally distinct from the FABP1^{+}APOA1^{+} LAM subset that we highlight in the manuscript. We have now discussed this distinction and referenced the relevant literature, including the reported TREM2^{+} LAMs in HCC (line 321 to 327).

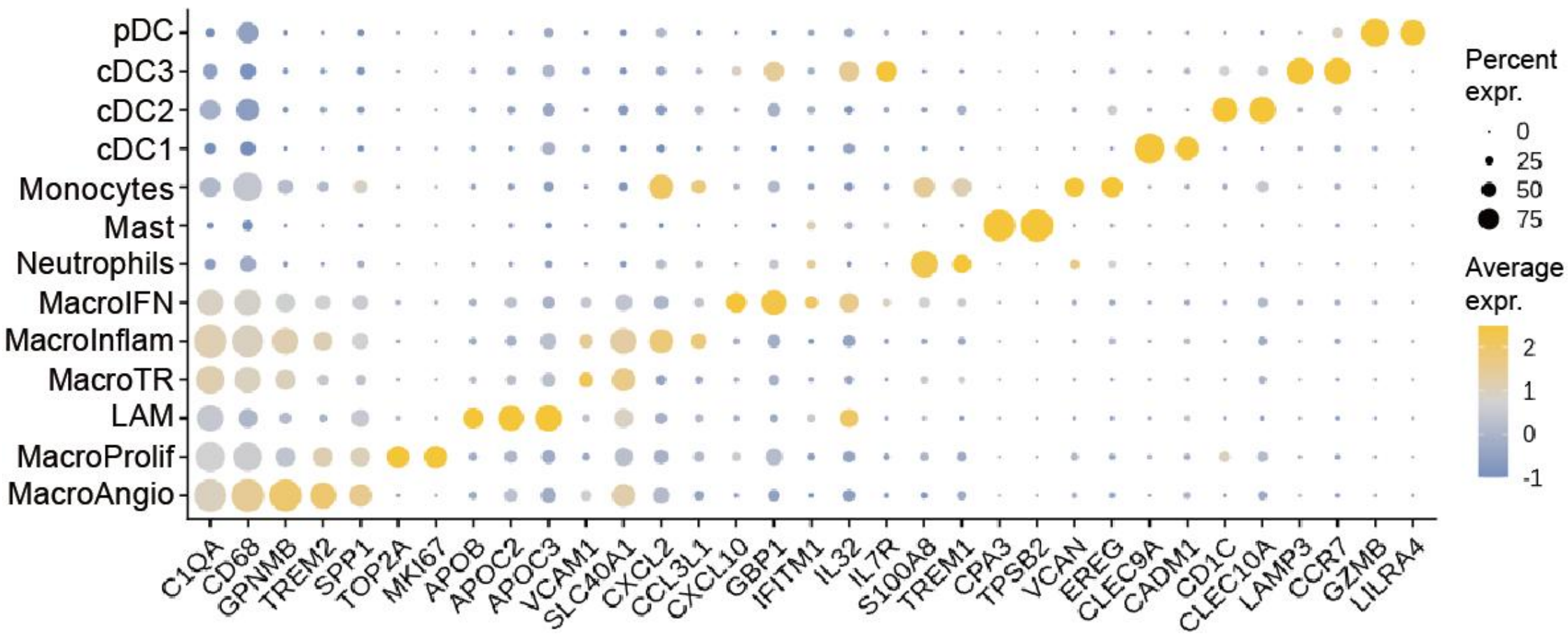

Gene expression features of 13 myeloid subtypes.

Reference:
Zhou L, Wang M, Guo H, Hou J, Zhang Y, Li M, Wu X, Chen X, Wang L. Integrated Analysis Highlights the Immunosuppressive Role of TREM2+ Macrophages in Hepatocellular Carcinoma. Front Immunol. 2022 Mar 14;13:848367.
Ma RY, Black A, Qian BZ. Macrophage diversity in cancer revisited in the era of single-cell omics. Trends Immunol. 2022 Jul;43(7):546-563.

8. Many of the figures are confusing and do not clearly support the study's main message—for example, Figure 1H. In my view, such figures are more distracting than informative. A more focused presentation would be beneficial.

Author's response: We appreciate the reviewer's insightful comment. The original purpose of Fig. 1h, for instance, was to illustrate the stability and robustness of our clustering strategy. This panel presents the non-negative matrix factorization (NMF) decomposition of the major cell populations, demonstrating that cells with distinct transcriptional programs can be consistently summarized into coherent and biologically meaningful components.
Importantly, Fig. 1h is complementary to Fig. 1c rather than redundant. While Fig. 1b and 1c depict cell clusters derived from unsupervised clustering based on single-cell expression profiles, Fig. 1h provides an independent decomposition of the same data using an NMF-based computational framework. The high concordance between unsupervised clustering results and NMF-derived components supports the accuracy and robustness of our cell-type and subcluster identification.
Moreover, NMF has been widely applied and validated in multiple high-impact single-cell and tumor microenvironment studies as a reliable approach for extracting stable transcriptional programs and reducing dimensional complexity (Chen, et al.), (Liu, et al.). By presenting both analyses, we aimed to provide complementary evidence that the identified cellular subtypes and transcriptional features are not artifacts of a single computational method, but instead represent reproducible and biologically grounded structures within the dataset.

Reference:
Chen Y, Wang D, Li Y, Qi L, Si W, Bo Y, Chen X, Ye Z, Fan H, Liu B, Liu C, Zhang L, Zhang X, Li Z, Zhu L, Wu A, Zhang Z. Spatiotemporal single-cell analysis decodes cellular dynamics underlying different responses to immunotherapy in colorectal cancer. Cancer Cell. 2024 Jul 8;42(7):1268-1285.e7.
Liu Z, Yang Z, Wu J, Zhang W, Sun Y, et al. A single-cell atlas reveals immune heterogeneity in anti-PD-1-treated non-small cell lung cancer. Cell. 2025 May 29;188(11):3081-3096.e19.

9. The correlations presented in the study are not clearly defined, particularly with regard to the clinical outcome data. For example, the clinical outcome appears to be represented on a 0–1 scale, but the criteria for this scoring system are not explained. The manuscript refers to this score as a measure of "immunotherapy efficacy" or "response", but a precise definition or explanation of how it was derived is missing. This lack of clarity makes it difficult to interpret the validity.

Author's response: We thank the reviewer for pointing out the need to clarify how immunotherapy efficacy was quantified. In our study, the clinical response score is directly derived from the postoperative pathological tumor residual rate, which is considered the gold standard for assessing therapeutic response. This method has already been described in the manuscript; however, we agree that its current placement may not be sufficiently prominent. To improve clarity, we will revise the text to present this information earlier and more explicitly in the results sections (line 337 to 340), ensuring that readers can clearly understand the basis and rationale of the 0–1 response scale.

In addition, we have provided the pathological tumor residual rate for each individual patient in the Table.S1, allowing transparent assessment of the underlying data used to derive the response score. Our classification strategy follows current Chinese clinical guidelines for hepatocellular carcinoma, which shows a 50% residual tumor threshold is recommended for pathological response assessment (Zhou, et al.).

For pathological response categorization, patients were classified as non- major pathological response (non-MPR), major pathological response (MPR), or complete pathological response (CPR) based on residual viable tumor percentages of >=50%, 0–50%, and 0%, respectively. We acknowledge that cutoff values for MPR can vary across tumor types and clinical trials (Llovet, et al.), (D'Alessio, et al.) and our research was conducted according to the recommendations of practice from clinical guidelines within the region of our research project.

Supplementary Table 1. Patient treatment line of our scRNA-seq cohort

Sample_ID	Pathology	Resource	Sex	Age	Other disease	BMI	PD-1 antibody	Dose Schedule	Cycles	Interval from Last Dose to Surgery (Days)	Perioperative Delays
Patient01	HCC	Post_surgery	Female	47	HBV	26.67	Tislelizumab	200mg q3w	3	24	NO
Patient02	HCC	Post_surgery	Male	41	HBV	29.04	Camrelizumab	200mg q3w	3	41	NO
Patient03	HCC	Post_surgery	Male	56	HBV	22.84	Tislelizumab	200mg q3w	5	47	NO
Patient04	HCC	Post_surgery	Male	28	HBV	25.14	Tislelizumab	200mg q3w	5	54	NO
Patient05	HCC	Post_surgery	Male	41	HBV	28.69	Camrelizumab	200mg q3w	3	40	YES
Patient06	HCC	Post_surgery	Female	55	HBV+Fatty liver	23.23	Tislelizumab	200mg q3w	5	32	NO
Patient07	HCC	Post_surgery	Male	67	HBV	24.34	Tislelizumab	200mg q3w	5	27	NO
Patient08	HCC	Post_surgery	Male	56	HBV	20.76	Tislelizumab	200mg q3w	5	27	NO
Patient09	HCC	Post_surgery	Male	59	HBV	30.49	Camrelizumab	200mg q3w	4	39	NO
Patient10	HCC	Post_surgery	Male	45	HBV+Fatty liver	21.26	Camrelizumab	200mg q3w	5	38	NO
Patient11	HCC	Post_surgery	Male	65	HBV	24.84	Camrelizumab	200mg q3w	5	44	NO
Patient12	HCC	Post_surgery	Male	53	HBV+Fatty liver	28.96	Sintilimab	200mg q3w	5	27	YES
Patient13	HCC	Post_surgery	Male	63	HBV+Fatty liver	24.00	Camrelizumab	200mg q3w	3	34	NO
Patient14	HCC	Post_surgery	Male	66	HBV	24.80	Camrelizumab	200mg q3w	3	34	NO

Sample_ID	Reasons for Perioperative delay	Treatment period	Adverse Events Level	Adverse Events	Surgical Complications	RECIST	Pathologic response	Efficacy group	Residual tumor	RFS	OS
Patient01	/	9w	/	/	NO	PR	MPR	Responder	0.2	62	420
Patient02	/	9w	/	/	NO	SD	non-MPR	Non-responder	0.5	131	1032
Patient03	/	15w	2	Transaminase Elevation	NO	SD	MPR	Responder	0.2	792	992
Patient04	/	15w	2	Proteinuria	NO	/	MPR	Responder	0.15	34	573
Patient05	Hypoproteinemia	9w	3	Hypoproteinemia, Proteinuria	NO	PR	non-MPR	Non-responder	0.55	723	800
Patient06	/	15w	2	Hypertension, Dizziness	NO	PD	MPR	Responder	0.45	637	880
Patient07	/	15w	2	Hypertension, Gingival Bleeding	NO	SD	CPR	Responder	0	648	782
Patient08	/	15w	3	Hypothyroidism, Fatigue	NO	PR	non-MPR	Non-responder	0.6	90	285
Patient09	/	12w	2	Transaminase Elevation	NO	SD	non-MPR	Non-responder	0.65	622	730
Patient10	/	15w	3	Hypertension, Dizziness	NO	PR	MPR	Responder	0.1	571	876
Patient11	/	15w	2	Loss of Appetite, Nausea	NO	/	non-MPR	Non-responder	0.7	566	724
Patient12	Headache accompanied by right-sided eyelid ptosis was observed, considering neurologic adverse event	15w	3	Headache, Upper Eyelid Ptosis	Wound Oozing	PR	MPR	Responder	0.1	601	847
Patient13	/	9w	2	Nausea, Abdominal Discomfort	NO	SD	non-MPR	Non-responder	0.8	/	/
Patient14	/	9w	2	Hypertension	NO	SD	CPR	Responder	0	542	590

Result section:

One of the advantages of our 14-patient HCC immunotherapy cohort is the integration of pathological evaluations of resected tumor lesions, we defined the clinical outcomes of patients as "1 - residual tumor rate", thereby allowed us to quantitatively assess the immunotherapeutic efficacy following αPD-1 treatment.

Reference:

Llovet JM, Pinyol R, Yarchoan M, Singal AG, Marron TU, Schwartz M, Pikarsky E, Kudo M, Finn RS. Adjuvant and neoadjuvant immunotherapies in hepatocellular carcinoma. Nat Rev Clin Oncol. 2024 Apr;21(4):294-311.

D'Alessio A, Stefanini B, Blanter J, Adegbite B, Crowley F, Yip V, Slater S, Fulgenzi CAM, Celsa C, Manfredi GF, Pai M, Goldin RD, Ward SC, Fiel MI, Shu DH, Su YY, Cortellini A, Baretti M, Anders R, Yarchoan M, Hsu C, Marron TU, Pinato DJ. Pathological response following neoadjuvant immune checkpoint inhibitors in patients with hepatocellular carcinoma: a cross-trial, patient-level analysis. Lancet Oncol. 2024 Nov;25(11):1465-1475.

Zhou J, Sun H, Wang Z, Cong W, Zeng M, et al. China Liver Cancer Guidelines for the Diagnosis and Treatment of

Hepatocellular Carcinoma (2024 Edition). Liver Cancer. 2025 Jul 10;14(6):779-835.

10. Figure 1C: I am surprised by mixing T and NK cells in the same cluster characterised by CD3D,CD3E, CD3G etc. NK cells are known to be CD3-. Can authors comment on this point?

Author's response: We thank the reviewer for raising this important point. In the initial stage of our analysis, T cells and NK cells were grouped together as a broad lymphocyte category because of their functional similarity and partially overlapping transcriptomic features at the coarse clustering level. This practice is also commonly used in other single-cell studies (Liu, et al.) (Cui, et al.), where major immune lineages are first grouped into broad categories before being resolved into finer subsets during subsequent sub-clustering.

Importantly, in all downstream analyses, including refined sub-clustering and lineage-specific characterization, we clearly separated T cells from NK cells based on canonical markers.

To avoid any potential misunderstanding and based on your recommendation, we will revise the major-cluster annotation in the manuscript to present T cells and NK cells as distinct clusters from the outset and will update the corresponding figures accordingly.

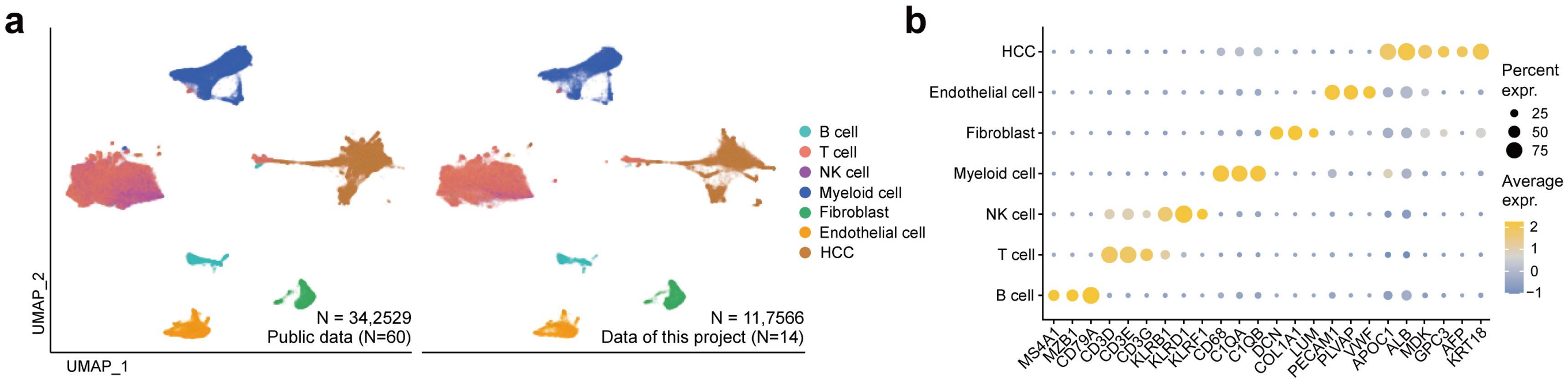

Single cell atlas integrated by public datasets of HCC patients and our HCC immunotherapy cohort. a Uniform manifold approximation and projection (UMAP) plot of all cells colored by major cell types (B cells, T cells, NK cells, Myeloid cells, Fibroblasts, Endothelial cells and HCC) according to specific markers. **b** Major cell types identified from **(a)** and corresponding gene expression features.

Reference:

Liu Z, Zhang Y, Ma N, Yang Y, Ma Y, Wang F, Wang Y, Wei J, Chen H, Tartarone A, Velotta JB, Dayyani F, Gabriel E, Wakefield CJ, Kidane B, Carbonelli C, Long L, Liu Z, Su J, Li Z. Progenitor-like exhausted SPRY1+CD8+ T cells potentiate responsiveness to neoadjuvant PD-1 blockade in esophageal squamous cell carcinoma. Cancer Cell. 2023 Nov 13;41(11):1852-1870.e9.

Cui X, Liu S, Song H, Xu J, Sun Y. Single-cell and spatial transcriptomic analyses revealing tumor microenvironment remodeling after neoadjuvant chemoimmunotherapy in non-small cell lung cancer. Mol Cancer. 2025 Apr 9;24(1):111.

11. Legends of graphs are often not completed: for instance, Figure 2E, what are the value 0-1, and the second value 0-1?

Author's response: We appreciate the reviewer's comment regarding the unclear axis description in Fig. 2e. The current visualization directly reflects the default output format generated by the scMetabolism R package, which indeed may lead to ambiguity in how the 0–1 scales are displayed.

To improve clarity, we will revise the legend of Fig. 2e to explicitly define what each 0–1 value represents and how these values are computed within the scMetabolism framework. The updated legend and accompanying text will ensure that the figure is fully interpretable to readers.

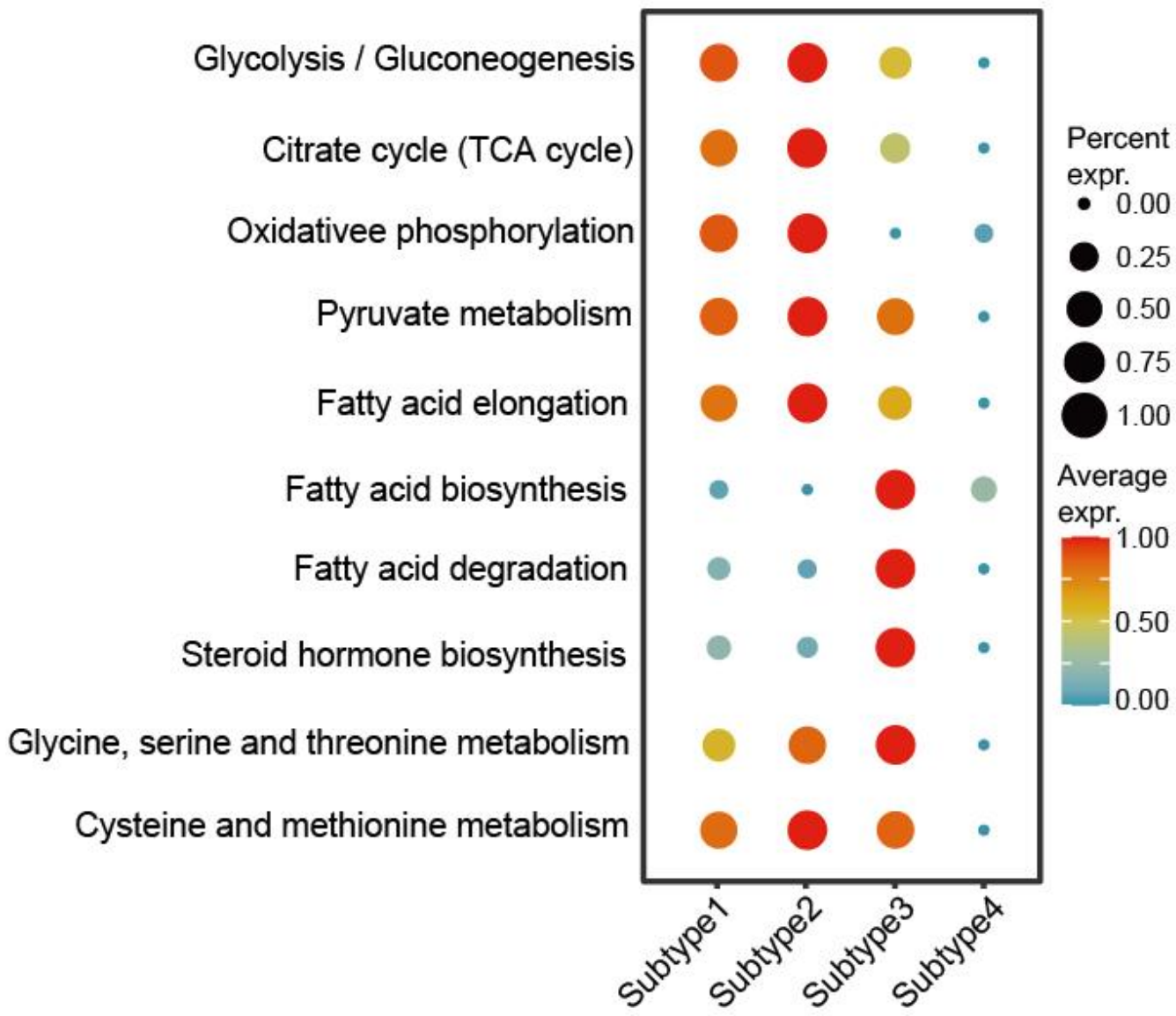

Metabolism pathway analysis of HCC cells. The size of the dot means percentage of expression in metabolism pathway and the color of dot shows average expression level of each metabolism pathway.

12. Table S1: "Expression characteristics of each major cell type" – This list raises many questions about the clusters. HCC markers are rather hepatocyte markers, so their presence suggests hepatic origin, but they are not specific enough to be definitive for HCC. Why AFP (Alpha-fetoprotein) or GPC3 (Glypican-3) were not included? Some markers listed as NK cell markers, are not relevant for NK cells and indicate contamination by other cell types. TRDC – TCR delta chain; associated with γδ T cells, not NK cells. KRT81, KRT86 are typically expressed in epithelial cells, and CLIC3 is also not an established NK cell marker, at least to my knowledge. Concerning T cells: TUBA4A – A tubulin gene is ubiquitously expressed, should not be used as T cell marker. SPOCK2 is an ECM-related gene not specific to T cells, and its expression is generally low in immune cells. PTPRC (CD45) is a pan-leukocyte marker that is expressed in all lymphocytes, not specifically in T cells.

Author's response: We thank the reviewer for the detailed and insightful comments regarding the gene lists presented in supplementary table and would like to clarify several important points.

First, the genes shown in Supplementary Table 3, 7 and 9 represent the differentially expressed genes (DEGs) of each broad cluster, rather than manually curated canonical markers. Broad clusters generated during the initial coarse clustering step may contain a small proportion of infiltrating or transcriptionally neighboring cells, which is common in single-cell analyses. As a result, the DEG output may occasionally include genes associated with other cell types. Importantly, this does not indicate misannotation but rather reflects the inherent heterogeneity captured before finer subclustering. In addition, due to space limitations in the initial submission and the fact that DEG ranking is influenced by multiple factors, including average fold change, statistical significance, and the proportion of expressing cells. Some well-recognized characteristic genes may not have been included in the originally displayed lists. We have therefore optimized and revised Supplementary Table 3 accordingly to better reflect representative transcriptional features.

Final cell type annotation in our study was performed on refined subclusters using canonical marker genes and manual curation. To avoid further confusion, we will revise the table title to explicitly clarify this distinction.

Second, regarding the recognition malignant hepatocellular carcinoma (HCC) cells, we agree that it cannot always be reliably distinguished using markers alone as the carcinogenesis of HCC cells are continuous. To address this challenge, we performed inferCNV analysis, which enabled us to identify copy-number variation to definite the relatively more malignant HCC cells.

Third, concerning canonical HCC markers, we confirm that *GPC3* and *AFP* are indeed expressed in our HCC cell population, consistent with their established diagnostic relevance in HCC. In response to the reviewer's valuable suggestions, we will revise Fig. 1c to add *GPC3* and *AFP* expression into dot plot.

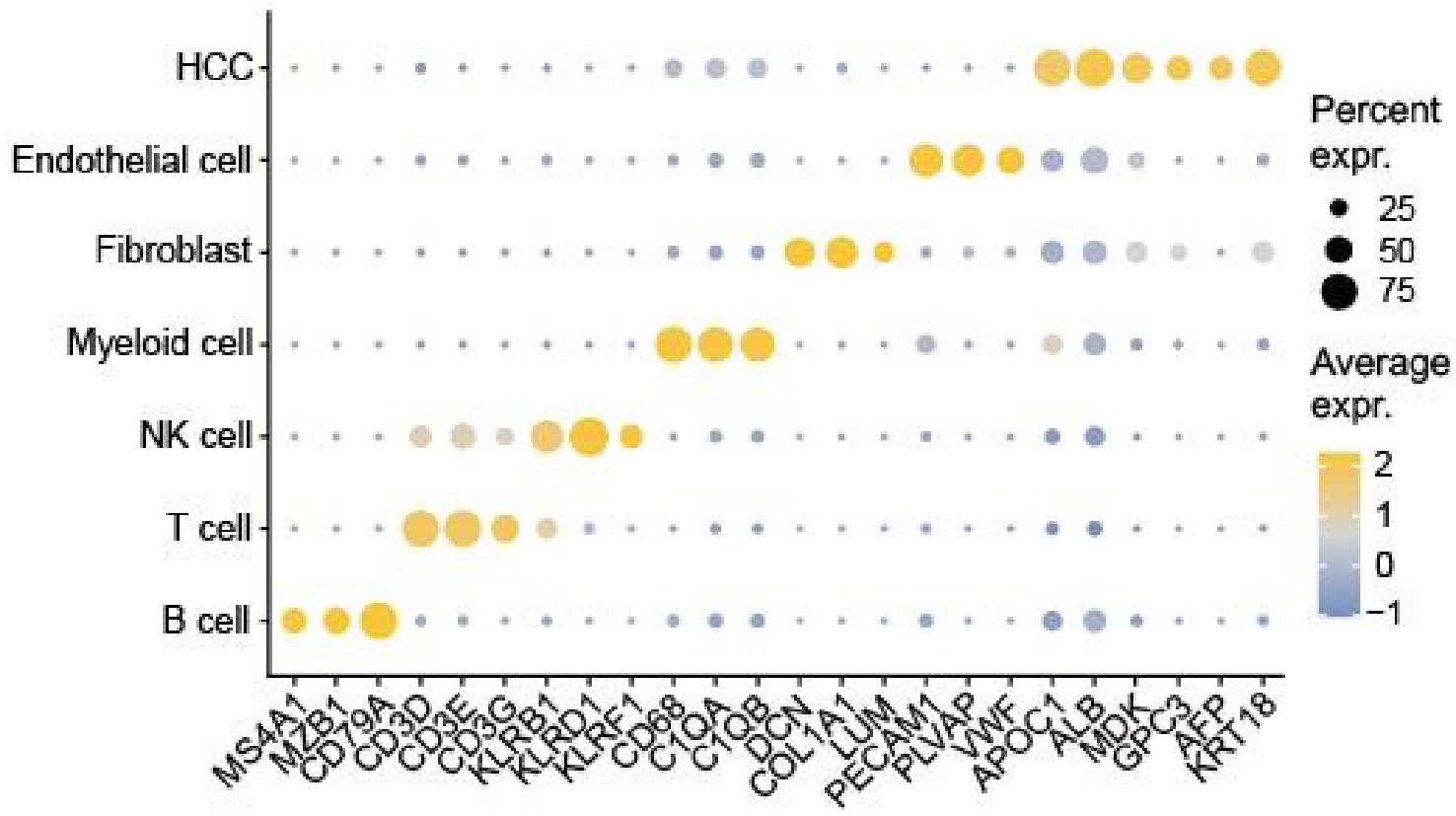

Major cell types and corresponding gene expression features.

13. Figure 4: DCs are traditionally marked by genes like CLEC4C (BDCA2), TCF4, IRF7, IL3RA (CD123), but GZMB expression is generally absent or very low in bona fide pDCs. In this study, GZB is used as a marker of pDC, which is very surprising to me.

Author's response: We thank the reviewer for raising this important point. We fully acknowledge that canonical plasmacytoid dendritic cell (pDC) markers include CLEC4C (BDCA2), IL3RA (CD123), TCF4, and IRF7, and these markers were indeed the primary basis for our pDC annotation.

Accordingly, in our dataset, pDCs were annotated based on canonical markers, and GZMB emerged as one of the top DEGs within this lineage, consistent with pDC phenotypes described in previous pDC researches (Swiecki, et al.), which shows immune regulation function of pDC by induction of cell death or suppression of T cells. Thus, the presence of GZMB in our pDC cluster reflects established biological and transcriptomic patterns rather than misclassification.

[editorial note: third party material redacted]

Immune responses and expression features of pDC (Swiecki, et al.).

Reference:

Swiecki M, Colonna M. The multifaceted biology of plasmacytoid dendritic cells. Nat Rev Immunol. 2015 Aug;15(8):471-85.

14. The English requires revision, as some sentences lack clarity or coherence—for example, sentence: "To address these issues above, our study established a cohort of 14 HCC patients undergoing neoadjuvant immunotherapy and performed single-cell sequencing on their resected tumor lesions." Should be replaced by "To address these issues, we recruited 14 patients with HCC undergoing neoadjuvant immunotherapy and performed single-cell sequencing on resected tumour specimens." As "The study" can't establish a cohort.

Author's response: We thank the reviewer for pointing out the issues related to clarity and coherence in the English language. We fully agree that the original phrasing ("the study established a cohort…") was inappropriate and could cause confusion. In the revised manuscript, we have corrected this sentence to: *"To comprehensively investigate the factors within TIME that affect the efficacy of HCC immunotherapy, we established a clinical cohort of 14 HCC patients that received αPD-1 based immunotherapy.*" (line 117 to 119).

In addition to this specific correction, we have conducted a comprehensive language revision throughout the entire manuscript to improve clarity, readability, and grammatical accuracy. We also refined several other sentences to ensure precise scientific communication. We thank the reviewer again for the kindly reminder.

Reviewer #2: In this study, the authors perform single-cell RNA sequencing (scRNA-seq) on 14 hepatocellular carcinoma (HCC) specimens obtained from patients who received neoadjuvant anti-PD-1 immune checkpoint inhibitor (ICI) prior to surgical resection. The primary aim of the study is to elucidate mechanisms underlying resistance to ICI in HCC and to characterize tumor immune microenvironment (TIME) features associated with treatment response. Through a comparative analysis of responders (R) versus non-responders (NR), the authors identify a fatty-acid associated HCC subtype (HCC_FA) negatively correlated with ICI efficacy. They further identify key immune components associated with clinical outcome. Specifically, enhanced infiltration of $CD4^+$ T helper cells, $CD8^+$ cytotoxic T lymphocytes, and inflammatory macrophages (MacroInflam) is associated with treatment response, while enrichment of $CD4^+$ regulatory T cells and a lipid-associated macrophage (MacroLA) population expressing C1QA, FABP1, and APOA1 is linked to resistance. Their findings are further supported by validation in multiple publicly available HCC scRNA-seq datasets and posterior preclinical assays (in vitro), flow cytometry and IHQ. The study effectively recapitulates previously reported associations, including reduced T cell exhaustion and elevated $CD4^+$ helper activation in responders, while also identifying novel immune subtypes, such as a metabolically distinct HCC subtype (HCC_FA) and the MacroLA population, which may influence therapeutic outcomes.

This manuscript represents a well-executed and thoughtfully designed study. The hypotheses are clearly articulated, the methods are rigorous and appropriately applied, and the results are coherently presented. The conclusions are well-supported by the data, and the discussion clearly contextualizes the findings within the current landscape of immunotherapy in HCC. The identification of lipid-associated macrophages as mediators of immunotherapy resistance and the link with metabolic HCC subtypes offer novel mechanistic insights with potential translational relevance. The work contributes significantly to the understanding of the heterogeneity of immunotherapy responses in HCC and may contribute to the future development of predictive biomarkers and combinatorial therapeutic strategies.

Below, I provide constructive comments and suggestions aimed at improving the quality of this manuscript and encouraging further discussion and clarification of certain results.

Major Points for Discussion:

1. The observed response rate in the study cohort (8 responders vs. 6 non-responders) appears higher than the typically reported ~20-30% response to immunotherapy in HCC. Can the authors clarify whether this discrepancy may be due to differences in disease stage (e.g., early-stage, resectable HCC vs. advanced, unresectable), the use of neoadjuvant rather than palliative immunotherapy, or other clinical factors?

Author's response: We thank the reviewer for raising this important point. As correctly noted, the proportion of responders and non-responders in our single-cell sequencing cohort does not mirror the overall response rate typically observed in neoadjuvant immunotherapy for HCC. This discrepancy arises from the specific intent underlying the construction of our single-cell cohort.

The primary objective of our single-cell analysis was to systematically compare tumor immune microenvironment (TIME) features between patients with different therapeutic responses, rather than to estimate clinical response rates. Therefore, during cohort assembly, samples were pre-selected based on radiological assessment (based on RECIST 1.1 criteria) of response to neoadjuvant immunotherapy, enabling the inclusion of relatively balanced numbers of responder (R) and non-responder (NR) cases for downstream single-cell profiling.

Taken together, this design strategy ensured adequate representation of both response groups and sufficient statistical power for comparative analyses of immune cell states associated with treatment efficacy, but it was not intended to reflect the real-world incidence of response in the broader clinical population.

2. In the clinical cohort, patients received Camrelizumab, Tislelizumab or Sintilimab. Are there known differences between these PD-1 inhibitors? Please, clarify whether differences in dosing regimens or administration protocols could have been applied.

Author's response: We thank the reviewer for raising this important question regarding the use of different PD-1 inhibitors in our clinical cohort.

Regarding clinical decision-making in real-world practice, the choice among camrelizumab, tislelizumab, and sintilimab in our cohort was primarily driven by drug accessibility rather than by an expectation of differential efficacy. During the study period and within our geographic region, all three agents had been approved in China for the treatment of advanced HCC and were recommended by national clinical guidelines as first- or second-line options. In routine clinical practice, when multiple agents within the same therapeutic class are available, prescription decisions are largely influenced by practical considerations, including (i) differences in national or regional reimbursement policies, (ii) hospital formulary availability, and (iii) patient-specific financial considerations and access to patient assistance programs. As a result, the selection of a specific PD-1 inhibitor in our cohort reflects real-world treatment patterns in China rather than biologically driven stratification, which is a common feature of real-world oncology studies.

From mechanistic perspective, we do not believe that the use of different PD-1 inhibitors introduces a major confounding factor for the central conclusions of our study. Camrelizumab, tislelizumab, and sintilimab are all high-affinity humanized monoclonal antibodies targeting PD-1, sharing the same core mechanism of action which blocks the PD-1 axis to restore T cells antitumor immunity. Pivotal phase III clinical trials have demonstrated broadly comparable efficacy of these agents in HCC relative to standard therapies(Qin, et al.) (Qin, et al.) (Ren, et al.), and to date, no head-to-head studies have established clinically differences in efficacy among them in HCC.

Moreover, we compared postoperative pathological tumor residual rates among patients treated with the three different PD-1 inhibitors and found no statistically significant differences. This analysis further supports the notion that the specific PD-1 antibody used did not materially influence treatment response in our cohort.

Finally, we note that this study was conducted within the framework of our group's prospectively registered perioperative clinical research program (ClinicalTrials. gov identifier: NCT06571369), which provides standardized clinical evaluation, treatment documentation, and pathological assessment across patients, further mitigating variability related to treatment administration. Taken together, these considerations suggest that the heterogeneity of PD-1 inhibitors used in this study is unlikely to bias our key findings linking lipid-associated macrophages to immunotherapy response.

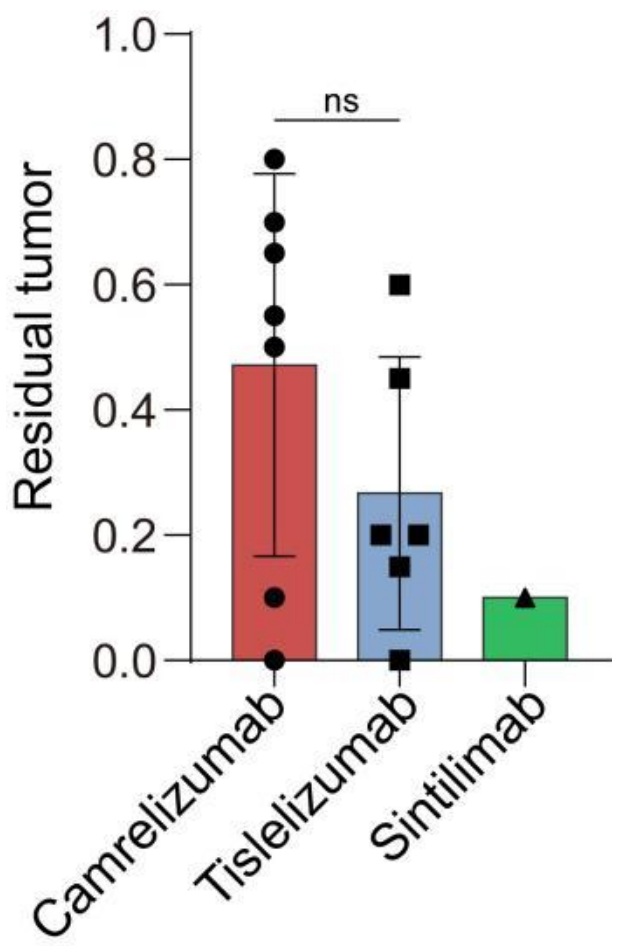

Postoperative pathological tumor residual rates among 14 HCC patients from our immunotherapy cohort that treated with camrelizumab, tislelizumab or sintilimab.

Reference:

Qin S, Chan SL, Gu S, Bai Y, Ren Z, et al. Camrelizumab plus rivoceranib versus sorafenib as first-line therapy for unresectable hepatocellular carcinoma (CARES-310): a randomised, open-label, international phase 3 study. Lancet. 2023 Sep 30;402(10408):1133-1146.

Qin S, Kudo M, Meyer T, Bai Y, Guo Y, et al. Tislelizumab vs Sorafenib as First-Line Treatment for Unresectable Hepatocellular Carcinoma: A Phase 3 Randomized Clinical Trial. JAMA Oncol. 2023 Dec 1;9(12):1651-1659.

Ren Z, Xu J, Bai Y, Xu A, Cang S, et al. Sintilimab plus a bevacizumab biosimilar (IBI305) versus sorafenib in unresectable hepatocellular carcinoma (ORIENT-32): a randomised, open-label, phase 2-3 study. Lancet Oncol. 2021 Jul;22(7):977-990.

3. The scRNA-seq datasets referenced in the manuscript sum to 60, not 61 samples as stated (e.g., GSE156625 in ref. 23 includes 14 samples). Please reconcile this discrepancy and provide a detailed table summarizing each dataset, including sample size, treatment status, and clinical context.

Author's response: We thank the reviewer for pointing out the discrepancy in the sample count. Upon re-checking the datasets, we found that the miscount resulted from discontinuous patient ID in the GSE156625 dataset, which led us to mistakenly overcount the total number of samples. We have corrected the patient number in the revised manuscript accordingly (line 35, 101 and 131). In addition, we have summarized clinical data of in housed and external datasets in Supplementary Table 2.

4. The datasets used are heterogeneous in terms of treatment status, disease stage, etiology (e.g., MASH-related HCC), and other clinical variables. Did the authors observe any associations between MacroLA frequency or HCC_FA subtype and these clinical parameters beyond response status? This is a very relevant aspect to address since MASH disease is highly associated with hepatic lipids accumulation and fatty acid metabolism.

Author's response: We thank the reviewer for raising this important point regarding cohort heterogeneity and the potential influence of clinical variables related to lipid metabolism, including MASH-related HCC. We agree that disease etiology and metabolic background, especially MASH are highly relevant factors to consider when interpreting the enrichment of lipid-associated macrophages (LAMs) and fatty acid–associated tumor states(Chan, et al.).

In response, we have systematically examined the associations between LAM abundance and available baseline clinical parameters beyond treatment response, including liver disease etiology, presence and degree of hepatic steatosis, and body mass index (BMI). As now detailed in the revised Supplementary Table 1 and 2, a subset of patients in our cohort exhibited mild hepatic steatosis at baseline, and none met diagnostic criteria for NAFLD/NASH or MASH-related HCC.

Importantly, correlation analyses revealed that LAM frequency showed only a weak and non-significant association with BMI, suggesting that LAM enrichment in non-responders cannot be simply explained by systemic metabolic status or underlying liver etiology. Instead, LAM abundance demonstrated a much stronger and more consistent association with immunotherapy response status. Similar results were observed for the HCC_FA transcriptional subtype, which correlated with treatment outcome but not with baseline metabolic parameters. These results are added in Supplementary Fig. 6a and below, we have also added corresponding discussion to our revised manuscript in line 673 to 676.

These findings indicate that, in our cohort, LAM enrichment and fatty acid–associated tumor programs are more closely linked to the immunotherapy-shaped tumor immune microenvironment than to pre-existing metabolic disease alone.

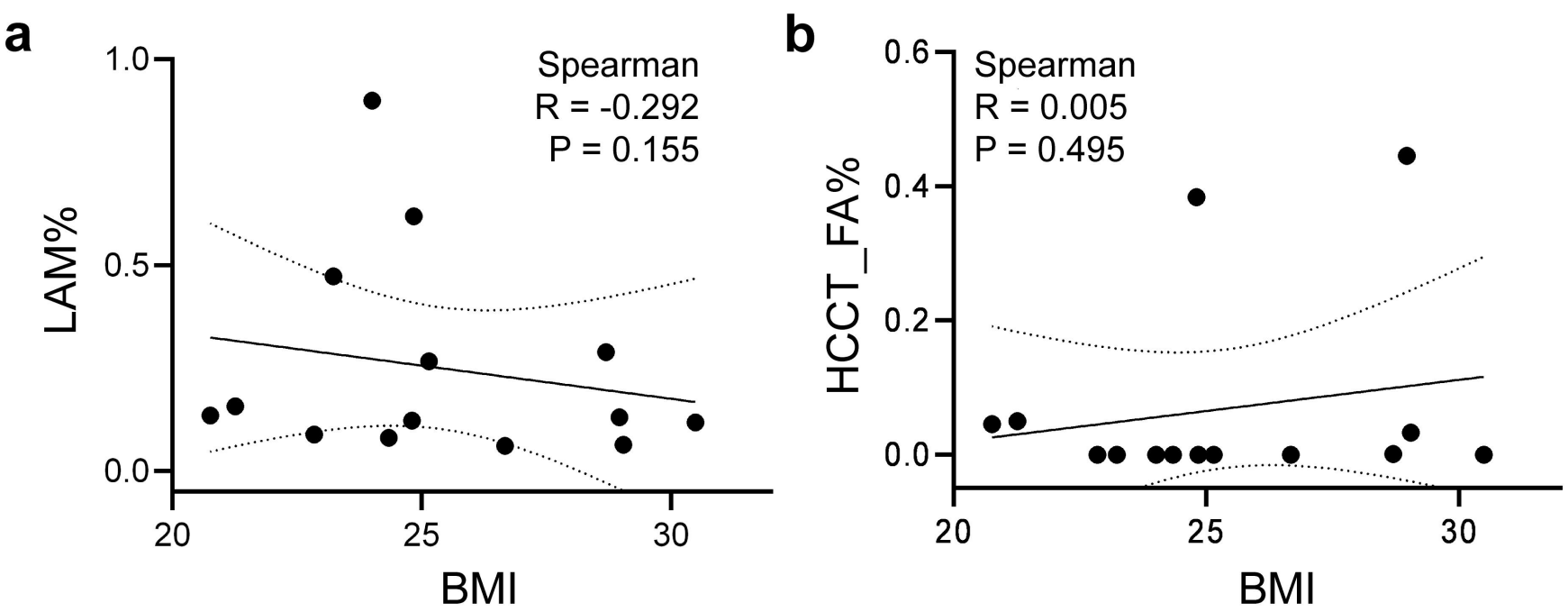

Correlation analysis between BMI and LAM, HCCT_FA proportion. a Correlation analysis between proportion of LAM in myeloid cells and BMI of each patient. **b** Correlation analysis between proportion of HCCT_FA in HCC cells and BMI of each patient.

Reference:

Chan SL, Sun HC, Xu Y, Zeng H, El-Serag HB, et al. The Lancet Commission on addressing the global hepatocellular carcinoma burden: comprehensive strategies from prevention to treatment. Lancet. 2025 Aug 16;406(10504):731-778.

5. Have the authors examined whether pre-defined signatures previously associated with immunotherapy response in HCC (ex. Cappuyns, S., et al. Single-cell RNA sequencing-derived signatures define response patterns

to atezolizumab + bevacizumab in advanced hepatocellular carcinoma. J Hepatol (2024)) are validated in their cohort?

Author's response: We thank the reviewer for raising research focusing on HCC immunotherapy. Notably, several of our findings are generally consistent with the single-cell–derived signatures reported by Cappuyns et al. (J Hepatol 2024), further supporting the robustness and generalizability of our conclusions, yet the core conclusions are partially different.

First, both studies highlight the pivotal role of CD8⁺ T cell functional states in determining immunotherapy response. In our cohort, responders exhibited a higher abundance of activated and functionally competent CD8⁺ T cells, whereas non-responders showed exhausted or metabolically dysregulated T cell states. Notably, CD8⁺ cytotoxic T cells (CD8⁺ Tcyto) and CD4⁺ helper T cells were detected in a subset of patients and displayed a positive association with therapeutic efficacy in our dataset, suggesting that these T cells necessarily confer benefit in the neoadjuvant immunotherapy setting. Correspondingly, Cappuyns et al. reported enrichment of CD8 Temra and CD8 Tex effector populations in responders, accompanied by up-regulation of key effector molecules such as IFNG, GZMB, and CXCL9/10.

Second, both datasets underscore the importance of pro-inflammatory macrophage subsets in supporting effective antitumor immunity. In our study, inflammatory and antitumor macrophage phenotypes were more active in responders, while Cappuyns et al. specifically identified CXCL10⁺ macrophages as a defining feature of treatment-sensitive tumors.

Third, both studies consistently identify immunosuppressive macrophages as key drivers of treatment resistance. Cappuyns et al. demonstrated that TREM2⁺ macrophages and CD14⁺ monocytes constitute major resistant cell states in atezolizumab–bevacizumab non-responders. However, we observed a marked accumulation of lipid-associated macrophages (LAM) in non-responders, characterized by metabolic reprogramming and immunosuppressive activity.

Together, these findings across independent cohorts and therapeutic regimens reinforce the conclusion that the interplay between macrophage functional states and CD8⁺ T cell activity is central to shaping immunotherapy outcomes in HCC.

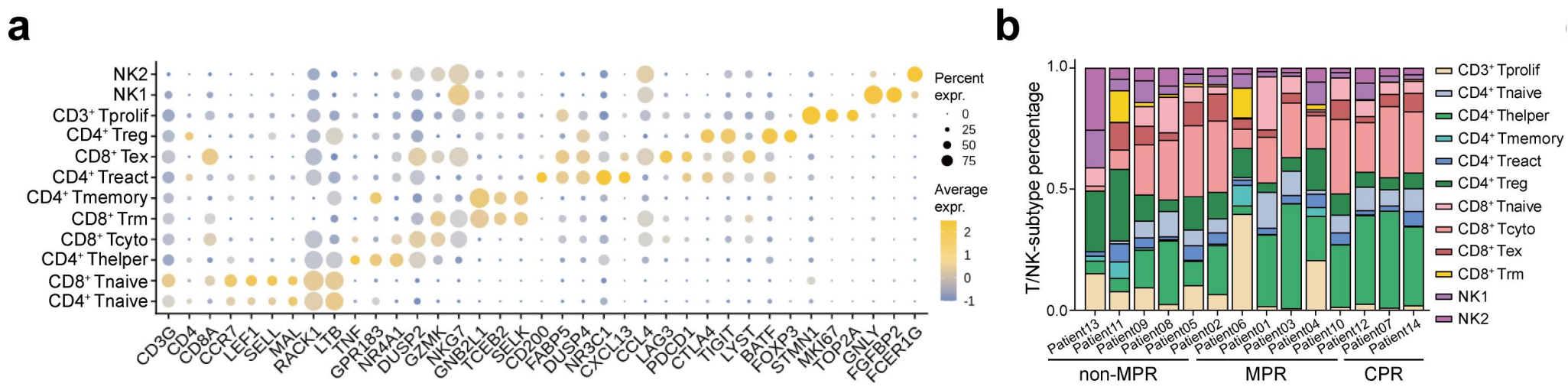

[editorial note: third party material redacted]

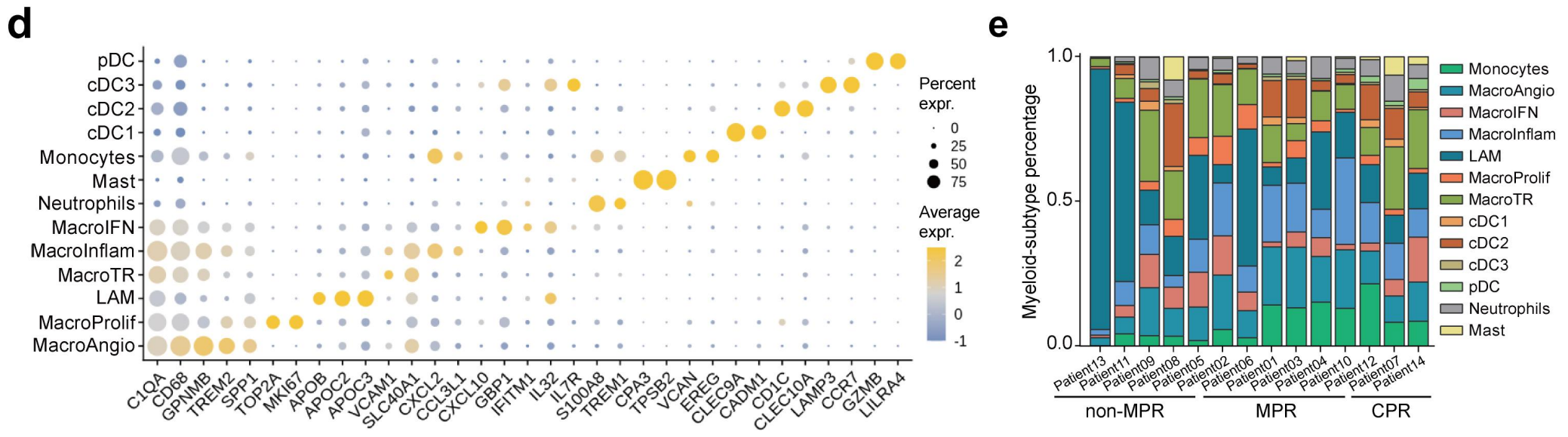

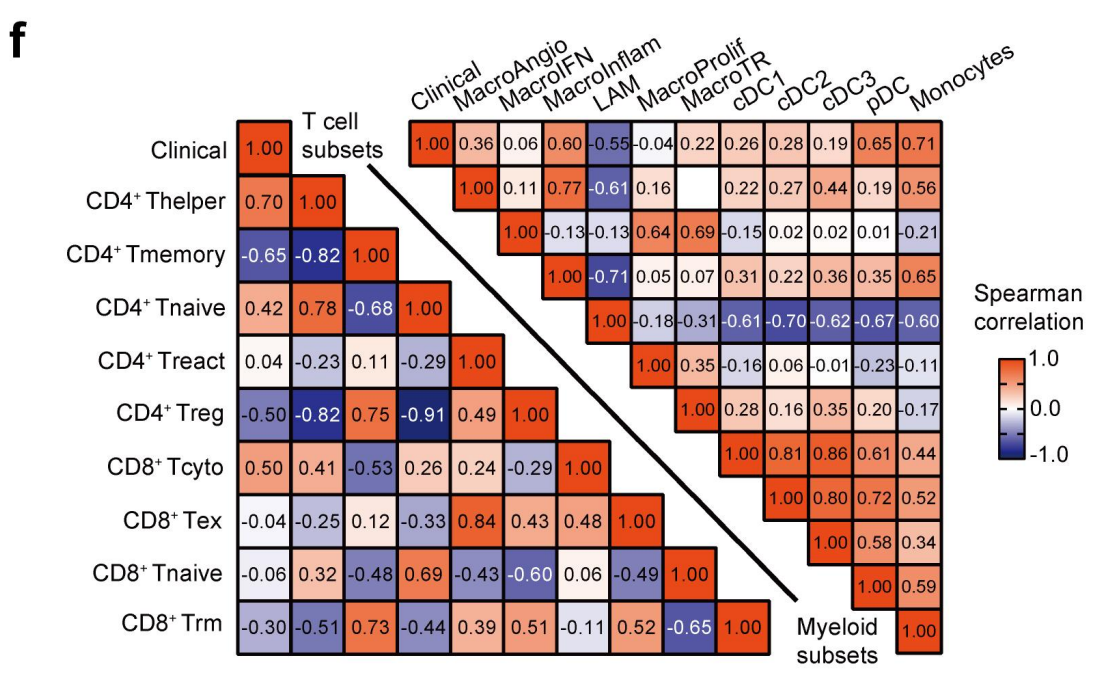

[editorial note: third party material redacted]

Cellular heterogeneity and clinical associations of T/NK and myeloid subtypes in HCC, from our cohort and Cappuyns, et al. a Gene expression features of 12 T/NK subtypes. **b** Bar plots indicating the proportion of T/NK subtypes among 14 patients. **c** Top: Enrichment score of effector T cells (CD8 Temra, CD8Tex) and inflammatory macrophages between responders and non-responders in Cappuyns, et al. research. Bottom: Receiver-operating characteristic curves showing the performance of each signature in predicting response to atezolizumab–bevacizumab in Cappuyns, et al. research. **d** Gene expression features of 13 myeloid subtypes. **e** Bar plots indicating the proportion of

myeloid subtypes among 14 patients. **f** Matrix of Spearman correlation analysis across clinical outcomes and proportions of T cell subtypes and macrophage subtypes. **g** Enrichment score and relations to $CD8^{+}$T cells and pro-inflammatory macrophages of $CD14^{+}$ monocytes and $TREM2^{+}$ macrophage in Cappuyns, et al. cohort.

6. In Figure S1C, all datasets show consistent TIME composition except for GSE189903, which has fewer HCC cells and a higher proportion of T/NK cells. Do the authors attribute this to technical differences in tissue dissociation or biological differences (e.g., tumor reactivity or early-stage disease)? Could this hypothesis be supported by the similarity with the CPR cohort shown in Figure S1F?

Author's response: We thank the reviewer for this insightful comment. As noted, GSE189903 indeed shows a lower proportion of malignant hepatocytes and a higher proportion of T/NK cells compared with the other datasets. We would like to clarify that GSE189903 is an untreated cohort without available response information, and therefore reflects baseline TIME characteristics rather than therapy-induced remodeling.

Importantly, GSE189903 performed multi-regional sampling of tumor core and tumor margin, and this design provides a strong biological explanation for the observed immune enriched composition (Ma, et al.). Tumor margins are well known to harbor higher densities of immune cells and fewer malignant hepatocytes compared with tumor cores (Li, et al.). Because the dataset includes multiple peripheral samples, the averaged single-cell composition naturally shifts toward an immune-infiltrated profile with fewer tumor epithelial cells.

This explains why GSE189903 shows a pattern similar to the CPR cohort in Supplementary Fig. 1f, which likewise represents untreated samples with strong baseline immune infiltration. Thus, the T/NK-rich profile of GSE189903 is consistent with both its multi-regional sampling strategy and biological context, rather than reflecting technical artifacts.

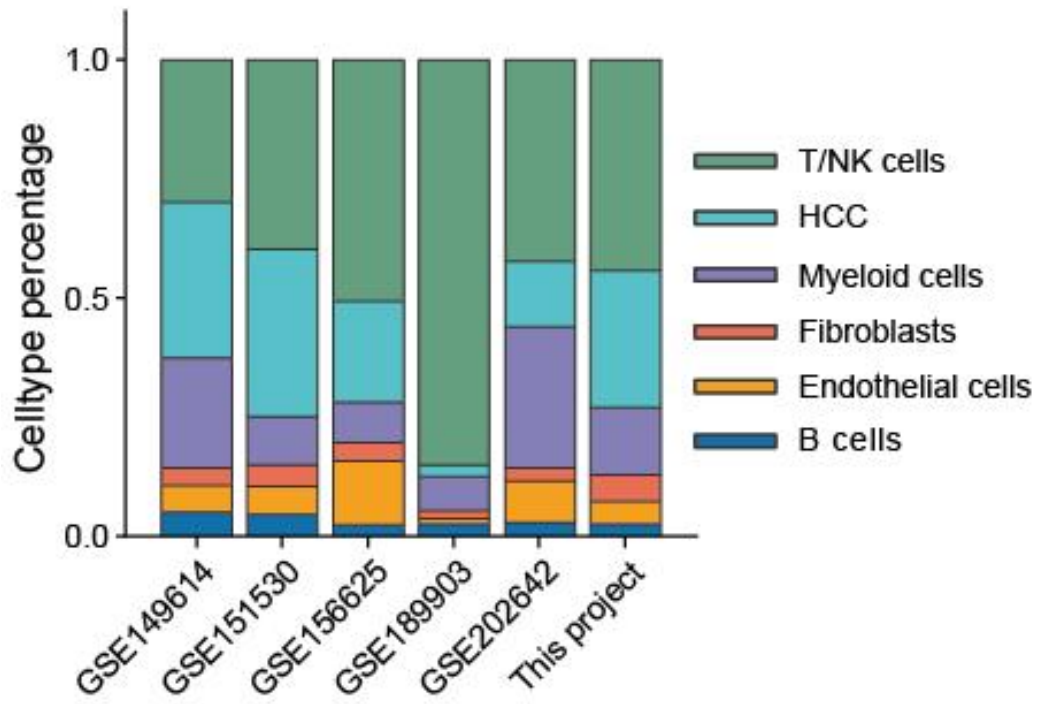

Bar plots indicating the proportion of major cell types in each dataset.

Reference:

Ma L, Heinrich S, Wang L, Keggenhoff FL et al. Multiregional single-cell dissection of tumor and immune cells reveals stable lock-and-key features in liver cancer. Nat Commun 2022 Dec 7;13(1):7533.

Li Z, Hu Y, He Z, Xu H, Wang H, He Y. Comparative transcriptomic and genomic analysis of tumor cells in the marginal and center regions of tumor nests in human hepatocellular carcinoma. Front Cell Dev Biol. 2025 Jul 16;13:1611951.

7. In Figure 2I, the authors validate their findings using two independent scRNA-seq datasets. Why were these not included in earlier integrative analyses? Sample sizes of each validation cohort should be clearly stated.

Author's response: We thank the reviewer for this helpful comment. To independently validate our findings, we analyzed two external scRNA-seq datasets from immunotherapy-treated HCC patients. These datasets were intentionally kept separate from our in house cohort and were not included in the integrative analysis. In contrast, only treatment-naïve external HCC datasets were integrated to establish robust cell type annotations. Specifically:

First, we included multiple treatment-naïve HCC scRNA-seq datasets as an integrative reference cohort in Fig. 1a. The primary purpose of this integration was to establish a robust transcriptional framework for cell type annotation, particularly for accurately identifying immune and macrophage subpopulations, including relatively rare cell states, in the context of large-scale background data. These datasets were not intended for treatment-response analysis, as most lacked immunotherapy exposure or uniform clinical response information.

In contrast, the two independent scRNA-seq datasets (GSE206325: R=8 NR=21, skrx2fz79n: R=3 NR=8) presented in Fig. 2i were intentionally designated as validation cohorts. A key distinguishing feature of these datasets is that all patients had received anti–PD-1 immunotherapy, making them directly relevant for evaluating whether the TIME features identified in our discovery cohort are reproducible in independent, clinically comparable settings. For this reason, these datasets were not included in the initial integrative analysis, but instead analyzed separately to specifically test the robustness of our response-associated findings.

By keeping the validation cohorts independent from the discovery and annotation pipeline, we aimed to avoid analytical circularity and ensure that the association between LAM abundance and immunotherapy outcome could be unbiasedly and independently validated across external PD-1–treated cohorts. Additionally, we have now clearly stated the sample sizes and clinical context of each validation dataset in the revised Methods section (line 897 to 903) as requested.

Public scRNA-seq and spatial transcriptome datasets

Publicly available single-cell RNA-seq (scRNA-seq) and spatial transcriptomics (ST) datasets were used for reference and validation analyses. ST data were obtained from Mendeley Data (skrx2fz79n). Treatment-naïve HCC scRNA-seq datasets were retrieved from GEO (GSE149614, GSE151530, GSE156625, GSE189903, and GSE202642). scRNA-seq datasets from anti－PD-1－treated HCC patients were obtained from GSE206325 and Mendeley Data (skrx2fz79n).

8. In Figures 3A and S3A, 12 T/NK clusters are identified. The manuscript states that clustering was recapitulated only in the authors' own cohort. Does this mean the clustering was not reproducible in external datasets, or that these datasets were not analyzed in other datasets?

Author's response: We thank the reviewer for this helpful comment. To clarify, the integrated dataset consists of our and external scRNA-seq datasets were used for precise identification of 12 T/NK subtypes initially, whereas the downstream analyses were performed exclusively in our cohort, including pseudotime analyses through Monocle approaches.

Specifically, in Fig. 3a, T/NK cells from our cohort were analyzed in the context of the integrated reference framework, and the major T/NK subtypes were identified based on conserved transcriptional programs observed across datasets.

In Supplementary Fig. 3a, we performed an additional analysis in which T/NK cells from our in house cohort were re-clustered independently, without integration with external datasets. The purpose of this analysis was not to replace or supersede the integrative analysis, but rather to double-check the robustness and accuracy of subtype identification within our own data. Importantly, this independent re-clustering yielded similarly well-defined subtype boundaries, demonstrating that the identified T/NK subtypes are stable and reproducible even when analyzed in isolation.

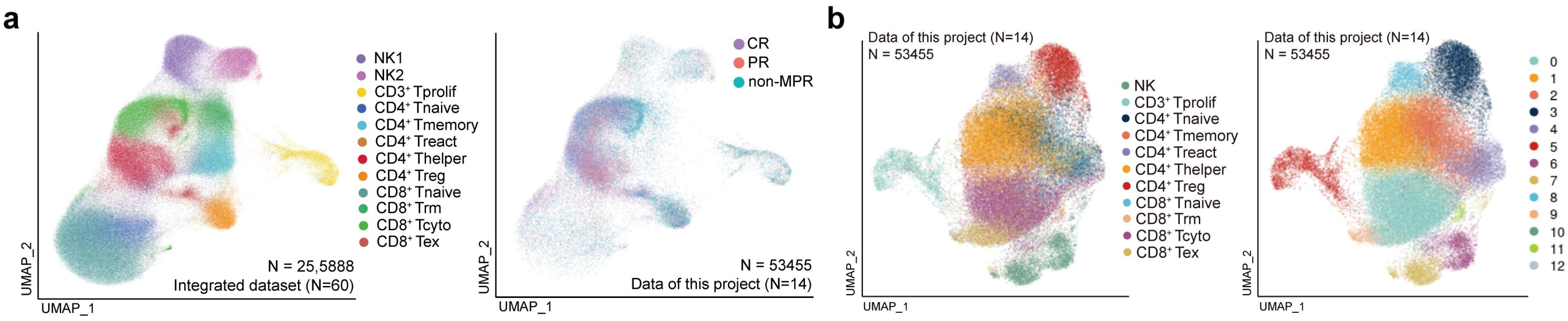

Clustering of T/NK cells from integrated data and in house data. a UMAP plots of T/NK cells from the integrated single-cell atlas which identified into 12 subtypes, and the T/NK cells from our HCC immunotherapy cohort labeled with clinical efficiency. **b** UMAP plots showing the re-clustered T/NK cells from in house HCC immunotherapy cohort, colored by subtypes and unsupervised clusters.

9. High naïve CD8⁺ T cell frequency could reflect poor antigen exposure and impaired effector function. Differences in naïve CD8⁺ T cell proportions have been linked to response in prior studies. Was a similar association observed in this study?

Author's response: We thank the reviewer for this important comment. In light of the reviewer's suggestion, we specifically examined the association between naïve CD8⁺ T cell abundance and treatment response in our cohort. At the

sample level, the correlation between the naïve CD8+ T cell proportion and immunotherapy efficacy score was very weak (Spearman correlation, r = –0.06), indicating no meaningful association in our dataset.

Within the tumor microenvironment, naïve CD8+ T cells constitute only a minor fraction (6.8% of T cells in our cohort) compared with effector, memory, and exhausted CD8+ subsets. In our cohort, the major determinants of response appeared to be the functional states of effector/exhausted CD8+ T cells and the abundance of immunosuppressive macrophages (LAMs), which exert much stronger effects on treatment efficacy. Notably, LAMs are associated with metabolic reprogramming, inhibitory cytokine signaling, and impaired T cell activation, thereby contributing to a suppressive immune environment that limits effective antitumor T cell responses.

10. Page 7, lines 265–269 refer to several markers (TNF, IL7R, PDCD1, LAG3, FOXP3), but only IL7R and PDCD1 are shown in Figure S3C. Please ensure that all highlighted markers are included in the figures referenced. A similar issue occurs in Figure 4F–G and S4C for macrophage markers.

Author's response: We thank the reviewer for pointing out the inconsistency between the markers listed in the text and those displayed in the corresponding figures. As noted, lines 264 to 266 refers to several markers (*TNF*, *IL7R*, *PDCD1*, *LAG3*, *FOXP3*), while Supplementary Fig. 3c only shows *IL7R* and *PDCD1*.

To address this, we have revised Supplementary Fig. 3c to include all markers mentioned in the text, namely *TNF*, *LAG3*, and *FOXP3*, in addition to *IL7R* and *PDCD1*, to ensure full consistency between the manuscript and the figures.

A similar issue was identified for macrophage markers in Fig. 4f and Fig. 4g. We will likewise update these figures to incorporate the complete set of macrophage markers referenced in the Results, so that all highlighted genes are visually presented in the corresponding panels (Supplementary Fig. 3c, Supplementary Fig. 4b).

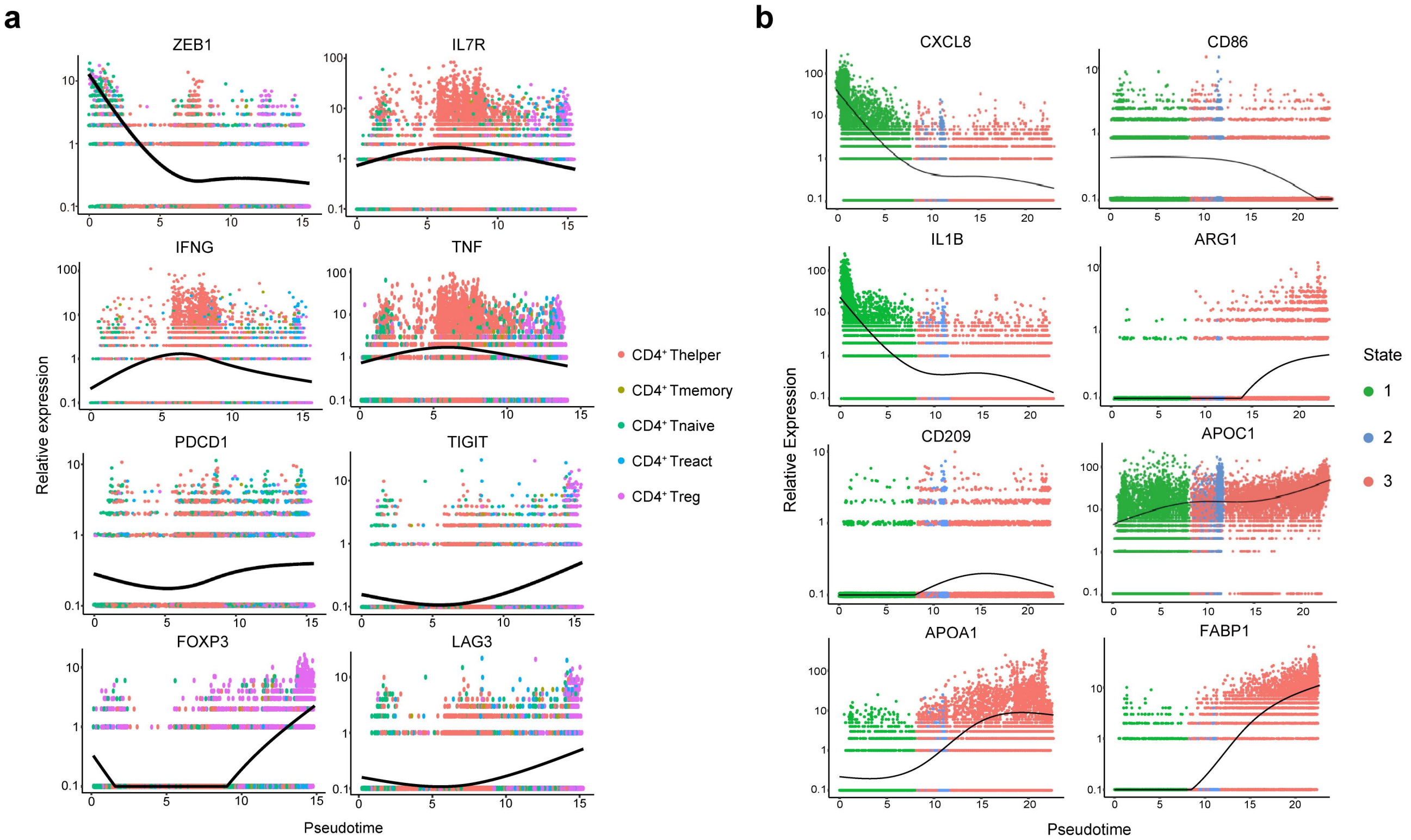

The expression changing along the pseudotime of CD4+T and macrophage characteristic genes. a Scatter plots exhibited the expression changing of genes along the pseudotime related to CD4+T cell naïve (*ZEB1*, *IL7R*) effect (*IFNG*, *TNF*) and exhaustion (*PDCD1*, *TIGIT*, *FOXP3*, *LAG3*). **b** The change in expression levels of M1 (*CXCL8*, *CD86*, *IL1B*), M2 (*ARG1*, *CD209*) and lipid-associated (*APOC1*, *APOA1*, *FABP1*) genes in macrophage along the axis of pseudotime.

11. In Figure 3J, antigen presentation-related pathways are enriched in non-responders, which is unexpected in T cells (which typically do not present antigen but recognize). Could the authors comment on whether this is an artifact of pathway annotation or if it reflects aberrant gene expression signatures in T cells?

15. Figure 7C shows differential expression of MHC-I and MHC-II molecules between R and NR. Given the relevance of antigen presentation in immunotherapy response, a deeper discussion of these findings is warranted.

(This may be discussed together with my prior comment #11)

Author's response: We thank the reviewer for this insightful comment. We agree that classical antigen presentation is not a canonical function of T cells, and therefore enrichment of antigen presentation–related pathways in the T cell compartment may appear unexpected.

Upon closer examination, we believe this observation reflects annotation-related pathway overlap rather than true antigen-presenting activity by T cells, for the following reasons:

Many "antigen presentation" pathways include genes involved in IFN-γ response (Zhou, et al.), such as HLA-A/B/C, B2M, TAP1, TAPBP, which are broadly upregulated in IFN-γ treated cell. Thus, pathway enrichment does not imply that T cells are functioning as antigen-presenting cells, but rather that they express MHC–associated genes as part of inflammatory response.

In our dataset, the T cell clusters enriched in non-responders indeed include several MHC class II–related genes, such as HLA-DRA, HLA-DQA1, and HLA-DRB5. Besides these T cell also display highly dysfunction markers, suggesting that the antigen presentation signatures likely reflect aberrant or compensatory transcriptional programs induced by chronic stimulation rather than APC-like behavior. This pattern is consistent with prior scRNA-seq study (Wang, et al.), which has shown that exhausted T cells can exhibit up-regulation of MHC-related transcripts, without acquiring functional antigen-presenting capacity.

In addition, ligand–receptor analyses in Fig. 8e and Supplementary Fig. 8d further support this interpretation. As receiver, the interactions involving MHC molecules (e.g., HLA-E–CD8A and HLA-DRA–CD4) sent by antigen presenting cells reflect T cells acting as recipients of antigen presentation signals rather than as antigen-presenting cells themselves. These interactions are consistent with immune recognition and signaling processes within the TIME, not with antigen presentation performed by T cells. As sender, T cells in the non-responder group exhibited higher expression of MHC-related molecules (e.g., HLA-A, HLA-B, HLA-E, HLA-DPB1 and HLA-DRA), which is consistent with our conclusions described above. Together, the expression of these MHC-associated genes was misinterpreted by the CellChat analysis as indicative of antigen-presenting activity.

Overall, in non-responders, the increased expression of MHC-I and MHC-II modules likely reflects a state of persistent IFN signaling, chronic antigen stimulation, and immune exhaustion rather than enhanced antigen-presenting capacity. This interpretation is supported by the concomitant enrichment of exhausted T cell signatures and LAM, both of which are associated with non-productive inflammation. In contrast, responders exhibit more effective CD8^{+} T cell activation with less chronic stimulation, resulting in lower global HLA transcription yet improved therapeutic efficacy. These findings highlight that high MHC-I/II expression does not necessarily equate to functional antigen presentation in the context of immunotherapy response.

To avoid any misunderstanding that T cells are actively presenting antigen, we will revise pathway naming in manuscript. Instead of labeling this GO module as "Antigen presentation", we will adopt a more biologically accurate descriptor such as: "Exhaustion-associated MHC activation". In addition, we will change the description of interaction about MHC related genes in CellChat analysis from "Antigen" into "MHC related" in Fig. 8e and Supplementary Fig. 8d.

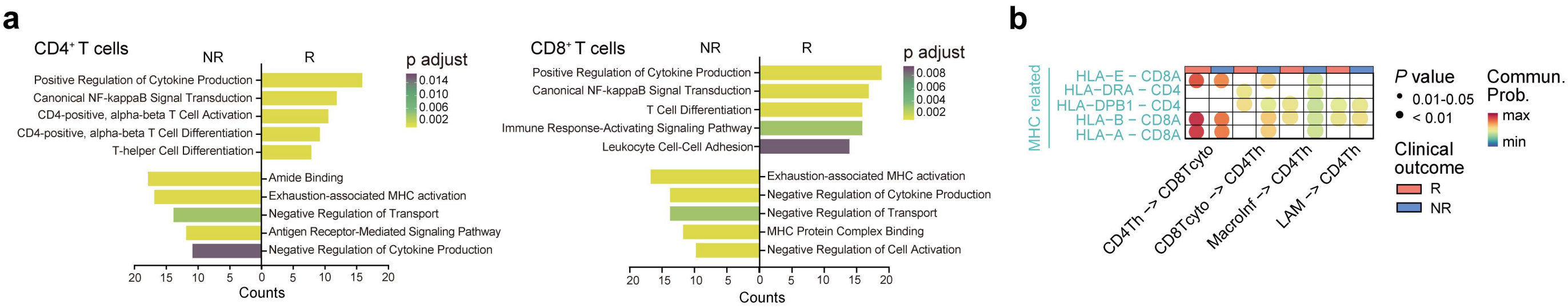

Enrichment and cellchat analysis of HLA-related biological process. **a** Bar charts showing the GO pathway enrichment of specific biological process based on the differentially expressed genes in CD4^{+} T cells and CD8^{+} T cells between responders and non-responders. **b** Bubble plot illustrated the ligand-receptor interaction of MHC related function among critical TIME components, including CD4^{+} Th, CD8^{+} Tcyto, MacroInflam, and LAM of responder and non-responder groups.

Reference:

Zhou F. Molecular mechanisms of IFN-gamma to up-regulate MHC class I antigen processing and presentation. Int Rev Immunol. 2009;28(3-4):239-60.
Wang X, Chen Y, Li Z, Huang B, Xu L, Lai J, Lu Y, Zha X, Liu B, Lan Y, Li Y. Single-Cell RNA-Seq of T Cells in B-ALL Patients Reveals an Exhausted Subset with Remarkable Heterogeneity. Adv Sci (Weinh). 2021 Oct;8(19):e2101447.

12. While macrophage subtypes are well-characterized, the role of other relevant myeloid populations such as MDSCs is not addressed, did authors check if this population was present in their cohort? On the other hand, monocytes appear to be enriched in responders (Figure 5D). Could the authors elaborate on these findings and their potential clinical implications?

Author's response: We appreciate the reviewer's insightful comments. Using canonical markers (IL1B, CCR1, CXCL2, GRINA, IER3) for monocytic and granulocytic MDSCs (Tsutsumi, et al.), we specifically examined whether a distinct MDSC population was present in our cohort. We did not identify a transcriptionally well-defined MDSC cluster. While scattered MDSC-associated genes were expressed in certain myeloid states, these cells did not form a coherent suppressive MDSC module. We therefore conclude that bona fide MDSCs are not prominent in our dataset, which may be related to (i) the neoadjuvant treatment context, (ii) the rapid turnover or depletion of MDSCs after PD-1 blockade, or (iii) the tissue sampling location in post-treatment surgical specimens.

In contrast, monocytes were relatively enriched in responders, as shown in Fig. 5d of the manuscript. This observation is biologically meaningful, because accumulating evidence shows that circulating or tumor-infiltrating monocytes can serve as precursors for antitumor macrophage states (Zhang, et al.), support antigen presentation, and contribute to early T cell activation in immunotherapy (Vermare, et al.).

In our dataset, monocytes in responders expressed higher levels of genes associated with inflammatory activation and immune recruitment, suggesting that these cells may participate in generating a more immunostimulatory myeloid ecosystem that favors PD-1 response. Clinically, this raises the possibility that:

Baseline or early-treatment monocyte signatures may serve as biomarkers for predicting responsiveness to PD-1 therapy.

Enhancing monocyte to antitumor macrophage differentiation could be explored as a potential therapeutic strategy to augment neoadjuvant immunotherapy.

The relative absence of monocytes in non-responders may contribute to the expansion of suppressive macrophage phenotypes (e.g., LAM) and poor immune priming.

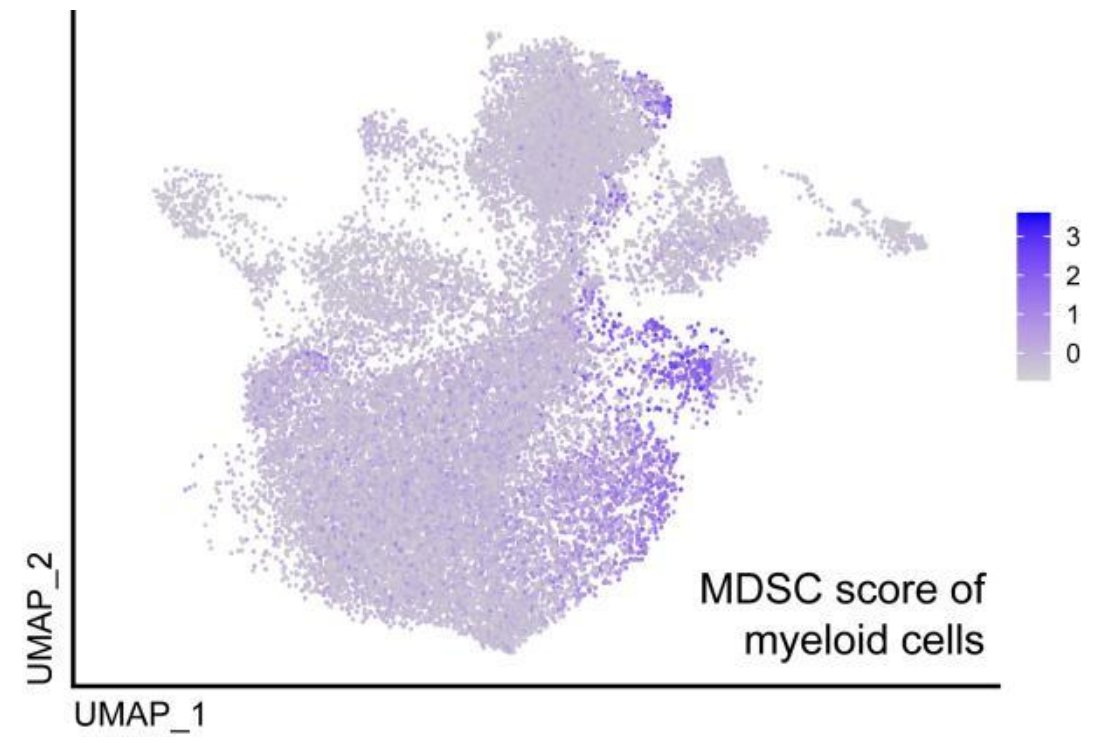

Feature plot showing expression level of MDSC markers among myeloid cells from our dataset.

Reference:

Tsutsumi C, Ohuchida K, Katayama N, Yamada Y, Nakamura S, Okuda S, Otsubo Y, Iwamoto C, Torata N, Horioka K, Shindo K, Mizuuchi Y, Ikenaga N, Nakata K, Nagai E, Morisaki T, Oda Y, Nakamura M. Tumor-infiltrating monocytic myeloid-derived suppressor cells contribute to the development of an immunosuppressive tumor microenvironment in gastric cancer. Gastric Cancer. 2024 Mar;27(2):248-262.
Zhang H, Liu L, Liu J, Dang P, Hu S, Yuan W, Sun Z, Liu Y, Wang C. Roles of tumor-associated macrophages in anti-PD-1/PD-L1 immunotherapy for solid cancers. Mol Cancer. 2023 Mar 21;22(1):58.
Vermare A, Guérin MV, Peranzoni E, Bercovici N. Dynamic CD8+ T Cell Cooperation with Macrophages and Monocytes for Successful Cancer Immunotherapy. Cancers (Basel). 2022 Jul 21;14(14):3546.

13. It would greatly strengthen the manuscript to include histological assessment (e.g., Masson's trichrome, Sirius red, Oil red…) of fibrosis and lipid accumulation to support the proposed functional roles of MacroLA in ECM remodeling and steatosis.

Author's response: We appreciate the reviewer's constructive suggestions. In response, we have expanded our experimental validation using an independent, newly enrolled HCC neoadjuvant immunotherapy cohort (R=5, NR=5), which was specifically collected for additional histological and flow cytometric analyses. The clinical data of these patients were in Supplementary Table 2.

Using this independent cohort, we conducted Masson's trichrome, Sirius Red, and Oil Red O staining to assess extracellular matrix remodeling and lipid accumulation in the tumor microenvironment. Oil Red O staining revealed significantly increased lipid deposition in non-responders, while both Masson's trichrome and Sirius Red staining demonstrated more pronounced fibrosis in the NR group compared with responders.

These histological findings are highly consistent with the transcriptional features of the lipid-associated macrophage (LAM) population identified in our single-cell analysis, which is characterized by up-regulation of genes involved in lipid metabolism and ECM remodeling. Importantly, the use of an independent validation cohort provides orthogonal evidence supporting the functional relevance of LAM in shaping a fibrotic and lipid-enriched tumor microenvironment.

Together, these data strengthen our conclusion that enhanced fibrosis and lipid accumulation which associated with increased LAM abundance may contribute to an immunosuppressive TIME and impaired response to αPD-1 in HCC.

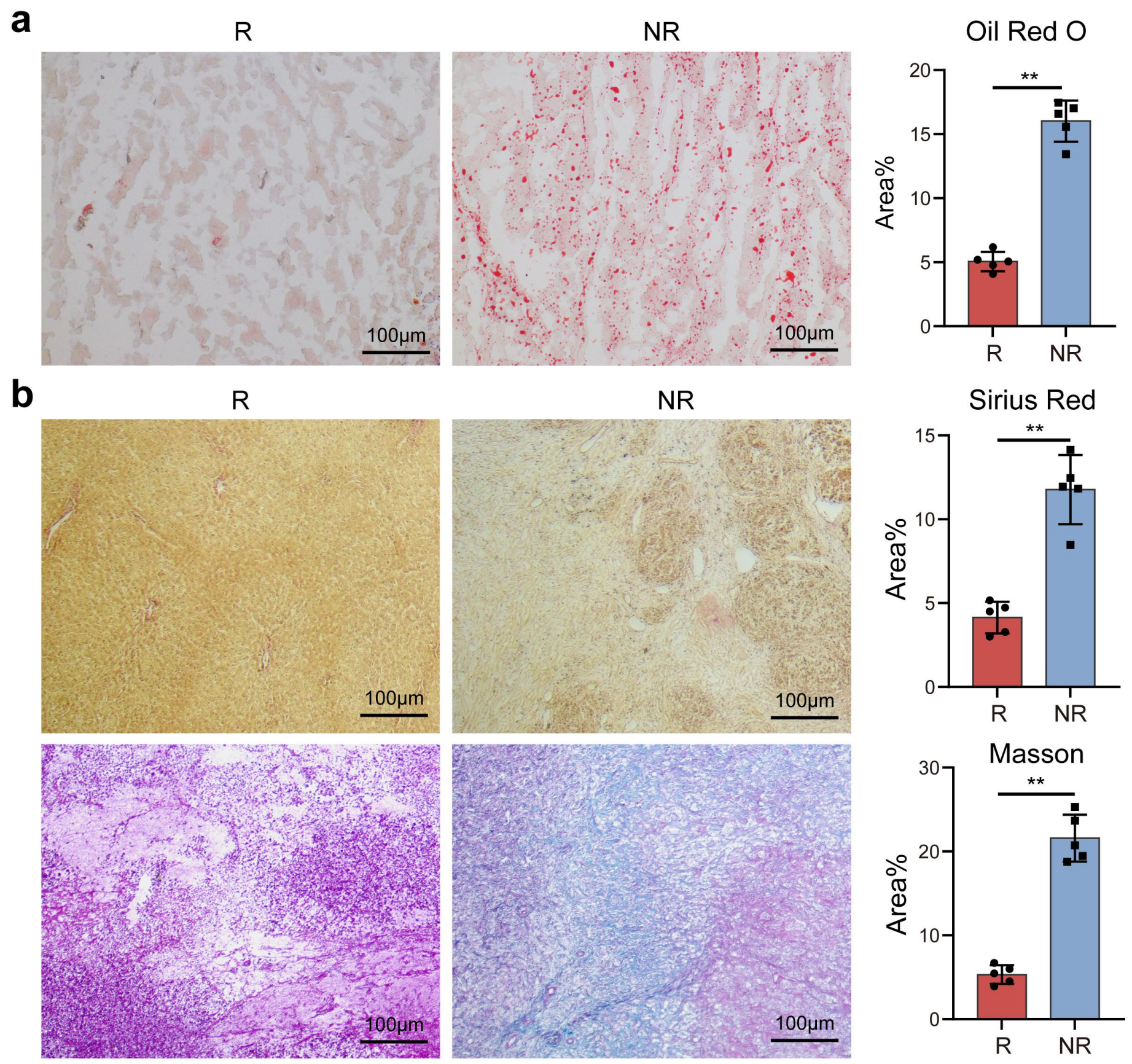

Histological validation of fibrosis and lipid accumulation in responders and non-responders. a Representative Oil Red O staining of tumor regions from responders (R) and non-responders (NR) demonstrating markedly higher levels of lipid droplets in NR samples. **b** Sirius red and Masson's trichrome staining illustrating increased collagen deposition in NR samples. (ImageJ was used to quantify the percentage of lipid/fibrosis positive area in representative fields. Stained regions were thresholded uniformly, and positive area (%) was calculated as ratio of stained pixels to total tissue area.)

14. Including staining from a responder sample in parallel to the non-responders in Figure C would provide a useful comparison. In Figure 6D, adding cytometry data from a responder (as negative control) or including multiple NR samples would improve scientific rigor (N=1 is insufficient). If this additional samples cannot be provided, consider moving these data to Supplementary figure.

Author's response: We appreciate the reviewer's constructive suggestions. In response, we have expanded our experimental validation using an independent, newly enrolled neoadjuvant immunotherapy cohort (R=5, NR=5), which was specifically collected for additional histological and flow cytometric analyses. The clinical data of these patients were detailed in Supplementary Table 2.

For Fig. 6d, we have included flow cytometry data from additional non-responders as well as responders, thereby increasing the rigor of the analysis and addressing the concern regarding sample size. These additional datasets consistently confirm the selective enrichment of the $C1Q^+$ $APOA1^+$ $FABP1^+$ LAM population in NR patients.

Moreover, we have added multiplex immunohistochemistry (mIHC) results in previous cohort from responder (R) samples in parallel with the non-responder (NR) samples in Fig. 6c. This side-by-side comparison more clearly illustrates that NR patients exhibited a greater abundance of $CD68^+$ $APOA1^+$ LAMs, which were in close spatial proximity and interaction with HCC cells. In contrast, macrophages in the R group less frequently expressed APOA1 and commonly collaborated with $GZMB^+$ effector T cells, facilitating more robust infiltration and cytotoxic killing functions.

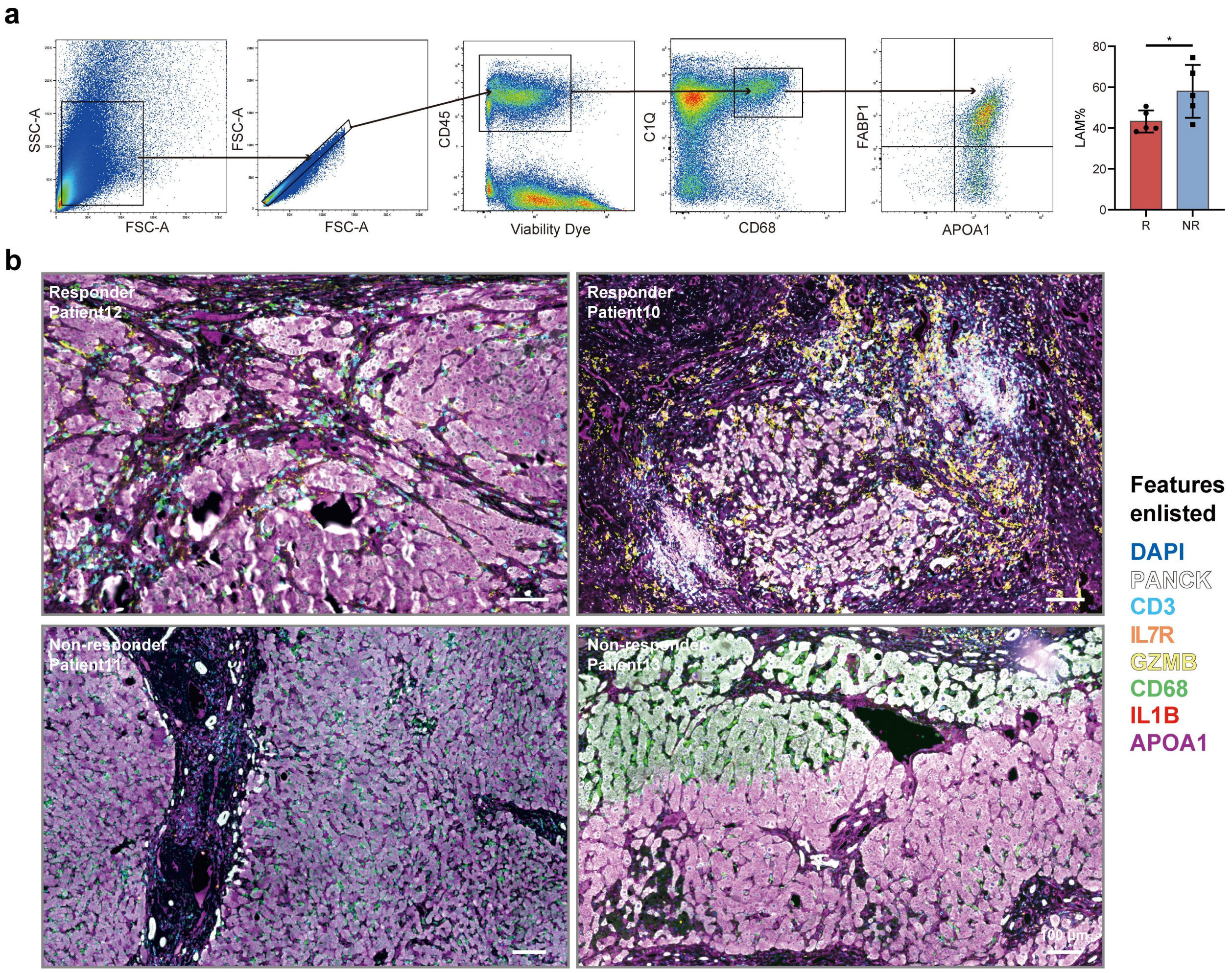

Increased abundance of LAM in non-responders. a Flow cytometry analysis of tumor derived immune cells demonstrates a significantly increased proportion of LAMs in NR patients relative to R patients. **b** Representative mIHC staining of tumor tissues from responders and non-responders. LAMs are identified based on the expression of CD68 and APOA1, while the density of LAMs is significantly higher in non-responders than responders within TIME of HCC.

16. Line 536, Page 13 states that MacroLA promote ECM fibrosis via SPP1 and FN1. This would be better substantiated by direct histological evidence (e.g., fibrosis staining) of induced fibrosis in patients (non-responders) with higher MacroLA.

Author's response: We thank the reviewer for this valuable comment. To substantiate the proposed pro-fibrotic function of LAM, we have now incorporated additional cytometric and histological evidence from the cohort mentioned in question 13 and 14 into the revised manuscript (line 440 to 443, line 611 to 613).

As suggested, we performed Masson's trichrome and Sirius Red staining on tumor tissues to directly assess ECM fibrosis. Importantly, these histological analyses were conducted on the same cohort of patients for whom we quantified

LAM abundance by flow cytometry. This paired analysis revealed a highly consistent pattern that Non-responders (NR) exhibited significantly higher LAM frequencies by flow cytometry, and the same NR patients displayed markedly increased fibrosis, including greater collagen deposition and ECM fiber accumulation, compared with responders (R). Thus, both the LAM proportion and the fibrosis phenotype converge within the same individuals, providing strong orthogonal support for the conclusion that LAM contribute to a pro-fibrotic, immunosuppressive tumor microenvironment. These findings align with the single-cell transcriptomic signatures of LAM, as ECM-remodeling is one of the immunosuppressive functions exerted by LAM, including SPP1 and FN1.

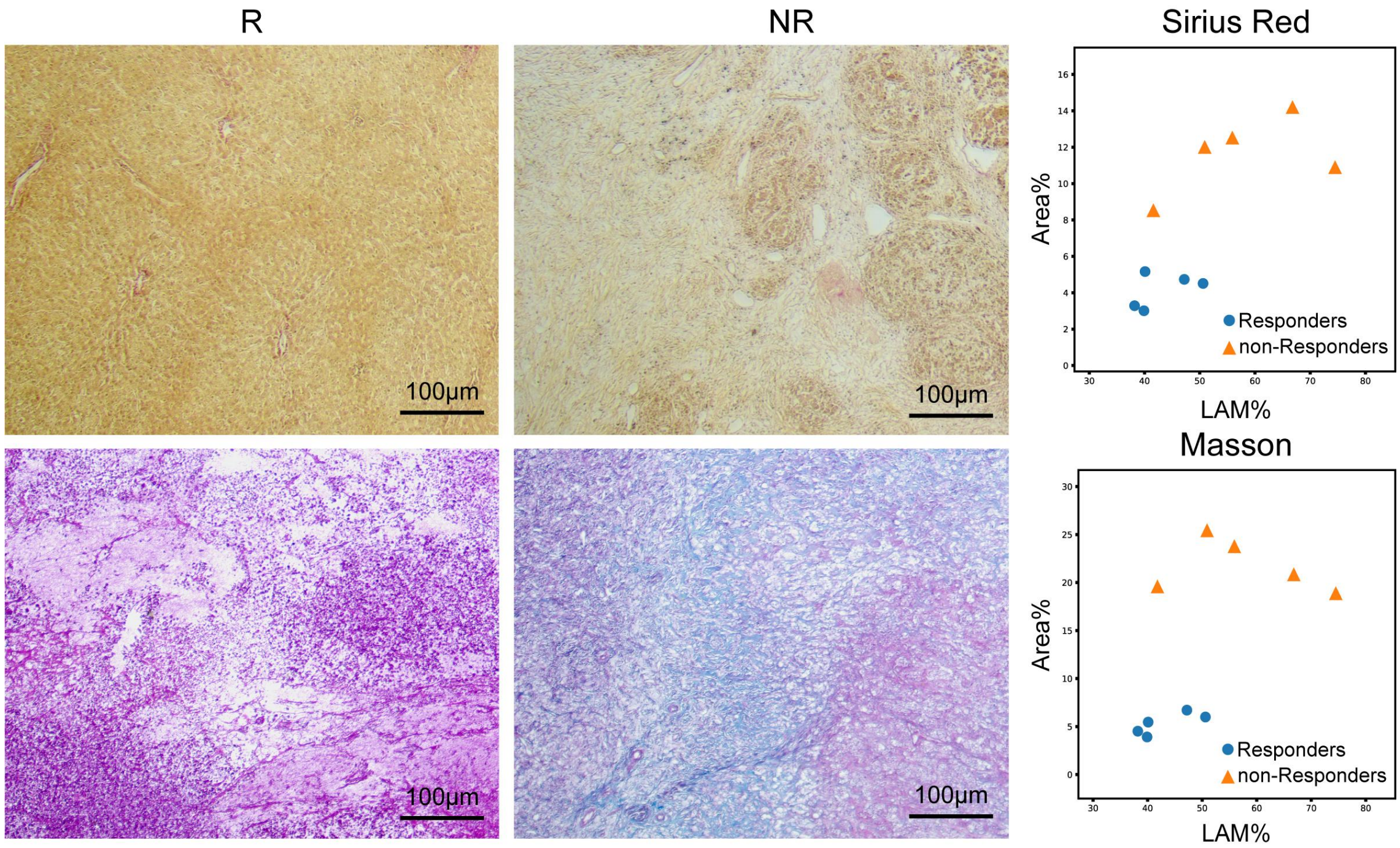

Association of LAM abundance and fibrosis features between responders and non-responders. Scatter plots showing patient-level distributions of LAM proportions and fibrosis staining metrics in responders and non-responders.

Minor Comments:

1. The current title is overly long (29 words) and descriptively dense. A more concise and impactful title is recommended.

Author's response: We appreciate this suggestion and will revise the title to "Integrated Single-cell Multi-omics Characterization Reveals Lipid-associated Macrophages Impairing Neoadjuvant Immunotherapy Efficacy in Hepatocellular Carcinoma".

2. Abstract (Page 1, line 34): Please replace "hepatocellular carcinoma" with the abbreviation "HCC," as the term has already been introduced.

Author's response: We agree with this comment and will replace "hepatocellular carcinoma" with the abbreviation **"HCC"** in the Abstract (line 33), as the term has already been defined earlier.

3. Minor grammatical inconsistencies and typographical errors are present and should be corrected. For example, "respons" (Page 12, line 479) should be "response."

Author's response: We thank the reviewer for pointing out these issues. All identified grammatical inconsistencies and typographical errors, including correcting "respons" to **"**response**"** (line 619), will be carefully revised throughout the manuscript.

4. More details in the statistical methos section or including references of the computational methods may provide stronger transparency and reproducibility.

Author’s response: We agree that additional details would improve transparency and reproducibility. We will expand the Statistical Methods section and, where appropriate, add references to the computational and analytical methods used in this study to provide clearer methodological context (line 904 to 914).

Statistical analysis

*All statistical analyses were performed using R software or GraphPad Prism as appropriate. Data are presented as mean ± standard error of the mean or median with interquartile range, depending on data distribution. Normality was assessed using the Shapiro–Wilk test. Differences in cellular composition between two independent groups were evaluated using the unpaired two-tailed Student’s t test. To assess the association between the relative proportions of all annotated cell types and clinical treatment efficacy, Spearman’s rank correlation coefficient was calculated, given its suitability for evaluating monotonic relationships between continuous or ordinal variables without assuming linearity or normality. Statistical difference results were demonstrated: ns, not significant; *, P < 0.05; **, P < 0.01; ***, P < 0.001.50 (Ibrahimi, et al.).*

Reference:

Ibrahimi E, Wolford BN. Ten essential tips for robust statistics in cell biology. Nat Cell Biol. 2025 Nov;27(11):1884-1886.

5. Title of Results 1 may be revised to "Cohort of Patients with HCC Treated with Immunotherapy" instead of "HCC Immunotherapy Cohort."

Author’s response: We appreciate reviewer’s suggestion on title of Results 1. However, due to limited length requirement for subheadings, we have revised all the structure of subheadings. The title of Result 1 now is “Establish single-cell atlas including immunotherapy-treated HCC patients” (line 116).

Reviewer #3: This manuscript by Peng et al. integrates single-cell transcriptomic data from 14 hepatocellular carcinoma (HCC) patients treated with neoadjuvant PD-1/PD-L1 blockade together with five large publicly available HCC scRNA-seq datasets (GSE149614, GSE151530, GSE156625, GSE189903, GSE202642). The authors identify responder-associated $CD4^+$ and $CD8^+$ T cell states, and highlight a macrophage population termed "MacroLA," enriched in non-responders, which they propose as immunosuppressive. Functional assays using THP-1–derived macrophages, patient-derived organoids, and lycopene supplementation are presented as supporting evidence. Spatial transcriptomic data (PMID: 36708811; Mendeley skrx2fz79n) are also incorporated.
The integration of large public datasets with new single-cell data from a neoadjuvant immunotherapy cohort is a major strength and reflects substantial work. The dataset will likely be useful for the field. However, the manuscript suffers from several critical limitations in terms of novelty, clinical reporting, methodological rigor, macrophage annotation, and functional validation. These issues significantly limit the mechanistic conclusions and translational impact.

Major concerns:

1. The clinical description in the current manuscript is incomplete and requires substantial clarification. Response assessment is presented only using RECIST 1.1 and CPR/MPR categories, but no information is provided on recurrence-free survival (RFS) or overall survival (OS)—both of which are essential endpoints in the neoadjuvant immunotherapy setting and are routinely reported in comparable studies. In addition, patient demographics and critical treatment details are missing or insufficiently described. Specifically, the manuscript does not provide: Major inclusion and exclusion criteria for trial enrollment. The exact PD-1 inhibitor used for each patient (tislelizumab or camrelizumab). Dosing schedule, number of cycles, and whether the regimens were standardized across patients. The interval from last dose to surgery, whether any perioperative delays occurred, and how long patients remained on treatment prior to surgery. Reporting of treatment-emergent adverse events (AEs) or surgical complications, which are crucial for evaluating feasibility and safety in the neoadjuvant setting. Moreover, the pathological response definitions are unconventional. The authors classify CPR = 0% residual tumor and MPR < 50% residual tumor, which are unusually lenient. Established neoadjuvant criteria in solid tumors (including HCC) typically use ≤10 – 30% residual viable tumor as the cutoff for MPR. The rationale for adopting a less stringent threshold is not justified. Sensitivity analyses using widely accepted cutoffs should be provided, and the justification for the authors' chosen thresholds must be explicitly stated. These omissions limit the interpretability and clinical relevance of the reported findings. At minimum, the missing details should be provided in the Supplementary Information to allow readers to properly contextualize the results and assess their reproducibility in relation to existing neoadjuvant immunotherapy trials.

Author's response: We thank the reviewer for this comprehensive and constructive critique regarding the clinical characterization of our cohort. We fully agree that clear reporting of clinical endpoints, patient characteristics, and treatment details is essential for appropriate interpretation and contextualization of neoadjuvant immunotherapy studies.
In response to these comments, we have taken the following steps in the revised manuscript. First, the major inclusion and exclusion criteria for patient enrollment have now been explicitly added to the Methods section (line 711 to 748) to clearly define the clinical context and eligibility of the study population which were also attached below.
Second, all remaining clinical details raised by the reviewer (including patient demographics, the RFS and OS, the specific PD-1 inhibitor administered to each patient, dosing schedules and number of treatment cycles, the interval between the last dose and surgery, perioperative considerations, and treatment-emergent adverse events or surgical complications) have been systematically compiled and provided in the Supplementary Table 1. This allows transparent access to the full clinical metadata without overloading the main text.
For pathological response categorization, we acknowledge that cutoff values for MPR can vary across tumor types and clinical trials (Llovet, et al.) (D'Alessio, et al.). In this study, our classification strategy follows 2024 Chinese clinical guidelines for hepatocellular carcinoma in which patients were classified as non- major pathological response (non-MPR), major pathological response (MPR), or complete pathological response (CPR) based on residual viable tumor percentages of >=50%, 0–50%, and 0%, respectively (Zhou, et al.).

Supplementary Table 1. Patient treatment line of our scRNA-seq cohort

Sample_ID	Pathology	Resource	Sex	Age	Other disease	BMI	PD-1 antibody	Dose Schedule	Cycles	Interval from Last Dose to Surgery (Days)	Perioperative Delays
Patient01	HCC	Post_surgery	Female	47	HBV	26.67	Tislelizumab	200mg q3w	3	24	NO
Patient02	HCC	Post_surgery	Male	41	HBV	29.04	Camrelizumab	200mg q3w	3	41	NO
Patient03	HCC	Post_surgery	Male	56	HBV	22.84	Tislelizumab	200mg q3w	5	47	NO
Patient04	HCC	Post_surgery	Male	28	HBV	25.14	Tislelizumab	200mg q3w	5	54	NO
Patient05	HCC	Post_surgery	Male	41	HBV	28.69	Camrelizumab	200mg q3w	3	40	YES
Patient06	HCC	Post_surgery	Female	55	HBV+Fatty liver	23.23	Tislelizumab	200mg q3w	5	32	NO
Patient07	HCC	Post_surgery	Male	67	HBV	24.34	Tislelizumab	200mg q3w	5	27	NO
Patient08	HCC	Post_surgery	Male	56	HBV	20.76	Tislelizumab	200mg q3w	5	27	NO
Patient09	HCC	Post_surgery	Male	59	HBV	30.49	Camrelizumab	200mg q3w	4	39	NO
Patient10	HCC	Post_surgery	Male	45	HBV+Fatty liver	21.26	Camrelizumab	200mg q3w	5	38	NO
Patient11	HCC	Post_surgery	Male	65	HBV	24.84	Camrelizumab	200mg q3w	5	44	NO
Patient12	HCC	Post_surgery	Male	53	HBV+Fatty liver	28.96	Sintilimab	200mg q3w	5	27	YES
Patient13	HCC	Post_surgery	Male	63	HBV+Fatty liver	24.00	Camrelizumab	200mg q3w	3	34	NO
Patient14	HCC	Post_surgery	Male	66	HBV	24.80	Camrelizumab	200mg q3w	3	34	NO

Sample_ID	Reasons for Perioperative delay	Treatment period	Adverse Events Level	Adverse Events	Surgical Complications	RECIST	Pathologic response	Efficacy group	Residual tumor	RFS	OS
Patient01	/	9w	/	/	NO	PR	MPR	Responder	0.2	62	420
Patient02	/	9w	/	/	NO	SD	non-MPR	Non-responder	0.5	131	1032
Patient03	/	15w	2	Transaminase Elevation	NO	SD	MPR	Responder	0.2	792	992
Patient04	/	15w	2	Proteinuria	NO	/	MPR	Responder	0.15	34	573
Patient05	Hypoproteinemia	9w	3	Hypoproteinemia, Proteinuria	NO	PR	non-MPR	Non-responder	0.55	723	800
Patient06	/	15w	2	Hypertension, Dizziness	NO	PD	MPR	Responder	0.45	637	880
Patient07	/	15w	2	Hypertension, Gingival Bleeding	NO	SD	CPR	Responder	0	648	782
Patient08	/	15w	3	Hypothyroidism, Fatigue	NO	PR	non-MPR	Non-responder	0.6	90	285
Patient09	/	12w	2	Transaminase Elevation	NO	SD	non-MPR	Non-responder	0.65	622	730
Patient10	/	15w	3	Hypertension, Dizziness	NO	PR	MPR	Responder	0.1	571	876
Patient11	/	15w	2	Loss of Appetite, Nausea	NO	/	non-MPR	Non-responder	0.7	566	724
Patient12	Headache accompanied by right-sided eyelid ptosis was observed, considering neurologic adverse event	15w	3	Headache, Upper Eyelid Ptosis	Wound Oozing	PR	MPR	Responder	0.1	601	847
Patient13	/	9w	2	Nausea, Abdominal Discomfort	NO	SD	non-MPR	Non-responder	0.8	/	/
Patient14	/	9w	2	Hypertension	NO	SD	CPR	Responder	0	542	590

Inclusion and Exclusion criteria of cohort construction

Participants must meet all of the following criteria to be eligible for enrollment:

1. Age ≥ 18 years.

2. Histologically, cytologically, or radiologically diagnosed hepatocellular carcinoma.

3. Child–Pugh liver function class A.

4. Eastern Cooperative Oncology Group (ECOG) performance status score of 0–1.

5. Estimated life expectancy ≥ 12 months.

6. Patients who received at least one cycle of PD-1 antibody–based immunotherapy prior to curative-intent surgical resection.

Participants meeting any of the following criteria were excluded from the study:

1. Known diagnosis of intrahepatic cholangiocarcinoma, sarcomatoid hepatocellular carcinoma, mixed hepatocellular–cholangiocarcinoma, or fibrolamellar carcinoma.

2. Current interstitial pneumonitis or interstitial lung disease, or a prior history of interstitial lung disease requiring systemic corticosteroid treatment.

3. Use of immunosuppressive agents or systemic corticosteroids within 2 weeks prior to enrollment for immunosuppressive purposes.

4. Presence of active infection; unexplained fever ≥38.5 °C within 1 week prior to randomization; baseline leukocyte count >15 × 10^9/L; or receipt of therapeutic oral or intravenous antibiotics within 2 weeks prior to randomization (excluding prophylactic intravenous antibiotics administered for ≤48 hours).

5. Congenital or acquired immunodeficiency, including known HIV infection.

6. Clinically significant bleeding events or clear bleeding tendency within 6 months prior to treatment initiation, such as gastrointestinal bleeding, severe esophagogastric varices, hemorrhagic gastric ulcer, or vasculitis. If fecal occult blood test was positive at baseline, require repeat test; persistent positivity mandated gastroscopic evaluation.

7. Known hereditary or acquired bleeding disorders (e.g., coagulation dysfunction) or thrombotic predisposition, high surgical risk, or inability for curative-intent surgery.

8. Major vascular diseases occurring within 6 months prior to randomization (e.g., aortic aneurysm requiring surgical repair or recent peripheral arterial thrombosis) or other conditions directly increasing surgical risk and precluding surgery.

9. Receipt of major surgery (excluding diagnostic procedures) within 4 weeks prior to study enrollment, or anticipated need for non-oncologic major surgery during the study period.

10. Known hypersensitivity or allergy to any investigational drug or its excipients.
11. Participation in another interventional drug clinical trial.
12. Pregnant or breastfeeding women.
13. Any other condition deemed by the investigator to make the patient unsuitable for participation in the study.

Reference:
Llovet JM, Pinyol R, Yarchoan M, Singal AG, Marron TU, Schwartz M, Pikarsky E, Kudo M, Finn RS. Adjuvant and neoadjuvant immunotherapies in hepatocellular carcinoma. Nat Rev Clin Oncol. 2024 Apr;21(4):294-311.
D'Alessio A, Stefanini B, Blanter J, Adegbite B, Crowley F, Yip V, Slater S, Fulgenzi CAM, Celsa C, Manfredi GF, Pai M, Goldin RD, Ward SC, Fiel MI, Shu DH, Su YY, Cortellini A, Baretti M, Anders R, Yarchoan M, Hsu C, Marron TU, Pinato DJ. Pathological response following neoadjuvant immune checkpoint inhibitors in patients with hepatocellular carcinoma: a cross-trial, patient-level analysis. Lancet Oncol. 2024 Nov;25(11):1465-1475.
Zhou J, Sun H, Wang Z, Cong W, Zeng M, et al. China Liver Cancer Guidelines for the Diagnosis and Treatment of Hepatocellular Carcinoma (2024 Edition). Liver Cancer. 2025 Jul 10;14(6):779-835.

2. The authors' findings overlap extensively with previously published neoadjuvant anti–PD-1 translational studies, most notably the Nature Medicine study (PMID: 37322116) and the Molecular Cancer study (PMID: 40205519), which have already characterized the immunotherapy-associated tumor microenvironment, including T cell and macrophage states in HCC. Also, there are 5 similar published neoadjuvant IO trials in HCC that the authors should at least discuss and compare with.
To strengthen the manuscript, the authors should directly compare their clinical readout and translational results with landmark datasets, at minimum discussing the degree of similarity or divergence between their MacroLA cluster and the previously described macrophage populations, including phenotypic markers and proposed functions. Without such cross-comparison, it is difficult to assess what is truly novel in this study relative to prior work.

Author's response: We thank the reviewer for this important comment and fully agree that several recent neoadjuvant anti-PD-1 translational studies in HCC, including the Nature Medicine study (PMID: 37322116) and the Molecular Cancer study (PMID: 40205519), have provided valuable insights into the HCC immunotherapy-associated tumor immune microenvironment (TIME), particularly regarding T cell activation and macrophage states. However, the central biological questions and conclusions of the two studies as well as other published neoadjuvant immunotherapy clinical trials are fundamentally different from our research.
Magen et al. (Nature Medicine) focus on T cell differentiation dynamics and spatial immune niches, demonstrating that intratumoral triads composed of mregDC, CXCL13^{+} CD4 T helper cells and progenitor-exhausted CD8^{+} T cells enable the differentiation of effective CD8^{+} T cells after PD-1 blockade. Their work primarily addresses how antigen presentation and DC–T cell interactions instruct CD8 T cell fate within T cell–rich tumors. Notably, in our study, we also utilized the scRNA-seq data from this publication (GSE206325) in Fig. 2i and found the negative correlation between HCCT_FA and clinical efficacy that were not discussed in their original article.
Moreover, our study addresses a distinct and complementary question: why a subset of patients develops a metabolically and structurally immunosuppressive tumor microenvironment that is refractory to PD-1 therapy, despite immune infiltration. Our central finding is the identification of a lipid-associated macrophage (LAM) population enriched in non-responders, characterized by APOA1 and FABP1 expression, and functionally linked to lipid accumulation, extracellular matrix fibrosis, and impaired antitumor immunity. T cells in NR group were reflected as a downstream imprint of a hostile microenvironment. Importantly, we support this conclusion through an integrated evidence chain including single-cell transcriptomics and paired spatial transcriptomics in silico, flow cytometry, multiplex immunohistochemistry, direct histological validation of fibrosis and steatosis in the newly enrolled neoadjuvant immunotherapy cohort, as well as functional validation in animal models.
Thus, while both studies analyze post-treatment HCC samples, they operate at different mechanistic layers: Magen et al. define T cell instructional niches, whereas our work uncovers a myeloid-driven metabolic and fibrotic program that constrains immunotherapy efficacy. We believe these findings are complementary rather than redundant, and together

highlight the multifaceted nature of immune resistance in HCC.
In the potential overlap with the Molecular Cancer study. We agree that both studies examine the post–neoadjuvant anti–PD-1 tumor immune microenvironment in HCC and include analyses of T cells and macrophages. However, the primary emphasis and mechanistic focus are different.
Zeng, et al. (Molecular Cancer) mainly highlights the functional antagonism and spatial interplay between TAMs and $CD8^+$ T cells, and proposes macrophage-associated signaling axes that constrain $CD8^+$ T cell function in non-responders.
However, Zeng, et al. mostly focused on the interaction between TAM and $CD8^+$ T cells and illustrating the crucial role of TAM in HCC immunotherapy. In Zeng, et al.'s research, TAM was only divided into M1/M2 categories, which may ignore the diversity in macrophage subtypes. In contrast, based on functional sub-clustering, our work focuses on identifying and functionally characterizing a specific LAM subset enriched in non-responders, and links this subset to a metabolic–stromal program involving lipid handling and ECM remodeling. Importantly, beyond transcriptomic associations, we provide orthogonal validation (flow cytometry multiplex immunostaining and animal model) and direct tissue-level evidence supporting the LAM-associated phenotype in the same patient cohort.
Therefore, while both studies address macrophage–T cell interactions in the context of neoadjuvant immunotherapy, the Molecular Cancer work primarily describes TAM–T cell antagonism and checkpoint-related communication, whereas our study reveals a distinct LAM-driven metabolic/fibrotic microenvironment that is associated with impaired T cell function and poor response.
In the comparison between our study and neoadjuvant IO trails, we fully agree that recent neoadjuvant studies, including the Notable-HCC trial (Li, et al.), the Camrelizumab-Apatinib study (Xia, et al.), the TCR dynamic study (Zhang, et al.), the PLENTY trial (Lv, et al.), and the translational analysis (Ho, et al.) have substantially advanced the field by establishing clinical feasibility, demonstrating treatment-induced immune activation, and elucidating T cell trafficking dynamics in the neoadjuvant setting.
Importantly, these studies primarily address whether immunotherapy can be safely and effectively administered, what an immune-responsive tumor microenvironment looks like, or how T cells migrate and expand during treatment. In contrast, our study is specifically designed to interrogate a distinct and previously underexplored biological question: why a substantial subset of tumors fails to respond to immunotherapy despite exposure to PD-1 blockade.
Using single-cell RNA sequencing combined with orthogonal validation approaches, we identify LAM population enriched in non-responders and demonstrate its association with metabolic reprogramming, extracellular matrix remodeling, fibrosis, and T cell dysfunction. Unlike prior clinical trials, which largely rely on bulk immune signatures, spatial profiling, or TCR clonotype analyses, our work provides a cellular and mechanistic dissection of an immunosuppressive tumor immune ecosystem that constrains effective antitumor immunity.
Therefore, rather than reiterating prior observations, our study complements existing neoadjuvant immunotherapy trials by providing a mechanistic explanation for immunotherapy resistance in HCC.
Then, we'd like to compare LAM in our dataset with previously described TAM in the level of phenotype and function.
First, at the level of phenotypic markers, the LAM population identified in our cohort shares key transcriptional features with previously reported LAMs across tumor types (Ma, et al.). Consistent with prior studies, these macrophages are built upon an M2-like macrophage backbone and are characterized by the expression of apolipoprotein (APO) family genes and (FABP) family genes, supporting their classification as lipid-associated macrophages. However, in previously reported LAM, the expression levels of TREM2 and SPP1 are also relatively higher than other macrophages. But LAM in our data showed average SPP1 expression and lower TREM2 expression among macrophages, which highlighted the negative regulation of lipid associated features to immunotherapy.
Moreover, despite these molecular features, the functional role of LAMs in the context of immunotherapy response in hepatocellular carcinoma has not been comprehensively defined. In contrast to colorectal cancer, where $FOLR2^+$ $APOE^+$ macrophage populations have been reported to associate with improved responses to immune checkpoint blockade (Li, et al.), our data demonstrate that LAMs in HCC are negatively associated with immunotherapy efficacy. Specifically, LAM enrichment in non-responders is accompanied by the formation of an immunosuppressive and pro-fibrotic tumor microenvironment, characterized by extracellular matrix remodeling and lipid accumulation.
In addition, we have conducted an integrated analysis according to the reviewer's suggestion in Question 5 by introducing the external HCC immunotherapy scRNA-seq dataset (PMID: 36708811, Liu, et al.) into our 14-patient HCC immunotherapy data. Integrated analyses showed that while SPP1 was expressed in LAMs, it appears not to be the most

prominent features compared to other lipid-associated features; conversely, lipid metabolism related genes were rarely expressed in typical in SPP1⁺ macrophages, where the SPP1 expression is extensive and intense. These findings demonstrate that LAM constitutes a macrophage subtype distinct from SPP1⁺ macrophages, characterized by enhanced lipid metabolic features with comprehensive immunosuppression. The detailed results were shown in Question 5.

In summary, our study differs fundamentally from the Nature Medicine work in that it focuses on a myeloid-driven metabolic and fibrotic program that constrains immunotherapy efficacy, rather than on T cell instructional niches and differentiation trajectories. Compared with the Molecular Cancer study, which emphasizes TAM–T cell antagonistic signaling, our work identifies and functionally validates a distinct lipid-associated macrophage (LAM) population that shapes an immunosuppressive, lipid- and ECM-enriched tumor microenvironment and imprints downstream T cell dysfunction.

In relation to published neoadjuvant HCC immunotherapy trials, our findings provide a mechanistic cellular explanation for primary resistance, complementing clinical and immune-activation observations from these studies by revealing how a LAM-dominated metabolic–stromal ecosystem may limit the efficacy of PD-1 blockade despite immune infiltration. Discovering the mechanism link between LAM and limited neoadjuvant immunotherapy efficacy.

Reference:

Magen A, Hamon P, Fiaschi N, Soong BY, Park MD, et al. Intratumoral dendritic cell-CD4+ T helper cell niches enable CD8+ T cell differentiation following PD-1 blockade in hepatocellular carcinoma. Nat Med. 2023 Jun;29(6):1389-1399.

Zeng F, Zhang Q, Tsui YM, Ma H, Tian L, Husain A, Lu J, Lee JM, Zhang VX, Li PM, Cheung GC, Cheung TT, Ho DW, Ng IO. Multimodal sequencing of neoadjuvant nivolumab treatment in hepatocellular carcinoma reveals cellular and molecular immune landscape for drug response. Mol Cancer. 2025 Apr 9;24(1):110.

Li Z, Liu J, Zhang B, Yue J, Shi X, Cui K, Liu Z, Chang Z, Sun Z, Li M, Yang Y, Ma Z, Li L, Zhang C, Sun P, Zhong J, Zhao L. Neoadjuvant tislelizumab plus stereotactic body radiotherapy and adjuvant tislelizumab in early-stage resectable hepatocellular carcinoma: the Notable-HCC phase 1b trial. Nat Commun. 2024 Apr 16;15(1):3260.

Xia Y, Tang W, Qian X, Li X, Cheng F, et al. Efficacy and safety of camrelizumab plus apatinib during the perioperative period in resectable hepatocellular carcinoma: a single-arm, open label, phase II clinical trial. J Immunother Cancer. 2022 Apr;10(4):e004656.

Zhang Y, Tsui YM, Lee JM, Cheung TT, Ho DW, Ng IO. Spatiotemporal dynamics of T cells in peripheral blood and tumor underlying differential responses to neoadjuvant PD-1 blockade in hepatocellular carcinoma. J Immunother Cancer. 2025 Dec 1;13(12):e013516.

Lv Z, Xiang X, Yong JK, Zhou Y, Wu Y, Li L, Wang Y, Zhang Z, Xia Q, Feng H. Pembrolizumab in combination with LEnvatinib in participants with hepatocellular carcinoma before liver transplant as Neoadjuvant TherapY-PLENTY pilot study. Int J Surg. 2024 Oct 1;110(10):6647-6657.

Ho WJ, Zhu Q, Durham J, Popovic A, Xavier S, et al. Neoadjuvant Cabozantinib and Nivolumab Converts Locally Advanced HCC into Resectable Disease with Enhanced Antitumor Immunity. Nat Cancer. 2021 Sep;2(9):891-903.

Ma RY, Black A, Qian BZ. Macrophage diversity in cancer revisited in the era of single-cell omics. Trends Immunol. 2022 Jul;43(7):546-563.

Li W, Pan L, Hong W, Ginhoux F, Zhang X, Xiao C, Li X. A single-cell pan-cancer analysis to show the variability of tumor-infiltrating myeloid cells in immune checkpoint blockade. Nat Commun. 2024 Jul 21;15(1):6142.

Liu Y, Xun Z, Ma K, Liang S, Li X, Zhou S, Sun L, Liu Y, Du Y, Guo X, Cui T, Zhou H, Wang J, Yin D, Song R, Zhang S, Cai W, Meng F, Guo H, Zhang B, Yang D, Bao R, Hu Q, Wang J, Ye Y, Liu L. Identification of a tumour immune barrier in the HCC microenvironment that determines the efficacy of immunotherapy. J Hepatol. 2023 Apr;78(4):770-782.

3. The methodology for tissue dissociation prior to single-cell RNA-Seq is not described. Dissociation procedures (enzymatic digestion, timing, temperature) can introduce artifacts, bias cell recovery, and alter gene expression signatures, and therefore need to be reported in sufficient detail. In addition, key sequencing quality metrics such as matrix characteristics (number of cells captured, median genes per cell, sequencing depth) and library saturation are not provided. These are essential for evaluating dataset robustness and reproducibility. The authors should include both dissociation details and standard scRNA-seq QC metrics, ideally in the Methods or

Supplementary Information. Furthermore, it is unclear how the authors handled potential artifacts such as cell doublets or ambient RNA contamination ("RNA soup"), which can significantly affect clustering and downstream inferences. Beyond the standard CellRanger pipeline and typical cell filtering (e.g., mitochondrial read percentage, gene/cell thresholds), were additional steps taken (e.g., Scrublet, DoubletFinder, SoupX) to ensure data quality? Explicitly addressing these points would increase confidence in the robustness of the single-cell dataset.

Author's response: We thank the reviewer for this thorough and important comment regarding the technical rigor and transparency of our single-cell RNA-sequencing methodology. We fully agree that detailed reporting of tissue dissociation procedures, sequencing quality metrics, and strategies for mitigating technical artifacts such as doublets and ambient RNA contamination is essential for assessing data robustness, reproducibility, and biological interpretability.
In response to this comment, we have carefully reviewed and expanded the Methods part (line 749 to 799) to provide a more comprehensive description of sample processing, sequencing quality control and data-cleaning procedures, which are also provided in the following text.

Single cell sample collection and suspensions preparation

Fresh tumor samples were obtained during surgical resection and rapidly transported to the research facility on ice. Tissues were transferred into a sterile culture dish containing 10 mL of 1× Dulbecco's phosphate-buffered saline to remove residual storage solution and then minced into 1–3 mm³ fragments. Tissue digestion was performed using type I collagenase dissolved in 10 mL RPMI 1640 medium supplemented with 10% fetal bovine serum. Samples were incubated at 37 °C with gentle agitation (50 r.p.m.) until adequate dissociation was achieved.
The resulting cell suspensions were passed through a 70-μm nylon cell strainer, and red blood cells were removed using 1× Red Blood Cell Lysis Solution. Cells were washed with DPBS containing 2% FBS. Cell viability was assessed using 0.4% Trypan Blue staining on a Countess® II Automated Cell Counter, and only samples with viability >85% were processed further.
Cells meeting the quality criteria were washed, resuspended to an appropriate concentration of 700–1200 cells/μl, and prepared for operation on 10x Genomics Chromium™ system. Specificly, cells were loaded according to the expected number of target cells to construct Gel Bead-in-Emulsion (GEM) droplets for single-cell isolation. Once GEMs were successfully formed, they were collected and subjected to reverse transcription in a PCR machine for tagging. After reverse transcription, GEMs were processed to break the oil emulsion, and single-strand cDNA was purified and enriched using magnetic beads. The cDNA was then amplified in a PCR machine and quality-checked. Quality-checked cDNA was used to construct sequencing libraries through fragmentation, adapter ligation, and sample index PCR. The libraries were subsequently quantified and subjected to quality control. Finally, the constructed libraries were sequenced using the Illumina HiSeq platforms, employing a PE150 sequencing mode to achieve a read depth of 50,000 read pairs per cell or greater.

Raw sequencing data processing and quality control

Raw sequencing data was subjected to quality control analysis using FastQC software. The 10x Chromium single-cell transcriptome was analyzed through 5' end transcript sequencing, alongside sequencing analysis of T cell and B cell receptor gene TCR/BCR sequences. Across samples, a median of 8932.5 cells were captured per library. Libraries were sequenced to a median depth of 71049.5 reads per cell, yielding a median of 1577 genes and a median of 1692.5 unique molecular identifiers (UMIs) per cell, with library saturation ranging from 90% to 95%. From the Fastq Reads, a cell gene expression matrix was generated, with one of the key steps being the identification of valid cells from background signals. Cell Ranger software was employed to perform data quality statistics on the raw Fastq data, align it to the Ensemble reference genome, carry out cell demultiplexing, remove PCR duplicates, and identify cells. Standard quality control filters were applied to remove low-quality cells based on gene counts, UMI counts, and mitochondrial gene percentages. We examined the overall distribution of gene counts in each cell and filtered out cells with gene counts of < 200 or > 6000. To remove damaged cells indicated by the leakage of mitochondrial genes, we evaluated sample-wise percentage of mitochondrial reads sequenced per cell to eliminate potential debris and damaged cells that could affect the overall quality of the data. Cells with over 30% mitochondrial reads were removed from the dataset. To further address technical artifacts, DoubletFinder was used to identify potential doublets and the cells in top 1% pANN score

were removed. For each sample, the optimal principal component neighborhood size (pK) was selected based on the highest bimodality coefficient using the mean–variance–normalized bimodality coefficient (BCMVN) maximization strategy, thereby improving doublet detection accuracy.

4. The spatial transcriptomics data presented in the manuscript are obtained from an external dataset (PMID: 36708811; Mendeley skrx2fz79n). However, the manuscript provides little discussion on whether these data are directly comparable or transferrable to the current study cohort, given potential differences in sample processing, sequencing depth, and clinical context. The limitations of relying on a public dataset for validation, rather than generating in-house spatial data from the same patients, should be explicitly acknowledged. At minimum, the authors should clarify how differences in cohort characteristics and methodology might affect the interpretation and generalizability of their findings.

Author's response: We thank the reviewer for raising this important point regarding the use of external spatial transcriptomics (ST) data. Although the ST dataset used in our initial manuscript was not generated from samples paired with our in-house single-cell cohort, it is important to clarify that this external dataset was also derived from HCC patients who had also received immunotherapy, making it well suited as an independent validation cohort rather than a purely descriptive reference. In this context, the external ST data provide an unbiased means to examine whether the spatial organization and functional associations of lipid-associated macrophages (LAMs) observed in our single-cell analyses can be recapitulated across independent immunotherapy-treated cohorts.

Importantly, in response to the reviewer's valuable suggestion, we have now additionally conducted spatial transcriptome sequencing by using tumor samples paired with our own single-cell cohort (N=6) during this revision. These in-house ST experiments were performed using a higher-resolution platform (DynaSpatial HD) with a spot diameter of 15 µm, approaching near–single-cell resolution. This paired spatial analysis serves two key purposes: first, it provides direct spatial validation of the presence of LAMs within the tumor tissue; and second, it further strengthens the link between LAM enrichment and immunotherapy response, as LAMs were more prominently localized in non-responder samples compared with responders. Our supplementary spatial transcriptome data have been deposited in National Genomics Data Center of China National Center for Bioinformation (CNCB-NGDC) under project number PRJCA039315.

Taken together, we have revised the manuscript accordingly to clarify the combination of external ST data as an independent validation resource and newly generated paired in-house ST data substantially enhances the reliability of our discovery on lipid-associated macrophages (line 434 to 437).

[editorial note: third party material]

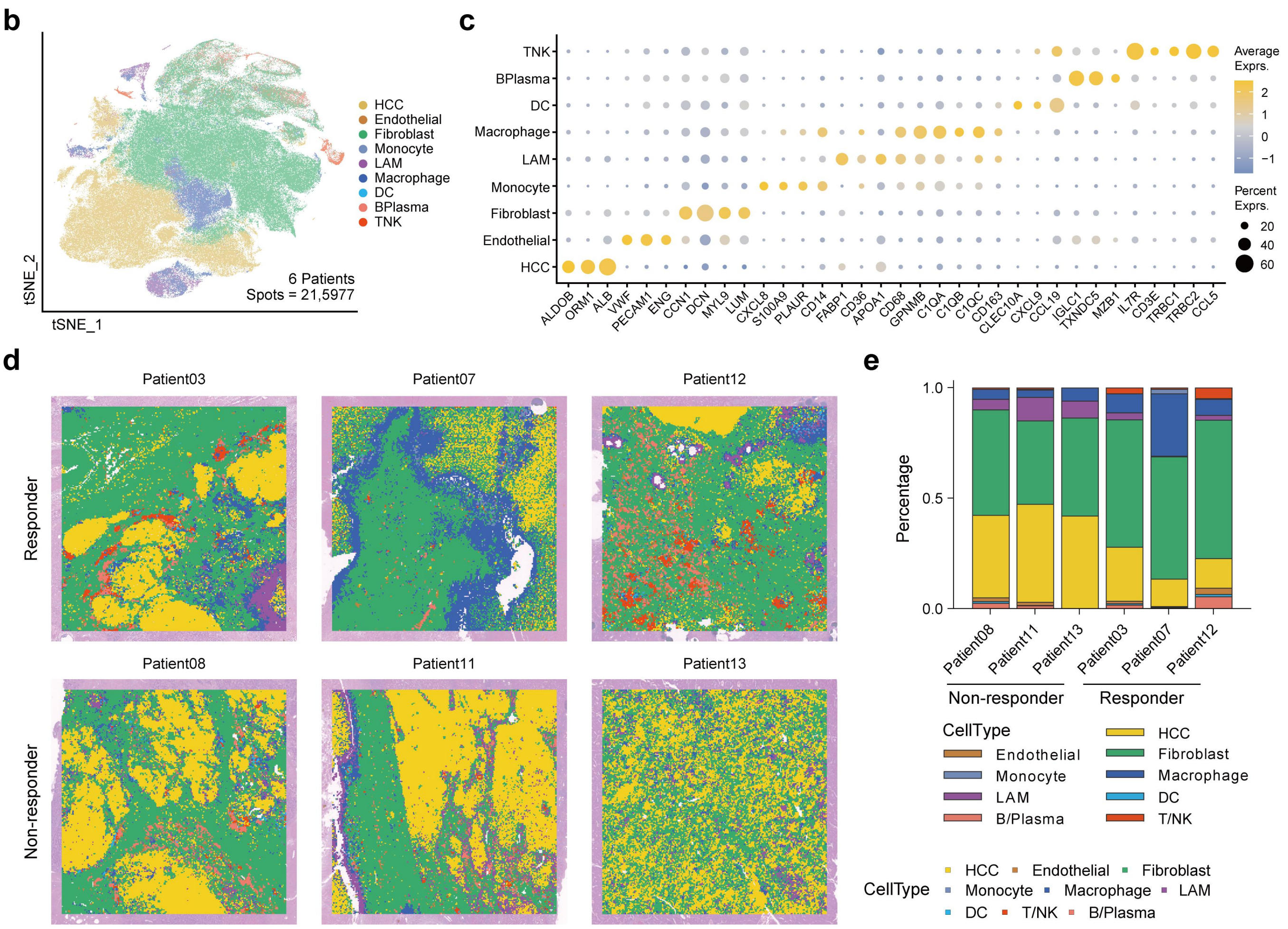

Spatial transcriptomic paired with our scRNA-seq data showing increased LAM infiltration in non-responders. a Scheme of additional study design, 6 spatial transcriptome that paired with our single cell cohort were added to better validate the conclusions in single cell analysis. **b** t-Distributed Stochastic Neighbor Embedding (tSNE) plot of all cells colored by major cell types (HCC, Endothelial cells, Fibroblasts, Monocytes, LAM, Macrophages, Dendritic cells, B and plasma cells and T/NK cells) according to specific markers. **c** Major cell types identified from **(b)** and corresponding gene expression features. **d** Characterization of spatial transcriptomic data by cell-type deconvolution. **e** Bar plot of cell type proportion among 6 patients in spatial transcriptomic sequence.

5. Macrophage annotation in the manuscript is insufficient. For example, PMID: 36708811 already identified five macrophage subtypes in HCC (SPP1⁺, FOLR2⁺, CXCL10⁺, CCL3L1⁺, and proliferating), and demonstrated that SPP1⁺ macrophages form the tumor immune barrier (TIB) with CAFs, thereby restricting immunotherapy efficacy. The authors' MacroLA cluster appears to strongly overlap with this previously described SPP1⁺ population: SPP1 is listed among the top five genes in Table S8, and line 499 introduces SPP1 in the context of ECM remodeling (together with FN1). Furthermore, ligand–receptor analysis (line 379) highlights fatty acid–enriched tumor cells interacting with TREM2 on MacroLA and SPP1–CD44 interactions, both of which are canonical features of SPP1⁺/TREM2⁺ LAM populations. Taken together, the data strongly suggest that the "MacroLA" described here is not a novel population but corresponds to the already reported SPP1⁺ and/or TREM2⁺ macrophages. A direct cross-comparison to the established macrophage subtypes from prior atlases is essential to properly contextualize these findings and to clarify whether the current work adds incremental insight or represents a rediscovery.

Author's response: We thank the reviewer for this detailed and insightful critique and fully agree that rigorous benchmarking against established macrophage atlases is essential. Our analyses indicate that although LAMs share partial transcriptional features overlap with previously reported SPP1^{+}macrophages, they cannot be considered equivalent populations and instead represent a distinct macrophage state or indicated the feature related to upstream causes of immunosuppression with unique functional relevance in the context of HCC immunotherapy. Specifically:

First, the gene lists in original Table.S8 were only ranked by log2FC, which may cause misleading as it cannot fully represent the universality features of a cluster while the ranking is influenced by multiple factors. In that case, we have conducted comprehensive evaluation by bringing log2FC, percent of expression and p value into ranking criteria and revised the gene list of characteristics in Supplementary Table 9, illustrating expression characteristics of each celltype compared to all other subtypes. According to the updated list, we noticed SPP1 is also one of the top DEGs expressed in LAM population, yet with multiple lipid-associated genes highly expressed (Fig. 4b).

Importantly, we have conducted an integrated analysis according to the reviewer's suggestion by introducing the external HCC immunotherapy scRNA-seq dataset (PMID: 36708811, Liu, et al.) into our 14-patient HCC immunotherapy data and processed batch effects in potential through Harmony method. According to the single-cell atlas in the following figures, we are able to compared the spatial distributions of the SPP1^{+} macrophages pre-defined in research of Liu, et al. and the LAMs identified in our study on a UMAP embedding. The results showed very few overlap between these two populations, indicating that SPP1^{+} macrophages and our LAM subset represent distinct macrophage subtypes. Furthermore, featureplots of signature genes revealed that while SPP1 was expressed in LAMs, it appears not to be the most prominent features compared to other lipid-associated features; conversely, these lipid metabolism related genes were rarely expressed in typical in SPP1^{+} macrophages, where the SPP1 expression is extensive and intense. Collectively, these findings demonstrate that LAM constitutes a macrophage subtype distinct from SPP1^{+} macrophages, characterized by enhanced lipid metabolic features with comprehensive immunosuppressive functionality.

Third, within our cohort, SPP1 is also highly expressed in a separate subset ("Macro_Angio"), whereas LAMs are prominently characterized by strong up-regulation of lipid metabolic genes, including FABP1, APOA1, and related pathways (Fig. 4b) which were not significantly expressed in SPP1^{+} TAMs in previous researches. In this context, SPP1 represents only a secondary component of the LAM transcriptional program and likely reflects one of several downstream immunosuppressive patterns rather than the primary determinant of LAM identity or function. Therefore, LAMs should not be directly equated with canonical SPP1^{+} TAMs described in prior atlases based on single-marker overlap alone. Additionally, the Macro_Angio subset with highest expression of SPP1 and TREM2 in our cohort does not show a strong negative association with immunotherapy efficacy. In contrast, LAM enrichment is consistently associated with poor clinical outcomes, indicating that SPP1/TREM2 expression is insufficient to explain treatment resistance. Instead, the combination of lipid metabolic reprogramming, pro-fibrotic activity, and immunosuppressive features uniquely defines LAMs as the macrophage population relevant to impaired antitumor immunity in our study.

Together, these analyses demonstrate that while LAMs share limited overlap with previously described SPP1^{+} macrophages, they represent a biologically and functionally distinct state in HCC immunotherapy, characterized by lipid handling, metabolic–stromal remodeling and immunosuppression rather than by SPP1 expression alone.

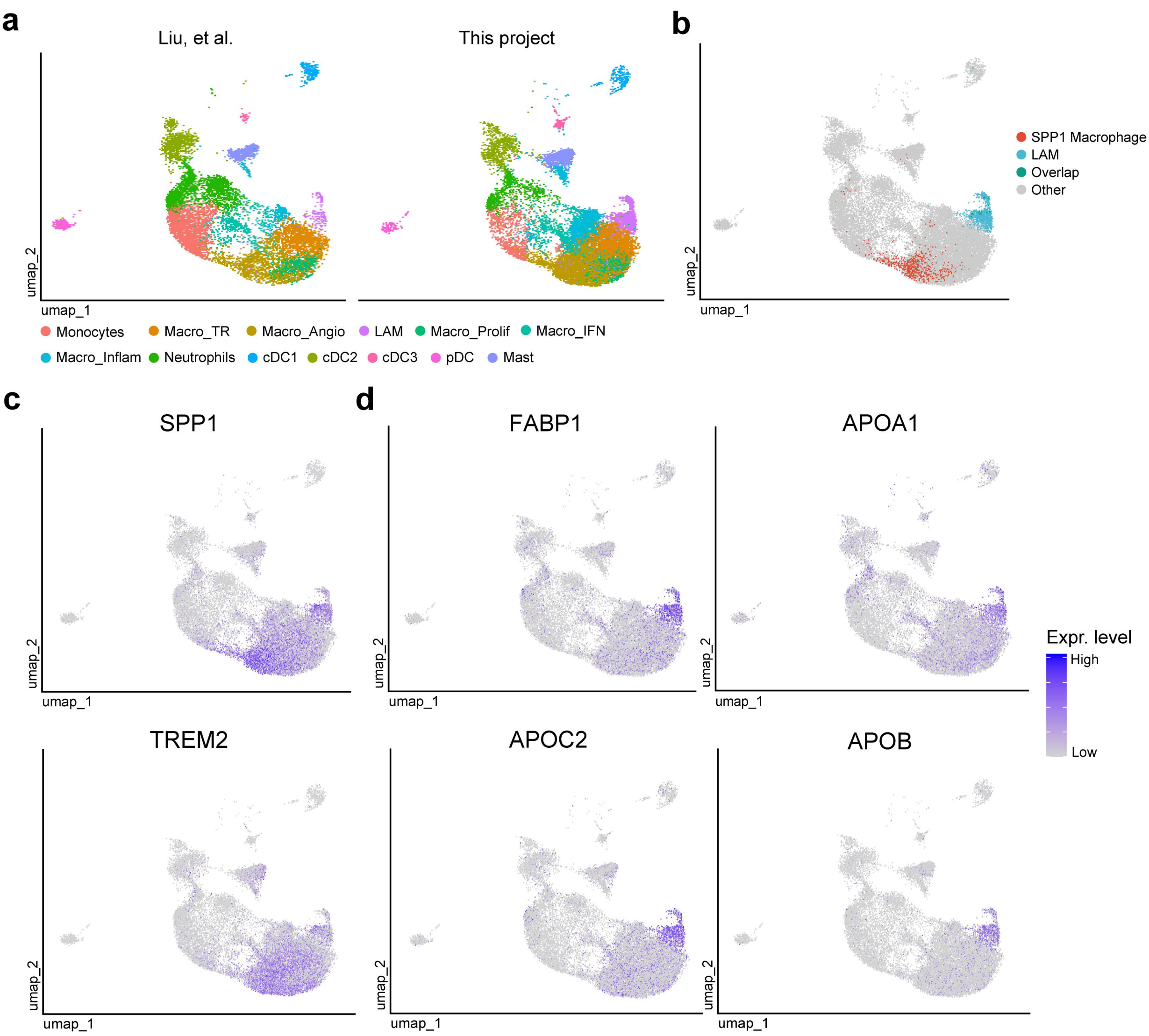

Differences in feature genes between SPP1 Macrophage and LAM. a UMAP plots of myeloid cells from the Liu, et al. data and our data. **b** UMAP plot showing distribution of SPP1 Macrophage pre-defined in Liu, et al. research, LAM defined by characteristics in our research, overlaped SPP1 macrophage and LAM, and other cells. **c** FeaturePlot showing expression levels of SPP1$^+$ Macrophage features (SPP1, TREM2) and LAM features (FABP1, APOA1, APOC2, APOB)

Reference:

Liu Y, Xun Z, Ma K, Liang S, Li X, Zhou S, Sun L, Liu Y, Du Y, Guo X, Cui T, Zhou H, Wang J, Yin D, Song R, Zhang S, Cai W, Meng F, Guo H, Zhang B, Yang D, Bao R, Hu Q, Wang J, Ye Y, Liu L. Identification of a tumour immune barrier in the HCC microenvironment that determines the efficacy of immunotherapy. J Hepatol. 2023 Apr;78(4):770-782.

6. "MacroLA" is not a commonly used term. The established terminology is lipid-associated macrophages (LAMs). The authors should adopt consistent nomenclature and explicitly align their cluster with prior SPP1$^+$ LAM descriptions.

Author's response: We appreciate the reviewer's suggestion regarding nomenclature and agree that consistency with established terminology is important. In the revised manuscript, we have therefore adopted the term "lipid-associated macrophages (LAMs)" throughout, replacing the original "MacroLA" designation to align with commonly used terminology in the field.

At the same time, we have positioned our LAM cluster in relation to previously described SPP1$^+$ LAM populations, clarifying both shared and distinct features in Question 5. In our research, the identified LAMs indeed express canonical tissue-remodeling markers such as SPP1, suggesting the shared downstream immunosuppressive mechanism of LAMs

with prior SPP1⁺ macrophages. However, we think there are certain impede on classifying LAMs into the broader SPP1⁺ macrophage spectrum, for the LAMs from our study exhibited more significant lipid metabolism characteristics such as *FABP1*, *APOA1* and *APOC1*, while these features were absent in typical SPP1⁺ macrophages defined in single-cell atlas of HCC immunotherapy (Liu, et al.).

Moreover, we believe that the contribution of our work is not about the introduction of a specific macrophage category, but rather exploring the function of this kind of LAMs with features mentioned above in HCC immunotherapy, highlighting their additional lipid metabolic, pro-fibrotic and T cell–suppressive properties that are specifically associated with poor therapeutic response, thus providing potential intervention strategies to improve clinical efficacy.

Reference:

Liu Y, Xun Z, Ma K, Liang S, Li X, Zhou S, Sun L, Liu Y, Du Y, Guo X, Cui T, Zhou H, Wang J, Yin D, Song R, Zhang S, Cai W, Meng F, Guo H, Zhang B, Yang D, Bao R, Hu Q, Wang J, Ye Y, Liu L. Identification of a tumour immune barrier in the HCC microenvironment that determines the efficacy of immunotherapy. J Hepatol. 2023 Apr;78(4):770-782.

7. The manuscript inconsistently applies Monocle2 for some pseudotime analyses and Monocle3 for others, without providing an explanation or justification for this choice. This inconsistency is concerning, as different versions of the tool use distinct algorithms and assumptions that may influence trajectory inference. The authors should: Clearly state in the Methods section why Monocle2 was used in some cases while Monocle3 was applied in others. Demonstrate concordance between the pseudotime inferences from Monocle2 and Monocle3 to increase confidence in the robustness of the results. Consider complementing these analyses with RNA velocity, which can provide orthogonal support for directionality of differentiation, particularly in the macrophage and T cell trajectories central to the study's conclusions.

Author's response: We thank the reviewer for raising this important methodological concern and apologize for the lack of clarity in the original manuscript. We would like to first clarify that all pseudotime analyses performed using Monocle2 and Monocle3 were applied to the same cell populations derived from our in-house cohort, and we emphasize this point in our revised manuscript (line 242 to 244, line 267 to 269). Thus, the underlying input data for trajectory inference were identical, and the two tools were not applied to different subsets of cells.

The rationale for using both Monocle2 and Monocle3 was to assess the robustness of trajectory inference across algorithmic implementations rather than to generate independent biological claims. Monocle2 was used for the primary pseudotime analyses because it has been widely adopted in immunology and cancer single cell studies and provides a well-established and more readable DDRTree-based framework for lineage ordering (Trapnell, et al.). Monocle3 was subsequently applied as an independent validation approach using a graph-learning strategy. Importantly, despite differences in algorithmic assumptions, both methods yielded concordant pseudotime structures and led to consistent biological interpretations regarding macrophage and T cell state transitions.

For the T cell analyses, CD4⁺ T cells and CD8⁺ T cells were first extracted from T/NK cells of our dataset and re-embedded and re-clustered within Seurat prior to downstream Monocle3 trajectory analysis. As a result, the UMAP layouts shown for Monocle3-based pseudotime analyses differ from those generated using the full immune compartment, reflecting differences in the analyzed cell subset rather than algorithmic inconsistency. For the macrophage analyses, because monocytes and macrophages constituted the dominant populations within the myeloid compartment, we directly extracted these cells from the Seurat-based myeloid clustering space and applied embeddings again with trajectory inference on this subset in computing frame of Monocle3.

Regarding RNA velocity, although it can provide orthogonal support for directionality by leveraging the relative abundance of spliced and unspliced transcripts (Bergen, et al.); However, its reliability is highly dependent on multiple technical and biological prerequisites that are not uniformly satisfied in our dataset. First, our study is based on post-treatment surgical specimens, where RNA integrity and dissociation-induced stress can vary across samples and cell states, potentially affecting the quantification of unspliced reads and violating key assumptions underlying velocity models. Second, RNA velocity analyses can be sensitive to batch effects and require careful harmonization of preprocessing steps (e.g., spliced/unspliced quantification, normalization, neighborhood graph construction) to avoid

artifactual flows. Given these constraints and our primary goal of establishing robust and reproducible state transitions, we prioritized a conservative strategy in which we validated trajectories using two independent pseudotime frameworks (Monocle2 and Monocle3) applied to the same cell populations, which yielded concordant lineage ordering and consistent biological conclusions. Notably, other single cell studies have demonstrated that consistent results obtained from multiple pseudotime algorithms applied to the same dataset are sufficient to support trajectory inference and biological interpretation, even in the absence of RNA velocity analysis (Balzer, et al.) (Li, et al.).

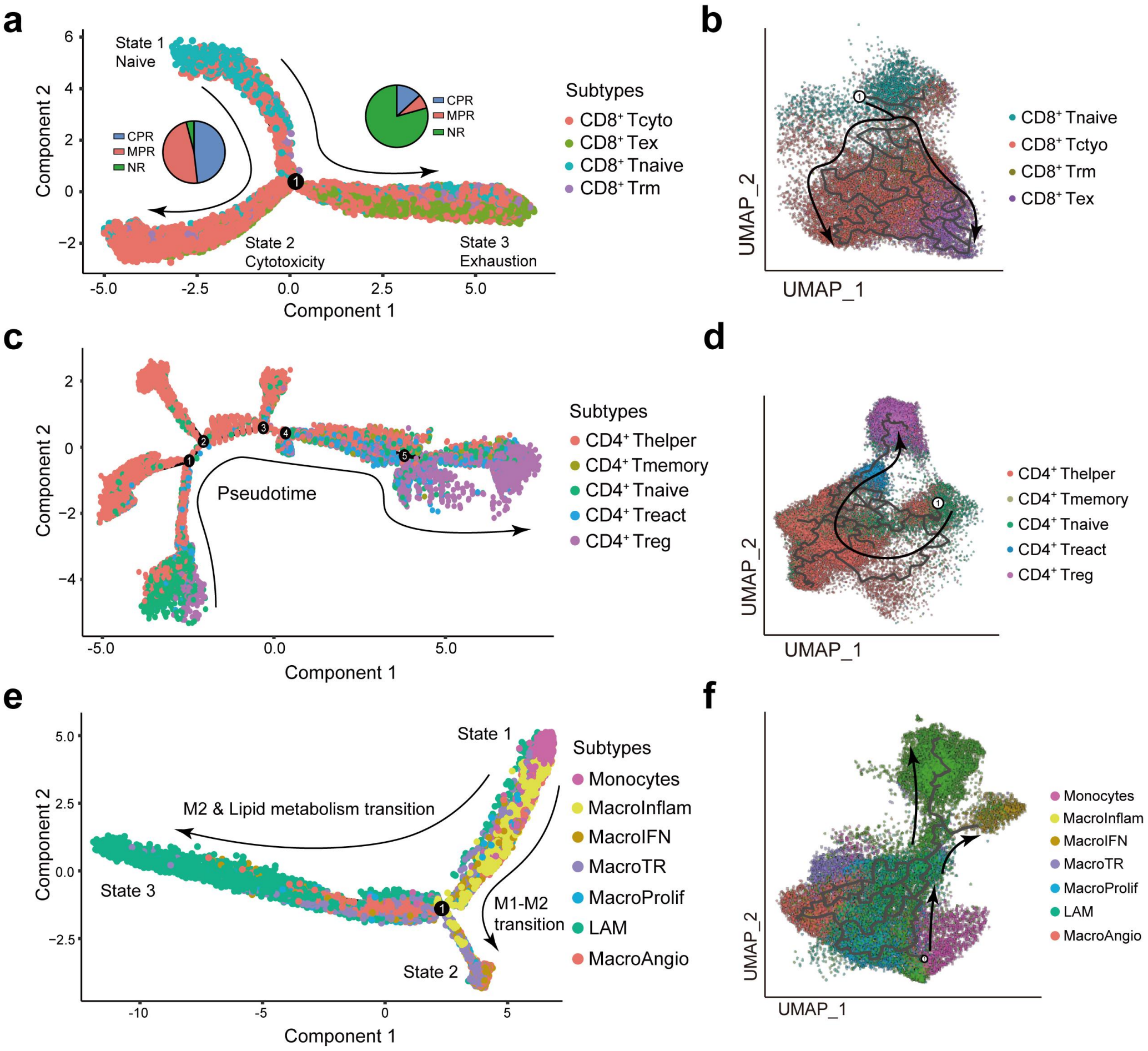

Pseudotime analysis of CD8$^+$ T cells, CD4$^+$ T cells and myeloid cells by Moncle2 and Monocle3. a The developmental trajectory of CD8$^+$ T cells from our HCC immunotherapy cohort inferred by Monocle2, labeled with the CD8$^+$ T subtypes. **b** Monocle3 analysis of CD8$^+$ T cells in complement with **(a)**. **c** The developmental trajectory of CD4$^+$ T cells from our HCC immunotherapy cohort inferred by Monocle2, labeled with the CD4$^+$T subtypes. **d** Monocle3 analysis of CD4$^+$ T cells in complement with **(c)**. **e** The developmental trajectory of monocytes and macrophages from our HCC immunotherapy cohort inferred by Monocle2, labeled with the myeloid subtypes. **f** Monocle3 analysis of monocytes and macrophages in complement with **(e)**.

Reference:

Trapnell C, Cacchiarelli D, Grimsby J, Pokharel P, Li S, Morse M, Lennon NJ, Livak KJ, Mikkelsen TS, Rinn JL. The dynamics and regulators of cell fate decisions are revealed by pseudotemporal ordering of single cells. Nat Biotechnol. 2014 Apr;32(4):381-386.

Bergen V, Lange M, Peidli S, Wolf FA, Theis FJ. Generalizing RNA velocity to transient cell states through dynamical modeling. Nat Biotechnol. 2020 Dec;38(12):1408-1414.

Balzer, M.S., Doke, T., Yang, YW. et al. Single-cell analysis highlights differences in druggable pathways underlying adaptive or fibrotic kidney regeneration. Nat Commun 13, 4018 (2022).

Li Y, Ma H, Wu Y, Ma Y, Yang J, Li Y, Yue D, Zhang R, Kong J, Lindsey K, Zhang X, Min L. Single-Cell Transcriptome Atlas and Regulatory Dynamics in Developing Cotton Anthers. Adv Sci (Weinh). 2024 Jan;11(3):e2304017.

8. The study employs pySCENIC for transcription factor (TF) activity inference and CellChat for cell–cell communication analysis. While both are widely used and appropriate for exploratory scRNA-seq analyses, the

current manuscript at times presents these outputs as though they provide direct mechanistic evidence. These tools are largely predictive and hypothesis-generating, rather than definitive. To strengthen their claims, the authors should reframe these results as exploratory insights rather than conclusive mechanisms, consider supplementing their analysis with NicheNet, which is designed to integrate ligand–receptor interactions with downstream gene expression changes in target cells, thereby providing a stronger link between intercellular signaling and observed transcriptional states, and where possible, cross-validate predictions with orthogonal datasets (e.g., spatial transcriptomics, mIHC, or functional assays).

Author's response: We thank the reviewer for this thoughtful and important comment. We fully agree that pySCENIC and CellChat are primarily exploratory, hypothesis-generating computational frameworks rather than tools that provide direct mechanistic proof. In the revised manuscript, we have carefully revised the wording throughout to ensure that the results derived from these analyses are presented as predictive insights into regulatory programs and intercellular communication, rather than as definitive mechanistic conclusions.

To further strengthen the biological relevance of our findings, and in line with the reviewer's suggestion, we additionally performed NicheNet analysis and spatial transcriptomics analysis to validate findings in single cell analysis. Using NicheNet, we compared efficacy-relevant sender–receiver interactions between patients from responder and non-responder group. This analysis revealed that, relative to non-responders, responders were characterized by ligand–target networks associated with enhanced cytotoxic and immune effector functions, whereas non-responders showed stronger activation of programs related to lipid metabolism, immunosuppression, and extracellular matrix remodeling. Importantly, these NicheNet-derived signaling–target relationships were highly consistent with the interaction patterns inferred by CellChat, providing independent cross-validation of the communication trends observed. Moreover, additional spatial transcriptome sequencing using tumor samples paired with our own single-cell cohort (N=6) was conducted as orthogonal datasets to further provide spatial validation on presence of LAMs within the tumor tissue and the link between LAM enrichment and immunotherapy response. (The detailed results were shown in Question 4)

In addition, to better clarify the key pathway and functions of LAM, we included lycopene and pan-PPAR agonist chiglitazar intervention in a murine HCC model, alongside high-fat diet (HFD) and regular diet controls. These interventions were specifically selected to modulate lipid metabolic pathways that emerged from our single-cell analyses, thereby providing pathway-relevant functional validation. (The detailed results were shown in Question 9)

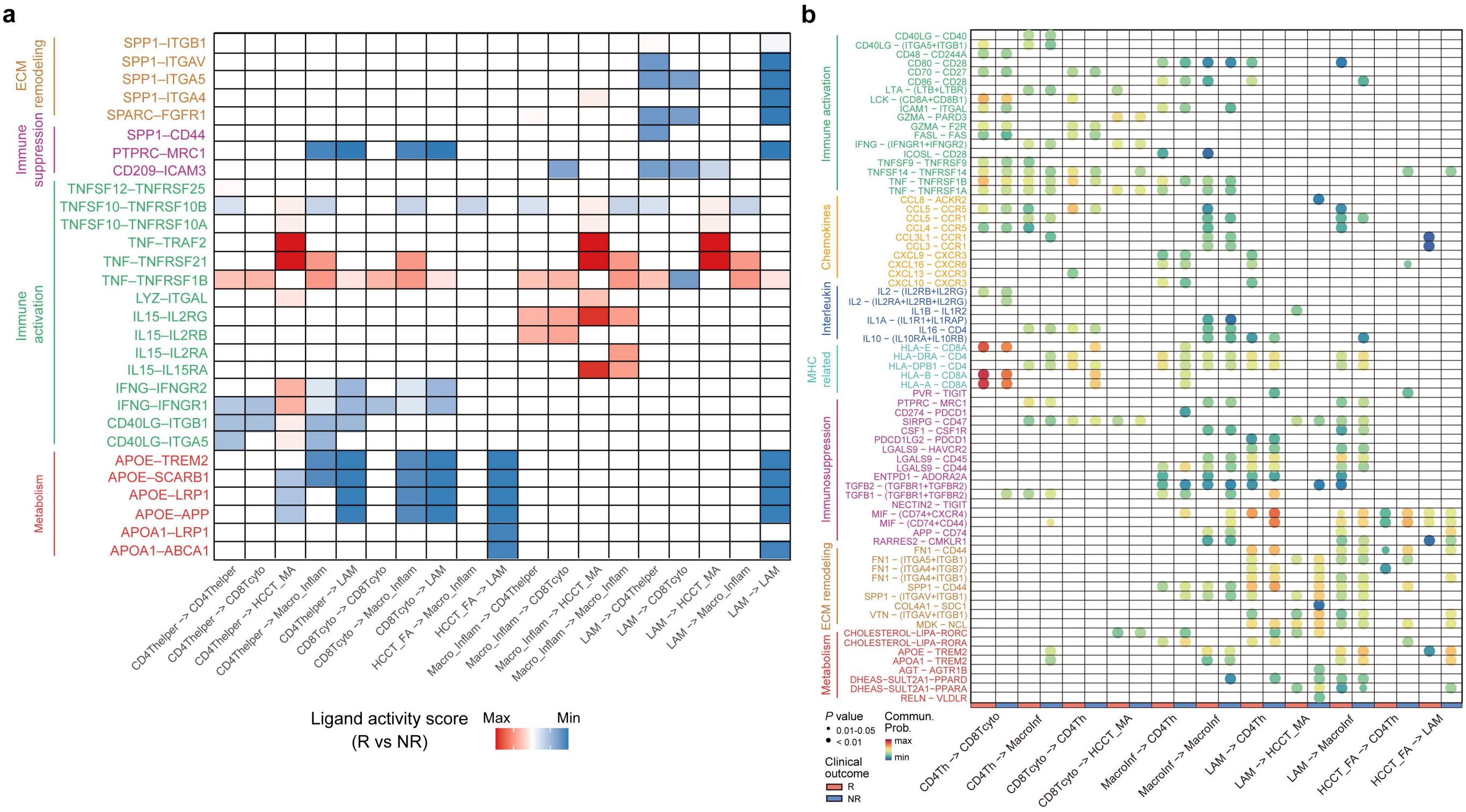

Cross-validation of CellChat results through additional NicheNet analysis. a Heatmap illustrated the comparison between responder and non-responder groups of ligand-receptor interaction landscape among critical TIME components, including CD4$^+$ Th, CD8$^+$ Tcyto, MacroInflam, LAM and HCC_FA. **b** Bubble plot illustrated the ligand-receptor

interaction landscape among critical TIME components, including $CD4^+$ Th, $CD8^+$ Tcyto, MacroInflam, LAMand HCC_FA of responder and non-responder groups.

9. The functional validation using patient-derived organoid–T cell co-culture assays is insufficiently described and raises several interpretive concerns:
- It is not clear how many independent organoid lines and patients' T cells were tested, what the take rate of organoid was, or whether these were derived from the same patients enrolled in the neoadjuvant PD-1 trial versus unrelated HCC patients with similar demographics. Patient demographics are not provided. This lack of detail prevents assessment of representativeness and clinical relevance.

Author's response: We thank the reviewer for this comment and apologize for the incomplete description of patient-derived hepatocellular carcinoma organoids (HCO) experiments. In our study, we successively constructed six patient-derived HCO models, including four HCOs in Initial submission and supplementary two during revision. Meanwhile, the T cells used in our relevant experiment were homologous to HCOs and derived from PBMCs of patients before surgery. Importantly, we here clarify the HCC patients that enrolled for HCO experiment have not received immunotherapy, for their pre-treatment may interference the condition of both HCO models and PBMC derived T cells, and these information have been added in manuscript (line 467 to 477). Collectively, all detailed clinical information from the six patient related to HCO experiments have been supplemented in Supplementary Table 2, including their demographics and disease characteristics.
In terms of methodology, we adopted the "Dual grinding and filtering" approach which have been applied and published for for pan-cancer tissues (Liu, et al.), (Wu, et al.). The key point of this method is to generate uniformed size of tumor organoids rapidly (range from 40-100 μm), without long-term enzymatic dissociation. By using this method for HCO construction, the success rate of is approximately 60% in both this research and the rest of our exploration on HCC. The detailed description of HCO construction have been deposited in the part of Methods of our manuscript (line 943 to 969) and in the following text.

Generation and culture of hepatocellular carcinoma organoids

The liver cancer tissues, which had been stored in a preservation solution (DMEM/F12 containing 1% penicillin and streptomycin), were transported to the laboratory, and processed within 24 hours. Initially, the liver cancer tissues were documented, weighed, and measured for volume. Then, the tissues were sectioned into multiple 1-5 mm3 pieces without bias. The pieces were minced into small fragments using surgical scissors, followed by resuspension in preservation solution (10 mL). The suspension was filtered through a strainer (Falcon, 100 μm) with gentle grinding and pushing of tissue masses through the strainer using a syringe plunger (5 mL). After rinsing, any residual impurities on the membrane were discarded along with the strainer. Subsequently, the filtrate containing cell clusters and single cells was strained through a strainer (40 μm) to collect the cell clusters with sizes ranging from 40 to 100 μm. The filter membrane was separated from strainer, placed in liver cancer organoid medium (HCOM, 2 mL), and thoroughly washed with a pipette to release cell clusters into the media. The membrane was then discarded, and samples were cultured in suspension overnight. The success rate of hepatocellular carcinoma organoids construction is approximately 60% and the clinical data of corresponding patients was reported in Supplementary Table 2.

For culturing pHCOs in a multi-well plate, the pHCOs in suspension were centrifuged at 500 × g for 5 min at 4 °C and then resuspended in cold growth factor-reduced Matrigel (BD Biosciences). Subsequently, drops (60 μL) of the Matrigel cell cluster suspension were plated onto ultra-low attachment 96- or 48-well plates with a flat bottom (Corning) and allowed to solidify at 37 °C for 20 min. The seeding density was adjusted to about 500 organoids per well. Once Matrigel had solidified, HCOM (100 μL) was added to each well, and the plate was transferred to a cell culture incubator (37 °C with 5% CO2). The HCOM medium required to be refreshed at least twice a week.

Reference:
Liu C, Li K, Sui X, Zhao T, Zhang T, Chen Z, Wu H, Li C, Li H, Yang F, Liu Z, Lu YY, Wang J, Chen X, Liu P.
Patient-Derived Tumor Organoids Combined with Function-Associated ScRNA-Seq for Dissecting the Local Immune

Response of Lung Cancer. Adv Sci (Weinh). 2024 Aug;11(31):e2400185.
Wu Y, Li K, Li Y, Sun T, Liu C, Dong C, Zhao T, Tang D, Chen X, Chen X, Liu P. Grouped-seq for integrated phenotypic and transcriptomic screening of patient-derived tumor organoids. Nucleic Acids Res. 2022 Mar 21;50(5):e28.

- It is not specified whether the T cells used in co-culture were autologous to the organoids or derived from unrelated donors. Without this clarification, the observed organoid killing could be explained by allo-reactivity rather than bona fide tumor antigen–specific T cell responses. The absence of information on HLA matching further limits the interpretability of the findings. In addition, the frequency of truly tumor antigen–specific T cells within bulk PBMC is typically very low; unless the relevant antigens were identified and the corresponding T cell populations enriched, it is difficult to conclude that the observed cytotoxicity reflects genuine antigen-specific recognition. At minimum, these caveats should be explicitly acknowledged and discussed.

Author's response: We thank the reviewer for raising this important point and apologize for the lack of clarity in the original description. We fully agree that allo-reactivity is not actual tumor killing effect from T cells, and as we described in the last question, we adopted PBMC derived T cells which homologous to HCOs of patients and emphasized this issue in revised version (line 471 to 472). As the reviewer kindly reminded, we understand the frequency of truly tumor-reactive T cells could be rather limited in PBMCs. The motivation for us to conduct co-culture of PBMC derived T cells with homologous HCOs is the previous "clonal expansion" and "clonal revival" theory (Liu, et al.), which demonstrated peripheral $CD8^+$ T cells can be traced to intratumoral T cells by specific TCR clonotype with 5' scRNA-seq, thus providing the tumor-reactive potential from PBMC derived T cells. Additionally, a similar experimental strategy has been adopted in prior studies (Li, et al.), in which autologous PBMCs were co-cultured with patient-derived tumor organoids to assess antitumor T cell activity and predict clinical outcomes, also based on the premise that circulating PBMCs can harbor T cell populations with tumor-reactive potential and exert anti-tumor effects during co-culture.

Therefore, the experimental design was mainly based on the existing framework above and intended to evaluate the immunomodulatory effects inferred from our single-cell analyses, including the impact of LAMs on T cell immune functions and cytotoxic activity, rather than formally demonstrate the in-depth anti-tumor immune processes driven by T cells, such as antigen-specific identification or analysis of T cell populations. However, we fully acknowledge that these issues are certain caveats for this part of experiments, thus we have supplemented this view in the discussion of our manuscript (line 676 to 678) and thank the reviewer again for raising this critical point.

Reference:
Liu B, Hu X, Feng K, Gao R, Xue Z, Zhang S, Zhang Y, Corse E, Hu Y, Han W, Zhang Z. Temporal single-cell tracing reveals clonal revival and expansion of precursor exhausted T cells during anti-PD-1 therapy in lung cancer. Nat Cancer. 2022 Jan;3(1):108-121.
Li K, Liu C, Sui X, Li C, Zhang T, Zhao T, Zhang D, Wu H, Liu Y, Wang S, Yang Y, Lin B, Wang W, Yang F, Chen X, Liu P. An organoid co-culture model for probing systemic anti-tumor immunity in lung cancer. Cell Stem Cell. 2025 Aug 7;32(8):1218-1234.e7.

- The only T cell function reported is IFN-γ secretion. No assessment of T cell proliferation (e.g., CFSE dilution, Ki-67), expression of exhaustion markers (PD-1, TIM-3, LAG-3), Treg abundance (FOXP3), or effector molecules (granzyme B, perforin, TNF-α) is presented. Without these metrics, it is unclear whether the co-cultured T cells mirror the dysfunctional states identified in the scRNA-seq analysis.

Author's response: We thank the reviewer for this important comment and agree that a broader assessment of T cell functional states would strengthen the interpretation of our findings. In the patient-derived HCOs–T cells co-culture system, IFN-γ secretion was used as the primary functional readout. We acknowledge that this ex vivo system is inherently limited by cell numbers and experimental scale, which restricts the feasibility of performing comprehensive multi-parametric flow cytometric analyses of T cell proliferation, exhaustion markers, and regulatory subsets.
To address this limitation and to more comprehensively evaluate T cell functional states in the context of LAM-mediated

immunosuppression, we therefore established an independent and well-controlled murine HCC model. In this model, HCC-bearing mice were stratified into groups receiving a high-fat diet (HFD) or a regular diet (RD) to induce differential accumulation of lipid-associated macrophages. In parallel, two metabolic intervention arms, HFD supplemented with lycopene or with the PPAR agonist chiglitazar were included to modulate lipid metabolism and macrophage states. All groups were subsequently evaluated for tumor immune phenotypes under comparable experimental conditions.

Using this model, we performed extensive multi-parametric flow cytometric analyses of tumor-infiltrating lymphocytes. Tumors from HFD-fed mice exhibited the highest abundance of LAMs and the most severe T cell dysfunction, characterized by reduced effector cytokine production (TNF and IFN-γ), impaired proliferative capacity (Ki-67), increased expression of exhaustion markers (TIGIT and PD-1), and an elevated proportion of regulatory T cells (Tregs). In contrast, mice maintained on a regular diet displayed preserved T cell effector function and the lowest degree of immune suppression. Notably, both lycopene and chiglitazar partially alleviated the HFD-induced immune dysfunction, resulting in intermediate LAM abundance and T cell functional states between the HFD and RD groups.

Together, these murine data provide a comprehensive functional *in vivo* validation that complements the organoid co-culture *in vitro* assays, demonstrating that LAM enrichment is causally linked to broad T cell dysfunction across multiple functional dimensions.

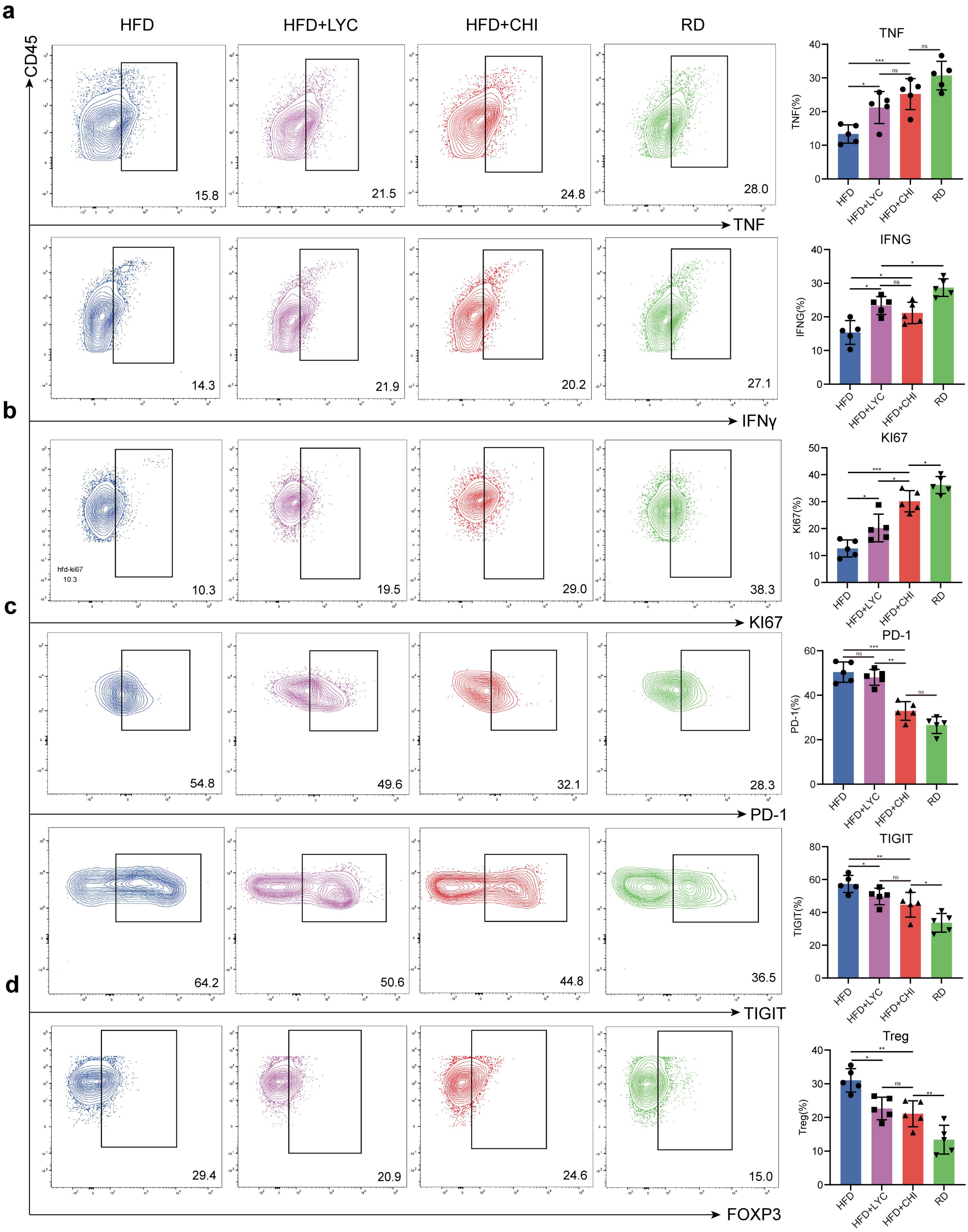

High-fat diet–induced lipid-associated macrophages impair T cell function in a murine HCC model. Tumor tissues were analyzed by flow cytometry for intratumoral T cell function (Gated on CD45+ CD3+). T cell function were analyzed from the aspects of **a** effector cytokine production (TNF and IFN-γ), **b** proliferative capacity (Ki-67), **c** exhaustion (PD-1 and TIGIT), as well as **d** quantification of Tregs by FOXP3 (Gated on CD45+ CD3+ CD4+).

- In the Methods, the authors state that "PMA induces M2 macrophages." This is not accurate: PMA is generally used to differentiate monocytes into macrophages (M0 state), but does not drive M2 polarization. Canonical M2 polarization typically requires exposure to IL-4 and/or IL-13. The authors should clarify the actual polarization conditions used, and provide supporting evidence that M2 identity was achieved, for example by presenting marker expression data (e.g., CD206, MRC1, ARG1). Without such clarification, the functional interpretations of their macrophage co-culture assays remain uncertain.

Author's response: We thank the reviewer for pointing out this inaccuracy and agree that our original wording was imprecise. We clarify that PMA was used solely to differentiate monocytes into macrophages (M0 state), rather than to induce M2 polarization. We apologize for the misleading statement in the Methods and have revised the text accordingly (line 448 to 453, line 915 to 923).

In our experiments, M2 polarization was achieved by subsequent stimulation with canonical cytokines (IL-4 20 ng/ml and IL-13 20 ng/ml) following PMA-induced macrophage differentiation. We have now explicitly described the two-step protocol in the revised Methods section to avoid confusion. To support successful M2 polarization, we additionally provide marker expression data, including increased expression of CD206 gated on $CD68^+$ $C1Q^+$ $APOA1^+$ $FABP1^+$, which is well-established indicator of M2-like macrophage identity in our THP-1 induced LAM.

Together, these clarifications and additions ensure methodological accuracy and complemented the characterization of LAM features in our manuscript.

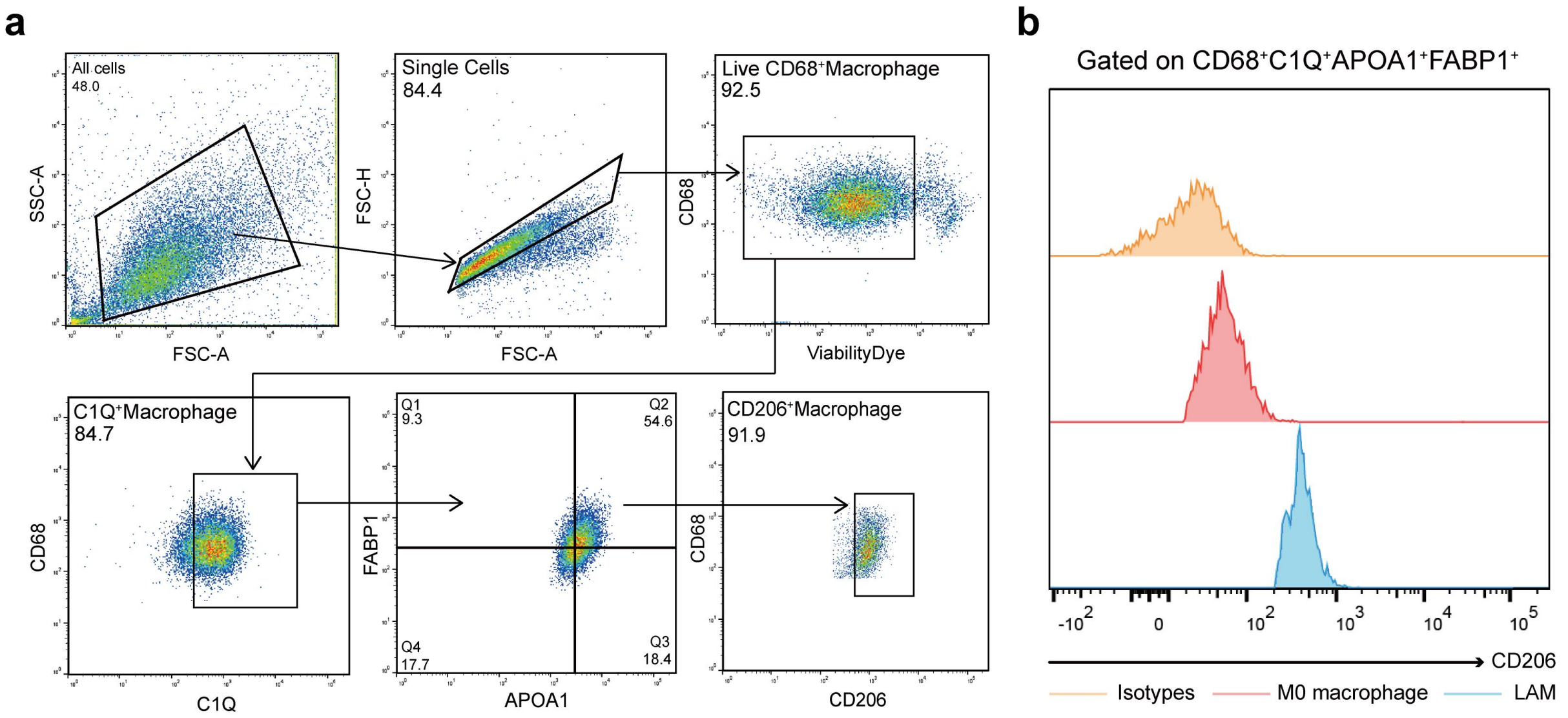

Validation of M2 characteristic of THP-1 induced LAM. a Gating strategy of THP-1 induced LAM. **b** Flow cytometry analysis of CD206 expression shows M2 characteristic of LAMs.

Cell culture of THP-1 and lipid-associated macrophage staining

THP-1 were cultured in RPMI medium with 10 % foetal bovine serum, 1 % penicillin and streptomycin at 37 °C with 5 % CO2. Cells were seeded in a 24-well plate at a density of 250,000 cells per well. At 24h after PMA(RPMI containing 100ng/ml PMA) inducement, cells were washed with PBS and induced by IL-4 and IL-13 (RPMI containing 20ng/ml IL-4 and 20 ng/ml IL-13) for 72h. Then, cells were washed with PBS and incubated in the presence of 0.5 ml of fatty acid medium (RPMI containing 250µM oleic acid) and 0.5ml Lycopene-fatty acid medium(RPMI containing 250µM oleic acid and 50µM lycopene) for 24h.

Macrophage were incubated in 24-well plate at a density of 250,000 cells per well. Cells were washed wih PBS and fixed with 4% paraformaldehyde and then incubated at room temperature in the dark for 15 minutes with 250µl BODIPY 493/503 staining solution per well. Fluorescence microscope with excitation at approximately 488 nm was used to observe green fluorescence.

- Only lycopene was tested, which is a nonspecific intervention. No targeted perturbations of the key pathways highlighted by the single-cell analyses (e.g., PPAR family signaling, SPP1, TREM2) were performed, limiting mechanistic insight.

Author's response: We thank the reviewer for pointing out this limitation, we therefore designed and conducted experiments. To better clarify the key pathways underlying LAM-mediated immunosuppression, in addition to dietary lycopene (LYC) supplementation, we included a pan-PPAR agonist, chiglitazar (CHI), as an intervention in a murine HCC model, alongside high-fat diet (HFD) and regular diet controls. Mice in the HFD groups were maintained on a high-fat diet for 12 weeks prior to tumor implantation to establish a lipid-enriched metabolic state. Each experimental

group consisted of five mice. These interventions were specifically selected to modulate lipid metabolic pathways identified in our single-cell analyses, thereby providing pathway-relevant functional validation of LAM-associated mechanisms. The corresponding results were illustrated in Fig. 7 of our revised manuscript and placed below.

In this setting, we observed that HFD tumors exhibited the highest abundance of lipid-associated macrophages (LAMs) and the most severe T cell dysfunction, whereas both lycopene and chiglitazar treatment partially reversed these phenotypes, resulting in intermediate LAM levels and improved T cell effector function compared with the HFD group. Based on the data that LAM from non-responders exhibited attenuated PPAR signaling compared with responders in scRNA-seq and the central role of PPAR pathways in regulating fatty acid oxidation and lipid homeostasis, we hypothesized that impaired PPAR activity may constrain lipid handling capacity in LAM, thereby sustaining a lipid-rich and immunosuppressive phenotype. Western Blot (WB) assay and quantitative PCR (qPCR) assay showing the expression level of PPAR pathway, suggested that responder-associated LAM maintain a higher lipid metabolic flux, enabling efficient lipid clearance and limiting lipid accumulation, whereas non-responder associated LAM remain metabolically constrained, reinforcing immunosuppressive and pro-tumorigenic functions. Together, by combining lipid-enriched and lipid-modulating interventions with multi-parameter immune profiling, our study provides functional evidence linking lipid metabolic programs to LAM accumulation and T cell dysfunction.

We agree that direct genetic or pharmacologic perturbation of individual molecules such as SPP1 or TREM2 would provide additional mechanistic granularity. However, our current study was designed to integrate human single-cell profiling with *in vivo* metabolic modulation, rather than to perform target-specific perturbation experiments. We have therefore revised the Discussion to explicitly acknowledge this limitation and to position targeted manipulation of SPP1/TREM2 signaling as an important direction for future investigation (line 679 to 682).

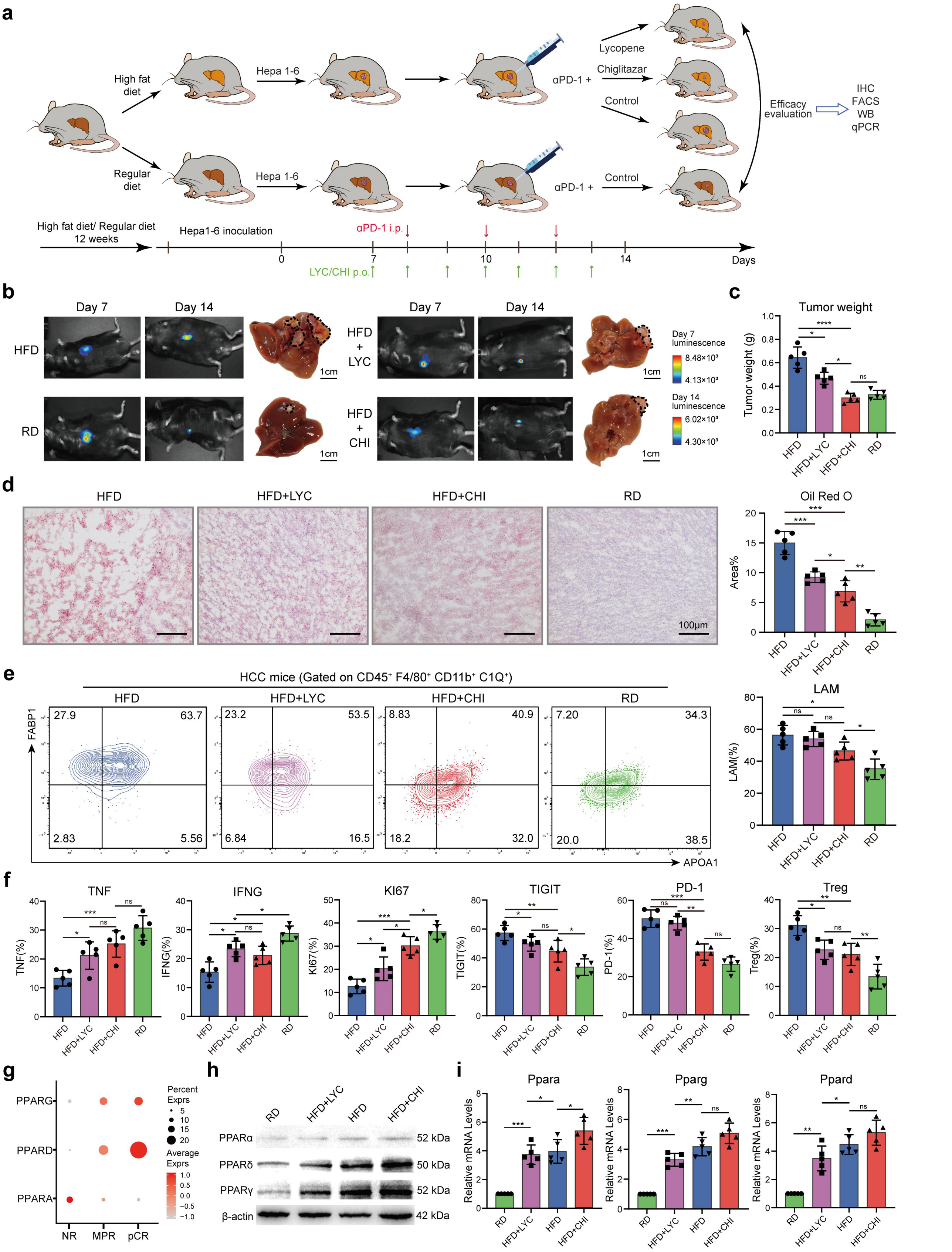

High-fat diet facilitates LAM-mediated immunosuppression in HCC and further regulated by lycopene and chiglitazar. High-fat diet facilitates LAM-mediated immunosuppression in HCC and further regulated by lycopene and chiglitazar. **a** The HCC mice received αPD-1 immunotherapy were randomly assigned to four experimental groups: mice with regular diet (RD), mice with high fat diet (HFD), mice with HFD and lycopene (LYC), mice with HFD and chiglitazar (CHI) (N=5). Subsequently, immunohistochemistry, flow cytometry, WB and qPCR were conducted to explore the features and mechanisms of LAM immunosuppressive function. **b** Representative in vivo bioluminescence images and tumor image of the growth of mice HCC. **c** Tumor weight of HCC mice (N=5). **d** Representative Oil Red O staining of tumor regions showing lipid accumulation degree of each group (N=5). **e** Tumor infiltrating immune cells were isolated from mouse HCC. The percentage of lipid-associated macrophage (LAM) was analyzed by flow cytometry

(N=5). **f** Flow cytometric analysis of intratumoral T cell function, including effector cytokine production (TNF and IFN-γ), proliferative capacity (Ki-67) and expression of exhaustion markers (PD-1 and TIGIT) and proportion of Treg cells (N=5). **g** Bubble plot showing the expression level of PPAR-related genes in LAMs from our HCC immunotherapy scRNA-seq cohort. **h** The protein expression levels of PPARα, PPARδ and PPARγ in mouse HCC tissue were measured by western blotting. **i** The relative expression levels of Ppara, Ppard and Pparg genes were measured by qPCR in mouse HCC tissue (N=5). (Unpaired student's t tests, *P < 0.05, **P < 0.01, ***P < 0.001, ****P < 0.0001, ns: not statistically significant)

Minor comments

1. The authors should use the widely recognized abbreviation "LAM" (lipid-associated macrophages) rather than introducing "MacroLA," unless a strong justification is provided.

Author's response: We agree with the reviewer and have replaced "MacroLA" with the widely recognized term "LAM" (lipid-associated macrophages) throughout the manuscript to ensure consistency with established literature.

2. Typographical errors: Several spelling errors should be corrected, including "demostrated" (lines 115, 431, 1095, 1100) and "Macropahge" (line 765).

Author's response: We thank the reviewer for kindly pointing out these errors. Spelling mistakes including "demostrated" and "Macropahge" have been corrected.

3. Provide full accession identifiers for all datasets analyzed (e.g., GSE IDs, Mendeley DOIs, and related citations) clearly in the Methods section for transparency and reproducibility.

Author's response: We agree that clear reporting of data sources for both our and external datasets is essential. We have now provided complete accession identifiers for all datasets analyzed, including GEO accession numbers, Mendeley Data identifiers, and relevant citations in the Methods section (line 897 to 903). Additionally, all the basic information of patients from our cohort and cohorts of public data have been integrated and recorded in Supplementary Table 2.

Public scRNA-seq and spatial transcriptome datasets

Publicly available single-cell RNA-seq (scRNA-seq) and spatial transcriptomics (ST) datasets were used for reference and validation analyses. ST data were obtained from Mendeley Data (skrx2fz79n). Treatment-naïve HCC scRNA-seq datasets were retrieved from GEO (GSE149614, GSE151530, GSE156625, GSE189903, and GSE202642). scRNA-seq datasets from anti–PD-1–treated HCC patients were obtained from GSE206325 and Mendeley Data (skrx2fz79n).

4. It is not clear whether the in-house single-cell dataset generated in this study will be deposited in a public repository (e.g., GEO, SRA, EGA). Public accessibility is essential for transparency, reproducibility, and re-use by the community, and should be clearly stated.

Author's response: We agree with the reviewer on the importance of public data availability, and have refined the data availability statement in our revised manuscript (line 1046 to 1051). The raw sequencing data has been deposited in National Genomics Data Center of China National Center for Bioinformation (CNCB-NGDC) under project number PRJCA039315. The data deposited and made public for non-commercial purposes, and are compliant with the regulations of the Ministry of Science and Technology of China. Currently, these data are able to be accessed through specific review links ahead of publication, including our scRNA-seq data and spatial transcriptome data, respectively (https://ngdc.cncb.ac.cn/gsa-human/s/qhFw3RD4, https://ngdc.cncb.ac.cn/gsa-human/s/oE9V0rq3). We believe that this would contribute to the transparency, reproducibility, and re-use by the community of our research.

Reviewers' comments

Reviewer #1:

I appreciate the authors' efforts to address the previous comments; however, I still have a number of concerns. In particular, several issues arise from the newly added data and information. These comments are outlined in detail below:

1. Main text: In the text of results is now written: "To more precisely identify the cell types and corresponding subtypes of our single-cell dataset, we integrated 5 public scRNA-seq datasets, consist of 60 untreated HCC patients, thus resulting in a high-throughput single-cell atlas of 74 HCC patients (Supplementary Table 2)." This is not in line with the information in Supplementary Table 2. The number of patients does not match, etc. The explanation of which patients were included is missing, etc. The legend of the table is missing.

Author's response: We thank the reviewer for carefully noting this point and apologize for the lack of clarity in the previous version of our manuscript. The discrepancy arises from the structure of GSE189903 dataset, in which multiple GSM accession numbers correspond to multi-region sampling from the same individual patient. In our analysis, patient numbers were calculated at the individual-patient level rather than the level of sample-accession (GSM series number). To avoid misleading, we have revised Supplementary Table 2 to categorize the GSM numbers into the corresponding four patients and added a detailed legend to clarify that patient counts reflect unique individuals. The legends of other Supplementary Tables were also provided in our revision. To further clarify the various composition of datasets in our research, we have improved the corresponding description and presentation. Currently, the scRNA-seq datasets of HCC including:
(I) 14 HCC patients from our own institution who received αPD-1 neoadjuvant immunotherapy;
(II) 60 HCC patients from five public scRNA-seq datasets (GSE149614, GSE156625, GSE202642, GSE189903, and GSE151530), who were not treated with αPD-1 immunotherapy. These datasets were integrated with our data to establish a HCC single-cell atlas with heterogeneous backgrounds, thus enabling the accurate and universal identification of TIME components within HCC patients.
(III) The 40 HCC patients who received αPD-1 immunotherapy from datasets of GSE206325 and skrx2fz79n. These datasets were independent of our in-house 14-patient datasets, specifically used for validation of the analyses findings in our research.

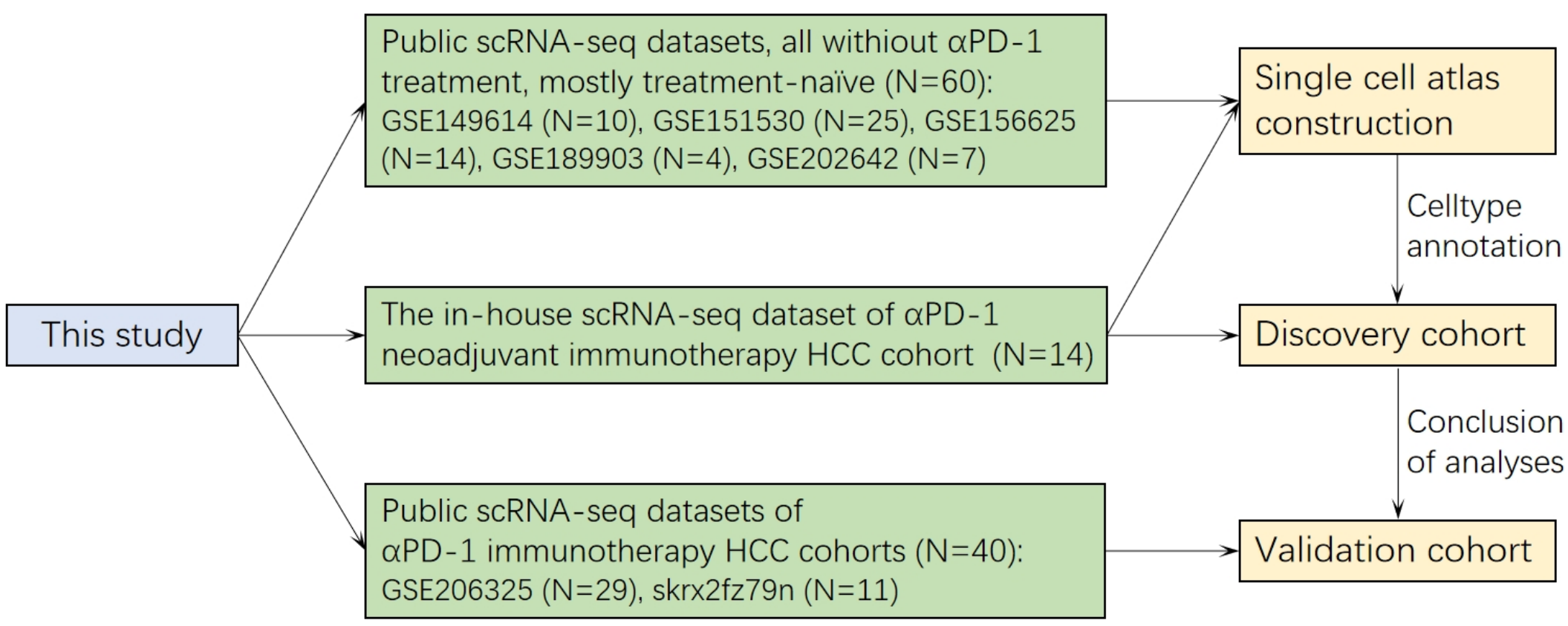

Schematic diagram for the usage of all scRNA-seq datasets of HCC patients in our research.

2. Authors newly added "To demonstrate that LAM and HCC_FA subsets are independent of hepatic steatosis in patients and associated with immunotherapy efficacy, we performed correlation analyses between the proportions of the LAM, HCC_FA subsets and patient BMI (Supplementary Fig. 6a). Neither subset showed a statistically significant correlation with BMI.". I find this argument unconvincing. BMI is an imprecise and indirect surrogate for hepatic steatosis and does not reliably reflect liver fat content, particularly in patients with cancer or advanced liver disease. Therefore, the lack of correlation between LAM or HCC_FA subsets and BMI is insufficient to support the claim that these subsets are independent of hepatic steatosis.

Author's response: We thank the reviewer for this important and constructive comment. We agree that BMI is an indirect and potentially imprecise surrogate for hepatic steatosis, particularly in patients with cancer or underlying liver disease.

To address this concern, we conducted additional correlation analyses using imaging-based measurements of hepatic fat accumulation for the 14 HCC patients from our scRNA-seq cohort. Specifically, liver and spleen attenuation values (Hounsfield units, HU) were obtained from pre-treatment CT scans, the liver-to-spleen (L/S) attenuation ratio was calculated as a radiologic measurement of hepatic steatosis (Zeb, et al.). The liver and spleen CT attenuation values and radiologic assessment of hepatic steatosis from 14 patients were added to Supplementary Table 2, while the spleen CT attenuation value of patient03 was missing due to the spleen resection.

We then assessed the association between L/S ratio and the proportions of LAM or HCC_FA subsets among 13 patients. According to the results below, neither LAM nor HCC_FA proportions showed statistically significant correlation with the L/S ratio, as well as the previous BMI-based results, we have added these results above in Results (line 399-404) and Supplementary Fig. 6a. These results suggest the enrichment of LAM and HCC_FA subsets is unlikely driven by hepatic steatosis alone and instead reflects metabolic reprogramming linked to immunotherapy response.

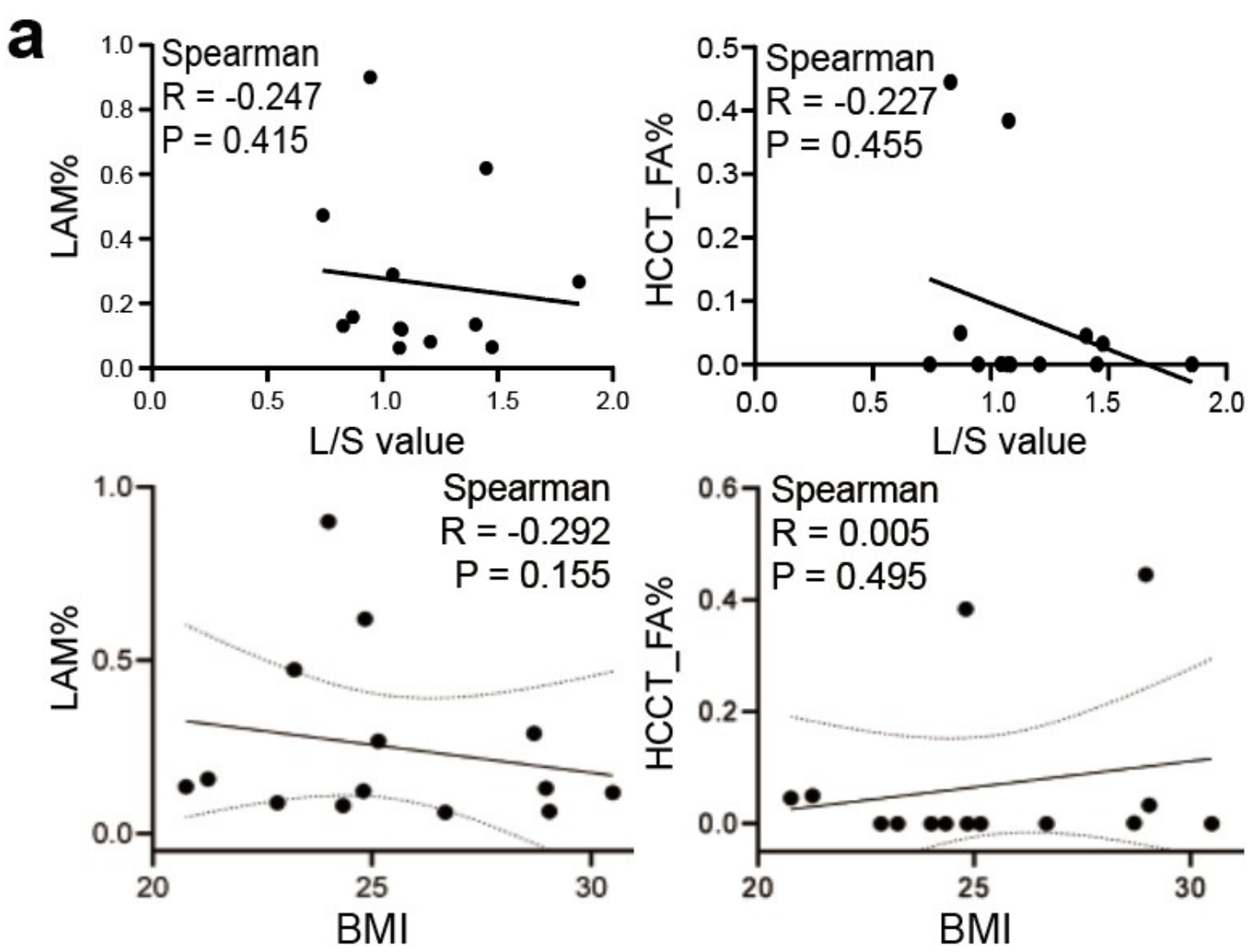

Correlation analysis between L/S attenuation ratio or BMI and proportions of LAM or HCCT_FA subsets among HCC patients of our cohort, from Supplementary Fig. 6a.

Reference:

Zeb I, Li D, Nasir K, Katz R, Larijani VN, Budoff MJ. Computed tomography scans in the

evaluation of fatty liver disease in a population based study: the multi-ethnic study of atherosclerosis. Acad Radiol. 2012 Jul;19(7):811-8.

3. Figure 7: Panel Figure 7e, representative pictures of flow cytometry IC control or FMO control for FABP1 and APOA1 are missing. In graph 7g meaning of "NR" MPR" "pCR" is missing in the figure legend.

Author's response: We thank the reviewer for this helpful comment. To improve transparency and interpretability of flow cytometry results in Fig. 7e, we have included the fluorescence minus one (FMO) control plots for FABP1 and APOA1, as well as other targets in the revised manuscript in Supplementary Fig. 7c, thus clarifying the gating strategy used for defining positive populations.
In addition, we have revised the legend of Fig. 7g to clarify the source of abbreviations. The NR, MPR, and CPR labels shown in Fig. 7g refer to the pathological responses of 14-patient HCC neoadjuvant immunotherapy cohort that analyzed in our scRNA-seq dataset (line 1422-1424), rather than to the experiments of mouse model presented in Fig. 7a–f.

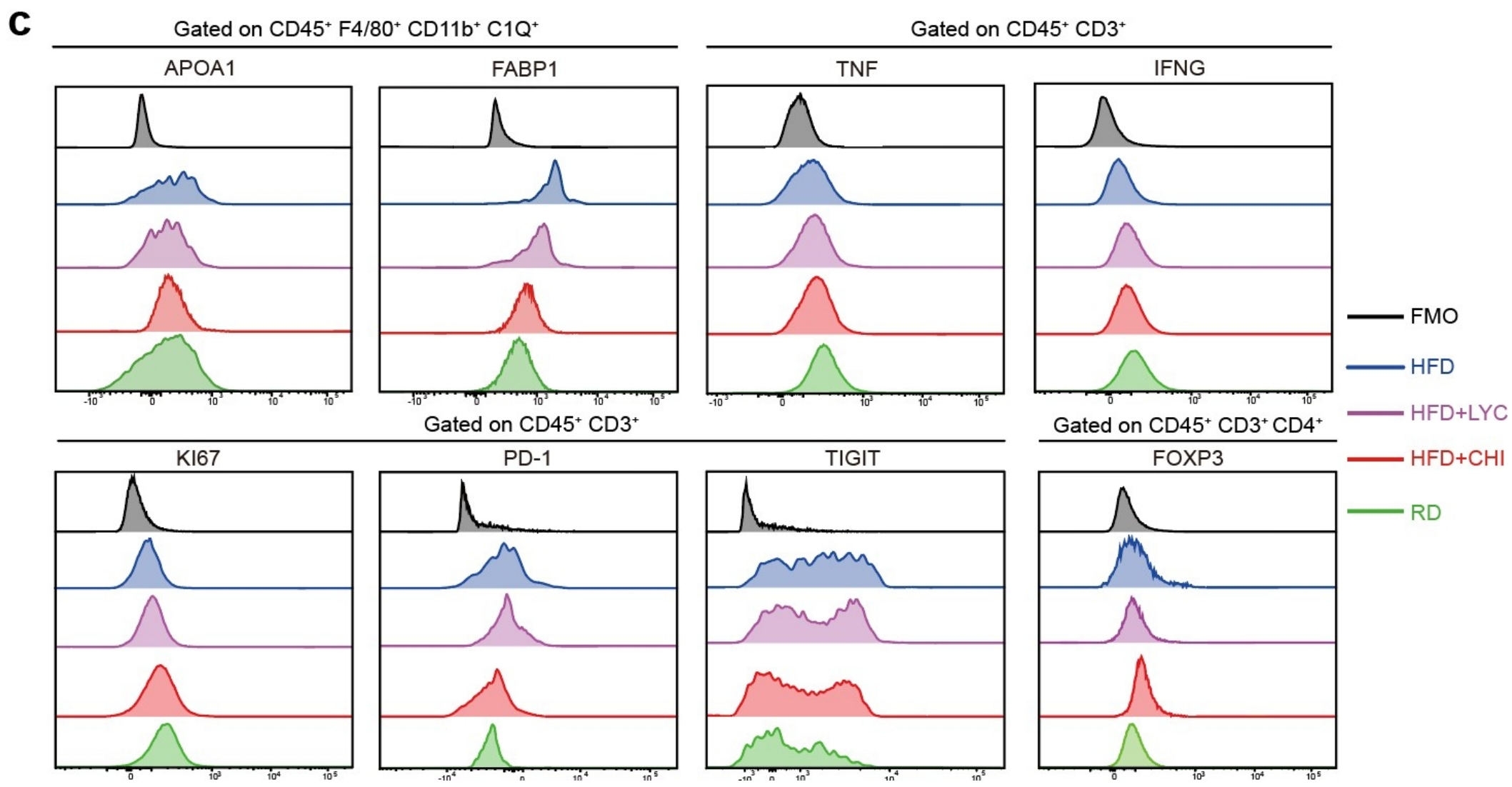

Expression features of HCC mouse model assessed by flow cytometry, from Supplementary Fig. 7c. Features of macrophages (APOA1, FABP1) and T cells (TNF, IFNG, KI67, PD-1, TIGIT and FOXP3 of $CD4^+$ T cells) with positive staining thresholds were defined based on FMO controls for each target to define gating boundaries and ensure discrimination of cell populations.

4. Statistical analysis are not correct for data in Figure 7. Unpaired student's t tests can be used if 2 groups are compared. Authors should either use ANOVA followed by Tukey's multiple-comparison test, if normality was proved. However, given the small sample size (n=5), authors should use Kruskal–Wallis test (non-parametric equivalent of one-way ANOVA). In addition, the graphs in figure 7 show only selected statistical comparisons, with "ns" reported inconsistently. All relevant pairwise comparisons should be displayed, and non-significant results should either be reported systematically or omitted entirely.

Author's response: We thank the reviewer for this important guidance on statistic analysis and we apologize for the improper use of unpaired student's t tests in Fig. 7. We have now re-analyzed all experimental multi-group data in Fig. 7 using a non-parametric framework appropriate for small

sample sizes. Specifically, we firstly applied Kruskal–Wallis test for all panels involving more than two groups, then conducted Dunn's post-hoc multiple-comparison test with multiplicity adjusted for each comparison. The description of statistic analysis in corresponding figure legends of Fig. 7 (line 1426-1430) were updated in the revised manuscript.

In addition, we have revised the figure annotations to ensure consistent reporting of statistic analyses. We now present a standardized set of pre-specified pairwise comparisons in Fig. 7c–f and Fig. 7i (HFD vs HFD+LYC; HFD vs HFD+CHI; HFD+CHI vs HFD+LYC; RD vs HFD+LYC; RD vs HFD+CHI) to directly address the biological hypotheses of diet-induced immunotherapy resistance and corresponding metabolic interventions. Meanwhile, all of the remaining pairwise comparisons, including non-significant results, are provided systematically in corresponding tables of Source data to ensure transparency while maintaining figure readability.

5. Supplementary table was not modified in the new submitted version, there is still the table without liver background etiology etc. (Previously comment 4)

Author's response: We sincerely apologize for this oversight and thank the reviewer for carefully re-evaluating the supplementary materials. In this revised submission, we have now updated Supplementary Table 1 and Supplementary Table 2 to explicitly include liver background etiology for all patients in our HCC immunotherapy cohort, including HBV, HCV, hepatic steatosis, and other liver background etiologies where applicable.

We have carefully verified that the updated table is included in the current version of the supplementary materials. We appreciate the reviewer's attention to this detail and hope these information would contribute to the clinical transparency and interpretability of our study.

Supplementary Table 1. Clinical baseline and treatment line of patients from our scRNA-seq cohort

Sample_ID	Pathology	Resource	Sex	Age	BMI	Hepatic steatosis	Liver CT attenuation value	Spleen CT attenuation value	Baseline tumor stage	Tumor size before treatment (cm)	Tumor size after treatment (cm)
Patient01	HCC	Post_surgery	Female	47	26.67	NO	73	68	T3N0M0	15.4	9.8
Patient02	HCC	Post_surgery	Male	41	29.04	NO	62	42	T3N0M0	9.9	7
Patient03	HCC	Post_surgery	Male	56	22.84	NO	66	Spleen resected	T3N1M0	12.1	10.6
Patient04	HCC	Post_surgery	Male	28	25.14	NO	89	48	T3N0M1	11.5	9
Patient05	HCC	Post_surgery	Male	41	28.69	NO	69	66	T3N0M0	7.1	3.7
Patient06	HCC	Post_surgery	Female	55	23.23	Mild	92	124	T3N0M0	8.1	12.7
Patient07	HCC	Post_surgery	Male	67	24.34	NO	58	48	T3N0M0	6.6	6
Patient08	HCC	Post_surgery	Male	56	20.76	NO	59	42	T3N0M0	14.1	5.1
Patient09	HCC	Post_surgery	Male	59	30.49	NO	90	83	T3N1M0	11.6	10.1
Patient10	HCC	Post_surgery	Male	45	21.26	Mild	88	101	T3N0M0	12.2	6
Patient11	HCC	Post_surgery	Male	65	24.84	NO	58	40	T3N0M0	8.3	7.6
Patient12	HCC	Post_surgery	Male	53	28.96	Mild	97	117	T3N0M0	12	8
Patient13	HCC	Post_surgery	Male	63	24.00	Mild	72	76	T3N0M0	6.1	6.8
Patient14	HCC	Post_surgery	Male	66	24.80	NO	85	79	T3N0M0	13.1	10.7

Sample_ID	High-risk histopathological feature	Macrovascular invasion	Microvasucular invasion	Number of nodules	Baseline AFP (ng/ml)	Perioperative AFP (ng/ml)	PD-1 antibody	Dose Schedule	Cycles	Interval from Last Dose	Perioperative Delays
Patient01	Cancer embolus,Satellite nodule, Poorly	Portal vein	0	Multiple	36926	314.4	Tislelizumab	200mg q3w	3	24	NO
Patient02	Cancer embolus,Poorly differentiated	Portal vein	1	Single	3.75	2.08	Camrelizumab	200mg q3w	3	41	NO
Patient03	Cancer embolus, Satellite nodule	Portal vein	0	Multiple	21	12.96	Tislelizumab	200mg q3w	5	47	NO
Patient04	No	No	0	Multiple	1093	532.2	Tislelizumab	200mg q3w	5	54	NO
Patient05	Cancer embolus,Poorly differentiated	Portal vein	1	Single	2.19	1.81	Camrelizumab	200mg q3w	3	40	YES
Patient06	No	Portal vein	2	Single	16301	10.3	Tislelizumab	200mg q3w	5	32	NO
Patient07	No	No	0	Multiple	1.63	6.23	Tislelizumab	200mg q3w	5	27	NO
Patient08	Cancer embolus	No	0	Single	340.4	8.33	Tislelizumab	200mg q3w	5	27	NO
Patient09	No	No	0	Single	549	113	Camrelizumab	200mg q3w	4	39	NO
Patient10	No	No	0	Single	24.2	4.7	Camrelizumab	200mg q3w	5	38	NO
Patient11	No	No	0	Multiple	766.5	710.9	Camrelizumab	200mg q3w	5	44	NO
Patient12	No	No	0	Single	1483	75.9	Sintilimab	200mg q3w	5	27	YES
Patient13	Satellite nodule	No	1	Multiple	2.78	2.62	Camrelizumab	200mg q3w	3	34	NO
Patient14	No	No	0	Single	4.44	2.77	Camrelizumab	200mg q3w	3	34	NO

Sample_ID	Reasons for Perioperative delay	Treatment period	Adverse Events Level	Adverse Events	Surgical Complications	RECIST	Pathologic response	Efficacy group	Residual tumor (%)	RFS (days)	OS (days)
Patient01	/	9w	/	/	NO	PR	MPR	Responder	20%	62	420
Patient02	/	9w	/	/	NO	SD	non-MPR	Non-responder	50%	131	1032
Patient03	/	15w	2	Transaminase Elevation	NO	SD	MPR	Responder	20%	792	992
Patient04	/	15w	2	Proteinuria	NO	SD	MPR	Responder	15%	34	573
Patient05	Hypoproteinemia	9w	3	Hypoproteinemia, Proteinuria	NO	PR	non-MPR	Non-responder	55%	723	800
Patient06	/	15w	2	Hypertension, Dizziness	NO	PD	MPR	Responder	45%	637	880
Patient07	/	15w	2	Hypertension, Gingival Bleeding	NO	SD	CPR	Responder	0	648	782
Patient08	/	15w	3	Hypothyroidism, Fatigue	NO	PR	non-MPR	Non-responder	60%	90	285
Patient09	/	12w	2	Transaminase Elevation	NO	SD	non-MPR	Non-responder	65%	622	730
Patient10	/	15w	3	Hypertension, Dizziness	NO	PR	MPR	Responder	10%	571	876
Patient11	/	15w	2	Loss of Appetite, Nausea	NO	SD	non-MPR	Non-responder	70%	566	724
Patient12	Headache accompanied by right-sided eyelid ptosis, considering neurologic adverse event	15w	3	Headache, Upper Eyelid Ptosis	Wound Oozing	PR	MPR	Responder	10%	601	847
Patient13	/	9w	2	Nausea, Abdominal Discomfort	NO	SD	non-MPR	Non-responder	80%	80	230
Patient14	/	9w	2	Hypertension	NO	SD	CPR	Responder	0	542	590

Clinical information and treatment line of our scRNA-seq HCC immunotherapy cohort, from Supplementary Table 1. Including baseline demographic, clinical treatment, and pathological features of 14 HCC patients that received αPD-1 immunotherapy from our cohort.

6. Supplementary Fig. 7 – The authors have added multiparametric flow cytometric analyses of tumor-infiltrating lymphocytes; however, the presented data appear to contain inaccuracies. It is written that the axis y represents CD45-PE-Cy7 in all graphs, which does not seem to be correct. Moreover, representative pictures of flow cytometry IC control or FMO control need to be included for all conditions.

Author's response: We thank the reviewer for carefully evaluating Supplementary Fig. 7 and highlighting the lack of clarity in the presentation. We apologize for the confusion caused by the previous axis labeling.

In Supplementary Fig. 7d–f, all analyses were performed within pre-gated live $CD45^+$ $CD3^+$ tumor-infiltrating T cells and analyses of Supplementary Fig. 7g were further gated on $CD45^+$ $CD3^+$ $CD4^+$. The CD45 was displayed on the y-axis in these representative plots for purpose of uniform visualization and was not used to define additional gating thresholds at this stage. The positive expression of pre-gated population above on TNF, IFN-γ, Ki-67, PD-1, TIGIT and FOXP3 was determined exclusively based on x-axis thresholds established using fluorescence minus one (FMO) controls. We agree that this was not sufficiently explained in the original figure legend.

To address this issue, we have now:

Added representative FMO control plots in Supplementary Fig. 7c for the relevant targets used to define positive populations.

Revised the legends of Fig.7e-f to explicitly state that gating was performed within pre-defined macrophages or T cells, and the FMO-based thresholds were applied for all experimental groups.

We believe these revisions could clarify the gating strategy and improve the transparency and reproducibility of the flow cytometry analyses.

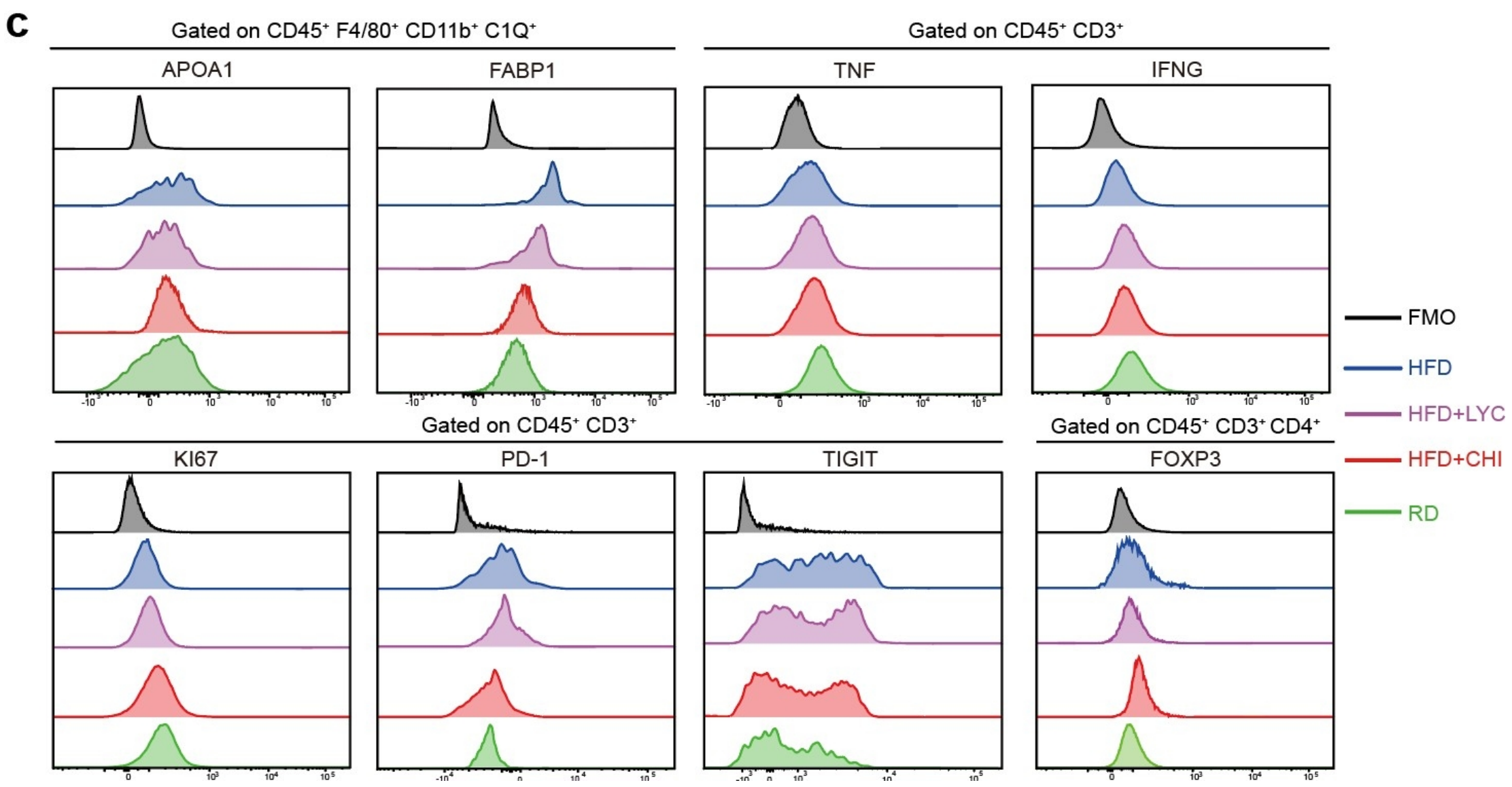

Expression features of HCC mouse model assessed by flow cytometry, from Supplementary Fig. 7c. Features of macrophages (APOA1, FABP1) and T cells (TNF, IFNG, KI67, PD-1, TIGIT and FOXP3 of $CD4^+$ T cells) with positive staining thresholds were defined based on FMO controls for each target to define gating boundaries and ensure discrimination of cell populations.

7. I could not find any publicly available clinical-trial registration or results for ClinicalTrials.gov identifier: NCT06571369

Author's response: We sincerely thank the reviewer for carefully checking the trial registration and apologize for the confusion caused by a manually transcription error in the ClinicalTrials.gov identifier provided in the previous version of our manuscript. The correct registration number is [NCT06571396] and publicly accessible at https://www.clinicaltrials.gov/study/NCT06571396. We have carefully revised the manuscript and supplementary files to correct the identifier and verified that the updated registration number is accurate throughout the text.

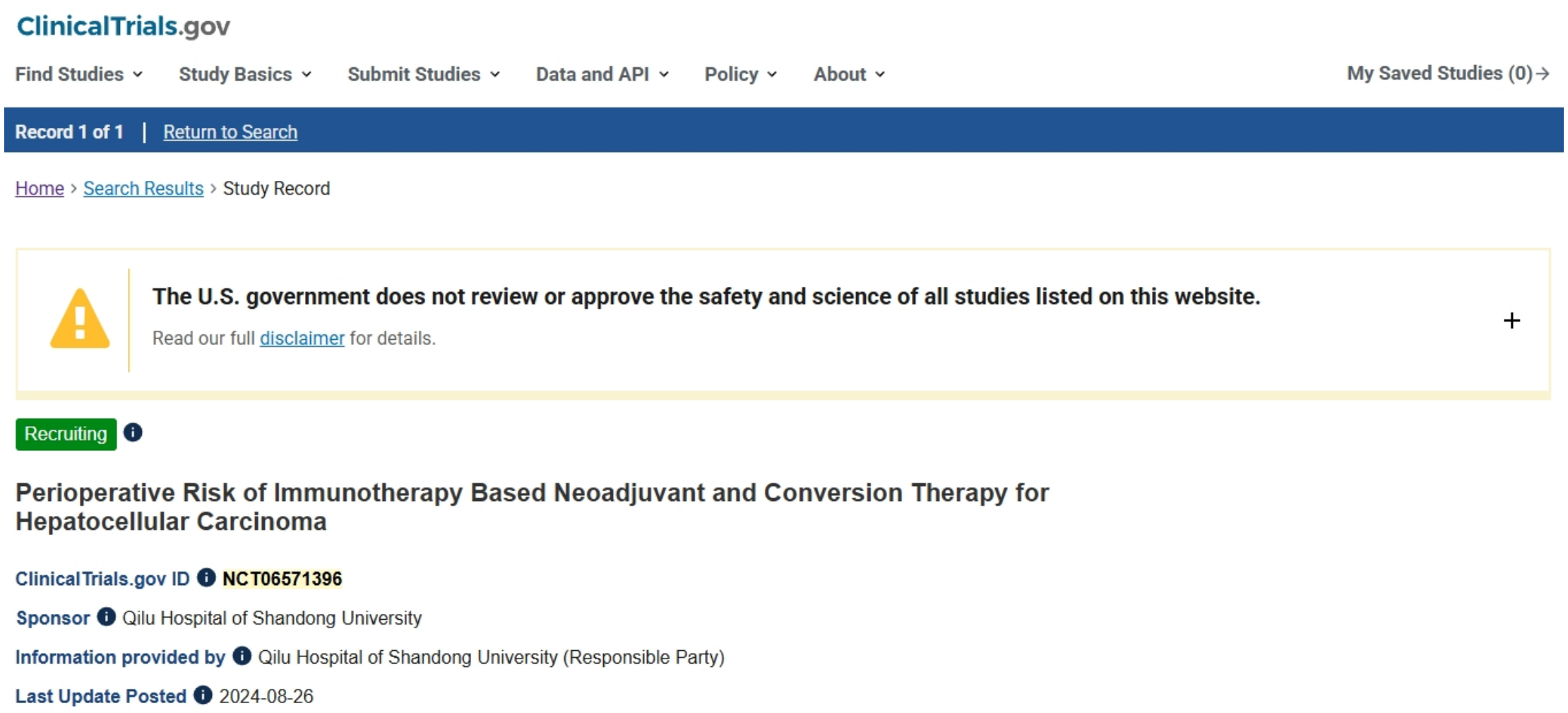

The page of clinical-trial registration under ClinicalTrials.gov identifier: NCT06571396.

8. Newly added paragraphs for the method part contain inaccuracies. For instance, lines982-986.

Author's response: We thank the reviewer for this important comment. We have revised the Methods section of line 982-986 in our previous manuscript to clarify the preparation and administration of chiglitazar. Specifically, we described it in detail that the chiglitazar was first dissolved in DMSO to prepare a stock solution (10 mg/ml) and subsequently diluted with sterile normal saline to a final volume of 0.4 mL (1 mg/ml) per mouse prior to oral gavage. The revised description has been incorporated into the Methods section (line 1034-1040).

In addition, we have expanded the experimental details in Western blot section and RT-qPCR section of the Methods to improve methodological transparency and ensure that the experimental procedures can be fully reproduced (line 1076-1107).

Method

C57BL/6J (male) mice were purchased from Beijing Vital River Experimental Animal Technology (Beijing, China). Chiglitazar was purchased from Chipscreen Biosciences Ltd. (Shenzhen, China). Chiglitazar was first dissolved in dimethyl sulfoxide (DMSO) to prepare a stock solution (10 mg/ml) and subsequently diluted with sterile normal saline to a final administration volume of 0.4 mL (1 mg/ml) per mouse for oral gavage. All animals were maintained in a specific pathogen-free (SPF) barrier facility at the Animal Center of Shandong University.

9. Line 1002-1003: "The survival status of HCC mice was observed daily until a fatal outcome for all animals." Why is this sentence added? The experiment was not designed as a

survival study.

Author’s response: We thank the reviewer for pointing this out. We agree that the experiment was not designed as a survival study, and apologize for the confusion caused by inclusion of this sentence. The primary endpoints of the mice experiments were tumor burden and immunological analyses at predefined time points. The original description was intended to indicate that animals were monitored until the experimental endpoint, at which time they were euthanized for tissue collection, rather than to imply a survival analysis. The previous sentence has been removed in our revised manuscript to avoid misunderstanding. Additionally, all animal experiments were conducted following the institutional ethical guidelines, and humane endpoints were applied as approved by the Ethics Committee of Qilu Hospital of Shandong University.

10. English should be carefully corrected in the whole manuscript and the supplementary part. For instance “In particular, we discover that the lipid-associated macrophages (LAM)characterized by C1QA, APOA1 and FABP1 expression, is closely related to immunosuppression among HCC patients.” Modify : “discover” → “discovered” past tense; “is” → “are” the plural subject “macrophages”

Author’s response: We thank the reviewer for this careful reading and helpful suggestion. We have thoroughly revised the manuscript and Supplementary Information to improve grammatical accuracy and language consistency. The sentence mentioned by the reviewer has been corrected to past tense (“discovered”) and the verb agreement has been revised to reflect the plural subject (“are”) (line 107-108). In addition, we have conducted further language editing to improve the clarity and rigor in our revised manuscript following the suggestion from the reviewer.

Introduction

In particular, we discovered that the lipid-associated macrophages (LAM) characterized by C1QA, APOA1 and FABP1 expression, are closely related to immunosuppression among HCC patients.

11. Abbreviations need to be corrected. Tables and Supplementary tables should include legends where all Abbreviations used in the tables are explained. For instance, abbreviations “CNV”, PR, PD, SD, MPR, CPR, etc…. Some abbreviations are introduced several times in the text, for instance, lipid-associated macrophage (LAM)

Author’s response: We thank the reviewer for this important suggestion. We have carefully reviewed and standardized all abbreviations throughout the main manuscript and Supplementary Information. All tables and Supplementary tables have been updated to include dedicated legends, in which all abbreviations (e.g., CNV, PR, PD, SD, MPR, CPR, etc.) are defined or explained.

In addition, we have carefully revised the main text to ensure that each abbreviation is introduced only once at first mention and used consistently thereafter. For example, “lipid-associated macrophage (LAM)” is now defined at its first occurrence (line 38) and not redundantly reintroduced in subsequent sections.

Reviewer #2 (Remarks to the Author):

The authors have thoroughly addressed all comments.

Reviewer #3:

Peng et al. have substantially revised and improved the manuscript in terms of both clarity and scientific rigor. In particular, the authors have addressed the major conceptual and technical concerns raised in the previous round of review. The additional analyses and methodological clarifications have strengthened the overall robustness of the study. However, there remain several areas where clarification, refinement, or minor additional adjustments would further improve the manuscript and enhance its interpretability. Below are specific comments related to individual points for the authors' consideration.

Related to point 1:
I appreciate the authors' efforts to expand the clinical description and provide inclusion/exclusion criteria and supplementary clinical tables. However, several clinically important elements remain insufficiently clarified. It would be ideal if these data could be provided, baseline tumor characteristics - including tumor stage, number of nodules, tumor size before and after treatment, presence of macro- or microvascular invasion, and other high-risk histopathological features. These can help better interpreting recurrence-free survival and pathological response outcomes. In addition, the units for RFS, OS, and residual tumor percentage should be explicitly specified. The rationale for administering 3–5 cycles of anti–PD-1 therapy prior to surgery remains unclear, and it is not evident whether treatment duration was standardized across patients or individualized. It would be ideal to list out in the method section about the authority approved treatment regimen. Finally, baseline and perioperative AFP measurements, and any associations with treatment response, should be reported if available. Inclusion of these details - at least within the Supplementary Information - would substantially improve the clinical transparency, reproducibility, and interpretability of the study in relation to existing neoadjuvant immunotherapy literature.
Since the authors have now provided RFS and OS data, these outcomes should be formally visualized using Kaplan–Meier survival analyses stratified by their pathological response classification. This would substantially improve the clinical interpretability of the study and allow readers to directly assess the prognostic relevance of the reported response categories.
While I understand that the authors have followed the 2024 Chinese clinical guidelines for hepatocellular carcinoma in defining MPR as >50% tumor regression, it would be important to additionally perform survival analyses using a more widely accepted pathological response cutoff (e.g., >70% tumor regression), as reported by D'Alessio et al. (PMID:39437804). This additional Kaplan–Meier analysis could be presented in the Supplementary Materials if space is a concern. Including such a comparison would improve the global comparability of the study and enhance its relevance to the broader international neoadjuvant immunotherapy literature, while allowing the main analyses to remain aligned with the 2024 Chinese guideline classification.
The authors should explicitly cite the 2024 Chinese clinical guidelines for hepatocellular carcinoma in the main text when introducing their pathological response classification criteria. In addition, the corresponding definition (e.g., MPR defined as >50% tumor regression) should be clearly stated in the relevant table or figure legend, with the guideline

properly referenced. This will ensure transparency of the classification framework and allow readers to clearly understand the rationale behind the chosen cutoff.

Author's response: We thank the reviewer for the thoughtful comments and for highlighting a series of important issues in clinical, we believe that supplement these information could further improve the transparency and interpretability of our study. Therefore, we have carefully revised the manuscript and Supplementary Information to address these points, as detailed below.

1. Baseline tumor characteristics and clinical variables: We agree that additional baseline tumor characteristics would facilitate better interpretation of recurrence-free survival and pathological response outcomes. In the revised manuscript, we have expanded the corresponding clinical information of HCC immunotherapy cohort. Baseline tumor characteristics, including tumor stage, number of nodules, tumor size before and after treatment, vascular invasion status, baseline and perioperative alpha-fetoprotein (AFP) measurements, and other relevant features, have now been summarized in Supplementary Table 1. These additions provide a more comprehensive overview on the clinical characteristics of our HCC immunotherapy cohort.

In addition, the units for recurrence-free survival (RFS) and overall survival (OS) are "days" and the unit of residual tumor is "percentage". These units have now been explicitly specified in the revised Supplementary Table 1.

2. Neoadjuvant treatment regimen and rationale for 3–5 cycles: We appreciate the reviewer's suggestion to clarify the treatment regimen. Briefly, patients received 3–5 cycles of αPD-1 therapy prior to surgery according to the institutional clinical protocol approved by regulatory authority and based on contemporary clinical practice guidelines for hepatocellular carcinoma (Bi, et al.).

We note that in real-world clinical practice, the number of neoadjuvant treatment cycles may vary across patients due to differences in treatment response, surgical scheduling, and individual clinical conditions. In this study, the specific number of treatment cycles was determined by the treating clinical team based on surgical timing and patient condition, resulting in a range of 3–5 cycles prior to surgery, which represents a clinically reasonable and standardized treatment window in our institution. Notably, this treatment duration is also broadly consistent with the neoadjuvant treatment cycles adopted in several reported clinical studies of PD-1 blockade in hepatocellular carcinoma (Li, et al; Xia, et al; Cao, et al).

3. Kaplan–Meier survival analyses stratified by pathological response: We thank the reviewer for this important suggestion. In the revised manuscript, we have now performed Kaplan–Meier survival analyses using >70% and >50% tumor regression cutoff (according to D'Alessio et al. and 2024 Chinese clinical guidelines, respectively) for recurrence-free survival (RFS) and overall survival (OS) stratified by pathological response categories. These analyses are presented in Supplementary Fig. 1e, allowing readers to directly assess the prognostic relevance of the reported pathological response classifications.

4. Citation and clarification of pathological response criteria: Following the reviewer's suggestion, we now explicitly cite the 2024 Chinese clinical guidelines for hepatocellular carcinoma in the main text (as reference No.28) when introducing the pathological response classification (line 146-150). We have also revised the legend of Fig. 1e and Supplementary Fig. 1d to clearly state the definition of non-MPR, MPR and CPR. These clarifications ensure transparency in the classification framework used in this study.

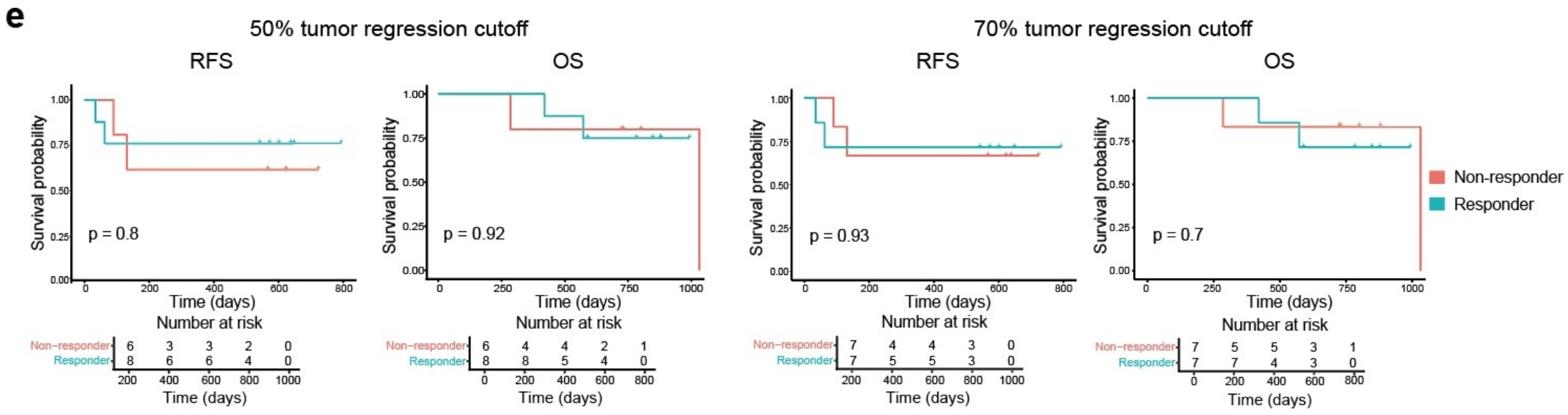

Kaplan–Meier survival analyses using >70% and >50% tumor regression cutoff for RFS and OS stratified by pathological response categories, from Supplementary Fig. 1e.

Supplementary Table 1. Clinical baseline and treatment line of patients from our scRNA-seq cohort

Sample_ID	Pathology	Resource	Sex	Age	BMI	Hepatic steatosis	Liver CT attenuation value	Spleen CT attenuation value	Baseline tumor stage	Tumor size before treatment (cm)	Tumor size after treatment (cm)
Patient01	HCC	Post_surgery	Female	47	26.67	NO	73	68	T3N0M0	15.4	9.8
Patient02	HCC	Post_surgery	Male	41	29.04	NO	62	42	T3N0M0	9.9	7
Patient03	HCC	Post_surgery	Male	56	22.84	NO	66	Spleen resected	T3N1M0	12.1	10.6
Patient04	HCC	Post_surgery	Male	28	25.14	NO	89	48	T3N0M1	11.5	9
Patient05	HCC	Post_surgery	Male	41	28.69	NO	69	66	T3N0M0	7.1	3.7
Patient06	HCC	Post_surgery	Female	55	23.23	Mild	92	124	T3N0M0	8.1	12.7
Patient07	HCC	Post_surgery	Male	67	24.34	NO	58	48	T3N0M0	6.6	6
Patient08	HCC	Post_surgery	Male	56	20.76	NO	59	42	T3N0M0	14.1	5.1
Patient09	HCC	Post_surgery	Male	59	30.49	NO	90	83	T3N1M0	11.6	10.1
Patient10	HCC	Post_surgery	Male	45	21.26	Mild	88	101	T3N0M0	12.2	6
Patient11	HCC	Post_surgery	Male	65	24.84	NO	58	40	T3N0M0	8.3	7.6
Patient12	HCC	Post_surgery	Male	53	28.96	Mild	97	117	T3N0M0	12	8
Patient13	HCC	Post_surgery	Male	63	24.00	Mild	72	76	T3N0M0	6.1	6.8
Patient14	HCC	Post_surgery	Male	66	24.80	NO	85	79	T3N0M0	13.1	10.7
**Sample_ID**	**High-risk histopathological feature**	**Macrovascular invasion**	**Microvasucular invasion**	**Number of nodules**	**Baseline AFP (ng/ml)**	**Perioperative AFP (ng/ml)**	**PD-1 antibody**	**Dose Schedule**	**Cycles**	**Interval from Last Dose**	**Perioperative Delays**
Patient01	Cancer embolus,Satellite nodule, Poorly	Portal vein	0	Multiple	36926	314.4	Tislelizumab	200mg q3w	3	24	NO
Patient02	Cancer embolus,Poorly differentiated	Portal vein	1	Single	3.75	2.08	Camrelizumab	200mg q3w	3	41	NO
Patient03	Cancer embolus, Satellite nodule	Portal vein	0	Multiple	21	12.96	Tislelizumab	200mg q3w	5	47	NO
Patient04	No	No	0	Multiple	1093	532.2	Tislelizumab	200mg q3w	5	54	NO
Patient05	Cancer embolus,Poorly differentiated	Portal vein	1	Single	2.19	1.81	Camrelizumab	200mg q3w	3	40	YES
Patient06	No	Portal vein	2	Single	16301	10.3	Tislelizumab	200mg q3w	5	32	NO
Patient07	No	No	0	Multiple	1.63	6.23	Tislelizumab	200mg q3w	5	27	NO
Patient08	Cancer embolus	No	0	Single	340.4	8.33	Tislelizumab	200mg q3w	5	27	NO
Patient09	No	No	0	Single	549	113	Camrelizumab	200mg q3w	4	39	NO
Patient10	No	No	0	Single	24.2	4.7	Camrelizumab	200mg q3w	5	38	NO
Patient11	No	No	0	Multiple	766.5	710.9	Camrelizumab	200mg q3w	5	44	NO
Patient12	No	No	0	Single	1483	75.9	Sintilimab	200mg q3w	5	27	YES
Patient13	Satellite nodule	No	1	Multiple	2.78	2.62	Camrelizumab	200mg q3w	3	34	NO
Patient14	No	No	0	Single	4.44	2.77	Camrelizumab	200mg q3w	3	34	NO
**Sample_ID**	**Reasons for Perioperative delay**	**Treatment period**	**Adverse Events Level**	**Adverse Events**	**Surgical Complications**	**RECIST**	**Pathologic response**	**Efficacy group**	**Residual tumor (%)**	**RFS (days)**	**OS (days)**
Patient01	/	9w	/	/	NO	PR	MPR	Responder	20%	62	420
Patient02	/	9w	/	/	NO	SD	non-MPR	Non-responder	50%	131	1032
Patient03	/	15w	2	Transaminase Elevation	NO	SD	MPR	Responder	20%	792	992
Patient04	/	15w	2	Proteinuria	NO	SD	MPR	Responder	15%	34	573
Patient05	Hypoproteinemia	9w	3	Hypoproteinemia, Proteinuria	NO	PR	non-MPR	Non-responder	55%	723	800
Patient06	/	15w	2	Hypertension, Dizziness	NO	PD	MPR	Responder	45%	637	880
Patient07	/	15w	2	Hypertension, Gingival Bleeding	NO	SD	CPR	Responder	0	648	782
Patient08	/	15w	3	Hypothyroidism, Fatigue	NO	PR	non-MPR	Non-responder	60%	90	285
Patient09	/	12w	2	Transaminase Elevation	NO	SD	non-MPR	Non-responder	65%	622	730
Patient10	/	15w	3	Hypertension, Dizziness	NO	PR	MPR	Responder	10%	571	876
Patient11	/	15w	2	Loss of Appetite, Nausea	NO	SD	non-MPR	Non-responder	70%	566	724
Patient12	Headache accompanied by right-sided eyelid ptosis, considering neurologic adverse event	15w	3	Headache, Upper Eyelid Ptosis	Wound Oozing	PR	MPR	Responder	10%	601	847
Patient13	/	9w	2	Nausea, Abdominal Discomfort	NO	SD	non-MPR	Non-responder	80%	80	230
Patient14	/	9w	2	Hypertension	NO	SD	CPR	Responder	0	542	590

Clinical information and treatment line of our scRNA-seq HCC immunotherapy cohort, from Supplementary Table 1. Including baseline demographic, clinical treatment, and pathological features of 14 HCC patients received αPD-1 immunotherapy from our cohort.

Reference:

Bi X, Zhao H, Zhao H, Li G, Wang X, Chen B, Zhang W, Che X, Huang Z, Han Y, Jiang L, Sun Y, Yang Z, Zhou J, Zhang Y, Zhu Z, Chen M, Cheng S, Cai J. Consensus of Chinese Experts on Neoadjuvant and Conversion Therapies for Hepatocellular Carcinoma: 2023 Update. Liver Cancer. 2024 Sep 3;14(2):223-238.

Li Z, Liu J, Zhang B, Yue J, Shi X, Cui K, Liu Z, Chang Z, Sun Z, Li M, Yang Y, Ma Z, Li L, Zhang C, Sun P, Zhong J, Zhao L. Neoadjuvant tislelizumab plus stereotactic body radiotherapy and adjuvant tislelizumab in early-stage resectable hepatocellular carcinoma: the Notable-HCC phase 1b trial. Nat Commun. 2024 Apr 16;15(1):3260.

Xia Y, Tang W, Qian X, Li X, Cheng F, Wang K, Zhang F, Zhang C, Li D, Song J, Zhang H, Zhao J, Yao A, Wu X, Wu C, Ji G, Liu X, Zhu F, Qin L, Xiao X, Deng Z, Kong X, Li S, Yu Y, Xi W, Deng W, Qi C, Liu H, Pu L, Wang P, Wang X. Efficacy and safety of camrelizumab plus apatinib during the perioperative period in resectable hepatocellular carcinoma: a single-arm, open label,

phase II clinical trial. J Immunother Cancer. 2022 Apr;10(4):e004656.
Cao F, Yang Y, Si T, Luo J, Zeng H, Zhang Z, Feng D, Chen Y, Zheng J. The Efficacy of TACE Combined With Lenvatinib Plus Sintilimab in Unresectable Hepatocellular Carcinoma: A Multicenter Retrospective Study. Front Oncol. 2021 Dec 20;11:783480.

Related to point 2:

I thank the authors for their detailed and thoughtful response regarding the relationship of the current study to previously published neoadjuvant anti–PD-1 translational analyses in HCC, including the Nature Medicine (PMID: 37322116) and Molecular Cancer (PMID: 40205519) studies, as well as other recent neoadjuvant immunotherapy trials. The clarification that the present work addresses a complementary mechanistic layer - focusing on a myeloid-driven metabolic and fibrotic program rather than primarily on T cell instructional niches - is helpful and improves conceptual positioning. That said, I believe the manuscript would benefit from a more explicit and structured cross-comparison to prior datasets in order to clearly delineate what is novel versus confirmatory, or at minimum to discuss these similarities and distinctions more systematically in the Discussion section. While the authors provide a narrative differentiation in the letter of reply to reviewers, a clearer comparison within the manuscript itself, such as highlighting shared and divergent macrophage phenotypes, key marker overlap, and differences in mechanistic interpretation, would strengthen the transparency of the study's contribution. A concise comparative summary (either textual or tabulated in the Supplementary Information) outlining clinical endpoints, immune subsets identified, and methodological approaches across studies would further help readers contextualize the current findings within the rapidly evolving neoadjuvant HCC immunotherapy literature. Overall, the authors' argument for complementarity is reasonable, and the manuscript has improved substantially. Clarifying the degree of overlap and divergence with landmark studies would further enhance its clarity and perceived novelty.

Author's response: We sincerely thank the reviewer for the insightful suggestion to provide a clearer and more structured cross-comparison with prior HCC neoadjuvant αPD-1 translational studies. We agree that explicitly delineate the overlaps and divergences would improve the conceptual clarity, and accurately position our work in the rapidly evolving immunotherapy area.
Therefore, we have now revised the Discussion of manuscript and included a part that contrasts our findings with previously published landmark studies (line 662-673). In addition, we have added Supplementary Table 13 that summarizes the characteristics and cohorts of these key studies with ours, including clinical settings, clinical endpoints, methodological approaches, identification of key immune subsets, and the characteristics of macrophages.
These revisions clarify both shared observations and important distinctions. The prior studies primarily focused on T cell–centric mechanisms, such as functional niches, clonotype expansion dynamics, or chemokine-driven trafficking, while our study mainly identified a lipid-associated macrophage (LAM) state with significant metabolic characteristics. Importantly, whereas earlier reports focused on immune activation or exclusion patterns, our study defines a myeloid driven metabolic–fibrotic and immunosuppress program associated with HCC immunotherapy resistance.
Beyond descriptive characterization, we further demonstrate that pharmacologic modulation of

this metabolic axis reshapes the tumor immune microenvironment and enhances αPD-1 efficacy *in vivo*, suggesting the therapeutic tractability of targeting the identified LAM-associated program.
We believe that these content and summary would improve the presentation of clarity and novelty for our study relative to existing findings, further help the readers contextualize the current findings within the rapidly evolving research area of neoadjuvant HCC immunotherapy.

Supplementary Table 13. Cross-comparison of αPD-1 immunoherapy studies in HCC

Category	Clinical setting	Patient number	Clinical endpoint	Methodological approaches	Metabolic reprogramming	Macrophage markers characterized	Core discovery of immune subsets
**Nature Medicine (PMID: 37322116)**	αPD-1	20	Surgery (Pathological response)	scRNA-seq+ST	No	Not specifically defined; general TAM signatures	T cell functional niche
**Molecular Cancer (PMID: 40205519)**	Neoadjuvant αPD-1	16	Surgery (Pathological response)	scRNA-seq+ST	No	Limited TAM characterization	T cell reprogramming related to therapeutic response
**Journal of Hepatology (PMID: 36708811)**	αPD-1	11	Surgery (Pathological response)	scRNA-seq+ST	No	SPP1, TREM2 TAM consisting tumor immune barrier	Immune exclusion barrier architecture
**J Immunother Cancer (PMID: 41330615)**	Neoadjuvant αPD-1	19	Surgery (Pathological response)	Bulk RNA-seq+Bulk TCR-seq	No	Not macrophage-focused	Systemic TCR clonotype dynamics
**Nature Communications (PMID: 38627377)**	Neoadjuvant αPD-1	20	Surgery (Pathological response) DFS and OS rates	Bulk RNA-seq+Bulk TCR-seq	No	Not macrophage-focused	Stereotactic body radiotherapy may systemically facilitate TCR clonal expansion
**Int J Surg (PMID: 38995162)**	Neoadjuvant αPD-1 +Lenvatinib	22	Surgery (Pathological response) RFS rates	No	No	No	No
**Nature Cancer (PMID: 34796337)**	Neoadjuvant αPD-1	15	Surgery (Pathological response)	Nanostring analysis+CyTOF	No	Not specifically defined; general TAM signatures	Spatial arrangement of B cells and plasma cells in responders
**This Study**	Neoadjuvant αPD-1	14	Surgery (Pathological response) RFS and OS rates	scRNA-seq+ST	Intervention of lipid metabolism reprogramming, including modulation of PPARs to improve immunotherapy outcomes	$C1QA^+$ $APOA1^+$ $FABP1^+$ $SPP1^+$ LAM induced lipid metabolism–associated immunosuppression and fibrotic function	Identification and modulation of immunosuppressive LAM to improve HCC immunotherapy

Cross-comparison of HCC neoadjuvant αPD-1 translational studies, from Supplementary Table 13. This table summarizes study design, patient cohorts, clinical endpoints, methodological strategies, characterized macrophages and core discoveries across representative reports, highlighting conceptual commonalities and distinctions relative to our study.

Related to point 3:

I thank the authors for substantially expanding the Methods section to describe tissue dissociation procedures, sequencing workflow, and quality control metrics. The inclusion of digestion conditions, cell viability thresholds, sequencing depth, median genes per cell, UMI counts, and library saturation meaningfully improves transparency. The addition of DoubletFinder-based doublet detection is also appropriate and increases confidence in data quality. However, a few clarifications would further strengthen methodological rigor:

1. While the digestion temperature and enzyme (type I collagenase) are now described, the duration of digestion is not explicitly stated and if the digestion time was standardized across samples.

2. an additional supplementary table showing sample-level QC would be helpful, for example, cells captured per sample, median genes and UMIs per sample, percentage of mitochondrial reads, and doublet rate per sample.

3. is any batch correction method used?

Overall, the methodological reporting has improved significantly and is now much more transparent. Addressing the above points would further strengthen the robustness and reproducibility of the single-cell dataset.

Author's response: We sincerely thank the reviewer for the positive assessment of the expanded Methods section and for the constructive suggestions to further improve methodological transparency.

1. Duration and standardization of sample digestion

We have now clarified the duration of enzymatic digestion in the revised Methods section. Tumor tissues were digested with type I collagenase at 37°C for 20 minutes, and the digestion duration was standardized across all samples to ensure procedural consistency. This information has been added to the Methods section (line 782-788).

2. Sample-level quality control metrics

According to reviewer's suggestion, we have added Supplementary Table 14 to summarize the sample-level quality control metrics. This table includes the number of cells captured, median genes detected, median UMI counts, median percentage of mitochondrial reads, and estimated doublet rate per sample by using R package DoubletFinder. These additions further enhance the transparency and strictness of our scRNA-seq dataset.

Supplementary Table 14. Sample-level quality control metrics for single-cell RNA sequencing data

Patient	Cells per sample	Median genes per cell	Median UMI counts per cell	Percentage of mitochondrial reads	Doublet rate per sample
Patient01	3904	739	1465	1.4987323	0.02%
Patient02	5436	1178	1324	1.7257494	0.10%
Patient03	5831	1426	1492	1.4663798	0.05%
Patient04	8182	2099	4881	2.1292548	0.14%
Patient05	10678	1787	4556	1.4227449	0.04%
Patient06	9388	1811.5	4697	1.393781	0.05%
Patient07	10180	1581	1725	2.5503356	0.04%
Patient08	10462	1143	1578	2.1451484	0.11%
Patient09	10843	1082	1549	1.7532874	0.01%
Patient10	5826	1828.5	4597	0.7201276	0.06%
Patient11	8477	1584	3852	2.7114967	0.07%
Patient12	9443	1573	1660	0.506791	0.10%
Patient13	11140	1379	1632	3.6231884	0.13%
Patient14	7776	1787	4691	1.790329	0.08%

Sample quality control metrics for scRNA-seq data of our HCC immunotherapy cohort, from Supplementary Table 14. Summary of cells captured, median genes and UMIs, median percentage of mitochondrial reads, and estimated doublet rates of 14 patients from our cohort.

3. Correction of batch effect

We have clarified the batch correction strategy in the revised Methods section. Specifically, we applied R package harmony with default parameters to correct the potential batch effects across patients and sequencing conditions. The corrected embeddings were used for downstream clustering and visualization. The relevant details are now included in the Methods section (line 833-839).

We believe these additions further strengthen the robustness and reproducibility of the dataset and we appreciate the reviewer's helpful guidance in improving our methodological clarity.

Related to point 4:

I thank the authors for their detailed response and, importantly, for generating additional in-house spatial transcriptomic (ST) data paired with their single-cell cohort. The addition of six paired spatial transcriptomic samples substantially strengthens the manuscript and meaningfully addresses the concern regarding reliance on external public datasets. The integration of spatial data directly from the same clinical cohort provides much stronger support for the reported enrichment of LAMs in non-responders.

Author's response: We thank the reviewer for the positive feedback and for recognizing the value of these additionally generated in-house spatial transcriptomic (ST) data. We agree that integrating spatial transcriptomic profiles with our single-cell dataset strengthens the robustness of our study. The inclusion of six paired ST samples from identical clinical cohort provides spatial validation for the enrichment of LAM populations in non-responders and further supports our findings.

Related to point 5:

I thank the authors for the substantial additional analyses performed in response to this comment. The integrated cross-dataset comparison using the Liu et al. (PMID: 36708811) HCC immunotherapy scRNA-seq dataset, the Harmony-based batch correction, and the side-by-side UMAP visualization of predefined SPP1^{+} macrophages and the LAM cluster significantly strengthen the manuscript. The updated gene ranking criteria and the new figure comparing canonical SPP1^{+}/TREM2^{+} features (SPP1, TREM2) with lipid-associated genes (FABP1, APOA1, APOC2, APOB) provide a more objective basis for distinguishing the populations.

Author's response: We thank the reviewer for the positive feedback on our additional analyses. During the previous round of revision, we followed the suggestion from reviewer and conducted cross-dataset comparison using HCC αPD-1 immunotherapy scRNA-seq dataset from Liu et al. The harmony-based batch correction and side-by-side visualization of predefined SPP1^{+} macrophages with the LAM cluster have strengthened the robustness of our conclusions, while the updated comparative analysis of canonical SPP1^{+}/TREM2^{+} markers and lipid-associated genes provide a more objective framework for distinguishing these macrophage populations.
Therefore, we have incorporated the results of integrated analyses above into the Supplementary Fig. 6f-g to support the existence and characterization of the LAM population in this revision.
These additions further improve transparency and allow readers to directly comprehend the features of LAM with lipid-metabolism conditions that demonstrated in our research, especially similarities and distinctions with SPP1^{+}TAMs that presented in previous research.

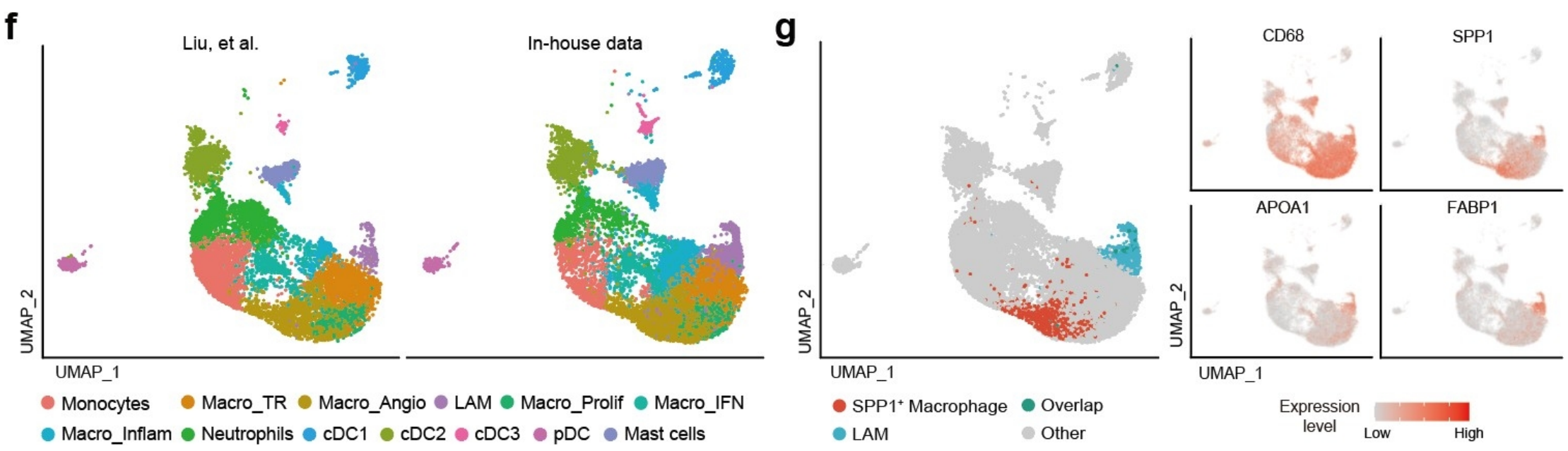

Cross-dataset comparison and integrated analysis of our and external HCC immunotherapy scRNA-seq data, from Supplementary Fig. 6f-g. Fig. 6f Integrated single-cell atlas of external HCC immunotherapy scRNA-seq dataset (Mendeley data, skrx2fz79n) and in-house dataset. Spatial distributions of the SPP1^{+} macrophages pre-defined in research of Liu, et al. and the LAMs identified in our study on UMAP embedding. The results showed very few overlaps between these two subsets, indicating that SPP1^{+} macrophages and LAMs represent distinct macrophage subtypes. **Fig. 6g** Featureplots showing expression levels of *CD68*, *SPP1* and lipid associated genes (*FABP1*, *APOA1*), indicating the shared features and especially distinctions of two subtypes.

Related to point 6:

I thank the authors for adopting the established terminology "lipid-associated macrophages (LAMs)" throughout the revised manuscript. This improves clarity and aligns the study with current conventions in the field. The authors also clarify the relationship between their identified LAM population and previously described SPP1$^+$ LAM subsets. The explanation that their LAMs share certain canonical tissue-remodeling markers (e.g., SPP1) but are additionally characterized by prominent lipid metabolic features (FABP1, APOA1, APOC1, etc.) is helpful and consistent with the expanded comparative analyses provided in response to Comment 5. That said, I recommend slightly refining the language in the manuscript to avoid over-fragmenting macrophage nomenclature. Given the partial overlap in marker expression and functional programs, it may be more accurate to describe the identified LAMs as a metabolically enriched LAM state within the broader lipid-associated macrophage spectrum, rather than implying a completely separate macrophage category. Careful framing will help ensure that the study is perceived as extending and functionally refining existing LAM concepts rather than redefining terminology. Overall, the nomenclature issue has been appropriately addressed. Minor clarification in the Discussion to position this LAM subset within the evolving macrophage-state landscape would further enhance conceptual coherence.

Author's response: We sincerely thank the reviewer for this thoughtful and constructive recommendation. We fully agree that careful terminology is important to avoid unnecessary fragmentation of macrophage nomenclature and to appropriately position our findings as a metabolically enriched LAM state within the evolving research area on macrophages.

In the revised manuscript, we have refined the language in the section of Results to describe the identified population as a metabolically enriched LAM state within the broader lipid-associated macrophage spectrum, rather than implying a completely distinct macrophage category (line 328-336). We now emphasize that this subset shares lipid-associated/tissue-remodeling features, including *SPP1* expression, while exhibiting additional enrichment of lipid metabolic programs (e.g., *FABP1*, *APOA1*, *APOC1*) that are particularly associated with immunotherapy resistance in our study. Moreover, in the integrated analysis of Supplementary Fig. 6f-g, the pre-defined SPP1$^+$ macrophage and LAM showed very few overlaps on UMAP embedding, indicating that SPP1$^+$ macrophages and our metabolic LAM subset represent distinct macrophage subtypes.

We have also added clarifying statements in the section of Discussion to explicitly position this metabolically enriched LAM state within the broader macrophage-state landscape, highlighting that our findings extend and functionally refine existing LAM concepts rather than redefining macrophage taxonomy (line 712-715).

Result

Collectively, our analysis of myeloid cells—especially macrophages—in our HCC immunotherapy cohort revealed the existence of a lipid-associated macrophage subset with metabolically enriched state that showed up-regulated expression of lipid-metabolic genes (such as FABP1 and APOA1), average SPP1 expression and lower TREM2 expression, which is different from traditional SPP1$^+$ or TREM2$^+$ macrophages.

Discussion

In summary, our study combined a valuable αPD-1 HCC immunotherapy cohort with single- cell transcriptomic profiling and multi- omics analyses to reveal critical immunomodulatory roles and mechanisms, particularly the immunosuppressive role of a metabolically enriched LAM state and corresponding intervention strategies.

Related to point 7:

I thank the authors for the detailed clarification regarding the use of Monocle2 and Monocle3. The explanation that both tools were applied to the same cell populations and used as complementary validation frameworks—rather than to generate independent biological claims—is helpful and resolves the initial concern about potential methodological inconsistency. The addition of the new figure directly comparing trajectory inferences from Monocle2 and Monocle3 substantially strengthens confidence in the robustness of the inferred state transitions. The rationale for prioritizing Monocle2 for primary visualization and using Monocle3 as algorithmic confirmation is reasonable. Demonstrating concordant lineage ordering across DDRTree- and graph-based approaches meaningfully increases methodological confidence. Regarding RNA velocity, the authors provide a reasonable explanation for not applying it, particularly considering potential variability in RNA integrity from post-treatment surgical specimens. This justification is acceptable. That said, it would be helpful if the manuscript explicitly states that trajectory directionality is inferred based on pseudotime structure rather than independently validated by splicing kinetics, to avoid overinterpretation of directional claims.

Author's response: We sincerely thank the reviewer for the careful evaluation and helpful suggestion regarding the trajectory of cell development. In this research, we conducted the pseudotime trajectory analyses for T cells and myeloid cells by using Monocle2 and Monocle3 approaches concurrently, thus strengthening the robustness of the inferred state transitions.

As recommended, we have now explicitly clarified in the part of Results that lineage directionality is inferred based on pseudotime structure and topological ordering within the trajectory framework (illustrated in Fig. 3e, Fig. 4d), rather than independently validated by splicing kinetics – based RNA velocity analysis (line 248-253, 304-306). We have also revised the relevant section in the Method to avoid any potential imprecision of claims (line 873-877).

Method

We used Monocle2 and Monocle3 to infer the cell lineage trajectory of T cells and myeloid cells based on the top 700 differential signature genes with q value < 0.001, calculated by differentialGeneTest function. The differentiation trajectory along with directionality was inferred with the pseudotime structure using default parameters of Monocle, after the procedure of dimension reduction and cell ordering.

Related to point 8:

I thank the authors for their thoughtful response and for revising the manuscript to more appropriately frame pySCENIC and CellChat analyses as hypothesis-generating rather than

definitive mechanistic evidence. The revised wording improves interpretive rigor and aligns with the exploratory nature of these computational tools. The addition of NicheNet analysis represents a meaningful strengthening of the study. Integrating ligand–receptor interactions with downstream target gene programs provides a more mechanistically coherent bridge between predicted intercellular signaling and observed transcriptional states. The concordance between CellChat and NicheNet interaction trends further increases confidence in the robustness of the communication patterns described. The inclusion of paired spatial transcriptomics and the murine intervention experiments targeting lipid metabolic pathways (e.g., high-fat diet, lycopene, pan-PPAR agonist) also provides important orthogonal support that moves the study beyond purely computational inference.

Author's response: We thank the reviewer for the insightful suggestion on incorporating NicheNet analysis alongside our original CellChat-based cell–cell communication analysis during previous revision. We agree that this complementary approach provides an additional angle of validation for the predicted ligand–receptor interactions. The integration of NicheNet with CellChat results further supported the robustness of the inferred signaling relationships and strengthened the biological interpretation of the communication patterns observed in our dataset. Importantly, these analyses also helped guide the design of subsequent *in vivo* experiments targeting lipid metabolic pathways. We appreciate the reviewer's constructive suggestion, which has contributed to the improvement of both analytical rigor and translational relevance of the study.

Related to point 9:

I thank the authors for the extensive clarifications and additional experimental data provided in response to this comment. The revised manuscript substantially improves the transparency and functional depth of the validation studies. The clarification that six independent patient-derived HCC organoid (HCO) lines were generated, with a reported take rate of approximately 60%, and that autologous PBMC-derived T cells were used for co-culture, meaningfully strengthens the methodological clarity. The inclusion of patient clinical information in Supplementary Table 2 is also appreciated. The explicit acknowledgment that the HCO patients had not received prior immunotherapy appropriately avoids confounding treatment-related immune alterations. The authors' explanation regarding the use of PBMC-derived T cells based on prior "clonal expansion/revival" frameworks is reasonable. Importantly, they now acknowledge the limitation that the frequency of tumor-reactive T cells within bulk PBMC is likely low. This caveat should remain clearly stated in the manuscript. The organoid co-culture system should be framed as an assay assessing immunomodulatory potential rather than definitive evidence of antigen-specific cytotoxicity. The addition of the murine HCC model with high-fat diet (HFD), lycopene, and pan-PPAR agonist (chiglitazar) interventions significantly strengthens the functional component of the study. The multi-parameter flow cytometric analysis demonstrating alterations in: Effector cytokine production (TNF, IFN-γ) Proliferative capacity (Ki-67) Exhaustion markers (PD-1, TIGIT) Regulatory T cell abundance provides a much more comprehensive assessment of T cell dysfunction associated with LAM enrichment. This in vivo validation meaningfully supports the link between lipid metabolic programs and

impaired antitumor immunity. The correction of the PMA polarization description is also appropriate. The clarified two-step protocol (PMA-induced differentiation followed by IL-4/IL-13 polarization) and supporting CD206 expression data adequately address the earlier methodological inaccuracy. That said, several interpretive boundaries should remain clearly defined: While autologous pairing excludes allo-reactivity, antigen specificity was not directly demonstrated. This limitation should be explicitly acknowledged. The murine HFD model modulates systemic lipid metabolism rather than macrophage-specific pathways. Therefore, while the data strongly support a lipid-driven immunosuppressive axis, they do not fully isolate LAM-intrinsic mechanisms. Although PPAR modulation was incorporated, direct perturbation of key molecules highlighted in the single-cell analyses (e.g., SPP1 or TREM2) was not performed. The Discussion should carefully distinguish between pathway-level validation and target-specific mechanistic proof. Overall, the functional validation arm has been substantially strengthened compared to the initial submission and now provides meaningful biological support for the central hypothesis. With careful framing of the remaining limitations, this section is sufficiently robust.

Author's response: We sincerely thank the reviewer for the thoughtful evaluation of the functional validation experiments and for the constructive guidance on elaborating the certain boundaries between findings and limitations remaining in our study.

As the reviewer suggested, we have further refined the section of Discussion systematically to clearly delineate the conclusions and limitations of our functional experiments:

First, we now explicitly state that although the HCC organoid–PBMC co-culture system excludes allo-reactivity through autologous pairing, the antigen specificity of tumor-reactive T cells with limited frequency was not directly demonstrated by specific experiments. Accordingly, this assay is framed as an assessment of immunomodulatory potential rather than definitive evidence of antigen-specific cytotoxicity (line 699-703).

Second, we have clarified that the high-fat diet (HFD) model modulates systemic lipid metabolism and therefore does not exclusively target the macrophage-intrinsic LAM mechanisms. The *in vivo* findings are interpreted as supporting a lipid-driven immunosuppressive axis at the tumor microenvironment level rather than as proof of cell-autonomous LAM regulation (line 703-707).

Third, we have revised the Discussion to distinguish between pathway-level modulation and target-specific mechanistic validation. While pharmacologic PPAR modulation supports the functional relevance of lipid metabolic programs, direct perturbation of specific LAM-associated molecules (e.g., SPP1 or TREM2) was not performed and will require further investigation in the future (line 707-711).

We believe these clarifications suggested by the reviewer appropriately helped us to define the interpretive boundary of the functional validation, improved the preciseness of central conclusion in our study, that metabolically enriched LAMs impaired antitumor immunity in HCC patients.

Discussion

Then, the comprehensive immune functions and processes exerted by T cells within patient-derived in vitro models were not fully studied. Antigen specificity of tumor-reactive T cells was not directly demonstrated, as the combination of scRNA-seq, scTCR-seq with co-culture models could be a

suitable strategy to explore this issue. Finally, the high-fat diet model modulates systemic lipid metabolism and does not exclusively target macrophage-intrinsic pathways. Thus, although the in vivo data support a lipid-driven immunosuppressive axis, they do not fully isolate cell-autonomous mechanisms of LAMs. Moreover, while pharmacologic PPAR modulation functionally implicates lipid metabolic programs, direct genetic perturbation of specific LAM-associated molecules, such as SPP1 or TREM2 was not performed and remains an important direction for future study.

Related to minor points:
The authors have comprehensively addressed all minor comments raised in the previous review round.

Comments on Revised Figures and Corresponding Text
- Related to revised Fig 1 and corresponding text
In the abstract, revised Fig 1, and corresponding text, the authors should explicitly state that the 60 public-domain cases are all treatment-naïve, whereas the authors' own cohort consists of patients treated with neoadjuvant immunotherapy. This distinction is essential for appropriate interpretation, as baseline (treatment-naïve) TIME features are not directly equivalent to post-treatment specimens. Explicitly highlighting this difference will improve transparency and contextual accuracy.

Author's response: We thank the reviewer for this important comment regarding the distinction between the public HCC datasets and our neoadjuvant immunotherapy HCC cohort.
We agree that treatment background of these datasets should be clearly specified to avoid misinterpretation. In the sections of Abstract and Fig. 1 with corresponding contents in our revised manuscript, we have clarified that the five publicly available scRNA-seq datasets used for atlas construction include patients with heterogeneous or incompletely annotated treatment histories. Specifically, all HCC patients from these public scRNA-seq datasets were not treated with αPD-1 blockade and most were treatment-naïve, while the scRNA-seq cohort of our project exclusively consists of post-treatment tumor specimens derived from 14 HCC patients who received neoadjuvant αPD-1 immunotherapy.
Importantly, this integration of HCC single-cell altals based on diverse high-throughput datasets with various backgrounds contributes to the robustness and universality, thus benefitting the precise and unbiased identification of major cell types and subtypes within the HCC tumor microenvironment. In addition, all further analyses of clinical responses related of HCC immunotherapy were restricted to our neoadjuvant immunotherapy cohort. We have revised the Abstract (line 32-35), Results (line 120-124) and corresponding legends in Fig. 1 (line 1262-1265) to explicitly state this distinction in order to improve transparency and contextual accuracy.

- Related to revised Fig 2 and corresponding text
Given that the authors have now generated paired in-house spatial transcriptomics data, these data should be prioritized in the main figure (e.g., Fig 2i–j), while the public-domain spatial dataset could be moved to the Supplementary Information. The in-house paired data provide stronger internal validation and are more directly aligned with the study cohort, and therefore should take precedence in the main narrative.

Author's response: We thank the reviewer for this helpful suggestion. We agree that the in-house spatial transcriptomic (ST) data, which are directly paired with our single-cell cohort, provide stronger internal validation of the findings. In the revised manuscript, we have therefore prioritized the in-house ST data in Fig. 2i-j to better align the spatial analyses with the study cohort. Meanwhile, the previous analyses results of public scRNA-seq (GSE206325) and ST dataset (skrx2fz79n) regarding the same issue have been moved to Supplementary Fig. 2f-g as independent supporting evidences, respectively. This adjustment improves the logical flow of the manuscript and highlights the spatial validation derived from our paired in-house patient samples.

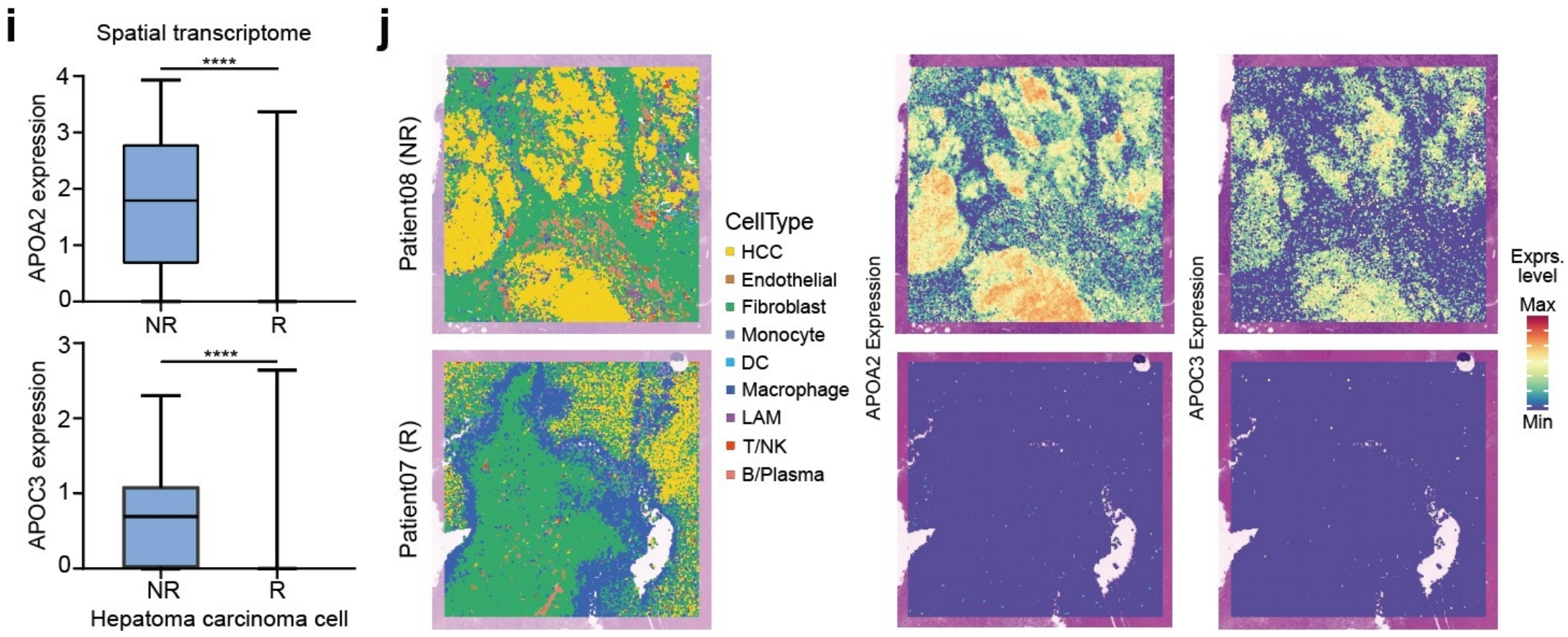

Expression level and spatial distribution of lipid-metabolism features in HCC cells from our cohort (Fig. 2i-j). Fig. 2i Expression levels of *APOA2* and *APOC3* among HCC cells from six patients with different clinical outcomes in paired ST data. **Fig. 2j** Spatial featureplots of ST data in our HCC immunotherapy cohort, showing distinct expression level of *APOA2* and *APOC3* among typical responder (patient07) and non-responder (patient08) of HCC patients.

- Related to revised Fig 5 and corresponding text

In Fig 5e, mIHC staining is presented; however, it is difficult to clearly visualize APOA1^{+} CD68^{+} macrophages in tissues where APOA1^{+} tumor cells are also abundant. It would strengthen the figure if the authors: a) Select one or two representative regions and provide zoomed-in/enlarged panels highlighting APOA1^{+} CD68^{+} double-positive cells. B) Provide single-channel images in the Supplementary Information to allow clearer assessment of co-localization. A similar improvement would be beneficial for the mIHC images presented in Fig 6c.

Author's response: We thank the reviewer for this helpful suggestion. We agree that the presence of APOA1^{+} tumor cells may make it difficult to clearly visualize APOA1^{+} CD68^{+} macrophages in the multiplex immunohistochemistry (mIHC) images.

To improve the clarity of signal co-localization, we have added enlarged views of two representative regions in Fig. 5e for both responder and non-responder patients, thus highlighting the enrichment of APOA1^{+} CD68^{+} LAMs in non-responder. In addition, the splited single-channel images of representative TIME features in responder and non-responder were provided in the Supplementary Fig. 5g to improve the visualization and assessment of signal overlap. Meanwhile, we have applied the same processing to the mIHC images in Fig. 6c by including representative

zoomed-in panels along with corresponding single-channel images in the Supplementary Fig.6h.

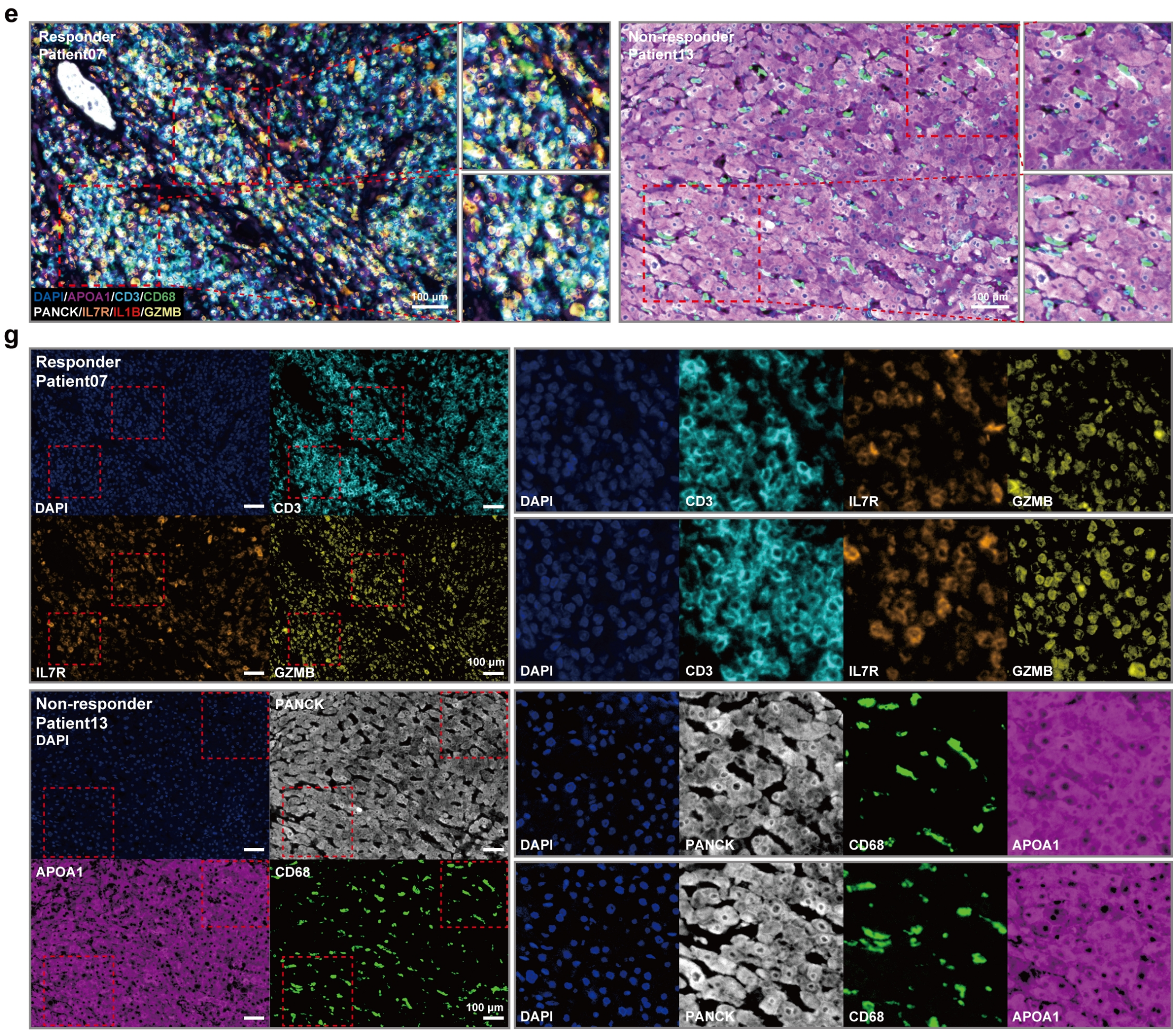

Multiplex immunohistochemistry (mIHC) results of typical responder and non-responder, from Fig. 5e and Supplementary Fig. 5g. Fig. 5e Merged images of mIHC staining from the responder (patient07) and non-responder (patient13) patients. **Supplementary Fig. 5g** Splited single-channel images of representative TIME features, related to Fig. 5e. Scale bars, 100 μm.

- Related to revised Fig 6 and corresponding text

In Fig 6b, spatial data from patients 3 and 11 are shown as representatives, but their responder/non-responder status is not labeled. In contrast, Fig 6c uses different representative cases (patients 13 and 10). For consistency and clarity, the same representative patients should ideally be used across the main panels. All remaining patient images can be presented in the Supplementary Information. In addition, Fig 6k would be more logically placed immediately after LAM characterization (i.e., after Fig 6f and before the co-culture experiments), to improve narrative flow.

Author's response: We thank the reviewer for this helpful suggestion. We have adjusted the representative mIHC panels in Fig. 6c as paired results with ST data of patient03 and patient11 in Fig. 6b, with labeled responses to improve clarity and consistency. In addition, we have provided the enlarged views and single-channel images in Supplementary Fig. 6h related to Fig. 6c, similar to the previous question.

Regarding the placement of Fig. 6k, we appreciate the reviewer's suggestion to move this panel closer to the LAM characterization section. Therefore, we additionally included a comparison of lipid accumulation imaging between LAM and M2-macrophages in the Supplementary Fig. 6i to further support the metabolic characterization of LAM. Meanwhile, the original Fig. 6k involved with the effect of lycopene treatment in alleviating lipid accumulation, which is part of the downstream functional exploration, we therefore tend to retain its position for a logical consecution from mechanistic insight to functional modulation.

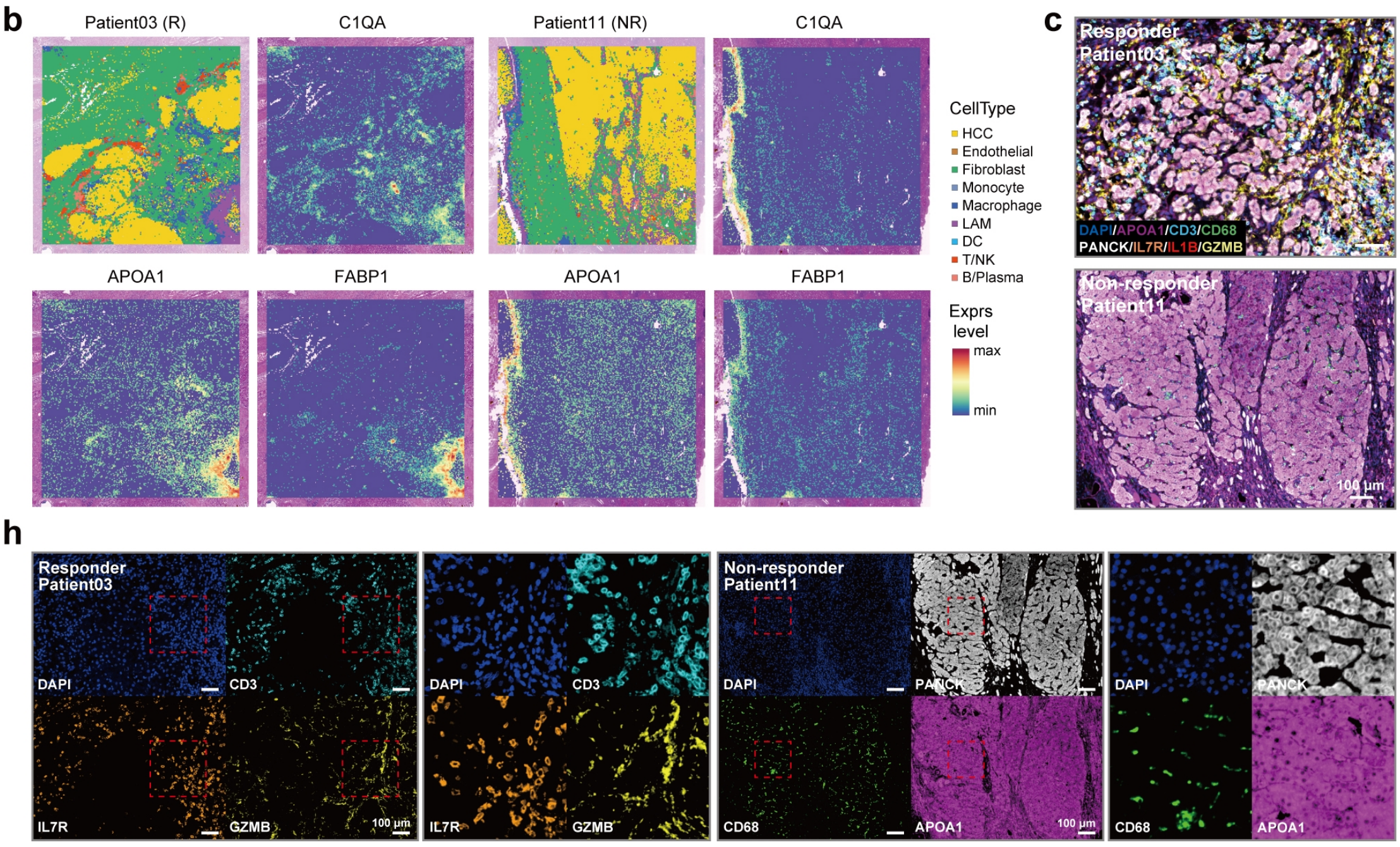

Spatial transcriptomic (ST) and multiplex immunohistochemistry (mIHC) results of typical responder and non-responder, from Fig. 6b-c and Supplementary Fig. 6h. Fig. 6b-c Paired ST and mIHC results from responder (patient03) and non-responder (patient11). **Supplementary Fig. 6h** Enlarged view and splited single-channel images related to Fig. 6c. Scale bars, 100 μm.

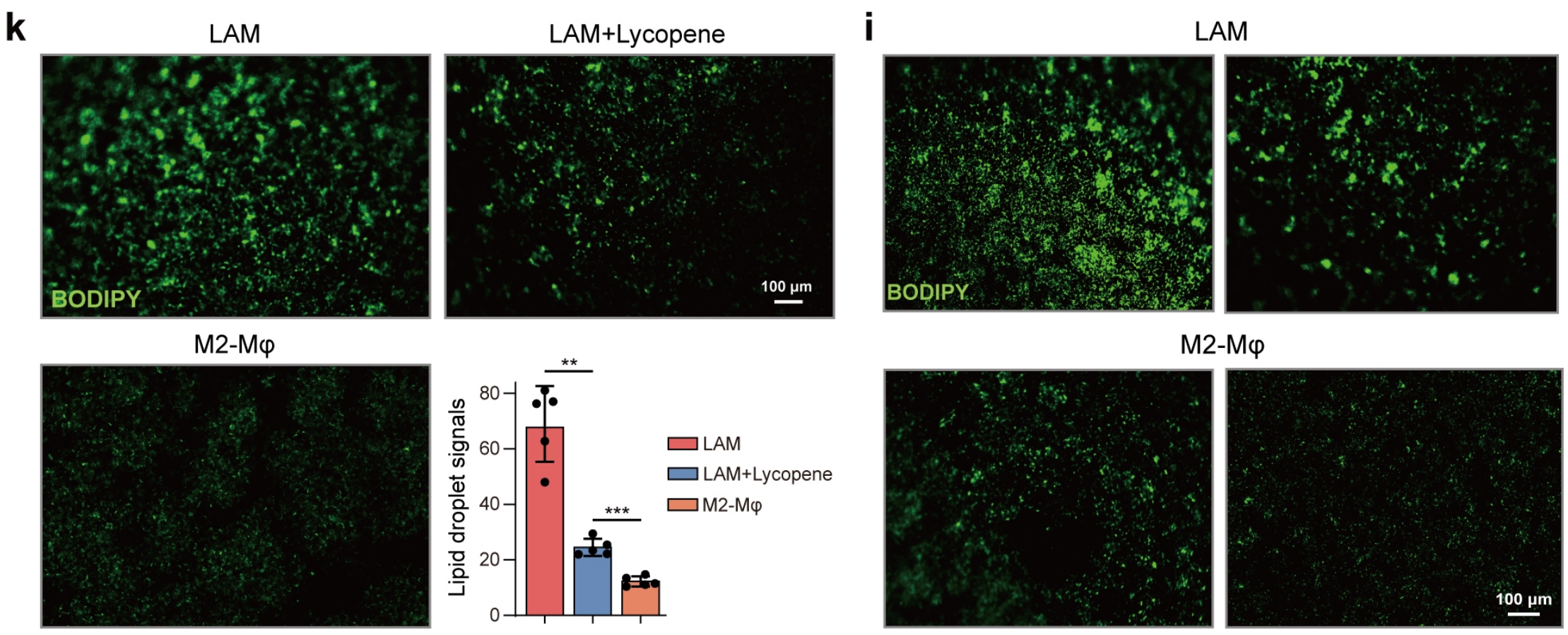

Lipid droplet staining of LAM and M2-macrophages (M2-Mφ) from Fig. 6k and Supplementary Fig. 6i. Fig. 6k Fluorescence imaging and quantification of lipid droplets stained with BODIPY in LAM, M2-Mφ, and LAM with lycopene. **Supplementary Fig. 6i** Fluorescence imaging of lipid droplets in LAM and M2-Mφ stained with BODIPY. Scale bars, 100 μm.

- Related to revised Fig 7 and corresponding text

The inclusion of in vivo experiments is commendable and substantially strengthens the manuscript. However, several clarifications and revisions are needed:

a. Fig 7a (schematic): The liver cartoon appears to depict human anatomy rather than mouse liver. This should be corrected for anatomical accuracy.

Author’s response: We thank the reviewer for pointing out this issue. We agree that the original schematic did not accurately reflect murine liver anatomy. In the revised version, Fig. 7a has been updated to depict anatomically correct mouse liver morphology to ensure accuracy and clarity. We appreciate the reviewer’s attention to this detail.

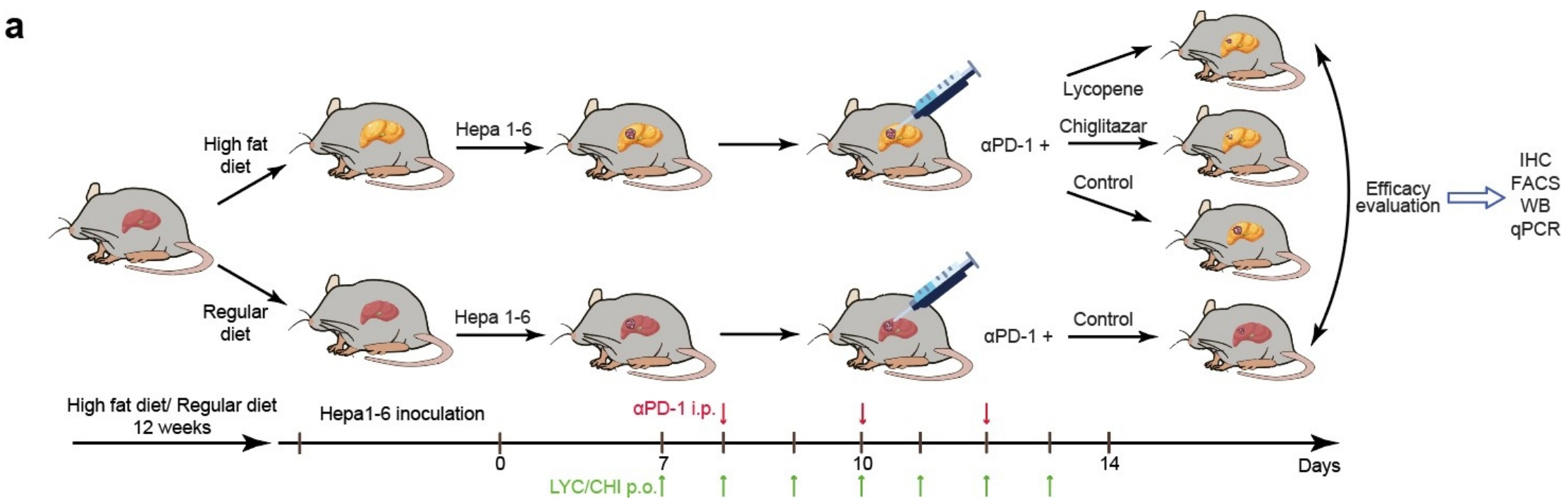

Diagram of the *in vivo* mice model experimental design, from Fig. 7a.

b. Fig 7b–c (bioluminescence and tumor weight): Fig 7b shows representative bioluminescence images. If available, quantitative bioluminescence signal measurements should be presented alongside tumor weight data in Fig 7c, with statistical comparisons between groups.

Author’s response: We thank the reviewer for this helpful suggestion. In the revised manuscript, we have added quantitative analysis of bioluminescence signal intensity alongside tumor weight measurements. Total photon flux values were quantified for each mouse and are now presented in the updated Fig. 7c, with statistical comparisons performed between groups. These additions provide objective quantitative support for images in Fig. 7b and further strengthen the robustness of *in vivo* findings.

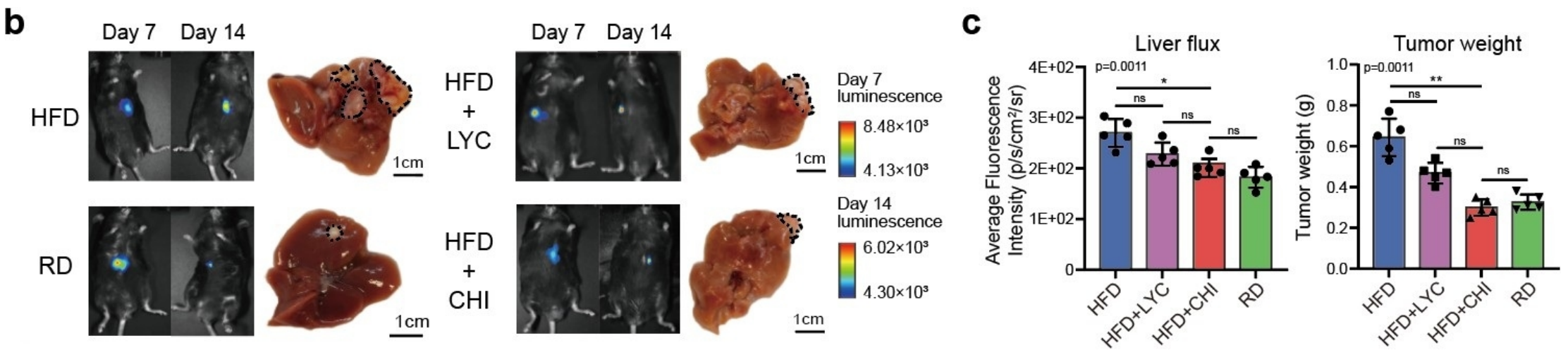

***In vivo* tumor status assessment by bioluminescence imaging and tumor weight measurement of HCC mouse model, from Fig. 7b-c. Fig. 7b** Representative *in vivo* bioluminescence images and tumor image. **Fig. 7c** Average fluorescence intensity in day 14 of HCC mice (N=5) and tumor weight of HCC mice at endpoints (N=5).

c. Fig 7d (Oil Red O staining): The methodology for Oil Red O staining quantification is not described. The authors should clarify:
How staining was performed,
How the positive area was quantified,
Whether quantification was automated or manual, and
How normalization was conducted.

Author's response: We thank the reviewer for this helpful comment regarding the Oil Red O staining methodology. We agree that additional methodological details are necessary to ensure transparency and reproducibility.

In the revised manuscript, we have expanded the Methods section to provide a detailed description of the Oil Red O staining procedure in Fig. 7d, including fixation, staining conditions, and imaging parameters. We have also clarified how lipid accumulation was quantified. Briefly, stained sections were imaged under identical exposure settings, and the positive staining area was quantified using ImageJ software. The percentage of Oil Red O–positive area relative to total tissue area was calculated for each section. Quantification was performed using a standardized thresholding approach applied uniformly across all samples to ensure comparability.

These additions clarify the staining protocol, quantification strategy, and normalization method (line 1060-1075), thereby strengthening the methodological rigor in our study.

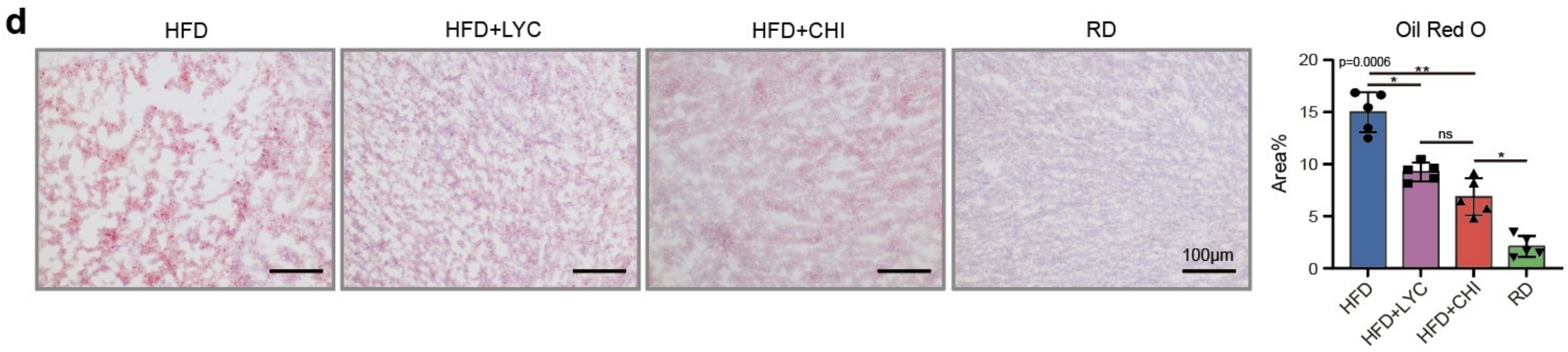

Representative Oil Red O staining of tumor regions and quantification showing lipid accumulation degree of each group (N=5), from Fig. 7d.

Method

Fresh liver tumor tissues were embedded in optimal cutting temperature (OCT) compound and sectioned into 8-μm cryosections. Sections were fixed in 4% paraformaldehyde for 15 minutes at room temperature, rinsed with distilled water, and incubated in 60% isopropanol for 5 minutes. Slides were then stained with freshly prepared Oil Red O working solution (0.5% Oil Red O in isopropanol, diluted 3:2 with distilled water and filtered before use) for 15 minutes at room temperature. After washing with 60% isopropanol and distilled water, nuclei were counterstained with hematoxylin for 1 minute. Sections were mounted in aqueous mounting medium and imaged immediately. Brightfield images were acquired under identical microscope settings for all samples. For quantitative analysis, at least three non-overlapping random fields per section were captured, and three sections per sample were analyzed. Oil Red O–positive areas were quantified using ImageJ software (NIH). Images were converted to RGB stacks, and a standardized color threshold was applied uniformly across all samples to identify lipid-positive regions. The percentage of Oil Red O–positive area relative to the total tissue area within each field was calculated. The mean percentage of positive area per mouse was used for statistical analysis.

d. Fig 7e (LAM gating and denominator clarification): The denominator for LAM percentage needs clarification.

Specifically: Is LAM frequency calculated as % of $CD45^+$ $F4/80^+$ $CD11b^+$ $C1Q^+$ macrophages? Or as % of $CD45^+$ cells?

If the denominator is macrophage-gated, then the statistic reflects only co-expression ($FABP1^+$ $APOA1^+$ within macrophages), not overall LAM abundance. It would be equally important to show absolute numbers or percentage relative to $CD45^+$ cells to determine whether total LAM abundance changes across conditions.

Author's response: We thank the reviewer for pointing out this important issue regarding the definition of LAM frequency.

We have now included both representations of LAM proportion in Fig. 7e to ensure the robustness of our finding. Specifically, LAM frequency is primarily calculated as the percentage of total macrophages ($CD45^+$ $F4/80^+$ $CD11b^+$ $C1Q^+$). In addition, we have also provided the proportion of LAMs relative to total $CD45^+$ immune cells as a complementary metric.

Both calculation approaches are now clearly labeled in the Fig. 7e and legends (line 1414-1417), with explicit indication of the denominator used. As a result, the overall trend remains consistent between both representations.

e

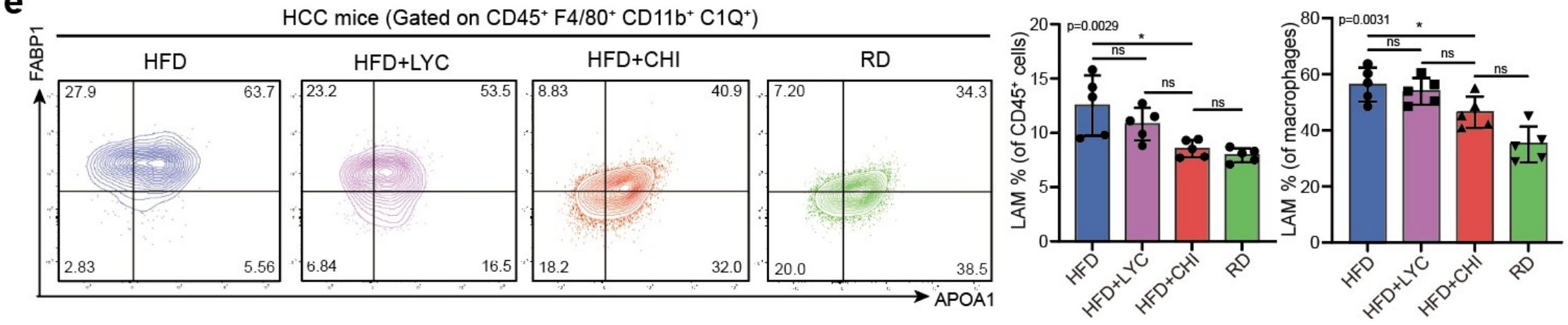

The percentage of lipid-associated macrophage (LAM) in $CD45^+$ cells and $CD45^+$ $F4/80^+$ $CD11b^+$ $C1Q^+$ macrophages, analyzed by flow cytometry (N=5), from Fig. 7e.

Furthermore, the gating suggests a relatively high proportion of $CD11b^-$ $F4/80^+$ cells (Kupffer-like cells) compared to $CD11b^+$ $F4/80^+$ monocyte-derived macrophages. This is unexpected in many HCC models, where monocyte-derived macrophages typically predominate. Therefore, the manuscript must clearly describe how mouse tissues were processed: Were tumor nodules microdissected prior to digestion? Or was whole liver homogenized for single-cell suspension? If whole liver was processed, interpretation of tumor-infiltrating macrophages may be confounded by resident Kupffer cells. Currently, there is insufficient description of mouse tissue processing and single-cell preparation in the revised Methods.

Author's response: We thank the reviewer for thoughtful observation of macrophage composition, from the C57BL/6 mouse model inoculated orthotopically with Hepa 1-6 cells in our study.

First of all, only the microdissected specific tumor nodules from liver tissue were processed for preparation of cell suspension, whole liver tissue was not homogenized. Therefore, the observed macrophage components reflect tumor-infiltrating populations rather than hepatic parenchyma. We have now explicitly described the microdissection procedure and tumor-specific processing steps of mouse tissue in the revised Methods section (line 981-986) to prevent ambiguity.

Meanwhile, accumulating evidence indicates that resident-like macrophages can persist within the tumor microenvironment and undergo tumor-associated reprogramming rather than being fully replaced by recruited populations (Zheng, et al.). For instance, Ye, et al. found that the dominating TAMs in orthotopic HCC mice manifested properties of Kupffer cells (KCs), thus suggesting TAMs are contained with different sources. In addition, KC–associated transcriptional programs and signaling pathways have been implicated in HCC progression and immune modulation, supporting the concept that resident-derived macrophages may functionally contribute to tumor-associated macrophages (Shirabe, et al.). Therefore, the presence of a substantial $CD11b^{low}$ $F4/80^{high}$ macrophage fraction in our model likely reflects heterogeneous macrophage state within the tumor microenvironment rather than contamination from non-tumorous liver tissue, especially for our orthotopic HCC mice model as Ye, et al. reported. Moreover, CD11b expression can exhibit a continuum rather than a strict binary distinction, and $CD11b^{low}$ $F4/80^{high}$ populations in flow cytometry may include macrophages that have adopted tumor-associated phenotypes. In summary, the enrichment of $CD11b^{-}$ $F4/80^{+}$ cells may represent KCs of a significant proportion that initially located in orthotopic tumor nodule. These KCs collaborate with macrophages derived from other sources and composed the total TAMs in HCC tumor immune microenvironment.

[editorial note: third party material redacted]

Major sources of TAMs in HCC: yolk sac, fetal liver, and bone marrow, as well as three direct sources: blood/bone marrow-derived monocytes, KCs, and MDSCs. (Zheng, et al.)

Reference:

Zheng H, Peng X, Yang S, Li X, Huang M, Wei S, Zhang S, He G, Liu J, Fan Q, Yang L, Li H. Targeting tumor-associated macrophages in hepatocellular carcinoma: biology, strategy, and immunotherapy. Cell Death Discov. 2023 Feb 15;9(1):65.

Ye YC, Zhao JL, Lu YT, Gao CC, Yang Y, Liang SQ, Lu YY, Wang L, Yue SQ, Dou KF, Qin HY, Han H. NOTCH Signaling via WNT Regulates the Proliferation of Alternative, CCR2-Independent Tumor-Associated Macrophages in Hepatocellular Carcinoma. Cancer Res. 2019 Aug 15;79(16):4160-4172.

Shirabe K, Mano Y, Muto J, Matono R, Motomura T, Toshima T, Takeishi K, Uchiyama H, Yoshizumi T, Taketomi A, Morita M, Tsujitani S, Sakaguchi Y, Maehara Y. Role of tumor-associated macrophages in the progression of hepatocellular carcinoma. Surg Today. 2012 Jan;42(1):1-7.

e. Fig 7f (T cell flow cytometry): The y-axis labels are unclear and should be clarified. Supplementary Fig 7 shows gating strategy but appears to focus primarily on $CD4^+$ $CD3^+$ cells. $CD8^+$ T cells are central to cytotoxic function and should be explicitly analyzed. The manuscript does not clearly state whether "T cells" refer to $CD4^+$, $CD8^+$, or total $CD3^+$ cells. If CD8 staining was not included, $CD4^-$ $CD3^+$ could serve as a surrogate for $CD8^+$ T cells, although direct CD8 staining would be preferable.

Author's response: We thank the reviewer for this important comment regarding T cell subset definition and gating clarity in flow cytometry.

In Fig. 7f, the y-axis represented the percentage of TNF, IFNG, KI67, TIGIT, PD-1 expression cells of total $CD3^+$ T cells, while the proportion of Regulatory T cells (Treg cells) with FOXP3 expression were calculated at the level of $CD4^+$ T cells.

Functional markers were assessed at the level of total $CD3^+$ T cells, as both $CD4^+$ and $CD8^+$ T cell subsets are capable of producing these effector cytokines (TNF, IFNG), or expressing proliferation (KI67) and exhaustion signatures (TIGIT, PD-1). Therefore, our analysis aimed to evaluate overall functional status of T cells rather than subset-specific cytotoxic activity.

CD4 staining was included in the panel, allowing identification of $CD4^+$ T cells. As most Treg cells are CD4 positive, Treg cells frequency was calculated specifically within the $CD4^+$ T cell compartment. CD8 staining was not included in this experimental set. Accordingly, the term "T cells" in the manuscript refers to total $CD3^+$ T cells unless otherwise specified.

We have revised the legends of Fig. 7f (line 1417-1422) to explicitly indicate the analyzed populations and clarify corresponding definitions to avoid ambiguity and we acknowledge that direct CD8 staining would enable more precise subset-specific functional analysis, which could be explored in future studies.

f. Fig 7h-i (Interpretation of chiglitazar effects):

Chiglitazar (a pan-PPAR agonist) appears to increase PPAR expression in mouse liver. However, as this is a systemic drug, it likely affects hepatocytes, tumor cells, and immune cells. Since tumor lipid deposition is also reduced, the authors should avoid directly attributing the effect solely to LAM modulation. The conclusions should be phrased more

cautiously as associations unless PPAR expression/activity was specifically measured in LAMs (e.g., via gated FACS analysis or sorted populations). Minor point: gene symbols should be italicized throughout.

Author's response: We thank the reviewer for this important and insightful comment regarding the targeting effect of Chiglitazar.

We agree that chiglitazar, as a systemic pan-PPAR agonist, could exert effects across multiple cellular compartments, including hepatocytes, tumor cells, and immune populations. Therefore, direct attribution of the observed phenotypic changes solely to LAM-specific modulation would be inappropriate without LAM–restricted measurements of PPAR expression or activity.

In the revised manuscript, we have carefully rephrased the relevant sections to avoid inaccuracy (line 548-551). The effects of chiglitazar are now described as supporting a lipid metabolic axis associated with tumor immune responses, rather than as definitive evidence of LAM-specific PPAR regulation. We explicitly acknowledge that PPAR activity was not selectively measured within sorted LAM populations, and the corresponding results likely reflect combined systemic modulation, which also associated with upstream impacts on LAMs.

Importantly, although chiglitazar is a systemic pan-PPAR agonist rather than macrophage-specific intervention, we additionally evaluated its impact on LAM proportions and phenotypes within the HCC tumor microenvironment. Under PPAR agonist treatment combined with αPD-1 therapy, we observed a reduction in LAM-associated features as illustrated in Fig. 7e, suggesting that modulation of lipid metabolic pathways is associated with changes in LAM states under HCC immunotherapy. These findings therefore support a role for lipid metabolic regulation in shaping macrophage-associated immunosuppressive programs, while we have carefully avoided attributing these effects to LAM-intrinsic PPAR signaling. We believe this revised framing more accurately reflects the scope of our experimental evidence.

Regarding the formatting point such as font of gene symbols, we sincerely thank the reviewer for pointing out these issues and for helping us to improve the presentation of our manuscript. In our revised version, we have corrected the gene symbols into italicized consistently throughout our manuscript, unless the name appears as form of protein.

Overall, the authors have made substantial revisions in response to the previous round of comments, including expanded clinical characterization, strengthened cross-dataset benchmarking, clarified macrophage annotation and nomenclature, improved methodological transparency for single-cell and spatial analyses, and added meaningful in vivo functional validation experiments. The major conceptual and technical concerns raised previously have been largely addressed. However, several remaining issues require clarification and refinement before the manuscript can be considered for acceptance. While these points are relatively minor in scope and primarily relate to presentation, methodological transparency, and figure organization, they are important for ensuring clarity, rigor, and interpretability. I therefore recommend minor revision, with the expectation that the authors address the remaining comments comprehensively prior to final consideration.

Reviewers' comments

Reviewer #1 (Remarks to the Author):

The authors have substantially revised and improved the manuscript. Most of the previous concerns have been adequately addressed, particularly comments 1-3, 5, 7-11, and I thank the authors for their detailed and thoughtful responses.

However, I still have some minor concerns regarding comments 4 and 6, which were not fully resolved in the previous round of review. Additional clarification on these points would further enhance the interpretability of data.

Concerning comment 4, authors stated in their response that "We now present a standardized set of pre-specified pairwise comparisons in Fig. 7c-f and Fig. 7i (HFD vs HFD+LYC; HFD vs HFD+CHI; HFD+CHI vs HFD+LYC; RD vs HFD+LYC; RD vs HFD+CHI) to directly address the biological hypotheses of diet-induced immunotherapy resistance and corresponding metabolic interventions. Meanwhile, all of the remaining pairwise comparisons, including non-significant results, are provided systematically in corresponding tables of Source data to ensure transparency while maintaining figure readability. "

If this were fully the case, I would not have further concerns. However, many of the specified comparisons are still missing in the figure. For example, the comparison between HFD+CHI vs HFD+LYC is not shown in Fig. 7i, and comparisons such as RD vs HFD+LYC seem to be absent in many graphs in Fig. 7c-f and Fig. 7i as well. Similarly, RD vs HFD+CHI does not appear to be included in Fig. 7i. These omissions make it difficult to fully interpret the data. In addition, I was unable to locate the referenced "Source Data tables" containing the remaining pairwise comparisons. It would be helpful if the authors could clarify it.

Author's response: We thank the reviewer for carefully re-examining this point and for identifying the remaining issues in the presentation of pairwise comparisons. We apologize for the lack of clarity in the previous revision.

In the revised manuscript, we have now ensured that all pre-specified pairwise comparisons are consistently presented across Fig. 7c-f and Fig. 7i, including HFD+CHI vs HFD+LYC, RD vs HFD+LYC and RD vs HFD+CHI mentioned by reviewer. We also summarized the panels of Fig. 7c-f and Fig. 7i with corresponding datasets and statistic results into a reorganized figure below. In addition, the corresponding numerical results, including datasets with Kruskal-Wallis test statistics and Dunn's multiple comparisons between each two groups, are systematically provided in the Source Data. These Source Data are supplied as a separate Excel file named "Source data.xlsx" and submitted alongside the manuscript according to the requirement of the journal.

We are hoping these revisions ensure consistency between the stated analysis strategy and the presented data, thus improve the transparency and interpretability of the results.

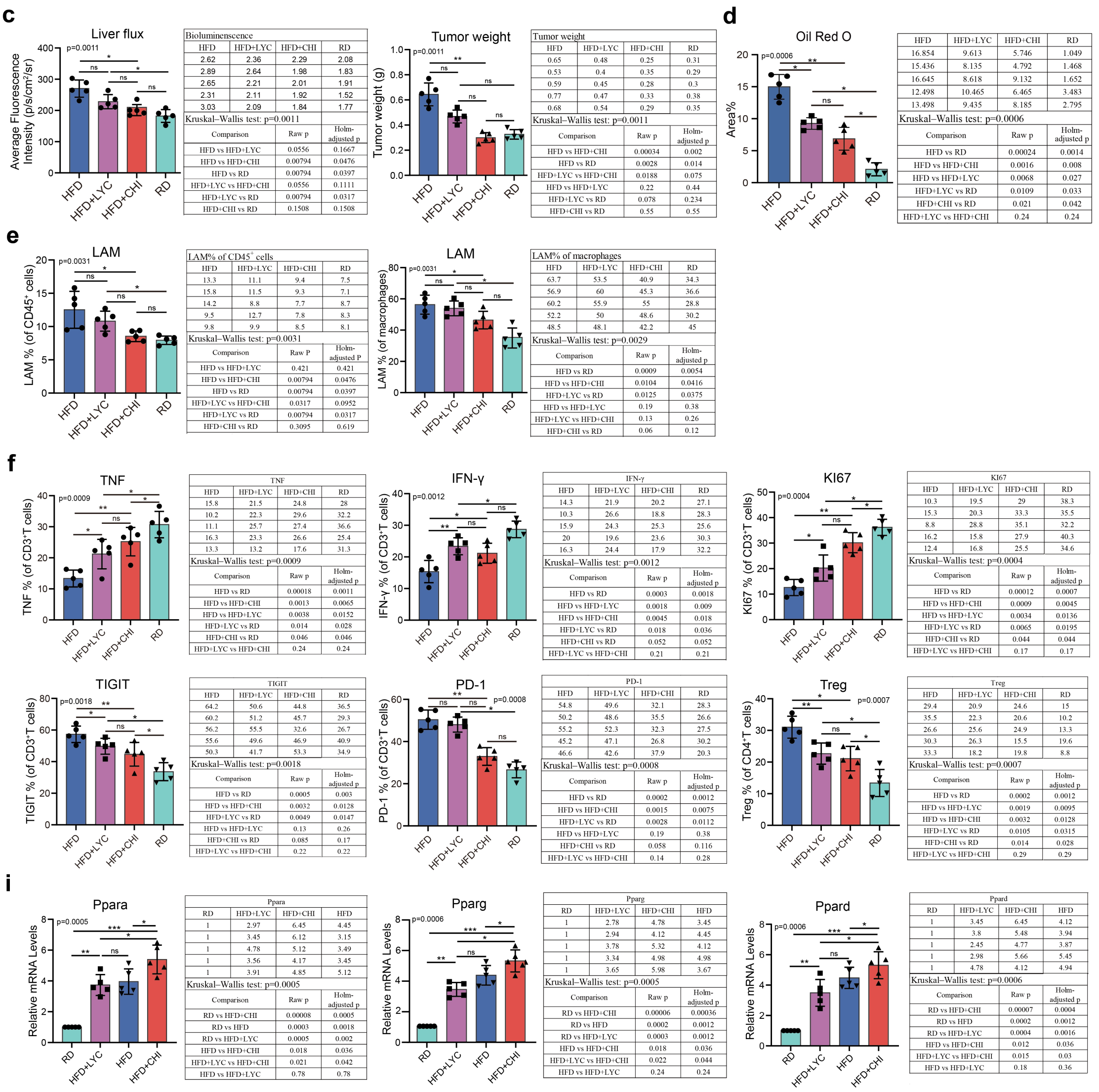

Figures, datasets and statistic results (Kruskal-Wallis test with Dunn's multiple comparisons) of complete pairwise comparisons, from Fig. 7c-f and Fig. 7i. Fig. 7c. *In vivo* bioluminescence imaging signals and tumor weight measurements among experimental groups with corresponding pairwise statistical comparisons. **Fig. 7d.** Quantification of lipid accumulation in tumor tissues by Oil Red O staining, with pairwise statistical comparisons. **Fig. 7e.** Proportion of lipid-associated macrophages (LAMs), presented as percentages of $CD45^{+}$ cells and macrophage populations with corresponding statistical analyses. **Fig. 7f.** Flow cytometry analyses of tumor-infiltrating T cell functional markers, including TNF, IFN-γ, KI67, TIGIT, PD-1, and regulatory T cells with statistical comparisons. **Fig. 7i.** Relative mRNA expression levels of PPAR molecules (*Ppara*, *Pparg*, and *Ppard*) across experimental groups with relative statistical analyses.

Concerning my comment 6. I previously wrote the following comment (6. Supplementary Fig. 7 - The authors have added multiparametric flow cytometric analyses of tumor-infiltrating lymphocytes; however, the presented data appear to contain inaccuracies. It is written that the axis y represents CD45-PE-Cy7 in all graphs, which does not seem to be correct.) This comment was not completely answered. The issue is that the plots of Supplementary Figure 7 d-f are clearly not gated on the same parent population, even though the caption claims they

are. The axis shows CD45-PE-Cy7, but the distribution and density of events are clearly different from panels d to f. If the same population had been used, only the marker on the other axis (for instance, TNF or TIGIT) would change, not the entire population. But here, the mean fluorescence intensity and overall distribution along the CD45 axis are not consistent between d, e and f panels. Therefore, the data seem to be derived from different upstream gating strategies, not from the same population. Please clarify it.

Author's response: We thank the reviewer for carefully revisiting this issue and for raising this important concern, and we apologize for the lack of clarity in our previous response.

In the flow cytometry experiments related to Fig. 7e-f and Supplementary Fig. 7d-g, tumor tissues were dissociated into single-cell suspensions and subsequently divided into five tubes averagely for the staining to evaluate LAM proportion and T cell conditions (including effector, proliferation, exhaustion and regulatory T cell), as it was not feasible to measure all markers simultaneously within a single staining panel, due to the limitations from the detector channels of flow cytometer and spectral overlap of antibodies. This operation is also a common practice during multiple marker measurements (Kargl, et al.). Specifically, for the evaluation of T cells, separate panels were all labeled with viability dye, CD45 and CD3, then used to assess different functional aspects of T cells, including effector function (IFNG, TNF), proliferation (KI67), exhaustion (PD-1, TIGIT), and regulatory T cells (FOXP3).

As a result, for the evaluation of T cells function, although all analyses were performed on T cells derived from the same biological samples and gated using a consistent strategy, the data shown in Fig. 7f and Supplementary Fig. 7d-g were obtained from different staining panels. This leads to differences in event distribution and density along the CD45 axis, reflecting independent acquisitions instead of differences in upstream gating. We also note that displaying CD45 as a common lineage marker on one axis while evaluating different functional markers on the other axis has been used in flow cytometry studies to visualize marker-positive populations across distinct immune panels (Zhang, et al.). However, when separate staining panels are used, differences in fluorescence distribution along the shared CD45 axis can occur because each panel is stained and acquired independently.

To address this point, we have expanded the Methods section to provide a more detailed description of the sample processing (line 985-1003). For further transparency, we have included a figure below in this response letter to illustrate the gating details of Supplementary Fig. 7d-g by using the results from regular diet (RD) group as example, thus allowing direct evaluation of the gating consistency.

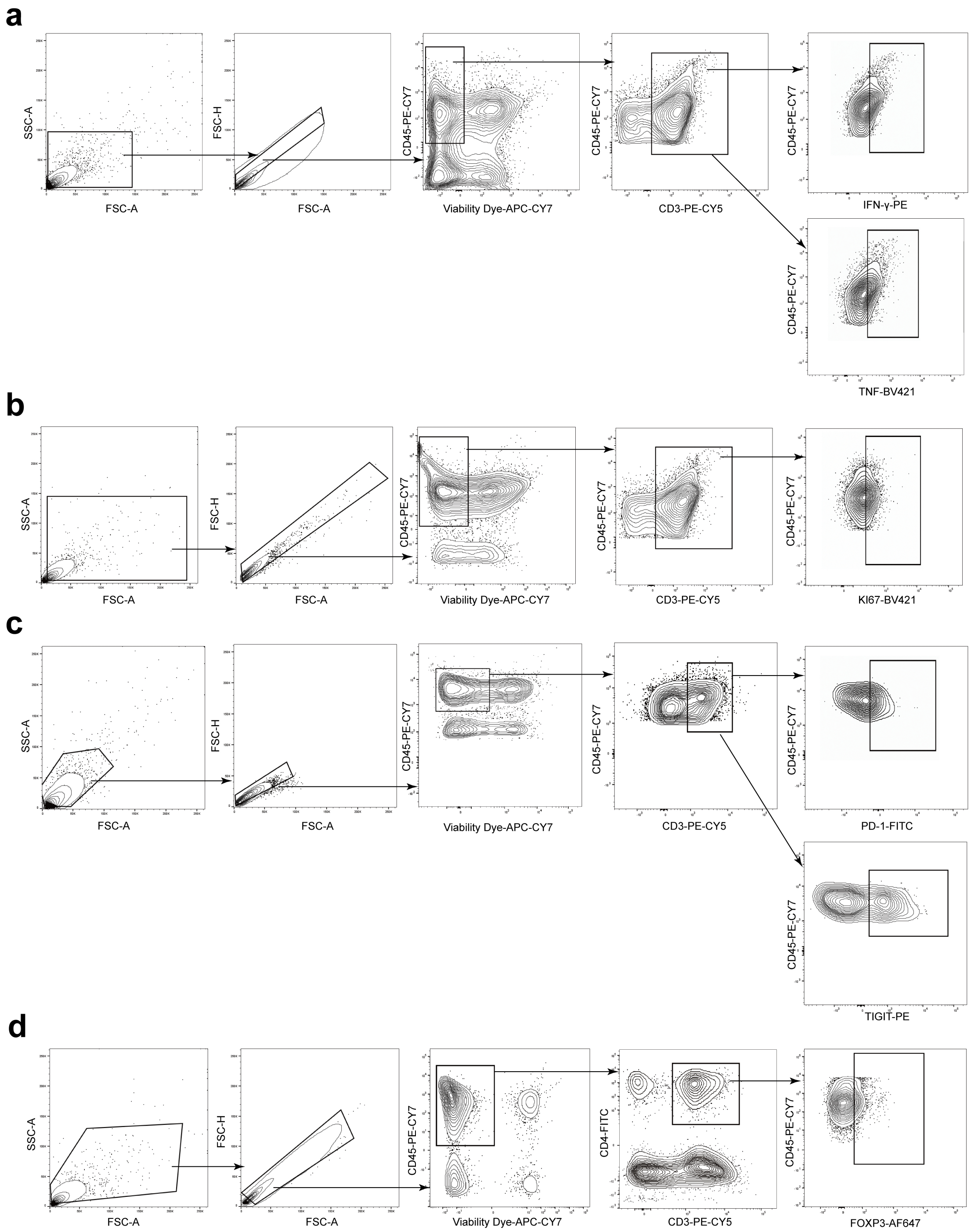

Flow cytometry gating strategy for tumor-infiltrating T cells from regular diet (RD) group as example, related to Supplementary Fig. 7d-g. a. Effector function panel (IFN-γ, TNF) showing effector cytokine expression of T cells, related to Supplementary Fig. 7d. **b.** Proliferation panel (KI67) showing proliferation ability of T cells, related to Supplementary Fig. 7e. **c.** Exhaustion panel (PD-1, TIGIT) showing exhaustion levels of T cells, related to Supplementary Fig. 7f. **d.** Treg panel (FOXP3) showing Treg proportion of $CD4^{+}$ T cells, related to Supplementary Fig. 7g.

Isolation of immune cell and flow cytometry

For PBMC-derived immune cells, peripheral blood mononuclear cells (PBMCs) were isolated from peripheral blood using Lymphoprep™ density gradient separation according to the

manufacturer's instructions. For tumor-derived immune cells, human or mouse tumor nodules were microdissected from liver tissues, minced into small fragments, and enzymatically digested with collagenase IV (1 mg/mL) and DNase I (0.25 mg/mL) at 37°C for 60 min. The resulting cell suspensions were filtered through a 70 µm nylon cell strainer. For flow cytometry analysis of mice tumor samples, the obtained single-cell suspensions were subsequently divided into five staining tubes averagely. Separate panels were used to evaluate LAM proportion and different functional aspects of T cells, including effector function (IFNG, TNF), proliferation (KI67), exhaustion (PD-1, TIGIT), and regulatory T cells (FOXP3).

The cells obtained after digestion were resuspended in a staining buffer and incubated with Fc block (BioLegend) for 10 min. Then surface antibodies were added and stained for 30 min at 4°C in the dark. Cells were fixed by IC Fixtion Buffer (ebioscience) for 30 min at 4°C in the dark, permeabilized by Permeabilization Buffer (Beyotime) for 30 min at 4°C in the dark and then washed twice with diluted 1×PBS. Subsequently, intracellular antibodies were added and stained for 30 min at 4°C in the dark. Finally, cells were analyzed by flow cytometer (BD Biosciences).

References:

Kargl J, Busch SE, Yang GH, *et al*. Neutrophils dominate the immune cell composition in non-small cell lung cancer. *Nature Communications*. 2017 Feb 1;8:14381.

Zhang S, Peng W, Wang H, *et al*. C1q+ tumor-associated macrophages contribute to immunosuppression through fatty acid metabolic reprogramming in malignant pleural effusion. *Journal for ImmunoTherapy of Cancer*. 2023 Aug;11(8):e007441.

Reviewer #3 (Remarks to the Author):

I thank the authors for their detailed and comprehensive responses, as well as for the substantial revisions made to the manuscript. The additional analyses, clarifications, and newly incorporated data have significantly improved the scientific rigor, transparency, and overall clarity of the study.

Below I provide a brief evaluation of the authors' responses to the major points:

Related to Point 1 (Clinical annotation and survival analyses)
The authors have satisfactorily addressed the concerns. The inclusion of expanded clinical characteristics in Supplementary Table 1, clarification of units, and the addition of Kaplan-Meier analyses using both >50% and >70% cutoffs are appropriate and significantly enhance the clinical interpretability of the study. The citation of the 2024 Chinese clinical guidelines is now clearly stated. Overall, this point is adequately resolved. For the newly added Fig. 1e, the authors may consider expanding the figure legend to clearly define the response groups (e.g., non-responders [non-MPR] and responders [MPR + CPR]) to improve clarity. In addition, in the updated Supplementary Table 1, "RECIST" should be specified as "RECIST 1.1."

The authors should briefly discuss the clinical implications of the lack of separation in RFS

and OS observed in the current cohort under Discussion section. In particular, this finding suggests that pathological response, as defined in this study, may not directly translate into measurable survival benefit within the available follow-up period. It would therefore be important for the authors to clearly position the study as primarily aiming to characterise the tumour microenvironment and pathological response to neoadjuvant immunotherapy, rather than to demonstrate treatment efficacy at the clinical outcome level. In this context, the current title may be misleading, as it implies that lipid-associated macrophages (LAMs) impair therapeutic efficacy. Given that no corresponding difference in RFS or OS is observed, such a causal or outcome-linked interpretation is not fully supported by the data. I would therefore recommend revising the title to adopt a more cautious, association-based framing that better reflects the scope and strength of the findings.

Author's response: We thank the reviewer for these helpful suggestions. The legend of Supplementary Fig. 1e has been revised to clearly define the response groups (non-responders [non-MPR] and responders [MPR + CPR]). In addition, "RECIST" has been specified as "RECIST 1.1" in Supplementary Table 1.

In the revised manuscript, we have added a dedicated part in the Discussion to explicitly address the lack of significant separation in RFS and OS observed in the current cohort (line 696-702). We agree that, within the available sample size and follow-up duration, pathological response as defined in this study does not translate into a measurable survival benefit. We now clearly state that the primary aim of this study is to characterize the tumor microenvironment and immune-metabolic features associated with pathological response to neoadjuvant immunotherapy, rather than to demonstrate treatment efficacy at the level of clinical outcomes.

Moreover, we have revised the title of our manuscript to avoid misleading and strength the scope of research findings, according to the reviewer's suggestion. The title have been modified as "Integrated Single-cell Multi-omics Characterization Reveals Lipid-associated Macrophages Mediated Immunosuppression in Neoadjuvant Immunotherapy of Hepatocellular Carcinoma", which is a more cautious framing and avoids implying a direct connection between LAMs and clinical efficacy, thus improve the rigor of statement in our manuscript.

Related to Point 2 (Cross-study comparison and positioning)
The addition of a structured comparison in the Discussion and Supplementary Table 13 meaningfully improves contextualisation relative to prior studies. While some narrative elements could still be slightly streamlined for clarity, the current revision is acceptable. This point is largely resolved.

Related to Point 3 (scRNA-seq methodology and QC)
The authors have adequately clarified digestion duration, batch correction strategy, and provided sample-level QC metrics in Supplementary Table 14. These additions sufficiently address concerns regarding data robustness and reproducibility. This point is resolved.

Related to Point 5-6 (Macrophage annotation and nomenclature)
The additional cross-dataset integration and refined framing of LAMs as a metabolically enriched state within the broader macrophage spectrum are appropriate. The revised

positioning avoids over-fragmentation of nomenclature and is now conceptually sound. These points are satisfactorily addressed.

Related to Point 7 (Pseudotime analysis)
The clarification regarding Monocle2/3 usage and the explicit statement on pseudotime-based directionality are appropriate. The addition of comparative analyses supports robustness. This point is resolved.

Related to Point 8 (Computational inference tools)
The authors have appropriately revised the language to reflect the exploratory nature of pySCENIC and CellChat analyses and have strengthened the study with NicheNet and orthogonal validations.
This point is well addressed.

Related to Point 9 (Functional validation)
The authors have substantially improved the clarity and depth of functional validation, including better description of organoid systems, appropriate acknowledgment of limitations, and strengthened in vivo data. The revised Discussion now appropriately delineates interpretive boundaries.
This point is satisfactorily addressed.

Figure-related and minor points
The authors have generally responded well to the figure-related suggestions, including improved visualisation, clearer gating strategies, and additional methodological details. Most concerns have now been adequately addressed.

Overall comments
Based on the revised manuscript and the clarified in vivo methodology, the data suggest that LAM enrichment may arise, at least in part, as a downstream consequence of lipid metabolism-active tumour states (e.g., APOA2^{+}/APOC3^{+} HCC), as indicated in Fig. 2i-j. In the in vivo experiments, systemic modulation of lipid metabolism (e.g., via chiglitazar or lycopene) may therefore reduce LAM abundance indirectly through effects on tumour metabolic activity, rather than through direct targeting of macrophage-intrinsic pathways.

In this context, the current title—"Integrated Single-cell Multi-omics Characterization Reveals Lipid-associated Macrophages Impairing Neoadjuvant Immunotherapy Efficacy in Hepatocellular Carcinoma"—may overstate both the directionality and causality of the findings. The data presented do not clearly establish that LAMs are primary drivers of therapeutic resistance; rather, they may reflect a tumour-driven metabolic microenvironment associated with non-response. I would therefore recommend that the authors revise the title to adopt a more cautious, association-based framing and to avoid implying a direct causal role of LAMs in impairing treatment efficacy. Such a revision would also better align with the absence of clear survival differences observed in the cohort.

Author's response: We thank the reviewer for these insightful overall comments and for highlighting the importance of carefully distinguishing between intrinsic metabolic mechanism and macrophage-associated pathways.

In line with the reviewer's interpretation, we have revised our manuscript to more cautiously frame the relationship between tumor metabolic activity and LAM enrichment. Specifically, we now emphasize that systemic modulation of lipid metabolism in our *in vivo* models may influence LAM abundance indirectly through effects on tumor metabolic programs, rather than implying a purely macrophage-intrinsic mechanism. These revisions improve the mechanistic rigor and align the interpretation more closely with the results.

Regarding the title, we agree that the original wording may overstate directionality and causality. As noted in our response to Related to Point 1, we have revised the title to adopt a more cautious framing that avoids implying a direct causal role of LAMs in impairing therapeutic efficacy. This revision also align with the absence of significant survival differences observed in our cohort.

Collectively, we appreciate the reviewer's constructive suggestions, which have helped us improve the conceptual clarity and rigor of mechanism in our study.

Final assessment

Overall, the authors have made substantial revisions and have addressed the major conceptual and technical concerns raised in the previous review. The remaining issues are relatively minor and primarily relate to presentation clarity, figure organisation, and language refinement. Provided these points are carefully addressed, the manuscript would be suitable for acceptance. I therefore recommend minor revision, with the expectation that the remaining comments are adequately incorporated.

Author's response: We thank the reviewer for the careful evaluation of our revised manuscript and for the constructive feedback. By adopting these valuable suggestions from all reviewers through previous rounds of revisions, we improved the transparency of clinical information, cross-study comparison and contextualization, methodology application of scRNA-seq, and especially the rigor, interpretability and conceptual framing of our findings between lipid-associated macrophages and neoadjuvant immunotherapy of hepatocellular carcinoma. We have also carefully incorporated the reviewers' suggestions to further improve the clarity, streamline the narrative, enhance figure presentation and methodological descriptions throughout the manuscript.

Once again, we express our appreciation to the reviewers, for their feedback and suggestions significantly improved the quality, rigor, and interpretability of our study.

Reviewers' comments

Reviewer #1 (Remarks to the Author):

All remarks are satisfactorily addressed.

Reviewer #1 (Remarks on code availability):

All remarks are satisfactorily addressed.

Reviewer #3 (Remarks to the Author):

Peng et al. have substantially revised and improved the manuscript in terms of both clarity and scientific rigor. In particular, the authors have addressed the major conceptual and technical concerns raised in the previous rounds of review. The additional analyses, methodological clarifications, expanded clinical annotations, cross-dataset benchmarking, improved spatial validation, and strengthened in vivo functional studies have significantly enhanced the robustness and interpretability of the study.

Regarding the clinical analyses, the authors have now appropriately acknowledged in the Discussion that pathological response in the current cohort does not clearly translate into measurable RFS or OS benefit within the available follow-up period. This clarification helps appropriately position the study as primarily focused on characterization of the tumor immune microenvironment and pathological response associated with neoadjuvant immunotherapy, rather than direct demonstration of treatment efficacy at the survival outcome level.

The revised title is also improved compared with the previous version and appropriately avoids directly claiming impairment of therapeutic efficacy. However, the current wording—"Integrated Single-cell Multi-omics Characterization Reveals Lipid-associated Macrophages Mediated Immunosuppression in Neoadjuvant Immunotherapy of Hepatocellular Carcinoma"—remains grammatically awkward and could be further refined for clarity and readability. In particular, the phrase "Lipid-associated Macrophages Mediated Immunosuppression" would benefit from grammatical restructuring (e.g., "Lipid-associated Macrophage-Mediated Immunosuppression" or equivalent association-based phrasing). The latter part of the title could also be simplified to improve readability and precision.

Overall, the authors have now satisfactorily addressed the major conceptual and technical concerns raised previously. The remaining issue primarily relate to title refinement, language polishing, presentation clarity, and overall manuscript readability. I therefore support acceptance of the manuscript pending editorial refinement. In my opinion, an additional full round of external review is not necessary once minor issues are addressed.

Author’s response: We thank the reviewer for the positive and constructive assessment of our revised manuscript. We are grateful that the reviewer acknowledges the improvements in clarity, scientific rigor, methodological reporting, clinical annotation, cross-dataset benchmarking, spatial validation and functional validation, and that the major conceptual and technical concerns have been satisfactorily addressed.

We also appreciate the reviewer’s helpful suggestion regarding title refinement. In response, we have revised the title to “Integrated Single-cell Multi-omics Characterization Reveals Lipid-associated Macrophages-mediated Immunosuppression in Neoadjuvant Immunotherapy of Hepatocellular Carcinoma”, as the reviewer suggested.

We have also carefully reviewed the manuscript for language, presentation clarity and overall readability. Minor textual revisions have been made throughout the manuscript to improve clarity and consistency. We thank the reviewer again for the recognition of our research article.
